# Supplementary material for: A phase I clinical trial to evaluate the tolerability and safety of an allogeneic iPSC-derived iNKT cell and α-GalCer-pulsed autologous DC combination therapy for patients with recurrent and advanced head and neck cancer: A study protocol
Source: PLoS One. 2026 Feb 26;21(2):e0342387. doi: 10.1371/journal.pone.0342387 (PMC12944769; doi:10.1371/journal.pone.0342387)
Supplement: S1 File — (PDF) [file pone.0342387.s001.pdf]

Please read carefully

For Patients

A Phase I clinical trial to evaluate the tolerability, safety and efficacy of iPS-NKT cell intra-arterial administration and autologous DC/Gal combination therapy for patients with recurrent and advanced Head and Neck Cancer

～Informed Consent Form～

This document explains a clinical trial focused on the safety, tolerability, and effectiveness of a combined treatment using iPS-NKT cells and autologous DC/Gal for patients with recurrent or advanced head and neck cancer. Please read this document carefully, and make sure you fully understand the trial' s objectives, methods, and the use of cell-based products. Based on this information, you can decide whether to participate in the trial.

If you have any questions or concerns after reading this document or during the doctor’ s explanation, please do not hesitate to ask.

Table of Contents

１．What are clinical trials and regenerative medicine? 4
２．Head and Neck Cancer, iPS-NKT Cells, and DC/Gal Therapy 4
２．１．Standard Treatments for Head and Neck Cancer 5
２．２．What are iPS-NKT Cells? 6
２．３．What is DC/Gal? 8
３．Purpose of the Clinical Trial 8
４．Methods of the Clinical Trial 9
４．１．Eligibility Criteria 9
４．２．Overview of the Clinical Trial Procedures 11
４．３．Dosage 12
４．４．Method of Administration 12
４．５．Examination Schedule 12
４．６．Examinations 15
４．7．Genetic Analysis 16
５．Duration of Participation in the Clinical Trial 18
６．Expected Number of Participants 19
７．Benefits and Risks 19
８．Other Treatment Options 20
９．Withdrawing from the Clinical Trial 21
１０．New Information That May Impact Your Decision 21
１１．Compensation and Medical Treatment for Health Issues Related to the Trial 21
１２．Costs Involved in the Clinical Trial 22
１３．Access to Medical Records and Protection of Privacy 22

１４．Handling of Blood Samples and Test Results..23
１５．Use of Samples for Other Research..24
１６．Conflict of Interest...24
１７．Intellectual Property Rights...25
１８．Freedom to Participate in the Trial...25
１９．Responsibilities of the Participants...25
２０．Trial Review Committees...26
21．Inquiries and Complaints About the Trial..27

1．What are clinical trials and regenerative medicine?

New drugs require approval from the government before being used for the general population. This process involves multiple steps to thoroughly examine the drug’ s effectiveness and safety. Initial steps include laboratory experiments and animal testing to confirm the potential drug’ s effects and safety. After that, trials in humans, including healthy individuals and patients, are conducted to investigate the details of the drug’ s performance and safety. This trial is the first step of the trials called “Phase I trial,” and we will investigate the tolerability and safety of the new medicine. This trial, therefore, does not assure the effectiveness of the new medicine against your disease.

The treatment being investigated in this clinical trial falls under the category of regenerative medicine, which focuses on regenerating or repairing damaged tissues and organs by using living cells. This trial involves the use of iPS-NKT cells, a type of immune cell derived from iPS (induced pluripotent stem) cells. These cells are created in the laboratory by reprogramming adult cells back into a stem-cell-like state. From there, they can be induced to form distinct types of specialized cells, such as NKT cells that are thought to be effective in fighting certain cancers.

Regenerative medicine treatments like this one are subject to Japan’s Act on Ensuring the Safety of Regenerative Medicine. This law mandates strict oversight of regenerative therapies, particularly in terms of their safety and ethical use. For this trial, Chiba University Hospital has submitted the necessary documentation to the Ministry of Health, Labor, and Welfare, and the trial has been approved by the Certified Regenerative Medicine Committee.

The information about conducting this clinical trial at our hospital is publicly available through the Japanese Registry of Clinical Trials (jRCT) and the Chiba University Hospital website.

https://jrct.niph.go.jp/search

2．Head and Neck Cancer, iPS-NKT Cells, and DC/Gal

Therapy

2．1．Standard Treatments for Head and Neck Cancer

Head and neck cancer refers to cancers occurring in the areas between the face and neck, excluding diseases of the brain, spinal cord, and eyes. In recent years, around 10,000 people in Japan are diagnosed with head and neck cancer annually, and the number is increasing.

The head and neck regions house essential functions such as breathing, eating (chewing and swallowing), as well as voice, taste, and hearing, which are critical for daily living. As head and neck cancer progresses, these functions may be impaired, and early treatment is necessary. However, treatments aimed at curing the cancer may also impair these functions, requiring a balance between effectiveness and minimizing side effects.

Recent advancements in endoscopic surgery and radiation therapy have made it possible to maintain treatment outcomes while reducing the burden on patients with early-stage head and neck cancer. However, for patients with advanced or recurrent cancer, combining aggressive surgery, chemotherapy, and radiation therapy has not always produced sufficient results. Many patients, unfortunately, experience a recurrence after these treatments, and surgical intervention becomes difficult. In such cases, systemic chemotherapy is often selected.

For advanced head and neck cancer that cannot be surgically treated, a combination of three drugs (cisplatin, fluorouracil, and pembrolizumab) or pembrolizumab alone is currently used. While these therapies have been shown to extend survival by an average of 2.3 months compared to conventional chemotherapy, they come with challenges such as kidney damage and immune-related adverse effects. Not all patients can tolerate these treatments.

For patients who do not respond to chemotherapy or cannot use chemotherapy, the immunotherapy drug nivolumab may be used. Clinical trials involving nivolumab have shown a 2.4-month survival extension compared to conventional chemotherapy. However, only about 13% of patients saw tumor shrinkage with nivolumab, highlighting the limited effect of this therapy. As a result, there is an urgent need to develop new treatments that are both effective and safe for patients with recurrent or advanced head and neck cancer.

2．2．What are iPS-NKT Cells?

NKT cells are a type of lymphocyte known to have strong anti-cancer activity. They are expected to demonstrate effectiveness against cancer cells that were previously difficult to target. In two previous clinical trials using NKT cells for patients with recurrent or advanced head and neck cancer, a tumor suppression effect was observed. However, because NKT cells exist in small quantities in the blood, it was difficult to expand them efficiently outside the body and secure enough cells for treatment.

With recent advancements in iPS cell technology, it has become possible to create iPS cells from NKT cells (known as NKT-iPS cells) and further derive large quantities of NKT cells from these iPS cells (called iPS-NKT cells). These iPS-NKT cells can be delivered into the arteries that supply nutrients to head and neck tumors, making this a promising treatment method.

NKT-iPS cells are derived from the lymphocytes of a healthy adult donor, with the NKT cells expanded in vitro and stored as iPS cells. These stored iPS-NKT cells are further induced and expanded when needed for treatment. During the process, mouse-derived cells are used in the culture phase. When you participate in this clinical trial, we will use iPS-NKT cells that have been expanded and induced from these stored cells.

After being introduced into the body and attacking the tumor,

these iPS-NKT cells are expected to be eliminated by your immune system. The entire process is designed to ensure that no mouse cells remain in the product used for treatment.

2．3．What is DC/Gal?

NKT cells are activated by receiving instructions from a type of immune cell called a dendritic cell (DC) loaded with a substance known as alpha-galactosyl ceramide（α-GalCer). This DC/Gal acts as a stimulator for NKT cells. Therefore, to further stimulate the iPS-NKT cells delivered into the body and enhance their anti-tumor activity, we will administer DC/Gal via nasal mucosal injection.

DC/Gal is produced from your own blood by collecting mononuclear cells (a type of white blood cell) and cultivating them under sterile conditions. After being treated with α-GalCer, these cells are injected into the nasal mucosa. This method of activating NKT cells with DC/Gal has been researched at Chiba University since the early 2000s, and several clinical studies have been conducted. However, this will be the first time that iPS-NKT cells and DC/Gal are used together in humans.

The combination of iPS-NKT cells and DC/Gal is expected to have a more powerful effect than when used individually, which is why we are conducting this clinical trial.

3．Purpose of the Clinical Trial

This clinical trial targets patients with recurrent or advanced head and neck cancer, where complete cure through existing treatments is difficult. The primary purpose is to confirm the safety and effect of injecting iPS-NKT cells into the arteries supplying nutrients to the tumor after administering DC/Gal into the nasal mucosa.

As mentioned in Section 1, this trial does not guarantee the efficacy of this innovative technology for your disease. However, through this study, we aim to assess the effect of the treatment on tumor size and its influence on the immune system.

After starting the trial, the first 20 days focus on confirming

whether the trial product can be safely administered in humans. After Day 20, the focus shifts to monitoring any emerging side effects.

4．Methods of the Clinical Trial

4．1．Eligibility Criteria

Before starting the clinical trial, we will review your medical history and test results to determine if you meet the criteria for participation. Please note that even if you agree to participate, there is a possibility that you may be excluded from the trial based on the results of the preliminary screening.

The clinical trial will involve 2 to 12 patients who meet the following conditions:

a) Eligibility Criteria:

1) Patients with recurrent or advanced head and neck cancer who are unable to undergo standard treatments or who did not benefit from prior treatments. The tumor must be accessible for cell injection and can be evaluated using imaging techniques such as contrast-enhanced CT.
2) At least one month must have passed since the last cancer treatment before the trial begins.
3) The patient must be aged between 20 and 79 at the time of consent.
4) The patient must be in good overall health.
5) The patient must meet certain blood and lung function criteria.
6) The patient must give written consent to participate in the trial.

b) Exclusion Criteria: You will not be able to participate in the trial if you meet any of the following conditions:

1) You have been diagnosed with hepatitis B or C, HIV, or HTLV.
2) You have taken steroids or immunosuppressive drugs for at least two weeks prior to the DC/Gal administration.
3) You are pregnant, breastfeeding, or planning to become pregnant during the trial. Both male and female participants must

agree to use effective contraception during the trial and for 14 days after the final administration of the trial product.

4) You have an autoimmune disease that requires systemic steroid or immunosuppressive therapy.
5) You have a history of immune-related side effects from checkpoint inhibitor therapy.
6) You have uncontrolled diabetes
7) You have a history of severe lung disease.
8) You have a severe heart condition.
9) You have an active malignancy that requires treatment for at least 2 years prior to enrollment in this trial.
10) You are allergic to contrast-enhancement, human serum albumin or animal-derived proteins.
11) You are currently participating in or have recently participated in another clinical trial that may interfere with this study.
12) You have completely the same type of HLA-A, B and C as the iPS-NKT cells used in this clinical trial.
13) You have been deemed ineligible for component blood collection.
14) The lead investigator or attending physician considers you unsuitable for participation in this clinical trial for any other reason not covered in the listed criteria.

4．2．Overview of the Clinical Trial Procedures

The trial will begin with various tests to determine whether you can participate. If you are eligible, we will collect mononuclear cells from your blood to produce autologous DC/Gal. After that, you will be hospitalized, and DC/Gal will be administered via nasal mucosal injection, followed by the injection of iPS-NKT cells into the tumor's arterial supply.

The first 14 days after the administration of iPS-NKT cells will be dedicated to assessing the tolerability of the trial product (referred to as the ˝DLT period˝). The dose of iPS-NKT cells will be divided into two levels: the first dose level (3×10^7 cells/m^2) and the second dose level (1×10^8 cells/m^2). If the first dose is well-tolerated, we will move to the second dose.

After completing the injection of iPS-NKT cells, we will observe you for four weeks to confirm the safety of the treatment. Depending on your condition, tumor biopsies may be taken before and after treatment, under local or regional anesthesia, with your consent.

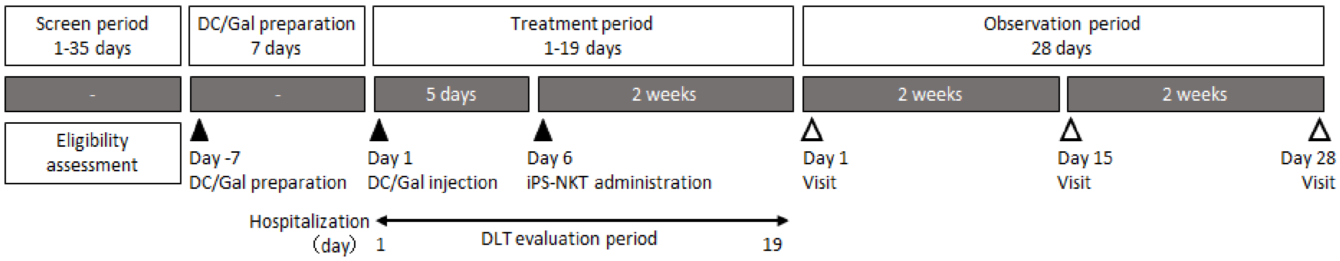

Fig.1. Study Outline

4．3．Dosage

The dosage of the trial products in this clinical trial is as follows:

iPS-NKT cells:
3×10^7 cells/m^2 or 1×10^8 cells/m^2（depending on body size）
DC/Gal: 1×10^8 cells per administration

4．4．Method of Administration

- DC/Gal Administration:
  DC/Gal will be injected directly into the nasal mucosa using a fine needle.
- iPS-NKT Cell Administration:
  iPS-NKT cells will be administered directly into the tumor-supplying arteries. To do this, a thin tube called a catheter will be inserted into the artery using the Seldinger technique.

In the Seldinger technique, a catheter will be inserted into an artery (usually in the groin or arm) and guided to the tumor-supplying artery. Once the catheter reaches the targeted artery, iPS-NKT cells will be injected. After the injection, the catheter will be removed.

Possible side effects of the DC/Gal administration include mild pain and minimal bleeding at the injection site. For iPS-NKT cell administration using the Seldinger technique, potential risks include kidney damage or allergic reactions to the contrast agent, bleeding or hematoma formation at the puncture site, and infection at the surgical site or from the catheter. Serious but rare complications include blood clots dislodging during the procedure and blocking blood vessels in other organs, leading to conditions such as stroke or heart attack.

4．5．Examination Schedule

This trial will involve the following tests according to the schedule outlined in the tables provided in the document.

Table1. Examination Schedule

		Screen period	Cell Preparation	Study Therapy Period						Observation Period[j]			Termination
Day ※Day 1 means the start day of study treatment		−42～−8	-7	1	6	7	8	13	19	1	15	28	—
Allowance (day)				+3	+3	—	—		±3	±3	±7	±7	+7
Informed Consent	●												
Patient Demographics		●											
Past Medical History		●											
Pregnancy Test[a]		●										●	●
Infection Examination[b]		●											
Chest X-ray		●						●		●	●	●	●
Electric Cardiogram		●											
CT or MRI		●								)	●[i]		●
DC/Gal Preparation			●										
DC/Gal Administration				●									
iPS-NKTAdministration					●								
Blood Test		●		●	●			●		●	●	●	●
Urine Test		●		●	●					●	●	●	●
General Health Status[d]		●	●	←―――	―――	―――	―――	―――	―――→				
mMRC				●	●								
PK/PD[e]					●	●		●					
Immune Cell Function Test[f]				●	●		●	●		●			
Adverse Events Evaluation		←―――	―――	←―――	―――	―――	―――	―――	―――→	―――	―――	―――	―――→
DLT Evaluation													
Hospital admission[g]				←―――	―――	―――	―――	―――	―――	―――→			
HLA Test		●											
Anti-HLA Antibody Test		●			●					●			●
Optional Tumor Biopsy[h]				●	●								

(a) Perform only in women of childbearing potential. Perform a urine qualitative test.

(b) Infectious disease tests include HIV antibody, HTLV-1 antibody, HBs antigen, HBs antibody, HBc antibody, and HCV antibody. If the HBs antigen is negative and the HBs antibody test or HBc antibody test is positive, the HBV-DNA quantitative test is performed. If the HCV antibody test is positive, the HCV-RNA quantitative test is performed.

(c) Secure the route of administration of the investigational product 1 week prior to the first administration date (allowance based on 1 week prior to the first administration date: -14 to +7 days). As described in the Administration Method section, there are two methods of administering the investigational product (method of securing the route of administration): the port method (e.g., for maxillary cancer) or the Selsinger method. In the case of the port method, a port is implanted under local anesthesia in an IVR room, and the investigational product is administered through the implanted port after confirming that there are no signs of infection in the wound. In the case of the Selsinger method (tongue cancer, etc.), the tip of the catheter is guided into the tumor-feeding artery in the IVR room, and the investigational product is administered directly.

(d) Vital signs shall include body temperature, pulse rate, respiratory rate, SpO2 , and blood pressure (systolic and diastolic) , and additional tests (chest X-ray, hematological and serological tests, blood gas tests, etc.) may be added as necessary based on the judgment of the PI or SI .
(e) The blood collection schedule for PK/PD and blood cytokine measurements will be as shown in Table 20
(f) Isolate PBMCs from blood samples and stain for cell surface markers.
(g) During the DLT evaluation period (from the first dose to Cycle 2 Day 14) and for 7 days after the third dose of the investigational product, the subject will be carefully monitored for safety by hospitalization in principle. In addition, the results of laboratory tests and medical examinations after the seventh day of administration of the investigational product (Day 8 of each cycle) should show that no Grade 2 or higher non-hematological toxicity or Grade 3 or higher hematological toxicity has occurred, and that the site and home health care facility can be contacted in the event of an adverse event and that the patient can be contacted promptly in an emergency. The PI or SI may stay overnight or leave the hospital at his/her own discretion only when a system is in place to contact the site and home health care facility in the event of an adverse event and to contact the patient promptly in an emergency.
(h) Tumor biopsy will be performed before administration of auto-DC/Gal and/or within 2 days of iPS-NKT cell administration only in subjects who are judged by the PI or SI to be capable of biopsy from the tumor under infiltration or local anesthesia, and who have given consent for tumor biopsy.
(i) Cycle 1 Day 14 contrast CT scan (tumor evaluation) will be performed prior to Cycle 2 transition.
(j) If the administration of the investigational product is discontinued, the observation period will begin at the later of the end of the cycle of administration of the investigational product immediately prior to discontinuation (2 weeks after the last administration of the investigational product) or the date on which the decision to discontinue administration was made.

Table2　Schedule for PK/PD

	Before DC/Gal administration	Before iPS-NKT administration	After iPS-NKT administration		
Hour	—	—	1	24	168
Allowance (minutes)	—	—	±15	±30	±60
Blood collection for PK/PD	—	●	●	●	●

4．6．Examinations

After you agree to participate in this clinical trial, screening tests will begin to determine your eligibility. The tests performed during the clinical trial are described below, and the schedule for these tests is detailed in Table 1 (observation items and test schedule) and Table 2 (blood collection schedule for pharmacokinetics/pharmacodynamics (PK/PD) testing).

1） Screening period

During the screening period, the following tests and evaluations will be conducted to determine if you meet the inclusion criteria and if any of the exclusion criteria apply:

・Medical History
・Physical Examination
・General Health Status
・Blood test About 15 mL of blood will be collected for the following tests:
Red blood cell count, hemoglobin, hematocrit, platelet count, total protein, albumin, total bilirubin, AST, ALT, ALP, LDH, BUN, creatinine, uric acid, sodium, potassium, chloride, calcium, phosphorus, amylase, HbA1c, and a white blood cell count with differential.
・Urine Tests: Evaluation of urine pH, glucose, protein, urobilinogen, ketones, and occult blood.

・Coagulation Tests: About 5 mL of blood will be collected to assess blood clotting ability.
・Chest X-ray
・Electrocardiogram (ECG)
・Contrast-Enhanced CT (or MRI) Scans
・Pregnancy Test
・Infection Screening
・HLA Typing
・HLA Antibody Testing

2）Treatment Phase (Trial Product Administration)
- Physical Examination
- General Health Status
- Blood test
- Urine Tests
- Coagulation Tests
- Contrast-Enhanced CT (or MRI) Scans
- Pharmacokinetic/Pharmacodynamic (PK/PD) Blood Tests
- Immune Cell Function Tests: About 20 mL of blood will be collected to analyze the function of immune cells during the trial.
- Chest X-ray
- Evaluation of Adverse Events
- HLA Antibody Testing
- Optional Tumor Biopsy

3）Observation Phase and End of Trial
- Physical Examination
- General Health Status
- Blood test
- Urine Tests
- Coagulation Tests
- Contrast-Enhanced CT (or MRI) Scans
- Immune Cell Function Tests
- Chest X-ray
- Pregnancy Test
- Evaluation of Adverse Events
- HLA Antibody Testing

Blood Sample Volumes

In total, approximately 5-45 mL of blood will be collected during each blood test, and tests will be conducted ten times throughout the clinical trial

4．7．Genetic Analysis

As part of this clinical trial, we may collect your blood samples for

genetic analysis. The purpose of this analysis is to evaluate the effect of iPS-NKT cell therapy on your immune system and to gain a better understanding of how the treatment works. Genetic analysis will only be performed with your separate consent and is entirely optional.

If you choose not to participate in the genetic analysis, you can still participate in the trial. Your refusal or withdrawal of consent will not affect your treatment or how you are treated by the trial team.

Genetic testing involves analyzing the DNA from your blood to understand how the immune cells function. Your genetic information will be anonymized and protected to ensure privacy.

＜Expected Benefits and Risks of Genetic Analysis＞

The results of the genetic analysis are unlikely to directly benefit your current treatment. However, this analysis may yield vital information that can contribute to future cancer treatments, especially for head and neck cancer patients. By studying how iPS-NKT cells work in your immune system, researchers may develop more effective treatments for others.

Since the genetic analysis will use leftover blood from the clinical trial, no additional samples will need to be collected, minimizing physical burden. Any risks associated with this analysis are minimal.

＜Access to Genetic Information and Privacy＞

If you wish, you can request access to the materials related to the genetic analysis being performed. Throughout the genetic analysis process, your personal information will be pseudonymized, meaning that your identity will be protected, and the samples will be labeled in a way that does not easily reveal your identity.

＜Sample Handling and Disposal＞

Once the genetic analysis is complete, any remaining samples and related data will be managed according to the guidelines mentioned in Section 14 of this document. These guidelines ensure that your

samples are stored securely, and any unused samples will be safely disposed of in compliance with privacy and safety regulations.

＜Disclosure of Genetic Test Results＞

Even if genetic differences are found during this research, they may not have immediate or definitive implications for your health. As a result, we do not plan to provide you with the results of the genetic analysis. However, if the research reveals findings that could have significant implications for your health or that of your relatives (such as a predisposition to a serious illness), we may contact you with that information following review by the Ethics Committee.

＜Use of Genetic Test Results＞

The results of this genetic analysis, including your genetic information, will be used to further the aims of this clinical trial. However, your identity will remain confidential, and no personal information will be disclosed.

＜Publication and Sharing of Results＞

The results of this genetic analysis, along with the findings from the clinical trial, may be shared in scientific publications, presented at academic conferences, or included in public databases. These results will be shared in a way that ensures your anonymity and privacy are maintained.

＜Ownership and Intellectual Property＞

If any patents or intellectual property arise from this research, the ownership will belong to the researchers or their institutions. As a participant, you will not have any claim to the intellectual property or any financial compensation.

5．Duration of Participation in the Clinical Trial

The estimated duration of participation in this clinical trial is approximately 2.5 months. This includes the pre-observation period (about 5 weeks), the treatment period (about 2 weeks), and the post-

treatment observation period (about 4 weeks).

6. Expected Number of Participants

This trial will be conducted solely at Chiba University Hospital, with an expected total of 2 to 12 patients with head and neck cancer participating.

7. Benefits and Risks

Benefits:

Preclinical studies using animals and other models have shown that DC/Gal and iPS-NKT cells have anti-cancer effects. In mice transplanted with human head and neck cancer cells, the treatment was effective in suppressing tumor growth.

Although we cannot guarantee the treatment's effectiveness in your case, the combination of DC/Gal and iPS-NKT cells may help reduce the size of your tumor or slow its progression.

Furthermore, the results of your treatment may contribute to the development of new therapies for head and neck cancer and potentially other types of cancer in the future.

Risks:

Possible adverse reactions from the combination therapy of DC/Gal and iPS-NKT cells include side effects that may arise due to the novel nature of this treatment. Although no significant safety concerns were observed in preclinical studies, it is difficult to predict the full range of possible side effects in humans.

In previous clinical trials using NKT cells in patients with head and neck cancer, mild side effects such as fever, dehydration, back pain, lymphopenia, headache, fatigue, and dizziness were reported.

When DC/Gal was administered nasally in combination with NKT

cells, eighteen patients were treated across two trials. One serious adverse event, a fistula (an abnormal connection) between the throat and skin, occurred as the tumor shrank. Other patients did not experience significant side effects.

In a clinical trial using nasal DC/Gal monotherapy, one patient out of twenty-three unfortunately died due to pneumonia, which was potentially unrelated to the treatment. In a different trial involving lung cancer patients, one out of seventeen patients developed a blood clot, requiring hospitalization (the clot was deemed unlikely to be related to the cell therapy).

There are potential unknown risks involved in using biological products, such as the possible presence of undetected viruses in the cells, or immune responses to foreign proteins used in cell production. Should any of these complications arise, we will promptly administer appropriate treatments like antibiotics or antiviral drugs.

This trial also involves the use of fetal bovine serum during the cell production process, which may cause an allergic reaction in some individuals. Additionally, other serious side effects have been reported when several types of immune cells were used in combination with immune checkpoint inhibitors, with six cases of severe adverse events, including one death, reported.

Finally, you may experience inconvenience due to the increased number of tests and blood draws compared to standard treatments. If you agree to tumor biopsy procedures, these may cause some pain or bleeding, though pain relief and treatment will be carefully provided.

８．Other Treatment Options

If you choose not to participate in this clinical trial, other treatment options may include conventional chemotherapy or participation in other clinical trials. However, these options are not guaranteed to be more effective or safer.

９．Withdrawing from the Clinical Trial

Even if you consent to participate in this trial, you may be excluded or asked to withdraw under the following circumstances:

1) Before DC/Gal Administration:
   If tests reveal that you do not meet the inclusion criteria, you will not be able to proceed with the trial.

2) After DC/Gal Administration:
   The trial may be stopped if your symptoms worsen, test results show significant changes, or side effects arise that require another treatment. You are also free to withdraw at any time for personal reasons. Additionally, the trial may be halted if the trial doctor deems it necessary for safety or other reasons.

If the trial is stopped, we will explain the reasons and continue to monitor your safety.

１０．New Information That May Impact Your Decision

If any added information comes to light that may affect your decision to continue participating, we will promptly inform you. You can then decide whether you wish to remain in the trial.

１１．Compensation and Medical Treatment for Health Issues Related to the Trial

Although this trial has been carefully designed, if any health issues arise due to your participation, we will provide the best possible treatment, based on your health insurance coverage. In case of death or serious disability resulting from adverse effects, compensation will be provided based on criteria from the Pharmaceutical Side Effects Relief System. However, compensation will not be available if the issue arises from your own intentional misconduct or serious negligence.

１２．Costs Involved in the Clinical Trial

１）Medical Costs During the Trial:

The cost of the investigational product and the catheter placement for administering it, as well as the tests (blood tests, imaging, PK/PD, etc.) and hospitalization required during the trial, will be covered by the research budget. Therefore, there will be no cost to you for participating in the trial.

However, if you experience side effects requiring additional medical treatment or if costs arise from treating existing medical conditions, those will be handled through regular health insurance, and a portion of the costs will be your responsibility.

２）Reimbursement for Participation:

Participation in this trial may require more visits to the hospital than usual, which may increase transportation costs and other burdens. To help offset this, you will be reimbursed ¥20,000 per hospitalization and ¥7,000 per visit. These payments will be transferred to the bank account you specify.

Additionally, if you receive treatment at a hospital other than Chiba University Hospital, we may contact the other hospital to obtain your medical information. In such cases, there may be additional costs for sharing medical records, which may also be your responsibility.

１３．Access to Medical Records and Protection of Privacy

The personal information of trial participants will be strictly protected and will not be disclosed externally. The data collected from patients will be assigned a code number to ensure that it cannot be easily linked to your identity (a process called ″pseudonymization″).

Since this trial is a collaborative study with the RIKEN research institute, the data collected (clinical data, blood test results,

pharmacokinetic analyses, HLA typing, immune function test results, etc.) will be shared with RIKEN in anonymized form for analysis. Additionally, to assess the safety and effectiveness of the investigational product in greater detail, data from the trial may be submitted to external committees. The results of the trial may also be presented at conferences, published in scientific journals, or included in public databases, but personal information will be protected, and your name and identity will not be revealed.

Representatives of regulatory authorities, such as the Ministry of Health, Labor, and Welfare, and representatives from organizations involved in the development of the investigational product may also review your medical records to ensure that the trial is being conducted properly. This includes reviewing medical records from before the trial started. All these parties are obligated to maintain confidentiality, and your privacy will be protected.

If you receive treatment at another hospital during the trial, we may contact the treating physician to obtain information about your treatment and condition. By signing the consent form at the end of this document, you will agree to allow access to your medical records and the sharing of information with other medical institutions.

You may withdraw your consent for the use of your medical information at any time. However, this will also mean that you can no longer participate in the trial. If you wish to withdraw your consent, please inform the trial doctor or the hospital staff. Even if you withdraw consent, the data collected up to that point will still be used in accordance with the guidelines mentioned above.

１４. Handling of Blood Samples and Test Results

Blood samples collected during the trial will be pseudonymized. Data obtained from the trial may be shared with pharmaceutical companies for drug development purposes. In such cases, the data will be shared in pseudonymized form.

Blood samples collected for PK/PD and HLA antibody testing will be frozen and stored at Chiba University Hospital's Future Creation Center during the trial. These samples will be tested during the trial period, and any remaining samples will be stored for 10 to 30 years under secure conditions to prevent mix-ups, loss, or theft.

With your separate consent, the samples may also be used for genetic analysis to investigate how iPS-NKT cells function immunologically or to study the characteristics of individuals who respond well to the treatment. You are free to withdraw your consent for the use of your samples at any time, and this will not affect your participation in the trial.

This genetic analysis will be conducted in compliance with relevant laws and approved by the Ethics Committee. In the event of sample disposal, all precautions will be taken to ensure the safe handling and confidentiality of your information.

１５．Use of Samples for Other Research

If you agree, the blood samples you provide for this trial may also be used for future research. These samples will be stored in a anonymized state at Chiba University Hospital's Future Creation Center until March 31, 2037, and may be used in other studies approved by the Ethics Committee. This research may be conducted both at Chiba University and external research institutions. If used for other research, your samples will be shared in anonymized form.

Once the samples are provided, they become the property of the research institution, and you will not retain ownership of them.

１６．Conflict of Interest

This trial is funded by the Japan Agency for Medical Research and Development (AMED). The Conflict-of-Interest Committee at

Chiba University Hospital has reviewed the trial to ensure that there are no conflicts of interest that could distort the interpretation of the results or compromise patient safety.

What is a conflict of interest? A conflict of interest occurs when there is a relationship between the researchers or institution conducting the trial and a company or organization that has a stake in the outcome. For example, if a pharmaceutical company provides funding or other support for the trial, this could be considered a conflict of interest. To prevent this from affecting the integrity of the trial, all conflicts of interest must be disclosed and managed.

１７．Intellectual Property Rights

If any patents or intellectual property rights arise from this trial, they will belong to the researchers or their affiliated institutions. You, as a participant, will not have any rights to these patents or intellectual property.

１８．Freedom to Participate in the Trial

Participation in this trial is entirely voluntary. You have the right to decide whether to participate in the trial based on the information provided. Even after giving consent, you are free to withdraw from the trial at any time without penalty or loss of benefits. Your decision not to participate, or to withdraw, will not affect the quality of medical care you receive.

If you choose to withdraw from the trial, the data collected until that point will still be used in research, but no new data will be collected after your withdrawal.

１９．Responsibilities of the Participants

If you agree to participate in the trial, you are expected to:

1) Follow the trial schedule as instructed by the trial doctor and staff.
2) Inform your trial doctor if you are receiving treatment from another medical department or hospital, or if you are taking any medications. We may contact your other healthcare providers to coordinate your care during the trial.
3) Consult with the trial doctor before taking any over-the-counter medications or supplements during the trial.
4) Undergo all required tests and check-ups to monitor your health and detect any potential side effects.
5) Report any changes in your health condition to the trial doctor or staff, especially if you experience any unusual symptoms.
6) Follow the guidance on contraception if applicable, as outlined in Section 4.1 (b) (3), to avoid pregnancy during the trial and for 14 days after the last administration of the trial products
7) Inform your trial doctor or coordinator if you or your partner have a possibility of pregnancy during the study period.
8) Follow the instructions from your trial doctor if any.

２０. Trial Review Committees

This clinical trial has been reviewed and approved by two separate committees to ensure that it is scientifically valid, safe, and ethical. These committees also ensure that the rights and well-being of participants are protected.

1) Chiba University Specified Authorized Regenerative Medicine Committee
   Established by the Director of Chiba University Hospital
   Address: 1-8-1 Inohana, Chuo-ku, Chiba City, Chiba 260-8677

2) Chiba University Hospital Ethics Committee
   Established by the Director of Chiba University Hospital
   Address: 1-8-1 Inohana, Chuo-ku, Chiba City, Chiba 260-8677
   URL: https://www.ho.chiba-u.ac.jp/crc/committee/erb2.html

Even after the trial has started, these committees will continue to monitor the trial. If there are any changes to the trial protocol or if significant new safety information becomes available, the committees will review the situation and decide whether the trial should continue.

The results of the trial, including any changes to the protocol or safety data, will be made publicly available through the Japanese Registry of Clinical Trials (jRCT) and the Chiba University Hospital website.

21．Inquiries and Complaints About the Trial

If you have any questions or concerns about this clinical trial, or if you wish to withdraw from participation, please contact your trial doctor or the hospital staff.

Consent Form

I got sufficient explanations and confirmed the contents written below about the clinical trial titled "A Phase I clinical trial to evaluate the tolerability, safety and efficacy of the iPS-NKT intra-arterial administration and autologous DC/Gal for patients with recurrent and advanced Head and Neck Cancer." And I agreed with the participation based on my own decision. Also, I received a copy of this informed consent form with signatures.

- What are clinical trials and regenerative medicine?
- Head and Neck Cancer, iPS-NKT Cells, and DC/Gal Therapy
- Purpose of the Trial
- Methods of the Trial
- Duration of Participation in the Trial
- Expected Number of Participants
- Benefits and Risks
- Other Treatment Options
- Withdrawing from the Trial
- New Information That May Impact Your Decision
- Compensation and Medical Treatment for Health Issues Related to the Trial
- Costs Involved in the Trial
- Access to Medical Records and Protection of Privacy
- Handling of Blood Samples and Test Results
- Conflict of Interest
- Intellectual Property Rights
- Freedom to Participate in the Trial
- Responsibilities of the Participants
- Trial Review Committees
- Inquiries and Complaints

Genetic analysis using residual samples

☐ Agree

☐ NOT Agree

Prolonged Storage and usability for future research of your Samples

☐ Agree

☐ NOT Agree

Optional Tumor Biopsy

☐ Agree

☐ NOT Agree

Reimbursement for Participation

☐ Agree

☐ NOT Agree

(Please check one box in each item)

Date of Consent (MM/DD/YYYY)：　　　/　　　/

Signature：

Trial doctor　Date of Explanation (MM/DD/YYYY)：　　　/　　　/

Signature (Doctor)：

Signature (Coordinator)：
