# Supplementary material for: A phase I clinical trial to evaluate the tolerability and safety of an allogeneic iPSC-derived iNKT cell and α-GalCer-pulsed autologous DC combination therapy for patients with recurrent and advanced head and neck cancer: A study protocol
Source: PLoS One. 2026 Feb 26;21(2):e0342387. doi: 10.1371/journal.pone.0342387 (PMC12944769; doi:10.1371/journal.pone.0342387)
Supplement: S5 File — (PDF) [file pone.0342387.s005.pdf]

研究計画書

再発・進行頭頸部がん患者を対象とした iPS-NKT 細胞動注療法及び自家 DC/Gal 併用療法の忍容性、安全性及び有効性に関する第 I 相試験

第一種再生医療等技術

研究計画書番号：CUH_ iPSNKT_DCGal_001

版数	**第 1.8 版**
作成日	**2023 年 3 月 7 日**

改訂履歴

作成日	版数	改訂理由／内容
2022.03.22	1.0	新規作成
2022.03.28	1.1	誤記修正、類似製品の臨床試験結果追記
2022.04.16	1.2	投与法におけるポート法削除、用語統一、記載整備
2022.06.01	1.3	非臨床有効性データ追加、iPS-NKT 細胞投与数変更、用語統一、誤記修正
2022.06.12	1.4	単位記載統一、誤記修正
2022.08.29	1.5	誤記修正
2022.10.17	1.6	再生医療等評価部会からの指摘に伴う修正
2023.2.6	1.7	再生医療等評価部会からの指摘に伴う修正
2023.3.7	1.8	再生医療等評価部会からの指摘に伴う修正

＜目次＞

0. 研究の概要 11
1. 緒言 17
1.1. 試験の背景 17
1.1.1. 頭頸部がんと治療の現状について 17
1.1.2. NKT 細胞免疫療法について 17
1.1.3. NKT 細胞免疫療法における課題と iPS 細胞の応用 18
1.1.4. iPS 細胞と自家 DC/Gal との併用 18
1.2. iPS-NKT 細胞及び DC/Gal について 19
1.2.1. iPS-NKT 細胞及び DC/Gal の概要 19
1.2.1.1. iPS-NKT の概要 19
1.2.1.2. 自家 DC/Gal の概要 20
1.2.2. iPS-NKT 細胞及び DC/Gal の製造 20
1.2.2.1. iPS-NKT 細胞の製造 20
1.2.2.2. iPS-NKT 細胞におけるドナー選別規格試験（**表 1**） 23
1.2.2.3. NKT-iPS 細胞選択規格試験（**表 2**） 23
1.2.2.4. NKT-iPS 細胞 MCB/WCB 規格試験（表 3 表 4） 24
1.2.2.5. iPS-NKT 細胞（凍結）規格試験（表 5） 25
1.2.2.6. iPS-NKT 細胞（製品）規格試験（表 6） 25
1.2.2.7. iPS-NKT 細胞（製品）の安定性（表 8） 27
1.2.2.8. 自家 DC/Gal の製造 30
1.2.2.9. 自家 DC/Gal の規格試験 31
1.2.2.10. 自家 DC/Gal の安定性試験 32
1.2.3. 動物実験における有効性の検証 33
1.2.3.1. iPS-NKT 細胞の抗腫瘍効果（K562 細胞） 33
1.2.3.2. iPS-NKT 細胞の抗腫瘍効果（FaDu 細胞） 33
1.2.3.3. iPS-NKT 細胞の NK 細胞活性化能（アジュバント活性） 34
1.2.3.4. DC/Gal と iPS-NKT 細胞の併用 35
1.2.3.5. in vivo 抗腫瘍効果および腫瘍内免疫細胞解析（DC/Gal 併用、ヒト肺がん PDX） 36
1.2.4. 一般毒性試験及び造腫瘍性試験 37
1.2.4.1. iPS-NKT 細胞の一般毒性試験 37
1.2.4.2. DC/Gal の一般毒性試験 38
1.2.4.3. 併用投与毒性試験（NKT-deficient B6, ヒト型 IL7/15 発現 NSG マウス） 38
1.2.4.4. 造腫瘍性試験 38
1.2.5. 薬理試験、動態試験 41
1.2.5.1. iPS-NKT 細胞の IFN- γ及び IL-4 産生能 41

1.2.5.2. iPS-NKT 細胞の *in vitro* 抗腫瘍効果 42
1.2.5.3. iPS-NKT 細胞の動態試験 42
1.2.6. 類似製品の臨床試験（有効性と有害事象） 45
1.2.7. 予想される副作用 48
1.3. 本試験実施が妥当であると判断した理由 48
1.3.1. 初回投与量及び用法の妥当性 48
1.3.2. 対象集団の妥当性 49
1.3.3. 評価方法の妥当性 49
1.3.4. 被験者安全性の確保 49
2. 試験の目的と必要性 50
3. 対象患者 50
3.1. 選択基準 50
3.2. 除外基準 52
4. 被験者の同意 53
4.1. 同意文書及びその他の説明文書の作成並びに改訂 53
4.2. 同意取得の時期と方法 53
4.3. 被験者に対する説明事項 54
5. 試験の方法 55
5.1. 試験のデザイン 55
5.2. 用量制限毒性（DLT） 56
5.3. 目標被験者数と試験実施期間 57
5.4. 施設登録及び症例登録 57
5.4.1. 施設登録 57
5.4.2. 症例登録 57
5.4.3. 症例登録先 58
5.5. 登録されなかった被験者の取り扱い 58
5.6. 投与スケジュール及び投与量・投与方法 58
5.7. 投与開始基準 58
5.8. 投与延期基準 58
5.9. 個々の症例における投与中止基準及び試験中止基準 59
5.9.1. 個々の症例における投与中止基準 59
5.9.2. 個々の症例における試験中止基準 59
5.10. 併用薬及び併用療法 60
5.10.1. 併用可能薬・併用可能療法 60
5.10.2. 併用薬・併用療法の記録 60
5.10.3. 併用禁止薬及び併用禁止療法 60

5.11. 試験中止又は終了後の対応 60
6. 特定細胞加工物 61
6.1.1. iPS-NKT 細胞の包装・表示 61
6.1.2. DC/Gal の包装・表示 61
6.2. 管理・調剤方法 62
6.2.1. iPS-NKT の管理・調剤方法 62
6.2.2. DC/Gal の管理方法 62
7. 観察・検査・評価項目、方法及び実施時期 62
7.1. 実施スケジュールと手順 62
7.1.1. スクリーニング検査 63
7.1.2. 被験者の情報 63
7.1.3. 観察・検査・評価項目 64
7.1.3.1. 細胞準備期（Day -7） 65
7.1.3.2. 試験治療期 65
7.1.3.3. 観察期 66
7.1.3.4. 試験中止時 66
7.1.3.5. 腫瘍評価 67
7.1.3.6. 妊娠の転帰調査 67
7.1.3.7. 研究終了後のフォローアップ 67
8. 有害事象・品質不良等発生時の取り扱い 67
8.1. 有害事象・品質不良の定義 67
8.2. 疾病等の定義 68
8.3. 重篤な有害事象の定義 68
8.3. 有害事象又は品質不良の発生時の被験者への対応 68
8.4. 報告の対象となる有害事象及び品質不良 69
8.5. 有害事象及び品質不良発生時の報告手順 69
8.6. 有害事象及び品質不良の評価に必要な記載内容 69
8.6.1. 有害事象 69
8.6.2. 品質不良 69
8.6.3. 有害事象の回復性と DC/Gal 又は iPS-NKT 細胞との因果関係 70
8.7. 疾病等の発生の場合の措置 70
8.8. 特定認定再生医療等委員会及び厚生労働大臣への疾病等報告 70
8.9. 特定認定再生医療等委員会及び厚生労働大臣への定期報告 71
8.10. 厚生労働大臣への重大事態報告 71
8.11. 不適合の管理 72
9. 評価項目 72

9.1. 主要評価項目 72
9.2. 副次評価項目 72
9.3. 探索的評価項目 73
9.4. 免疫学的評価項目 73
10. 統計学的事項 74
10.1. 解析対象集団 74
10.1.1. 安全性解析対象集団及び DLT 評価対象集団 74
10.1.2. 最大の解析対象集団（full analysis set：FAS） 74
10.1.3. 研究計画書に適合した対象集団（per protocol set：PPS） 74
10.2. 目標症例数と設定根拠 74
10.3. 症例の取り扱い 75
10.4. データの取り扱い 75
10.5. 統計解析項目及び解析計画 75
10.5.1. 被験者背景の解析 76
10.5.2. 安全性及び有効性の解析 76
10.5.2.1. 主たる解析 76
10.5.2.2. 副次解析 76
10.5.3. 中間解析 76
10.6. データモニタリング委員会 76
10.7. 最終解析 77
11. 研究計画書の遵守及び逸脱 77
12. 研究計画書、症例報告書又は解析計画に関する変更 77
12.1. 研究計画書及び症例報告書の改訂 77
12.2. 統計解析計画の変更 78
13. 試験の中止、中断又は終了 78
13.1. 試験全体での中止又は中断の基準 78
13.2. 試験全体での中止又は中断する場合の手続き 78
13.3. 試験の終了 78
14. データマネジメント 78
14.1. データマネジメントの手順 78
14.2. データの収集 79
14.3. 症例報告書の直接記載され、かつ原資料（原データ）を解すべき資料の特定 79
15. 原資料及びその他の記録の保存 79
15.1. 試験実施医療機関による記録の保存 79
15.2. 実施責任者による記録の保存 80
16. 採取した細胞の一部等と再生医療等に用いた細胞加工物の一部の保存期間 80

16.1. 試料の保存 80
16.2. 検体の廃棄 80
16.3. データの利用 80
16.4. 試料及びデータの再利用 80
17. 原資料の直接閲覧 80
18. 試験の品質管理及び品質保証 81
18.1. 品質管理 81
18.2. 品質保証 81
19. 倫理及び再生医療等法 81
20. 審査する委員会 82
21. 健康被害補償及び保険 82
22. 試験に関する費用負担 82
23. 研究資金及び利益相反 82
24. 試験のデータベース登録 83
25. 試験実施体制 83
26. 参考資料・文献リスト 83

略語・用語集

略号･略記･用語	定義	
ALP	alkaline phosphatase	アルカリフォスファターゼ
ALT	alanine aminotransferase	アラニンアミノトランスフェラーゼ
AMED	Japan Agency for Medical Research and Development	日本医療研究開発機構
aPTT	activated partial thromboplastin time	活性化部分トロンボプラスチン時間
AST	aspartic aminotransferase	アスパラギン酸アミノトランスフェラーゼ
BUN	blood urea nitrogen	血中尿素窒素
CDDP	cisplatin	シスプラチン
CI	confidence interval	信頼区間
Cmab	cetuximab	セツキシマブ
CMV	cytomegalovirus	サイトメガロウイルス
CT	computed tomography	コンピューター断層撮影
CTCAE	common terminology criteria for adverse events	有害事象共通用語規準
DC	dendritic cell	樹状細胞
DC/Gal	αGalCer pulsed DC	αGalCer 刺激後樹状細胞
CTL	Cytotoxic T lymphocyte	細胞障害性 T リンパ球
DLT	dose limiting toxicity	用量制限毒性
EBV	Epstein-Barr virus	エプスタイン・バー・ウイルス
ECOG	Eastern Cooperative Oncology Group	米国東海岸癌臨床試験グループ.
EDC	electronic data capture	電子的臨床検査情報収集
ELISA	enzyme-linked immuno sorbent assay	酵素免疫測定法
ELISpot	enzyme-linked immunoSpot	酵素免疫スポット法
ER/ES	electronic records/electronic signature	電子記録/電子署名
FACS	fluorescence-activated cell sorter	蛍光活性化細胞選別法
FAS	full analysis set	最大の解析対象集団
FDG	2-Deoxy-2-[18F]fluoroglucose	フルオロデオキシグルコース
FIH	first in human	ヒト初回投与
G-CSF	granulocyte-colony stimulating factor	顆粒球コロニー刺激因子
GLP	good laboratory practice	医薬品の安全性に関する非臨床試験の実施の基準に関する省令
GVHD	graft versus host disease	移植片対宿主病
HBV	hepatitis B virus	B 型肝炎ウイルス
HCV	hepatitis C virus	C 型肝炎ウイルス
HIV	human immunodeficiency virus	ヒト免疫不全ウイルス
HLA	human leukocyte antigen	ヒト白血球抗原
HTLV	human T-lymphotropic virus	ヒト T リンパ好性ウイルス
IFN	interferon	インターフェロン

略号･略記･用語	定義	
IL	interleukin	インターロイキン
iPS	induced pluripotent stem cells	人工多能性幹細胞
iPS-NKT		iPS 細胞由来 NKT 細胞
JCOG	Japan Clinical Oncology Group	日本臨床腫瘍研究グループ
LDH	lactate dehydrogenase	乳酸脱水素酵素
MCB	master cell bank	マスターセルバンク
MedDRA/J	Medical Dictionary for Regulatory Activities	ICH 国際医薬用語集/日本語版
MEM	Minimum Essential. Medium	最小必須培地
MHC	major histocompatibility complex	主要組織適合遺伝子複合体
mMRC	modified Medical Research Council	修正息切れスケール
MRI	magnetic resonance imaging	磁気共鳴画像
MTD	maximum tolerated dose	最大耐用量
NE	Not evaluable	評価不能
NK	natural killer （cells）	ナチュラルキラー（細胞）
NKR	natural killer receptor	ナチュラルキラー受容体
NKT	natural killer T （cells）	ナチュラルキラーT（細胞）
NKT-iPS		ドナーNKT 細胞由来 iPS 細胞
NOG	NOD.Cg-Prkdcscid Il2rgtm1Sug/Jic	
NYHA	New York Heart Association	ニューヨーク心臓協会
OS	overall survival	全生存期間
ParvoB19	Parvovirus B19	パルボウイルス B19
PBMC	peripheral blood mononuclear cells	末梢血単核細胞
PBS	phosphate buffered saline	リン酸緩衝生理食塩水
PCR	polymerase chain reaction	ポリメラーゼ連鎖反応
PD	pharmacodynamics	薬力学
PET	positron emission tomography	陽電子放射断像撮影
PK	pharmacokinetics	薬物動態学
PPS	per protocol set	研究計画書に適合した対象集団
PS	performance status	パフォーマンスステータス
PT	preferred terms	基本語
PT-INR	prothrombin time-international normalized ratio	プロトロンビン時間　国際標準比
PTT	partial thromboplastin time	部分トロンボプラスチン時間
QOL	quality of life	生活の質
RECIST	response evaluation criteria in solid tumors	固形がんの治療効果判定のための新ガイドライン
SOC	system organ class	器官別大分類
TCR	T-cell recepter	T 細胞受容体
TD	toxicodynamics	トキシコダイナミクス

略号･略記･用語	定義	
TK	toxicokinetics	トキシコキネティクス
WCB	working cell bank	ワーキングセルバンク
WNV	West Nile Virus	ウエストナイルウイルス
αGalCer	α-Galactosylceramide	アルファガラクトシルセラミド
再生医療等法		再生医療等の安全性の確保等に関する法律

0. 研究の概要

研究課題名	再発・進行頭頸部がん患者を対象とした iPS-NKT 細胞動注療法及び自家 DC/Gal 併用療法の忍容性、安全性及び有効性に関する第 I 相試験
試験の目的	根本治療が困難な標準治療後の再発・進行頭頸部がん患者に対する、iPS-NKT 細胞の腫瘍栄養動脈内投与及び自家 DC/Gal 併用療法の忍容性の検討並びに安全性及び有効性を探索的に評価すること。
試験デザイン	単施設、非盲検、非対照試験
フェーズ	第 I 相試験
特定細胞加工物	ヒト iPS 細胞由来 NKT 細胞（iPS-NKT 細胞）<br>自家 DC/Gal
選択基準	以下のすべての条件に該当する患者を対象とする。<br>1) 標準的治療に不応又は不耐の再発又は進行頭頸部がん患者のうち、腫瘍栄養動注投与可能かつ評価可能な病変を有する患者。<br>2) 前治療から試験治療開始までに 1 カ月以上が経過する見込みのある患者。前治療の種類は問わない。<br>3) 同意取得時の年齢が 20 歳以上 80 歳未満の患者。<br>4) ECOG の Performance Status が 2 以下の患者。<br>5) 以下の検査データを満たす患者。<br>・ヘモグロビン≧10 g/dL<br>・白血球数≧3,000/μL、血小板数≧75,000/μL<br>・血清クレアチニン≦1.5 mg/dL<br>・総ビリルビン≦1.5 mg/dL、AST （GOT）、ALT （GPT）≦施設基準値上限の 2.5 倍<br>・SpO_2（room air） ≧93 %<br>6) 3 カ月以上の予後が期待される患者。<br>7) 本人からの文書による同意が得られている患者。

除外基準	以下のいずれかの条件に該当する患者は対象としない。<br>1) HBs 抗原、HCV 抗体、HIV 抗体又は HTLV-1 抗体が陽性若しくは HBs 抗原陰性であるが HBV-DNA 定量検査で HBV-DNA が検出された患者。<br>2) 試験治療開始前の少なくとも 2 週間以内にコルチコステロイド（メチルプレドニゾロン 10 mg/日以上又は相当量）又は免疫抑制剤を内服又は注射している患者。<br>3) 妊娠、授乳中又は本試験中に妊娠を予定している女性患者。また妊娠可能な女性及び男性で、試験期間中及び iPS-NKT 細胞最終投与後 14 日間まで医師の指導のもと異性間の性交を禁止することに同意しない者（ただし本試験の同意を得る前に、本人又はパートナーの両側卵管閉塞術又は精管切除術を既に実施していた場合、異性間の性交禁止は該当しない）。<br>4) コルチコステロイドや生物学的製剤の全身投与もしくは免疫抑制療法を要する活動性の自己免疫疾患を有する患者。<br>5) 免疫チェックポイント阻害薬で免疫関連有害事象を生じた患者。<br>6) コントロール不良な糖尿病を有する患者。<br>7) 重症以上の肺疾患を有する患者（mMRC 息切れスケール Grade 2 以上）、又はステロイドによる治療を要する非感染性の間質性肺疾患の既往を有する患者。<br>8) 重大な心疾患を有する患者（NYHA class III 以上）。<br>9) 試験治療の初回投与前 2 年以内に進行中または積極的な治療を必要とする別の悪性腫瘍があることが分かっている。ただし、根治治療が実施された皮膚の基底細胞がん、皮膚の扁平上皮癌、根治的切除された非浸潤性乳管がん及び根治的切除された非浸潤性乳がん及び内視鏡的に根治切除された上部消化管癌を除く。<br>10) 造影剤を使用できない患者。<br>11) ヒト血清アルブミン製剤、又は異種由来タンパク質に対し過敏症の既往のある患者。<br>12) 同意取得時、他の試験又は臨床試験に参加し、他の試験製品等の投与を受けている又は当該試験製品等による有害事象の影響が残存していることが、実施責任者又は分担医師により判断される患者。<br>13) HLA-A、B 及び C の genotype が iPS-NKT 細胞と完全に一致する患者。<br>14) 成分採血禁忌（不安定狭心症、A-V block II 度以上、WPW 症候群、完全左脚ブロック、収縮期血圧 90 以下もしくは 170torr 以上）の患者。<br>15) 担当医が本試験への参加を不適当と判断した患者。

評価項目	主要評価項目<br>用量制限毒性（DLT）発現割合<br>副次評価項目<br>【有効性の副次評価項目】<br>・ 奏効割合（RECIST ver.1.1）<br>・ 病勢コントロール割合（RECIST ver.1.1）<br>【安全性の副次評価項目】<br>・ 有害事象の発現状況（種類、頻度及び重症度等）<br>・ 臨床検査値の推移<br>探索的評価項目<br>・ 末梢血中 iPS-NKT 細胞濃度推移<br>・ 免疫細胞分画（T 細胞分画、NKT 細胞マーカー等）<br>・ 末梢血免疫細胞における Omics 解析
投与量・投与方法	1) 本試験は iPS-NKT 細胞及び自家 DC/Gal 併用療法の First in human 試験であり、単用量における 3+3 デザインとする。すなわち、設定された用量を投与された最初の 3 例において DLT の発現が認められない場合は、当該用量を MTD とし、1 例 DLT 発現が認められた場合は同用量を追加の 3 例に投与し、DLT 発現例数が 6 例中 2 例以上の場合は試験を中止する。最終的に、合計 6 例において DLT 発現例数が 1 例以下であれば当該用量を MTD とする。<br>2) 自家 DC/Gal 投与 5 日後に iPS-NKT 細胞を投与する。<br>3) 投与細胞数は、自家 DC/Gal 1×10^{8} cells/回、iPS-NKT 3×10^{7} cells/m^{2}/回とする。<br>4) 投与回数は、自家 DC/Gal 及び iPS-NKT をそれぞれ 1 回とする。<br>5) 2 例以上の被験者に対する本併用療法の同日投与は行わず、少なくとも 7 日以上の間隔をあけて次の被験者における本試験治療を開始する。<br>6) 自家 DC/Gal は微量注射シリンジを用いた鼻粘膜下投与、iPS-NKT 細胞は腫瘍栄養動脈への投与とし、血管造影下で挿入されたカテーテル（セルジンガー法）を介して行う。

DLTの定義	自家DC/Gal投与からiPS-NKT細胞投与後14日目までの期間（DLT評価期間）に観察された、DC/Gal又はiPS-NKT細胞との因果関係が否定できない以下の有害事象をDLTとする。Grade判定はCTCAE ver.5.0 日本語訳JCOG版に従う。<br>・ Grade 4以上の血液毒性<br>・ 原疾患と関連のないあらゆる輸血及びG-CSF製剤を要する血液毒性<br>・ Grade 3以上の非血液毒性（一過性の臨床検査値異常、適切な処置によりGrade 2以下へ回復した下痢、悪心、嘔吐又はその他の管理可能な全身症状は除く）<br>その他、DC/Gal又はiPS-NKT細胞との因果関係が否定できない有害事象により、以下の状況となった場合、当該有害事象をDLTとする。<br>・ 輸血療法を行った場合<br>実施責任者はDLTの定義に該当すると疑われる疾患、徴候が発生した際には、速やかにデータモニタリング委員会に判断の妥当性の審議を依頼し、当該患者における試験継続の可否を決定する。
投与開始基準	本試験に登録された患者に対して、DC/Gal又はiPS-NKT細胞投与日に、「投与中止基準」のいずれにも該当しないことを確認した上で、DC/Gal又はiPS-NKT細胞の投与を開始する。
投与延期の基準	以下の基準に該当した場合、実施責任者又は分担医師はiPS-NKT細胞を最長3日まで延期できる。<br>1) 実施責任者又は分担医師がDC/Gal又はiPS-NKT細胞の投与を不適当と認めた場合。<br>2) DC/Gal又はiPS-NKT細胞の投与後に発現したすべての有害事象が管理可能なGrade 2若しくはGrade 1以下又はベースラインまで回復しない場合。<br>3) iPS-NKT細胞投与前24時間以内に、38℃以上の発熱が認められた場合を含む治療を要する感染症が発現した場合。

投与中止基準	以下のいずれかの基準に該当した場合、実施責任者又は分担医師は DC/Gal 又は iPS-NKT 細胞の投与を中止する。投与中止となった場合は、投与中止が決定された日から観察期に移行し、検査スケジュールに沿った検査・評価を継続して実施する。<br>1) DC/Gal 投与後に DLT が出現した場合、又は試験期間中に発生した Grade 3 以上のすべての有害事象が、DC/Gal 又は iPS-NKT 細胞投与直前までに回復若しくは Grade 1 に改善しない、又は臨床的に十分管理可能な Grade 2 に改善しない場合。<br>2) 治療を要する感染症が発現し、DC/Gal 又は iPS-NKT 細胞投与直前までに回復若しくは Grade 1 に改善しない、又は臨床的に十分管理可能な Grade 2 に改善しない場合。<br>3) SpO_2（room air）が 90 %未満で維持されている。<br>4) DC/Gal 又は iPS-NKT 細胞投与前の検査において mMRC 息切れスケール Grade 2 以上。<br>5) 被験者からの中止の申し出があった場合。<br>6) その他、実施責任者又は分担医師により被検者への DC/Gal 又は iPS-NKT 細胞投与中止の必要性が認められた場合。
個々の症例における試験中止基準	1) 実施責任者又は分担医師の判断により、併用禁止薬又は併用療法を必要とする原疾患の進行が認められた場合。<br>2) 被験者からの中止の申し出があった場合。<br>3) 症例登録後、被験者が対象として不適格であることが判明した場合。<br>4) その他、実施責任者又は分担医師が被験者の DC/Gal の投与が不可能と判断した場合。
併用禁止薬・療法	・ コルチコステロイド、免疫抑制剤、他の抗悪性腫瘍薬、放射線治療又は腫瘍切除を含む手術。<br>・ あらゆる輸血療法（ただし、DC/Gal 又は iPS-NKT 細胞に起因すると考えられる有害事象への対応として上記製剤又は療法が必要となった場合、当該有害事象を DLT とした上で、上記製剤又は療法を使用することは可能とする）。
各被験者の試験参加期間	各被験者の試験期間は、同意取得日から最終観察終了日とする。被験者が中止を希望した場合、及び転院等により観察・調査が困難になった場合には、その当該日の当日を中止日及び最終観察終了日とする。
目標被験者数	2 名～6 名
試験実施期間	試験実施期間：試験計画届終了後～2024 年 8月 31 日
試験施設数	1 施設、千葉大学医学部附属病院
実施責任者	飯沼　智久<br>千葉大学大学院医学研究院　耳鼻咽喉科・頭頸部腫瘍学　助教

試験調整医師	千葉大学大学院医学研究院　免疫細胞医学　教授　本橋　新一郎（代表）<br>千葉大学大学院医学研究院　耳鼻咽喉科・頭頸部腫瘍学　教授　花澤　豊行<br>千葉大学医学部附属病院　臨床試験部　教授　花岡　英紀
DC/Gal 又は iPS-NKT 細胞提供者	千葉大学医学部附属病院　未来開拓センター<br>国立研究開発法人　理化学研究所生命医科学研究センター
倫理指針及び再生医療等法	本試験の実施に際しては「ヘルシンキ宣言」に基づく倫理的原則、「再生医療等の安全性の確保等に関する法律」及びその他の関連する規制要件を遵守するものとする。
審査する委員会	本試験の実施に先立ち、実施医療機関の特定認定再生医療等委員会及び生命倫理審査委員会は、本試験の倫理的、科学的及び医学的妥当性を審査する。本試験は、特定認定再生医療等委員会及び生命倫理審査委員会の承認を得た後に実施する。特定認定再生医療等委員会及び生命倫理審査委員会の審議結果が「修正の上で承認する」であった場合には、審議結果に基づいて実施計画書又は症例報告書、同意説明文書等を修正し承認された後、本試験を実施する。また、特定認定再生医療等委員会及び生命倫理審査委員会は少なくとも 1 年に 1 回以上の頻度で本試験が適切に実施されているか否かを継続的に審査する。

1. 緒言

1.1. 試験の背景

1.1.1. 頭頸部がんと治療の現状について

国立がん研究センターがん対策情報センターの地域がん登録での推計値によると、頭頸部がんの発生頻度は人口 10 万人に対して口腔・咽頭癌は 8.6 人、喉頭癌は 2.8 人であり、頭頸部がん全体では我が国の全癌の 5%程度を占めると考えられている。頭頸部がんの多くは扁平上皮癌であり、非進行癌では経口的手術や強度変調放射線治療により高い治療成績を維持しながら生活の質を下げない治療が可能となりつつある。一方、進行癌に対しては、同時併用化学放射線療法と必要に応じて救済手術を行う、又は根治切除手術と術後化学放射線治療が第一選択となるが、侵襲性の高いこれらの治療によっても、多くの進行頭頸部がんの 5 年生存率は依然として 40～50%程度である。また、これらの治療により完全奏効が得られた患者でも、局所再発や遠隔転移再発に対する有効な予防法は存在しない。根治治療後の再発患者のうち、救済手術の適応がない場合や遠隔転移を有している場合には全身化学療法が選択される。KEYNOTE-048 試験において、ペムブロリズマブ単剤またはペムブロリズマブ、白金製剤および 5-FU の 3 剤併用療法は、従来の抗悪性腫瘍剤と比較して有意に全生存期間（OS）を延長させることが報告され[1]、上記患者に対する標準的治療とされている。しかしながら、全生存期間の延長効果（中央値）は 2.0-4.7 カ月程度であることに加え、すべての頭頸部がん患者で適応となる訳ではないことが課題となっている。また、白金系抗悪性腫瘍剤に対して不応である頭頸部がん患者を対象とした CHECKMATE-141 試験において、免疫チェックポイント阻害薬であるニボルマブは、他の抗悪性腫瘍剤と比較して全生存期間（中央値）で 2.4 カ月の延長が認められたこと等から[2]、本邦を含む世界各国で、再発又は遠隔転移を有する頭頸部がんを効能・効果として承認されている。しかし、CHECKMATE-141 試験におけるニボルマブの奏効率は 13%と限定的であり、頭頸部がん患者に対する新規免疫療法の開発が求められている。

1.1.2. NKT 細胞免疫療法について

NKT 細胞は細胞表面上に T 細胞抗原受容体（TCR）と NK 受容体（NKR）をともに発現するユニークなリンパ球であり[3]、リガンドとして、αGalCer が明らかにされている[4]。αGalCer により活性化した NKT 細胞は、癌細胞を MHC 発現に関わらず認識し、パーフォリンやグランザイムを放出することにより、癌細胞を直接傷害すると同時に、IFN-γ産生を介して NK 細胞及び CTL を活性化することにより、癌細胞を間接的にも傷害する[5, 6, 7]。

非小細胞肺癌を対象に、活性化 NKT 細胞や αGalCer をパルスした樹状細胞（DC/Gal : DC/Gal）を静脈内投与した際の安全性及び有効性を検討した臨床試験において、重篤な有害事象は認められず、NKT 細胞に由来する抗腫瘍免疫反応の誘導が確認された[8, 9]。

また、頭頸部がん患者を対象とした臨床研究において、担癌患者で認められる末梢血 NKT 細胞数の低下が頭頸部がん患者では認められなかったこと、及び NKT 細胞は通常の T 細胞と異なり、頭頸部がんの標準的治療として広く用いられている放射線治療に対して耐性を有していることが明らかとなり[10]、NKT 細胞免疫療法の頭頸部がんへの有用性が期待された。これまでに千葉大学病院で実施された臨床研究にお

いて、DC/Gal は鼻粘膜下に投与すると、頸部リンパ節に移行して活性化 NKT 細胞の誘導に作用することが明らかとなった[11, 12]。そこで根治治療適応外の再発・進行頭頸部がん患者を対象とした、DC/Gal の鼻粘膜下投与に関する第 I 相試験が実施された。重篤な有害事象は確認されず、抗腫瘍免疫活性の上昇及び腫瘍縮小効果を示す症例が認められた[13]。現在、根治治療後頭頸部扁平上皮がん患者を対象に、NKT 細胞免疫療法の再発抑制効果を評価することを目的とした DC/Gal 鼻粘膜下投与の第 II 相試験を二重盲検無作為化比較試験として解析中である。

NKT 細胞免疫療法として、上記の鼻粘膜下投与に加え、腫瘍栄養動脈内投与の検討も進めている。頭頸部がんの多くは外頸動脈の終末血行支配を受けており、当該腫瘍栄養動脈内への抗悪性腫瘍剤投与（以下、「超選択的動注」）が可能である。抗悪性腫瘍薬の超選択的動注は静脈内投与と比較して、局所に対する高い有効性と全身の副作用の軽減が期待できる。当該技術を応用し、標準的治療後の局所再発症例を対象とした、鼻粘膜下への DC/Gal 投与と活性化 NKT 細胞の超選択的動注との併用に関する 2 つの臨床試験が実施された。2005 年 8 月から切除不能な頭頸部がん再発症例に対して、DC/Gal の粘膜下投与と活性化した NKT 細胞を動脈に投与する治療法の安全性と有効性を確認するための試験（第 I/II 相臨床研究）を 8 例に行い、3 例で腫瘍の縮小が認められ、4 例で腫瘍増大が抑制された。2007 年 12 月からは、頭頸部がん局所再発症例の救済手術前に、DC/Gal の粘膜下投与と活性化した NKT 細胞を動脈に投与する治療法の有効性と安全性を確認するための試験（第 II 相臨床研究）を 10 例に行い、5 例で腫瘍の縮小が認められ、5 例で腫瘍増大が抑制された。

1.1.3. NKT 細胞免疫療法における課題と iPS 細胞の応用

NKT 細胞は元々白血球中に 0.01～0.1%程度と少ない上に、患者によるばらつきも多いことから治療効果に限界があることが示唆された。そこで、iPS 細胞を用いて *in vitro* で分化、成熟及び増殖された大量の NKT 細胞を、頭頸部がん患者に対して超選択的動注で投与することにより、さらに高い臨床的有用性が示されることが期待される。

理化学研究所と共同で研究開発している iPS-NKT 細胞を用いた非臨床試験において、*in vitro* での 6 種類の癌細胞株（K562；ヒト白血病、NCI-H460；ヒト大細胞癌、A549；ヒト肺細胞基底上皮腺癌、HT-29；ヒト結腸癌、COLO205；ヒト大腸癌、Detroit562；ヒト咽頭癌）に対する殺細胞効果に加え、ヒト頭頸部がん移植マウスに対する腫瘍増殖抑制作用が示され、安全性上懸念すべき所見は認められなかった。当該結果等を踏まえ、現在根治治療困難な再発・進行頭頸部がん患者を対象とした、iPS-NKT 細胞の超選択的動注の忍容性及び安全性等を評価することを目的とした第 I 相試験を医師主導治験として実施中である（jRCT2033200116）。

1.1.4. iPS 細胞と自家 DC/Gal との併用

上記 1.1.2 で記載したとおり NKT 細胞の活性化には DC/Gal による刺激が重要であることから、iPS-NKT 細胞においても同様の活性化機序により iPS-NKT 細胞単独投与と比較して iPS-NKT 細胞と自家 DC/Gal の併用療法を行うことでより高い有効性が期待される。マウスにおける前臨床試験において iPS-NKT 細胞単独では抗腫瘍効果のみられなかった患者由来腫瘍に対しても DC/Gal 併用療法において抗腫

瘍効果が認められた。またマウスを用いた異種モデルではDC/Gal併用療法伴う重篤な有害事象が認められなかった。

以上の結果を踏まえ、本臨床試験では医師主導治験と同様に根治治療困難な再発・進行頭頸部がん患者を対象として iPS-NKT 細胞と自家 DC/Gal の併用療法の忍容性及び安全性等を評価することを目的とした第 I 相試験を計画するに至った。

1.2. iPS-NKT 細胞及び DC/Gal について

1.2.1. iPS-NKT 細胞及び DC/Gal の概要

1.2.1.1. iPS-NKT の概要

1) 特定細胞加工物名（コード名）: iPS-NKT
2) 特定細胞加工物の成分名、又は予定される一般的名称：ヒト iPS 細胞由来 NKT 細胞
3) 特定細胞加工物の原材料：健康な成人ドナー末梢血由来の NKT 細胞
4) 特定細胞加工物の剤型、規格、性状及び組成：
   剤型：注射剤
   規格：表 6 参照
   性状：淡紅色懸濁液
   組成：αMEM（20％ヒト AB 型血清、5 ng/mL IL-7、10 ng/mL IL-15 含有）
5) 原材料の採取場所：千葉大学医学部附属病院
   〒260-8677　千葉県千葉市中央区亥鼻 1-8-1
6) 特定細胞加工物の細胞培養加工施設：理化学研究所生命医科学研究センター
   〒230-0045　神奈川県横浜市鶴見区末広町 1-7-22
7) 細胞の特性 : 本特定細胞加工物は、NKT 特異的 TCR（Vα24, Vβ11）を発現することを特徴とする CD3 陽性のリンパ球様細胞である。
8) 細胞の純度：CD3 陽性細胞であり NKT 特異的 TCR（Vα24, Vβ11）を発現する細胞集団（CD3、TCR Vα24、TCR Vβ11　陽性細胞）
9) 体内での予想される働き：腫瘍栄養血管に選択的に動脈内投与を行うと、iPS-NKT 細胞は腫瘍組織でがん細胞を直接傷害することが期待される。また、DC を介した活性化によるアジュバント作用により、NK 細胞及び CTL を活性化して抗腫瘍効果を発揮することも期待される。一部の細胞は全身を循環する可能性があるが、他家細胞は拒絶されることから、早期に排除されると考えられる。
10) 寿命：人体内での寿命は不明であるが、他家細胞であるため生着はしないと考えられる。
11) 他の細胞への影響：本特定細胞加工物は、ヒト iPS 細胞由来の NKT 細胞であり、自己複製能はあるが、増殖限界が存在する。腫瘍栄養血管への選択的な動脈内投与であること、及び他家細胞であることから投与後腫瘍組織での抗腫瘍効果発現後は宿主の免疫系によって排除されると考えられる。GVHD により宿主細胞が非特異的に傷害される可能性に関しては免疫系ヒト化マウスを用いた試験では観察されなかったため、可能性は低いと考えられる。

12) 細胞の安全性：1.2.4.項参照。

1.2.1.2. 自家 DC/Gal の概要

1) 特定細胞加工物名（コード名）： DC/Gal
2) 特定細胞加工物の成分名、又は予定される一般的名称：αGalCer パルス樹状細胞
3) 特定細胞加工物の原材料：自家末梢血単核球
4) 特定細胞加工物の剤型、規格、性状及び組成：
   剤型：注射剤
   規格：表 11 参照
   性状：淡黄色懸濁液
   組成：生理食塩水（2.5％ヒトアルブミン含有）
5) 原材料の採取場所：千葉大学医学部附属病院
   〒260-8677　千葉県千葉市中央区亥鼻 1-8-1
6) 特定細胞加工物の細胞培養加工施設：千葉大学医学部附属病院　未来開拓センター
   〒260-8677　千葉県千葉市中央区亥鼻 1-8-1
7) 細胞の特性：本特定細胞加工物は、CD86 を発現することを特徴とする CD45 陽性の単核球細胞群である。
8) 細胞の純度：CD45 陽性細胞であり T 細胞抗原受容体の共刺激分子 CD86 を 15%以上発現する細胞集団（CD45, CD86 陽性細胞）
9) 体内での予想される働き：Kurosaki, M.（Cancer Immunol Immunother, 2011）らによれば，鼻粘膜に投与されると、DC/Gal の一部は頸部所属リンパ節に遊走し、NKT 細胞及び iPS-NKT 細胞を活性化することで抗腫瘍効果を発揮することが期待される。また一部の細胞は全身を循環し、iPS-NKT 細胞を活性化することで、抗腫瘍効果を発揮することが期待される。
10) 寿命：人体内での正確な寿命は不明であるが、Kurosaki, M.（Cancer Immunol Immunother, 2011）らによれば，鼻粘膜下に投与後 1 週間後でも検出可能である。
11) 他の細胞への影響：DC/Gal は、自家末梢血単核球由来の抗原提示細胞であり、細胞上に提示された αGalCer を NKT 細胞が認識することで活性化し、抗腫瘍効果が発揮されると考えられる。DC/Gal が GVHD を引き起こし、宿主細胞が非特異的に傷害される可能性に関しては、DC/Gal が自家細胞であることや免疫系ヒト化マウスを用いた iPS-NKT 細胞との併用投与試験では観察されなかったため、可能性は低いと考えられる。
12) 細胞の安全性：1.2.4.項参照。

1.2.2. iPS-NKT 細胞及び DC/Gal の製造

1.2.2.1. iPS-NKT 細胞の製造

製造の各工程（重要中間体製造、誘導培養と凍結ストック作製、純化培養）の概略、各段階における規

格試験（NKT-iPS 細胞規格試験、iPS-NKT 工程内管理試験、iPS-NKT 規格試験）の実施時期及び製造時の管理項目を図 1 に示す。詳細な製造工程フロー図は 図 2 図 2 に示す[参考資料 i.]。製造工程におけるバリデーションポイントの設定に関しては参考資料 ii.に添付した。

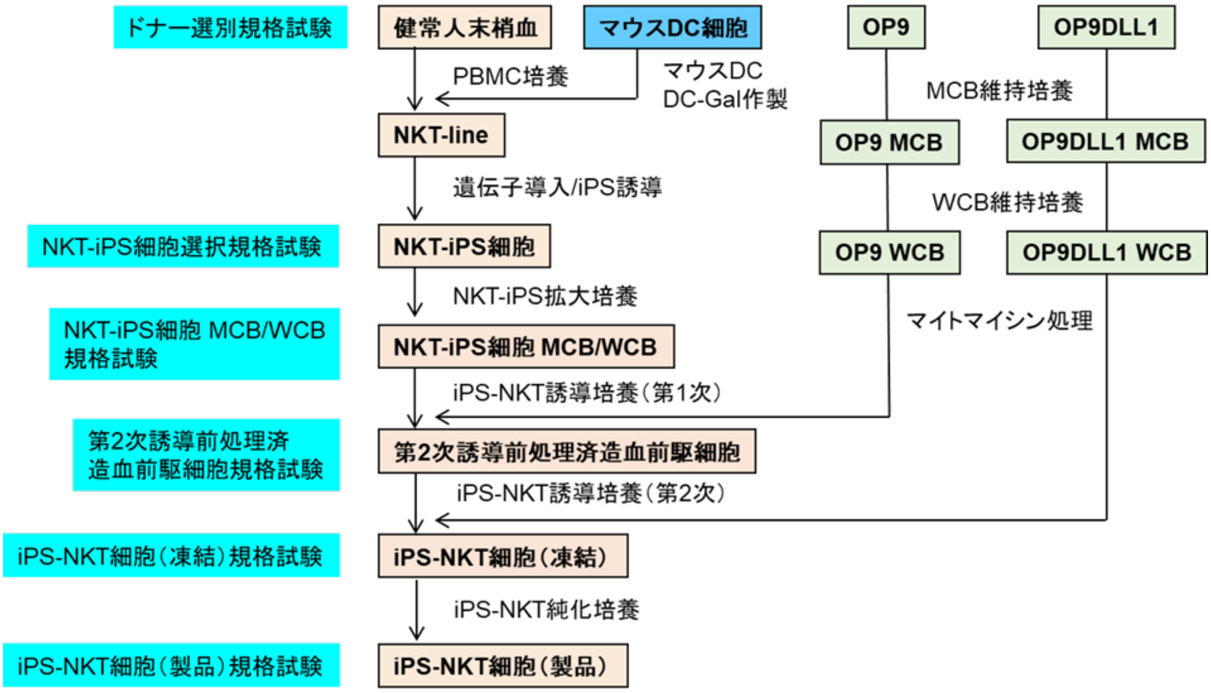

図 1　**iPS-NKT** 細胞　製造方法の概略・要素

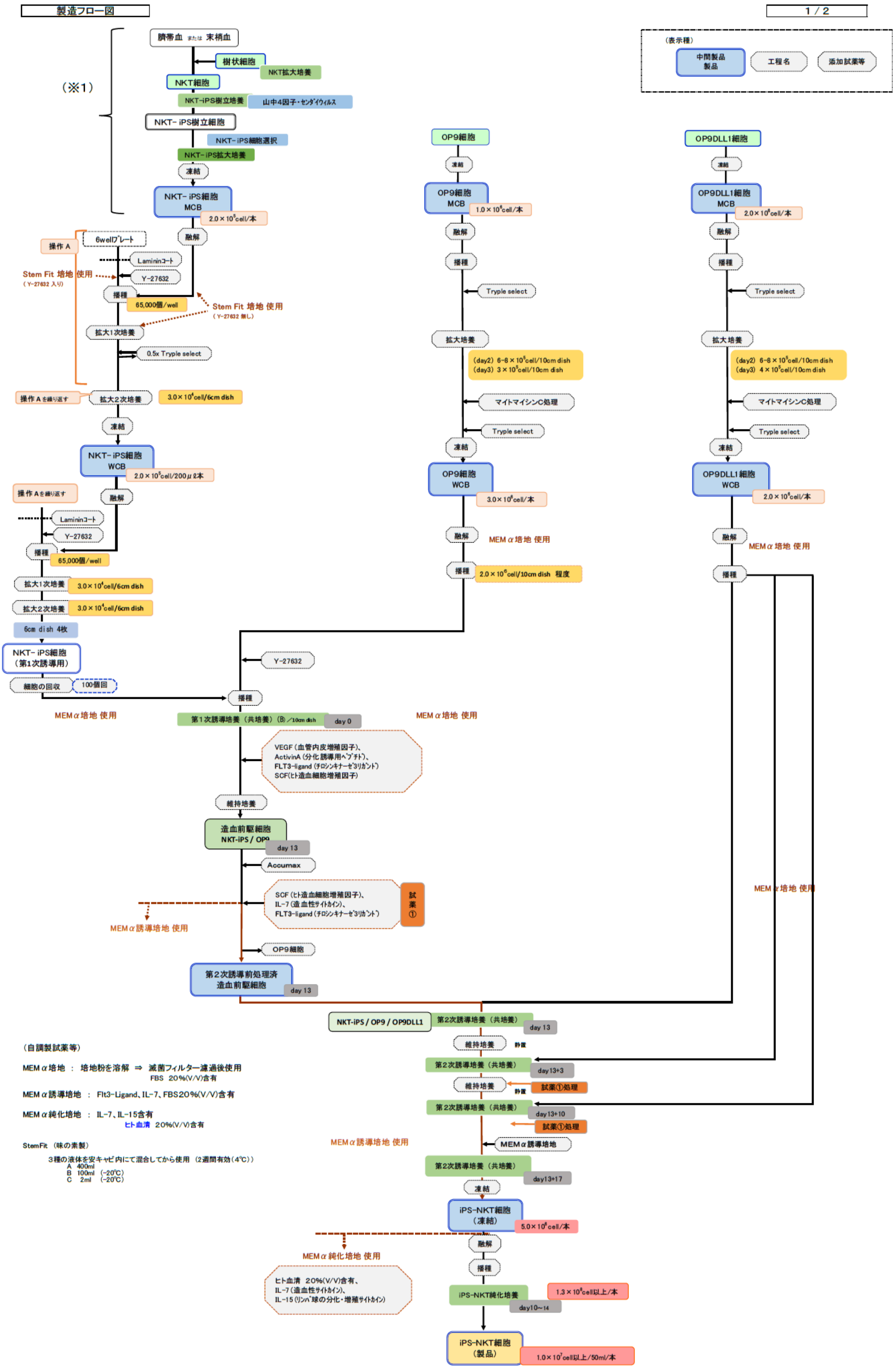

図 2 iPS-NKT 細胞 製造方法の概略・要素（詳細版）

1.2.2.2. iPS-NKT 細胞におけるドナー選別規格試験（表 1）

試験検体は、文書同意した健常人の末梢血、これから分離した末梢血単核球(PBMC)および NKT 細胞を使用する。ドナーとしての適格性はヒト細胞組織原料基準に準拠し、医師による問診、検診、血液検査、血液性化学検査、無菌検査、マイコプラズマ否定試験に加え、表 6 に示した特定の細菌およびウイルスに関する検査を実施することにより確認する。またこれら特定の細菌およびウイルスに関しては、ウインドウピリオドである 3~4 カ月後に再検査を実施し、陰性であることを再確認する。

表 1　ドナー選別規格試験の一覧表

試験項目	試験詳細	試験方法	暫定評価基準値
健康状態	医師による検診	問診、検診、検査	既往歴、血液検査、血液性化学検査、などから総合的に判断（健康）
	細菌・ウイルス否定	PCR 法、<br>血清学的方法<br>培養法	無菌、マイコプラズマ否定、梅毒トレポネーマ, HIV, HTLV, HCV, HBV, ParvoB19, EBV, CMV, WNV 陰性（ウインドウピリオドである 3-4 カ月後に再検査）
NKT 細胞特性	NKT 細胞増幅試験	培養	リガンド(DC/G)刺激により 1×10^6 個以上に増幅
遺伝子解析	HLA 解析	NSG 法	HLA-A, -B, -C, -DRB1 の決定

1.2.2.3. NKT-iPS 細胞選択規格試験（表 2）

試験検体は、培養後の NKT-iPS 細胞懸濁液を使用するが、細胞機能に関しては純化培養後の iPS-NKT 細胞（細胞数計測）および誘導培養開始から 33 日目(d33)および 43 日目(d43)の純化培養中の iPS-NKT 細胞を使用して測定する。

表 2　NKT-iPS 細胞選択規格試験及び特性解析試験の一覧表

試験項目	試験詳細	試験方法	暫定評価基準値
一般特性	細胞形態	顕微鏡観察	多能性幹細胞と同等のコロニー形態
	生細胞数	細胞数計測	凍結チューブ 1 本当たり 2×10^5 個以上
	NKT 特異的 TCR 再構成	PCR 法	NKT 特異的 TCR Vα24 遺伝子の再構成が確認される
細胞機能	NKT 細胞への分化能	細胞数計測	規格試験済みの OP9、OP9-DLL1 を用いて、100 クランプ／10 cm dish 1 枚あたり 1×10^6 個以上の NKT 細胞を誘導.且つその細胞が、純化培養条件下で増殖すること
		FACS 解析	(d33)回収した生細胞に、CD45+CD3+細胞が含まれていること。<br>(d43)純化培養条件下で増殖し回収した CD45+細胞のうち、CD3 発現細胞が 80%以上、かつ、CD3+細胞が NKT 特異的 TCR(Va24、Vb11)を発現していること
安全性	無菌試験	培養法	真菌、一般細菌陰性
	マイコプラズマ	PCR 法	陰性
	エンドトキシン	比色法・比濁法	基準値(0.3 EU/mL) 未満
	センダイウイルス否定	PCR 法	陰性
特性解析試験			
多能性マーカー発現	遺伝子発現	多能性マーカー遺伝子群発現	Actb 617-618、Oct 367-368、Sox2 369-370、Klf4 359-360、c-Myc 566-567、Nanog 363-364　陽性
	細胞表面マーカー発現	免疫染色	SSEA3, SSEA4, TRA1-60, TRA1-81 陽性

1.2.2.4. NKT-iPS 細胞 MCB/WCB 規格試験（表 3 表 4）

試験検体は、培養後の NKT-iPS 細胞マスターセルバンク（MCB）及びワーキングセルバンク（WCB）懸濁液を使用する。ウイルス試験および特性解析試験は、MCB についてのみ行う。細胞機能に関しては純化培養後の iPS-NKT 細胞（細胞数計測）および誘導培養開始から 33 日目（d33）および 43 日目（d43）の純化培養中の iPS-NKT 細胞を使用して測定する。

表 3　NKT-iPS 細胞 MCB 規格試験の一覧表

規格試験			
試験項目	試験詳細	試験方法	暫定評価基準値
一般特性	細胞形態	顕微鏡観察	多能性幹細胞と同等のコロニー形態、
	総細胞数	細胞数計測	凍結チューブ 1 本当たり 2×10^5 個以上
	生細胞数（又は生存率）	細胞数計測・生存率計測	融解時に凍結チューブ 1 本当たり 1.6×10^5 以上
細胞機能	NKT 細胞への分化能	細胞数計測	規格試験済みの OP9、OP9DLL を用いて、100 クランプあたり 10 cm dish あたり 1×10^6 個以上の NKT 細胞を誘導.且つその細胞が、純化培養条件下で増殖すること
		FACS 解析	(d33)回収した生細胞に、CD45+CD3+細胞が含まれていること。<br>(d43)純化培養条件下で増殖し回収した CD45+細胞のうち、CD3 発現細胞が 80%以上、かつ、CD3+細胞が NKT 特異的 TCR(Va24、Vb11)を発現していること
安全性	無菌	培養法	真菌、一般細菌陰性
	マイコプラズマ否定	PCR 法	陰性
	エンドトキシン	比色法・比濁法	基準値(0.3 EU/mL) 未満
	ウイルス否定<br>(MCB のみ)	感染性試験	陰性
		電子顕微鏡試験	陰性
		逆転写酵素活性試験	陰性
		ヒトウイルス試験	陰性
		in vitro 試験	陰性
		in vivo 試験	陰性
		ウシ・ブタウイルス試験	陰性
		マウス感染性試験	陰性
		マウス抗体産生試験	陰性
		マウスウイルス試験	陰性
特性解析試験			
多能性マーカー発現	遺伝子発現	PCR 法	Actb 617-618、Oct 367-368、Sox2 369-370、Klf4 359-360、c-Myc 566-567、Nanog 363-364　陽性
	細胞表面マーカー発現	FACS 解析	SSEA4 TRA-1-81 60
遺伝子解析	染色体核型安定性	染色体核安定性試験	正常核型が 9 割以上であり、１ヶ月以上(４パッセージ以上)後も安定している
	エクソーム<br>（がん関連遺伝子）"	約 600 のがん関連遺伝子領域のディープシーケンス（x1000）	既知のがん関連遺伝子領域において遺伝子変異が認められない

表 4　NKT-iPS 細胞 WCB 規格試験の一覧表

規格試験			
試験項目	試験詳細	試験方法	暫定評価基準値
一般特性	細胞形態	顕微鏡観察	多能性幹細胞と同等のコロニー形態、
	総細胞数	細胞数計測	凍結チューブ 1 本当たり 2×10^5 個以上
	生細胞数（又は生存率）	細胞数計測・生存率計測	融解時に凍結チューブ 1 本当たり 1.6×10^5 以上
細胞機能	NKT 細胞への分化能	細胞数計測	規格試験済みの OP9、OP9DLL を用いて、100 クランプあたり 10 cm dish あたり 1×10^6 個以上の NKT 細胞を誘導.且つその細胞が、純化培養条件下で増殖すること
		FACS 解析	(d33)回収した生細胞に、CD45+CD3+細胞が含まれていること。(d43)純化培養条件下で増殖し回収した CD45+細胞のうち、CD3 発現細胞が 80%以上、かつ、CD3+細胞が NKT 特異的 TCR(Va24、Vb11)を発現していること
安全性	無菌	培養法	真菌、一般細菌陰性
	マイコプラズマ否定	PCR 法	陰性
	エンドトキシン	比色法・比濁法	基準値(0.3 EU/mL) 未満

1.2.2.5. iPS-NKT 細胞（凍結）規格試験（表 5）

試験検体は、培養後の iPS-NKT 細胞（凍結）懸濁液を使用する。規格値の内、細胞機能（in vitro IFNγ 産生能及び in vitro 抗腫瘍効果）に関しては、その評価を本製品製造と比較してミニスケールで実施しており、臨床研究用製品製造において、上記暫定規格値が適用可能かは未だ明らかではない。また、臨床研究開始までに十分な量のロットを試験することが出来なかったため、2021 年 12 月 17 日時点では目標値または参考値として扱い、臨床研究用ロットの製造を通じて更に検討することにより、暫定規格値として適用可能か検証を行う。

表 5　iPS-NKT 細胞（凍結）規格試験及び特性解析試験の一覧表

規格試験			
試験項目	試験詳細	試験方法	暫定規格値、備考
一般特性 (Day 33)	生細胞数	細胞数計測	2~5×10^6 個/凍結チューブ 1 本
	細胞表面マーカー発現	FACS 解析	回収した生細胞に、CD45+CD3+細胞が含まれていること
安全性	無菌	培養法	真菌、一般細菌陰性
	マイコプラズマ否定	PCR 法	陰性
	エンドトキシン	比色法・比濁法	基準値(0.3 EU/mL) 未満
	細胞純度	PCR 法	未分化マーカー(LIN28)発現細胞陰性
細胞機能	in vitro IFNγ 産生能	ELISA	IFNγ の確認（目標値：陰性サンプルの平均値の 1.7 倍）
	in vitro 抗腫瘍効果	K562 細胞殺傷能	抗腫瘍効果の確認（目標値：7%）
特性解析試験			
細胞機能	増殖能確認	細胞数計測	純化培養工程で細胞増殖が確認されること
	細胞表面マーカー発現	FACS 解析	CD45+細胞のうち、CD3+細胞が NKT 特異的 TCR(Vα24、Vβ11)を発現していること

1.2.2.6. iPS-NKT 細胞（製品）規格試験（表 6）

試験検体は、純化培養後の iPS-NKT 細胞（製品）を使用する。無菌試験は、出荷 2～3 日前に製造中の製品を抜き取り迅速法および日局法（嫌気性菌）を用いて実施し、出荷判定試験とするとともに、出荷時に日局法による無菌試験も実施することとした。表 7 に示す迅速法では、好気性菌および真菌を 48 時間の培養で検出することが可能であるため、出荷 2～3 日前のサンプルを用いて評価することにより

患者への投与前に無菌性に関する情報を得ることが可能である。一方、嫌気性菌に関しては日局法でも検査が可能であるため、日局法で試験を行う。したがって、出荷前に迅速法および日局法による好気性菌、真菌および嫌気性菌の出荷判定を行うと共に、投与後に迅速法および日局法によって菌が検出された場合の対応を試験実施施設と取り決める。細胞機能に関しては、アジュバント効果に関係するインターフェロンγ（IFN-γ）産生能と直接的な in vitro 抗腫瘍効果のばらつきが多く、2021 年 12 月 17 日時点では測定精度が不十分であるため、iPS-NKT 細胞（凍結）と同様に基準値は設けず、目標値として設定し、製品製造完了後に再度検証する。

また、分化誘導時にフィーダー細胞としてマウス細胞、培地添加物として FBS を使用する事から、洗浄後の投与製剤において異種タンパクの測定をすることとする。

表 6　iPS-NKT 細胞（製品）規格試験の一覧表

規格試験			
試験項目	試験詳細	試験方法	暫定基準値、備考
一般特性	外観	目視	異物の付着、ラベル剥離等が無いこと<br>液漏れが無いこと
			色調に異常が無いこと、異物が混入していないこと
	細胞形態	顕微鏡観察	T 細胞様(浮遊細胞)形態
	生細胞数（製剤時）	細胞数計測	1×10^{7} 個／50mL チューブ以上
	生存率	FACS 解析	リンパ球ゲートに含まれる細胞の生存率(7AAD-)が 90%以上
	細胞表面マーカー発現	FACS 解析	CD45+細胞のうち、CD3 発現細胞が 80%以上、かつ、CD3+細胞が NKT 特異的 TCR(Vα24、Vβ11)を発現していること
安全性	細胞純度	PCR 法	未分化マーカー(LIN28)発現細胞 0.01%（検出限界）未満
	無菌	培養法	真菌、一般細菌陰性
		迅速法	真菌、一般細菌陰性（嫌気性細菌を除く）
	マイコプラズマ否定	PCR 法	陰性
	エンドトキシン	比濁法	基準値(0.3 EU/mL)未満
細胞機能	in vitro IFNγ産生能	ELISA	IFNγ の確認（目標値：陰性サンプルの平均値の 1.7 倍）
	in vitro 抗腫瘍効果	K562 細胞殺傷能	抗腫瘍効果の確認（目標値：7%）
特性解析試験			
安全性（不純物）	BSA 混入量	ELISA	<50 ng/dose (25 mL) @投与製剤
	マウスゲノム	PCR 法	検出限界未満 @投与製剤

表 7　マイクロコロニー法による試験用菌株の検出

試験用菌株		菌体添加量（計算値）	培地*	時間									
				12		24		48		72		120	
				コロニー数（平均）	回収率(%)**	コロニー数（平均）	回収率(%)	コロニー数（平均）	回収率(%)	コロニー数（平均）	回収率(%)	コロニー数（平均）	回収率(%)
好気性菌	S.aureus	10 個/filter	SCD	3.5	17.5	20.0	**100.0**	20.0	**100.0**	20.0	**100.0**	20.0	100.0
	P.aeruginosa			0.0	NA	25.0	**100.0**	25.0	**100.0**	25.0	**100.0**	25.0	100.0
	B.subtilis			4.5	90.0	5.0	**100.0**	5.0	**100.0**	5.0	**100.0**	5.0	100.0
嫌気性菌	C.sporogenes			0.0	NA	0.0	NA	1.0	33.3	3.0	**100.0**	3.0	100.0
真菌	C.albicans		SD	0.0	NA	1.0	66.7	1.5	**100.0**	1.5	**100.0**	1.5	100.0
	A.brasiliensis			0.0	NA	0.0	NA	1.0	**100.0**	1.0	**100.0**	1.0	100.0

n=2、32.5℃（S.aureus,　P.aeruginosa, B.subtilis, C.sporogenes）、22.5℃（C.albicans, A.brasiliensis）
*SCD：ソイビーン・カゼイン・ダイジェスト寒天培地、SD:サブローブドウ糖寒天培地
**回収率は 120 時間後のコロニー数を 100%としたときの相対値

1.2.2.7. iPS-NKT 細胞（製品）の安定性（表 8）

本品は純化培養工程の後、培地に懸濁して出荷されるが、時間と共に沈降による会合、細胞の失活等により生細胞数および活性の低下が認められるため、出荷後安定な期間は短く、長期安定性は保証されない。そこで、本品を治験実施施設に輸送した後、どの程度まで使用できるかを確認するため、まず培養状態を維持したまま治験実施施設に輸送し、施設内においてCO2インキュベーター内で保管する事を想定した実験を行った。本実験においては、本品を出荷した後、CO2インキュベーター内で培養時と同様にローラー培養装置で回転培養を行うかまたは静置し、出荷翌日に生細胞数、in vitro IFNγ産生能および in vitro 抗腫瘍効果を測定することにより、本品が使用可能かを判定することとした。細胞純度に関しては、これまでの試験において培養期間を延長しても変化が無いことが確認されているため実施しない事とした。無菌、マイコプラズマ否定およびエンドトキシンに関しては、容器を開封せず、混入の恐れが無いことから実施しない事とした。不純物に関しては、投与製剤時点で測定する項目であり、別の洗浄試験でバリデーションを実施したため、本試験では実施しない事とした。

その結果、表 9 に示したように出荷後 24 時間では、細胞機能は維持されるものの生細胞数が基準値未満となり、送付翌日の使用は望ましくないと判断した。そこで、生産施設（理研）から治験実施施設（千葉大学）までの細胞輸送および調剤にかかる時間を輸送試験により見積もったところ、3 時間を見込めば十分であることが明らかとなった。また、製品の千葉大学への到着から投与にかかる時間を見積もった結果、こちらも 3 時間を見込めば十分であることが明らかとなった。そこで、本品の輸送及び調剤に 3 時間、調剤後製剤の保存期間を 3 時間として、本品及び調剤後製剤の安定性試験を実施した。本試験は、本品を CO2 供給下で 37℃に保持したまま輸送ボックス中で保管し、3 時間後に投与製剤の調剤を行い、治験における投与濃度の調剤後製剤が得られるか、またその調剤後製剤が投与に必要な期間安定性を保持できるかを目的として行った。なお、調剤後製剤の細胞数は、調剤後の細胞数（2×10^6 個/mL）と容量から、50 mL あたりの細胞数を算出した。

その結果、表 10 に示すように、本品の出荷後 3 時間において細胞機能を維持した目標濃度（2.0×10^6 cells/mL）の調剤後製剤が得られることが明らかとなった。また、調剤後 3 時間であれば 4℃、25℃の何れにおいてもある程度細胞機能は維持されるが、25℃保管では生細胞数が不足するサンプルが存在する事、および in vitro 抗腫瘍活性が目標値を下回る可能性があることが明らかとなった。したがって、調剤後の保管条件は 4℃が望ましいと判断した。

以上の結果から、本品の安定性は出荷後 6 時間以内、かつ調剤化後 3 時間以内とした。また保管温度は 4℃（3℃±2℃）とした。

表 8　本品の安定性　（CO_2 インキュベーター）

規格試験				安定性試験（CO2 供給、37°C）					
試験項目	試験詳細	試験方法	暫定基準値、備考	出荷時		24 hr（輸送 2hr 後回転培養）		24 hr（輸送 2hr 後静置培養）	
				Lot.1	Lot.2	Lot.1	Lot.2	Lot.1	Lot.2
一般特	外観	目視	異物の付着、ラベル剥離等が無いこと、液漏れが無いこと	内部試験のためラベル貼付無し	内部試験のためラベル貼付無し	内部試験のためラベル貼付無	内部試験のためラベル貼付	内部試験のためラベル貼付	内部試験のためラベ

性						し	無し	無し	ル貼付無し
			色調に異常が無いこと、異物が混入していないこと	なし	なし	なし	なし	なし	なし
	細胞形態	顕微鏡観察	T 細胞様(浮遊細胞)形態	T 細胞様	T 細胞様	T 細胞様	T 細胞様	T 細胞様	T 細胞様
	生細胞数	細胞数計測	$1×10^7$ 個／50mL チューブ以上	$3.5×10^7$ 個	$3.6×10^7$ 個	$0.22×10^7$ 個	$0.19×10^7$ 個	$0.49×10^7$ 個	$0.56×10^7$ 個
	生存率	FACS 解析	リンパ球ゲートに含まれる細胞の生存率(7AAD-)が 90%以上	99.9%	未実施*1	99.7%	未実施*1	92.1%	未実施*1
	細胞表面マーカー発現	FACS 解析	CD45+細胞のうち、CD3 発現細胞が 80%以上、かつ、CD3+細胞が NKT 特異的 TCR(Vα24、Vβ11)を発現していること	92.9%	未実施*1	94.9%	未実施*1	93.0%	未実施*1
細胞機能	in vitro IFNγ 産生能	ELISA	IFNγ の確認（目標値:陰性サンプルの平均値の 1.7 倍）	8143pg/mL 9.42 倍	5953 pg/mL 17.54 倍	3305 pg/mL 6.09 倍	6726 pg/mL 20.37 倍	1366 pg/mL 4.88 倍	6712 pg/mL 7.92 倍
	in vitro 抗腫瘍効果	K562 細胞殺傷能	抗腫瘍効果の確認（目標値:7%）	31.6%	23.1%	17.0%	26.0%	10.7%	20.2%

*1: FACS 機器不調により未取得

表 9　本品および調剤後製剤の安定性

規格試験				安定性試験 （CO_2 供給、37℃）					
試験項目	試験詳細	試験方法	暫定基準値、備考	出荷時（本品）			3hr（調剤後製剤）		
				Lot.2	Lot.1	Lot.3	Lot.2	Lot.1	Lot.3
一般特性	外観	目視	異物の付着、ラベル剥離等が無いこと<br>液漏れが無いこと	内部試験のためラベル貼付無し			内部試験のためラベル貼付無し		
			色調に異常が無いこと、異物が混入していないこと	なし			なし		
	細胞形態	顕微鏡観察	T 細胞様（浮遊細胞）形態	T 細胞様			T 細胞様		
	生細胞数	細胞数計測	$1×10^7$ 個／50mL チューブ以上	$3.6×10^7$ 個	$3.5×10^7$ 個	$2.1×10^7$ 個	$3.0×10^7$ 個	$3.0×10^7$ 個	$1.5×10^7$ 個
	生存率	FACS 解析	リンパ球ゲートに含まれる細胞の生存率(7AAD-)が 90%以上	99.9	未実施*1	99.2	99.9	未実施*1	97.6
	細胞表面マーカー発現	FACS 解析	CD45+細胞のうち、CD3 発現細胞が 80%以上、かつ、CD3+細胞が NKT 特異的 TCR(Vα24、Vβ11)を発現していること	92.9	未実施*1	88.1	91.1	未実施*1	96.3
細胞機能	in vitro IFNγ産生能	ELISA	IFNγ の確認（目標値:陰性サンプルの平均値の 1.7 倍）	5953 pg/mL 17.54 倍	8143 pg/mL 9.42 倍	11289 pg/mL 29.46 倍	1663 pg/mL 9.76 倍	3174 pg/mL 13.35 倍	3784 pg/mL 9.93 倍
	in vitro 抗腫瘍効果	K562 細胞殺傷能	抗腫瘍効果の確認（目標値:7%）	23.1%	31.6%	19.4%	20.1%	12.0%	22.7%

*1: FACS 機器不調により未取得

表 10　調剤後製剤の安定性

規格試験				安定性試験														
試験項目	試験詳細	試験方法	暫定基準値、備考	調剤時（出荷から 3 hr）			3 hr（出荷から 6 hr）						21 hr（出荷から 24 hr）					
							4℃			25℃			4℃			25℃		
				Lot.2	Lot.1	Lot.3	Lot.2	Lot.1	Lot.3	Lot.2	Lot.1	Lot.3	Lot.2	Lot.1	Lot.3	Lot.2	Lot.1	Lot.3
一般	外観	目視	異物の付着、ラベル	内部試験のためラベル貼付無し														

特性			剥離等が無いこと液漏れが無いこと															
			色調に異常が無いこと、異物が混入していないこと	なし														
	細胞形態	顕微鏡観察	T 細胞様(浮遊細胞)形態	T 細胞様														
	生細胞数	細胞数計測	1×10^7 個／50mL チューブ以上（$\times10^7$ 個／50mL）	3.0×10^7	3.0×10^7	1.5×10^7	1.4×10^7	1.5×10^7	1.0×10^7	1.0×10^7	1.2×10^7	0.9×10^7	1.1×10^7	1.0×10^7	0.76×10^7	0.86×10^7	0.52×10^7	0.5×10^7
	生存率	FACS 解析	リンパ球ゲートに含まれる細胞の生存率(7AAD-)が 90%以上	99.9	未実施 *1	97.6	99.3	未実施 *1	99.3	99.5	未実施 *1	99.2	99.3	未実施 *1	未実施 *1	96.1	未実施 *1	未実施 *1
	細胞表面マーカー発現	FACS 解析	CD45+細胞のうち、CD3 発現細胞が 80%以上、かつ、CD3+細胞が NKT 特異的 TCR(Vα24、Vβ11)を発現していること	91.1	未実施 *1	96.3	90.4	未実施 *1	94.7	90.2	未実施 *1	95.6	89.5	未実施 *1	未実施 *1	92.0	未実施 *1	未実施 *1
細胞機能	in vitro IFNγ産生能	ELISA	IFNγ の確認（目標値：陰性サンプルの平均値の 1.7 倍）	1663 pg/mL 9.76 倍	3174 pg/mL 13.35 倍	3784 pg/mL 9.93 倍	1099 pg/mL 17.31 倍	3192 pg/mL 18.73 倍	1786 pg/mL 7.54 倍	793 pg/mL 19.20 倍	3670 pg/mL 17.01 倍	2185 pg/mL 9.49 倍	1116 pg/mL 3.36 倍	1019 pg/mL 5.82 倍	1047 pg/mL 11.29 倍	108 pg/mL 2.24 倍	684 pg/mL 9.84 倍	317 pg/mL 9.66 倍
	in vitro 抗腫瘍効果	K562 細胞殺傷能	抗腫瘍効果の確認（目標値：7%）	20.1%	12.0%	22.7%	9.5%	11.2%	13.9%	7.6%	2.9%	6.9%	13.6%	0%	3.5%	0%	0%	0%

*1: FACS 機器不調により未取得

1.2.2.8. 自家 DC/Gal の製造

製造の各工程の概略、各段階における規格試験の実施時期を示す。

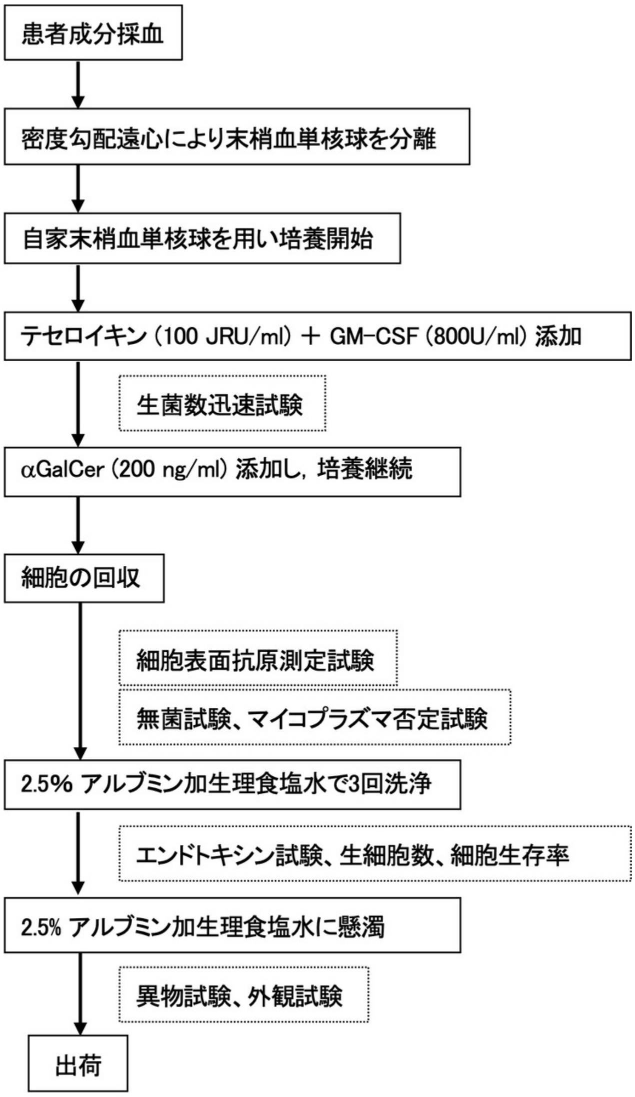

図 3　DC/Gal 調製のフローチャート

1.2.2.9. 自家 DC/Gal の規格試験

DC/Gal の製造・出荷に際して行う検査とその時期を示す。また表 11 に試験項目とそれら規格値／適否の判定基準を示す。

(1) 生細胞数：初回採血での単核球分画採取時および投与前
(2) 細胞生存率：投与前
(3) エンドトキシン（簡易法）：投与当日の出荷時
(4) 細胞表面抗原測定試験（FACS）：投与当日の出荷時
(5) 製剤の異物検査：製造時及び出荷時
(6) 製剤容器の外観検査：出荷時（直接容器、梱包の確認）
(7) 生菌数迅速試験：投与 3 日前に検査に提出する。
(8) エンドトキシン試験・無菌試験・マイコプラズマ否定試験：投与日に検査に提出する。細胞投与後に試験結果の陽性が判明した場合、直ちに実施責任者に連絡し、当該患者に関しては厳重に経過観察を行う。

表 11 自家 DC/Gal の規格試験と判定基準

試験項目名	試験方法	規格値/適否の判定
生細胞数	血球計算板を用いた測定	1×10^8 個以上
細胞生存率	トリパンブルーによる染色血球計算盤を用いて測定	60% 以上
生菌数迅速試験	メンブランフィルター法/迅速蛍光染色	陰性
無菌試験	メンブランフィルター法	陰性
エンドトキシン試験	ゲル化法／限度試験法	＜0.25 EU/mL
マイコプラズマ否定試験	NAT 法	陰性
エンドトキシン試験（簡易法）	カイネティック比色法（簡易法） による測定	＜1 EU/mL
細胞表面抗原測定試験	FACS 解析	CD45 陽性細胞のうち、CD86 発現細胞が 15%以上
製剤内の異物検査	目視及び顕微鏡検査	異物を認めない
製剤容器の外観検査	目視検査	直接容器、梱包に異常を認めない

1.2.2.10. 自家 DC/Gal の安定性試験

本品は 7 日間の培養工程の後、ヒトアルブミン添加生理食塩水に懸濁して出荷され、院内で迅速に鼻粘膜下に投与される。出荷時の細胞濃度が著しく高いため、時間と共に生細胞数の低下が認められることが予想され、長期安定性は保証されない。一方、細胞製造施設（千葉大学医学部附属病院未来開拓センター）から投与実施場所（千葉大学医学部附属病院耳鼻咽喉・頭頸部外科病棟）までの細胞輸送及び投与にかかる時間は 1 時間を見込めば十分である。そこで、本品を出荷後に室温で静置した後、どの程度の時間まで使用できるかを確認するため、出荷時の細胞濃度である 1×10^{8} 個/200μL の条件で室温（千葉大学医学部附属病院内室温 20~25℃前後）に静置した場合の細胞数と細胞表面抗原の経時的変化を計測した。

その結果、表 12 に示すように、本品の出荷後 4 時間においても、細胞表面抗原を維持しつつ出荷時の細胞数（1.0×10^{8} 個/200μL）が得られることが明らかとなった。

以上の結果から、本品の安定性は安全域を確保して、出荷後 2 時間以内とした。また温度は室温とした。

表 12　調製後の本品の安定性

規格試験				安定性試験											
試験項目	試験詳細	試験方法	基準値	調製時		1h		2h		3h		4h		24h	
				Lot.1	Lot.2	Lot.1	Lot.2	Lot.1	Lot.2	Lot.1	Lot.2	Lot.1	Lot.2	Lot.1	Lot.2
一般特性	生細胞数	細胞数計測	1×10^{8} 個／200µL	1.0×10^{8}	1.0×10^{8}	1.0×10^{8}	9.1×10^{7}	9.2×10^{7}	7.6×10^{7}	1.3×10^{8}	7.5×10^{7}	1.1×10^{8}	7.5×10^{7}	n.d.	1.6×10^{7}
	生存率	細胞数計測	トリパンブルー非染色細胞が60%以上	93.0	95.8	93.5	98.9	87.8	98.1	95.6	97.7	92.3	99.0	n.d.	20.9
	細胞表面マーカー発現	FACS解析	CD45+細胞のうちCD86を15%以上発現していること	n.d.	51.0	n.d.	60.3	n.d.	64.7	n.d.	66.6	n.d.	68.3	n.d.	67.2

1.2.3. 動物実験における有効性の検証

1.2.3.1. iPS-NKT 細胞の抗腫瘍効果（K562 細胞）

iPS-NKT 細胞の抗腫瘍効果を確認するために、ヒト白血病由来の細胞株であり、NK 細胞に対して感受性の高い K562 細胞にルシフェラーゼ遺伝子を導入した K562-luc 細胞を、超免疫不全マウスである NOG マウスの腹腔内に接種した。5 日後に K562-luc 細胞の増殖が確認されたマウスに対して iPS-NKT 細胞 3 ×10^6 を 13 日目まで 2 日ごとに腹腔内へ単独投与し、K562-luc 細胞数の増殖を体外から蛍光法で観察した。その結果、図 4 に示したように、iPS-NKT 細胞を投与したマウスにおいては K562-luc 細胞の増殖抑制が観察された。

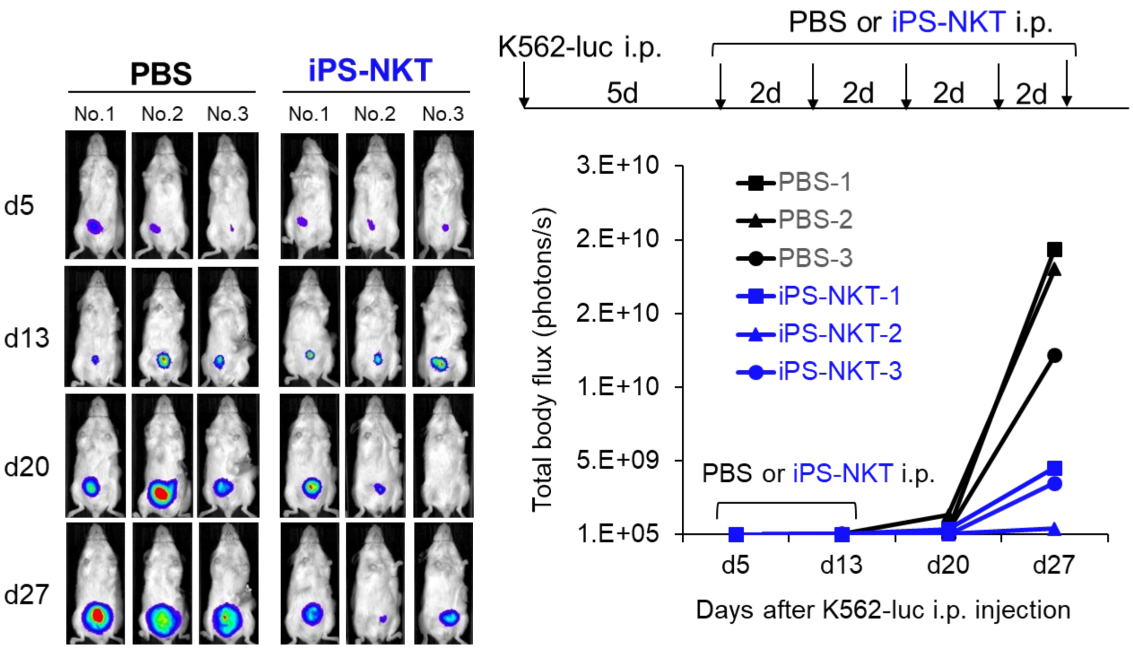

図 4　iPS-NKT 細胞の K-562-luc 細胞に対する抗腫瘍効果

1.2.3.2. iPS-NKT 細胞の抗腫瘍効果（FaDu 細胞）

iPS-NKT 細胞の抗腫瘍効果を確認するために、ヒト頭頸部がん由来の細胞株である FaDu 細胞にルシフェラーゼ遺伝子を導入した FaDu-luc 細胞 5×10^5 個を、超免疫不全マウスである NSG マウス皮下に接種し、13 日後に増殖が確認されたマウスに対して iPS-NKT 細胞 3×10^6 個を 21 日目まで 2 日ごとに腫瘍内へ単独投与した。13 日目の腫瘍サイズを 1 として、その後の腫瘍サイズの変化を観察した結果、図 5　iPS-NKT 細胞の FaDu-luc 細胞に対する抗腫瘍効果に示したように iPS-NKT 細胞を投与したマウスにおいて腫瘍サイズの増加抑制が観察された。

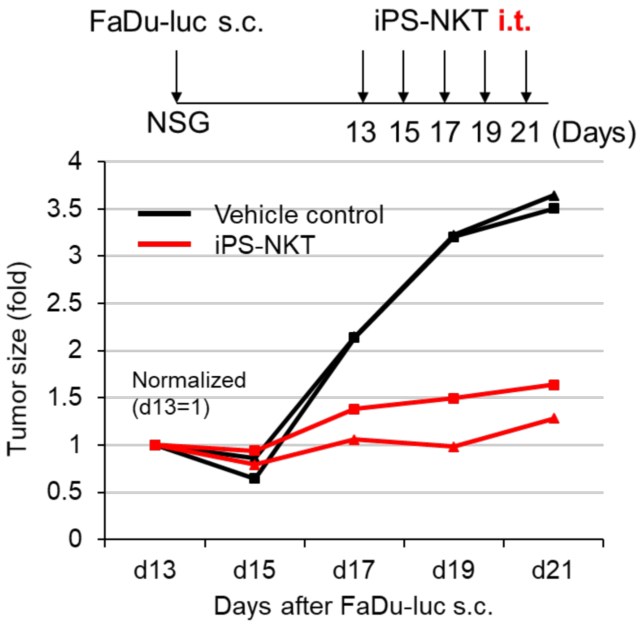

図 5　iPS-NKT 細胞の FaDu-luc 細胞に対する抗腫瘍効果

1.2.3.3. iPS-NKT 細胞の NK 細胞活性化能（アジュバント活性）

iPS-NKT 細胞が、ヒト NK 細胞を活性化する能力があるかを確認するために、iPS-NKT 細胞を、ヒト末梢血単核球を投与しておいた超免疫不全マウス（NOG マウス）に DC/Gal と共に投与し、NK 細胞の活性化を観察した。その結果、**図 6**　に示したように iPS-NKT 細胞を、末梢血単核球を投与しておいた NOG マウスに DC/Gal と共に投与した時のみ、ヒト NK 細胞の活性化が認められ、iPS-NKT 細胞による NK 細胞活性化（アジュバント活性）が確認された。

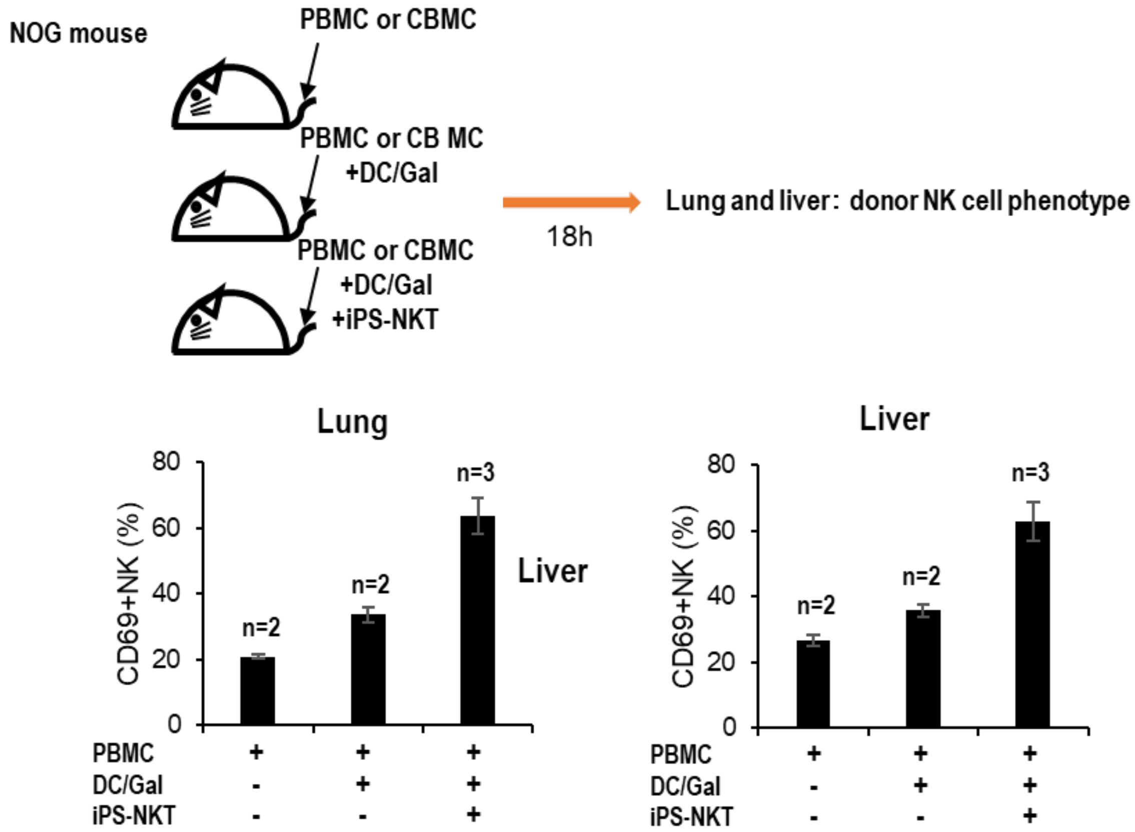

図 6　**iPS-NKT 細胞の NK 細胞活性化能（アジュバント活性）**

1.2.3.4. DC/Gal と iPS-NKT 細胞の併用

iPS-NKT 細胞および DC/Gal の併用により in vivo での抗腫瘍効果が増強されるかを検討するために、ヒト肺がん患者由来のがん細胞をヒト型 IL7/15 をノックインした NSG マウス皮下に接種し（PDX）、腫瘍増殖が確認されたマウスに対して iPS-NKT 細胞 3×10^{6} cells（1×10^{8} cells/kg）を 0 日から 8 日目まで 2 日ごとに腫瘍内へ投与した（緑矢印）。併用投与群では投与開始後 8 日目に、iPS-NKT 細胞に加えてマウス DC/Gal 1×10^{6} cells（3.3×10^{7} cells/kg）を同様に腫瘍内に投与した（赤矢印）。投与開始時の腫瘍サイズを元に、その後の腫瘍サイズの変化を観察した結果、**図 7　iPS-NKT 細胞および DC/Gal 併用投与によるヒト肺がん PDX に対する抗腫瘍効果**に示したように、iPS-NKT 細胞および DC/Gal を投与したマウスにおいて腫瘍サイズの増加抑制が観察された。また、投与開始後 8, 9, 10 日目に細胞傷害性因子の発現を観察した結果、FASLG, GZMA, TNF などの細胞傷害性分子の発現および GNLY, CCL3, CCL4 などのアジュバント効果に関連する遺伝子群の発現向上が認められた（**図 7　iPS-NKT 細胞および DC/Gal 併用投与によるヒト肺がん PDX に対する抗腫瘍効果**）。このことから iPS-NKT+DC/Gal 併用投与群は、iPS-NKT 単独投与群と比較して有意な抗腫瘍効果を示すことが確認された。

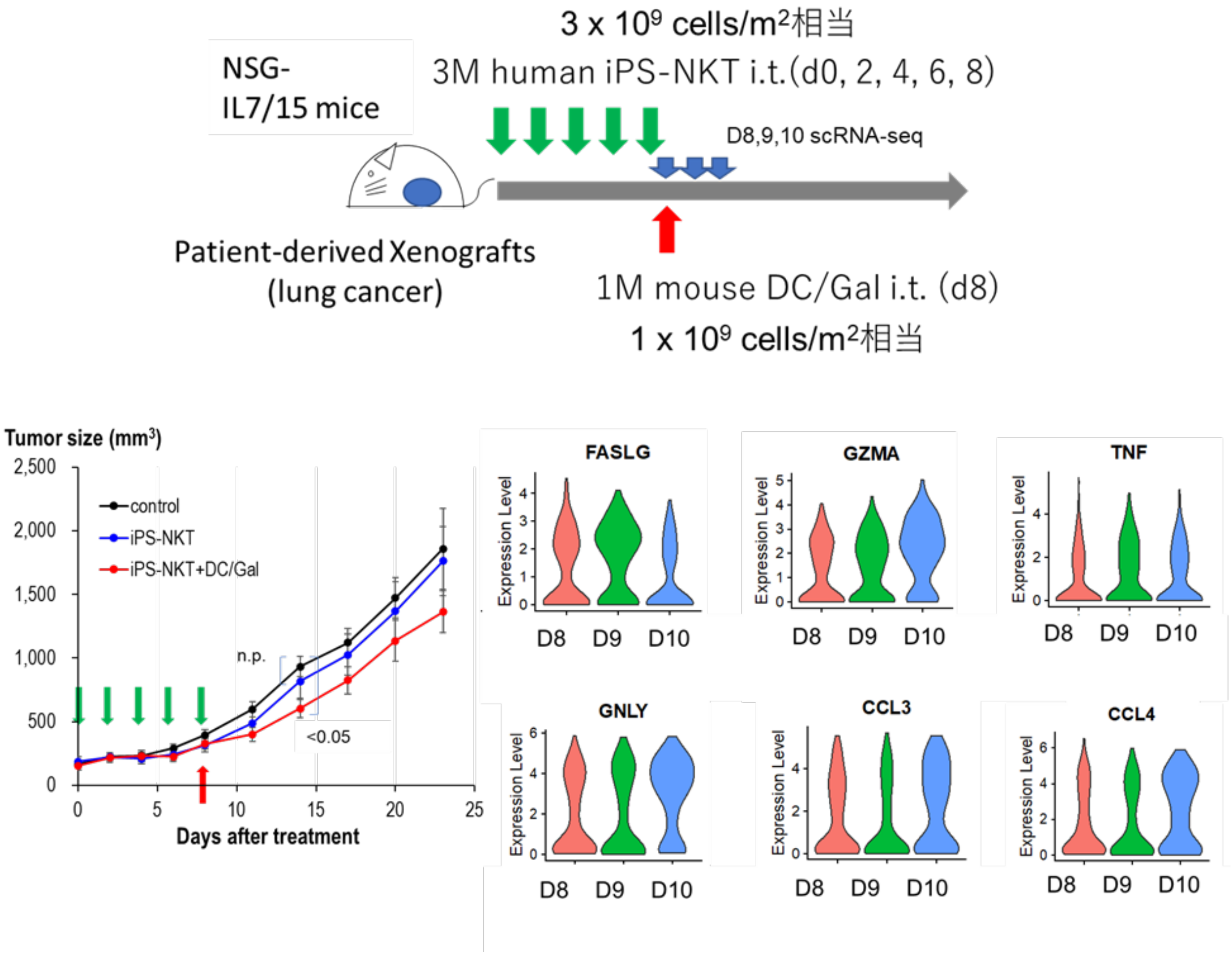

図 7　iPS-NKT 細胞および DC/Gal 併用投与によるヒト肺がん PDX に対する抗腫瘍効果

1.2.3.5. in vivo 抗腫瘍効果および腫瘍内免疫細胞解析（DC/Gal 併用、ヒト肺がん PDX）

iPS-NKT 細胞及び DC/Gal の併用により *in vivo* での抗腫瘍効果がどのように発揮されているかを確かめるために、ヒトの免疫細胞群の関与も評価可能なモデルでの検討を行った。ヒト肺がん患者由来のがん細胞をヒト型 IL7/15 をノックインした NSG マウス皮下に接種し（PDX）、腫瘍増殖が確認されたマウスに対して 0 日にヒト末梢血単核球（PBMC）を腫瘍内に投与し、その翌日に iPS-NKT 細胞 3×10^6 cells（1×10^8 cells/kg）を腫瘍内へ投与した。併用投与群では iPS-NKT 細胞投与と同じ日に、iPS-NKT 細胞に加えてマウス DC/Gal 1×10^6 cells（3.3×10^7 cells/kg）を同様に腫瘍内に投与した（図 8　PBMC, iPS-NKT 細胞および DC/Gal 併用投与によるヒト肺がん PDX に対する抗腫瘍効果上）。投与開始時の腫瘍サイズを元に、その後の腫瘍サイズの変化を観察した結果、PBMC に加え iPS-NKT 細胞及び DC/Gal を投与したマウスにおいてのみ腫瘍の増大抑制が観察された（図 8　PBMC, iPS-NKT 細胞および DC/Gal 併用投与によるヒト肺がん PDX に対する抗腫瘍効果 右下）。また治療開始後 14 日目の腫瘍内に残存する免疫細胞を解析した結果、そのほとんどが T 細胞であり、これは iPS-NKT 細胞と DC/Gal の併用投与によって惹起されたアジュバント効果によると考えられた（図 8　PBMC, iPS-NKT 細胞および DC/Gal 併用投与によるヒト肺がん PDX に対する抗腫瘍効果左下）。このことから iPS-NKT+DC/Gal 併用投与群は、iPS-NKT 単独投与群と比較して有意な抗腫瘍効果を示すことが確認された。

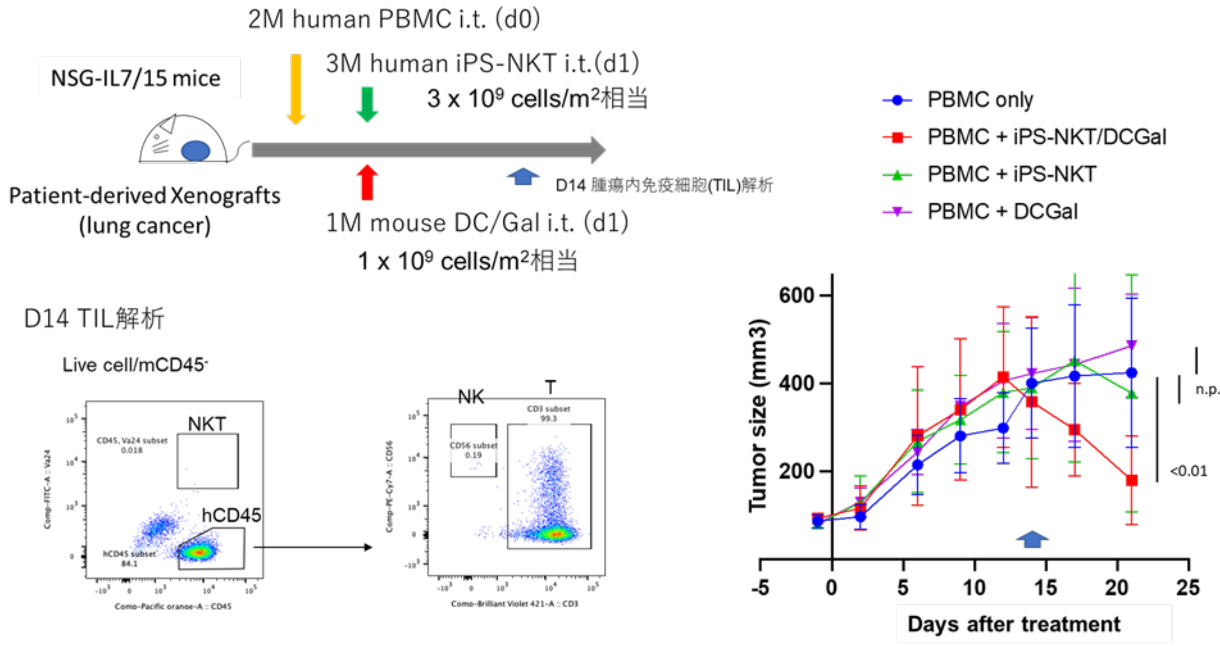

図 8　PBMC, iPS-NKT 細胞および DC/Gal 併用投与によるヒト肺がん PDX に対する抗腫瘍効果

1.2.4. 一般毒性試験及び造腫瘍性試験

1.2.4.1. iPS-NKT 細胞の一般毒性試験

iPS-NKT 細胞単独投与の治験は、頭頸部腫瘍の栄養動脈を経由して 1×10^7 から 1×10^8 cells/m^2 の用量で、2 週間程度の間隔で最大 3 回程度投与するよう計画した。また iPS-NKT 細胞と DC/Gal 併用投与の臨床研究に関しても、iPS-NKT 細胞の投与量は最大で 1×10^8 cells/m^2 で計画している。栄養動脈投与は、毛細血管が発達している頭頸部腫瘍への iPS-NKT 細胞の分布を期待したものである。マウスに投与する毒性試験の投与経路として腫瘍の栄養動脈は選択できないが、動態試験の予備試験において静脈内投与した iPS-NKT 細胞が、毛細血管が良く発達している肝臓、腎臓、脾臓等に分布することから、頭頸部腫瘍の栄養動脈投与においても腫瘍内に保持されない細胞は静脈内投与と同様にこれらの臓器に分布すると予想している。そこで、マウスへの投与経路として静脈内投与を選択し、投与量は臨床投与量の最大 10 倍となる、1×10^7 から 1×10^9 cells/m^2 とした。このヒト体表面積あたりの投与量をマウスの体重あたりの投与量に換算すると、3.3×10^6 cells/kg から 3.3×10^8 cells/kg となり、受託研究機関にヒアリングを行ったマウスへの投与量の限界にも近いため、安全性試験として妥当な投与量であると考えた。そこで 3.3×10^8 cells/kg を最大投与量として単回投与毒性試験を実施したところ、死亡例は無く、一般状態，Irwin の変法を用いた一般症状及び行動観察、体重、摂餌量、血液学的検査、血液生化学的検査、尿検査、剖検、器官重量及び病理組織学的検査に異常は認められなかった。したがって、iPS-NKT 細胞の無毒性量は雌雄とも 3.3×10^8 cells/kg と考えられた。

試験において、iPS-NKT 細胞の投与は単回のみであることから反復投与毒性試験は実施しないこととした。

1.2.4.2. DC/Gal の一般毒性試験

本被験物質のヒトへの投与は、1.0×10^8 個/body を経鼻粘膜下に 1 回予定している。被験物質の鼻粘膜下への投与は手技的に困難なため、経静脈的に全身に投与した際の毒性を検索することとした。α−ガラクトシルセラミドパルス樹状細胞 (DC/Gal)は、健常人血液ドナーの成分採血由来末梢血単核球を IL-2 および GM-CSF を添加した培地で 1 週間培養および 2 週間培養したものを用い、出荷後 21 ~ 23 時間で雌雄のヌードラット (F344/NJcl-rnu/ rnu) に静脈内投与し，亜急性および亜慢性全身毒性を評価した。投与量として、臨床で想定される投与細胞数の 50 倍以上に相当する 8.5×10^7 ~ 1.7×10^8 個/body 程度の細胞を，おおよその体重増加量に応じて体表面換算し、投与細胞数とした。その結果，DC/Gal 群の 4 週剖検群ならびに 13 週剖検群において，雌雄ともに一般状態，体重，摂餌量，眼科学的検査，尿検査，血液学的検査，血液化学的検査，剖検所見，器官重量および病理組織学的検査において，被験物質投与に関連する変化は認められなかった。以上のことから，本試験条件下において，α−ガラクトシルセラミドパルス樹状細胞には毒性はないと考えられた。

1.2.4.3. 併用投与毒性試験（NKT-deficient B6，ヒト型 IL7/15 発現 NSG マウス）

ホストの免疫細胞に与える影響を評価するため、iPS-NKT 細胞を 1~2×10^6 cells/mouse（0.5-1×10^8 cells/kg）、マウス DC/Gal を 1×10^6 cells/ mouse（0.5×10^8 cells/kg）の用量で NKT-deficient B6 (Ja18KO)マウスに 2 週間毎 3 回（0, 14, 28 日目）静脈内投与し、その後 42 日目まで観察した。対照群は iPS-NKT 細胞単独投与群および wild-type B6 マウス DC/Gal 単独投与群とした。投与期間を通じて、すべての投与群で死亡動物は観察されず、wild-type B6 マウス DC/Gal 単独投与群と比べて、iPS-NKT 細胞単独群、DC/Gal 併用投与群において炎症性サイトカインであるインターフェロンγ、IL-6 および IL-1b の上昇は認められなかった。

また iPS-NKT 細胞の移植片対宿主病の有無を評価するため、ヒト NKT 細胞を長期に維持可能なヒト型 IL7/15 発現 NSG マウスに iPS-NKT 細胞を 2×10^6 cells/mouse（1×10^8 cells/kg）、マウス DC/Gal を 1×10^6 cells/mouse（0.5×10^8 cells/kg）の用量で投与し、初回投与から 70 日目まで観察した。対照群は iPS-NKT 細胞単独群、DC/Gal 単独投与群、T 細胞単独群とした。iPS-NKT 細胞単独群、DC/Gal 併用投与群で一般状態、体重、剖検および組織検査においても、投薬起因性の毒性変化は認められなかった。

以上のことから、iPS-NKT 細胞および DC/Gal 併用投与時の無毒性量は 2×10^6 cells/mouse（1×10^8 cells/kg）を上回るものと推定された。

1.2.4.4. 造腫瘍性試験

造腫瘍性試験に関しては、はじめに *in vitro* 試験として軟寒天コロニー試験を実施したが、増殖は認められなかった。また、造腫瘍性が懸念されるゲノム所見に関して、原料となる NKT-iPS 細胞およびこれから製造した iPS-NKT 細胞に対して核型解析、腫瘍関連遺伝子（COSMIC CGC Tier1 および Sibata list）および構造異常（コピー数異常）の検査を実施したが、いずれも異常を認めなかった。*in vivo* 造腫瘍性試験を行うにあたり、ヒト細胞である iPS-NKT 細胞は免疫機能が正常なマウス中では拒絶により維持でき

ないため、高度免疫不全動物であり、薬効試験にも使用している NOG マウスを使用した。マウスに形成される iPS-NKT 細胞製剤由来の腫瘍としては、iPS-NKT 細胞に残留する未分化 iPS 細胞由来の奇形腫及び iPS-NKT 細胞に含まれる、又は iPS-NKT 細胞が形質転換したことによる腫瘍細胞由来の腫瘍が考えられるため、iPS 細胞及び iPS-NKT 細胞製剤を対象とした 2 種類の試験を実施している。投与経路に関しては、iPS-NKT 細胞が比較的小型の細胞であり、静脈内投与後肝臓、腎臓等に集積することから、これらの臓器内での造腫瘍性を確認するためにも静脈内投与とした。

iPS-NKT 細胞製剤に含まれる iPS 細胞は、未分化マーカーである LIN28 を対象として測定することにより、0.01%の混入を検出することが可能である。これは、臨床での最大予想投与量である 1×10^{8} cells/m^{2}（マウスにおける体重あたり投与量 3.3×10^{7} cells/kg）において 1×10^{4} cells/m^{2}（マウスにおける体重あたり投与量 3.3×10^{3} cells/kg）の iPS 細胞が混入する可能性があることを示している。したがって、混入が予想される量よりも多量の iPS 細胞を投与して奇形腫が形成されなければ、iPS-NKT 細胞製剤に混入する可能性のある iPS 細胞の量では奇形腫が形成されないことを証明できると考えた。そこで、投与量として iPS-NKT 細胞の単回投与毒性試験における予備試験で安全性が確認されている 3.3×10^{8} cells/kg を設定した。また、混入予想量の 10 万倍の投与量を設定することから、観察期間としては WHO TRS-878 section B.2.3.7 に従い、12 週間で実施した。その結果、NKT-iPS 細胞には造腫瘍性は認められず、ヒト細胞の残存も認められなかった。したがって、iPS-NKT 細胞製剤に混入する可能性のある iPS 細胞の量では奇形腫が形成されないことが証明された。PMDA との対面助言（平成 29 年 3 月 1 日実施、＃再戦確 P61-2）に基づき、新規に樹立した試験用 iPS 細胞株（HV#2）に関しても同様の試験を実施し、造腫瘍性およびヒト細胞の残存は認められなかった。したがって、iPS-NKT 細胞製剤に混入する可能性のある iPS 細胞の量では奇形腫は形成されないと考える。

iPS-NKT 細胞に由来する悪性形質転換細胞の造腫瘍性に関しては、可能な限り多くの細胞を投与し、可能な限り長期に観察することが求められている。そこで、投与量として iPS-NKT 細胞の単回投与毒性試験における予備試験で安全性が確認されている 3.3×10^{8} cells/kg を設定した。観察期間としては、NOG マウスを長期飼育した場合のバックグラウンドデータが明らかになっていないため、適正な期間が想定できないが、一部の受託試験会社では 1 年まで長期飼育を実施しており、終了時の死亡は 20 匹あたり 1 匹のみで他のマウスは健康であったとの情報を得ているため、1 年間とした。また使用動物数は、1 年後の生存率から 99.9%信頼区間での予想最低生存率を求めた結果、1 群 13 匹以上を用いれば 1 年後 10 匹の生存が確実になると予想されたため、これに更に 1 匹を追加して 1 群 14 匹で実施した。その結果、投与 52 週間後の病理組織学検査において、すべての動物（死亡/瀕死期剖検動物を含む）で被験物質投与に起因した腫瘍性変化は認められなかった。なお、iPS-NKT 投与群の雄 1 例の肺に、抗ヒト核抗体陽性細胞がみられたが、抗 Ki-67 抗体に陽性を示す細胞はみられなかった。従って、ヒト iPS-NKT 細胞は 3.3×10^{8} cells/kg で造腫瘍性は無いものと判断した。

iPS-NKT 細胞の適応はがんであるため、癌原性、遺伝毒性、生殖毒性試験は実施しない。また治療における投与はがんの栄養動脈への投与を計画しているため、局所刺激性試験も実施しないこととする。

現在までに実施した毒性試験及び造腫瘍性試験の概要を表 13 に示す。

表 13　非臨床安全性試験の総括表

試験項目名	被験物質等	試験系	投与方法	試験結果	実施状況	備考
単回投与毒性試験（予備試験）	ヒト iPS-NKT	ヌードマウス（BALB/c-nu）	単回<br>1×10^{6},	死亡例なし、一般状態、体重、血液学、血	終了	Non-GLP

		6 週齢<br>群構成：<br>雌雄各 5 匹/群×3 群<br>（媒体対照群，被験物質群 2 用量）<br>観察期間：15 日間	1×10^7 cells/kg,<br>静脈内投与	液化学、器官重量及び剖検で異常なし		[参考資料iii.]
	ヒト iPS-NKT	マウス（BALB/c）<br>6 週齢<br>群構成：<br>雌雄各 3 匹/群×5 群<br>（媒体対照群，被験物質群 3 用量）<br>観察期間：8 日間	単回<br>3.3×10^6,<br>3.3×10^7,<br>3.3×10^8 cells/kg<br>静脈内投与<br>（60-70 秒/匹）	死亡例無し、<br>一般状態、体重、剖検で異常なし	終了	Non-GLP<br>[参考資料iv.]
単回投与毒性試験（拡張型、コアバッテリー試験組み込み）	ヒト iPS-NKT 製剤	マウス（NOG）<br>6 週齢<br>群構成：<br>雌雄各 10 匹/群×4 群<br>（媒体対照群，被験物質群 3 用量）<br>観察期間：15 日間	単回<br>3.3×10^6,<br>3.3×10^7,<br>3.3×10^8 cells/kg<br>静脈内投与	死亡例無し、一般状態、Irwin の変法を用いた一般症状及び行動観察、体重、摂餌量、血液学的検査、血液生化学的検査、尿検査、剖検、器官重量及び病理組織学的検査で異常なし	終了	再生医療等製品GLP<br>[参考資料v.]
反復投与毒性試験	ヒト DC/Gal	ヌードラット<br>(F344/NJcl-*rnu*/ *rnu*)	反復(0,7, 42, 49d)<br>8.5×10^7 ~ 1.7×10^8 個/body<br>静脈内投与	一般状態，体重，摂餌量，眼科学的検査，尿検査，血液学的検査，血液化学的検査，剖検所見，器官重量および病理組織学的検査で異常なし	終了	再生医療等製品GLP<br>[参考資料vi]
併用投与毒性試験	ヒト iPS-NKT<br>マウスDC/Gal	NKT-deficient B6<br>(Ja18KO)	反復(0,14,28d)<br>1×10^9,<br>2×10^9 cells/m^2<br>(iPS-NKT)<br>1×10^9 cells/m^2<br>(DC/Gal)<br>静脈内投与	42 日目まで<br>死亡例無し、体重増加率、臨床所見異常なし、炎症性サイトカイン放出なし	終了	Non-GLP
	ヒト iPS-NKT<br>マウスDC/Gal	ヒト型IL7/15発現NSGマウス	反復(0,14,28d)<br>2×10^9 cells/m^2<br>(iPS-NKT)<br>1×10^9 cells/m^2<br>(DC/Gal)<br>静脈内投与	70日目まで<br>体重減少なし<br>GVHD症状なし	終了	Non-GLP
軟寒天コロニー試験	ヒト iPS-NKT	軟寒天培地	1×10^6 cells/well で播種	足場非依存性増殖能なし	終了	再生医療等製品GLP<br>[参考資料vii.]
ゲノム検査	ヒト NKT-iPS および iPS-NKT	核型解析	50 cells	核型異常なし	終了	Non-GLP
		（G-Band 法）	1×10^6 cells	病原性が報告される変異なし	終了	Non-GLP
		COSMIC CGC Tier1 および Shbata list における遺伝子変異のエクソーム解析	1×10^6 cells	コピー数異常なし	終了	Non-GLP
造腫瘍試験	ヒト NKT-iPS	マウス（NOG）<br>6 週齢<br>群構成：<br>雌雄各 10 匹/群×2 群<br>（媒体対照群，被験物質群 1 用量）<br>観察期間：12 週間	単回<br>3.3×10^8 cells/kg<br>静脈内投与	造腫瘍性無し<br>ヒト細胞残存無し	終了	再生医療等製品GLP

						[参考資料viii.]
	ヒト NKT-iPS（新規取得株 HV #2）	マウス(NOG)<br>6 週齢<br>群構成：<br>雌雄各 10 匹/群×2 群<br>(媒体対照群，被験物質群 1 用量)<br>観察期間：12 週間	単回<br>3.3×10^8cells/kg<br>静脈内投与	造腫瘍性無し<br>ヒト細胞残存無し	終了	再生医療等製品 GLP [参考資料ix.]
	ヒト iPS-NKT 製剤	マウス（NOG）<br>6 週齢<br>群構成：<br>雌雄各 14 匹/群×2 群<br>（媒体対照群，被験物質群 1 用量）<br>観察期間：1 年間	単回<br>3.3×10^8 cells/kg<br>静脈内投与	造腫瘍性無し<br>抗ヒト核抗体陽性 1 例、抗 Ki-67 抗体陰性	終了	再生医療等製品 GLP [参考資料x.]

1.2.5. 薬理試験、動態試験

1.2.5.1. iPS-NKT 細胞の IFN-γ及び IL-4 産生能

iPS-NKT 細胞のアジュバント作用の指標として重要と考えられているサイトカインの産生パターン及び産生量を通常の NKT 細胞（NKT line 細胞）と比較するために、iPS-NKT 細胞及び NKT line 細胞を DC 又は NKT 細胞のリガンドとして知られている α-GalCer をパルスした樹状細胞（DC/Gal）と共培養し、24 時間後に上清中のサイトカイン（IFN-γ及び IL-4）量を測定した。その結果、iPS-NKT 細胞は、通常の NKT line 細胞に比べて、免疫を活性化する IFN-γの産生量が多い一方、免疫を抑制する側にも働く IL-4 産生能が低いことが明らかとなった（図 9　iPS-NKT 細胞の IFN-□及び IL-4 産生能）。したがって、iPS-NKT 細胞は NKT line 細胞に比べて免疫を活性化する効果が強いことが期待できる。

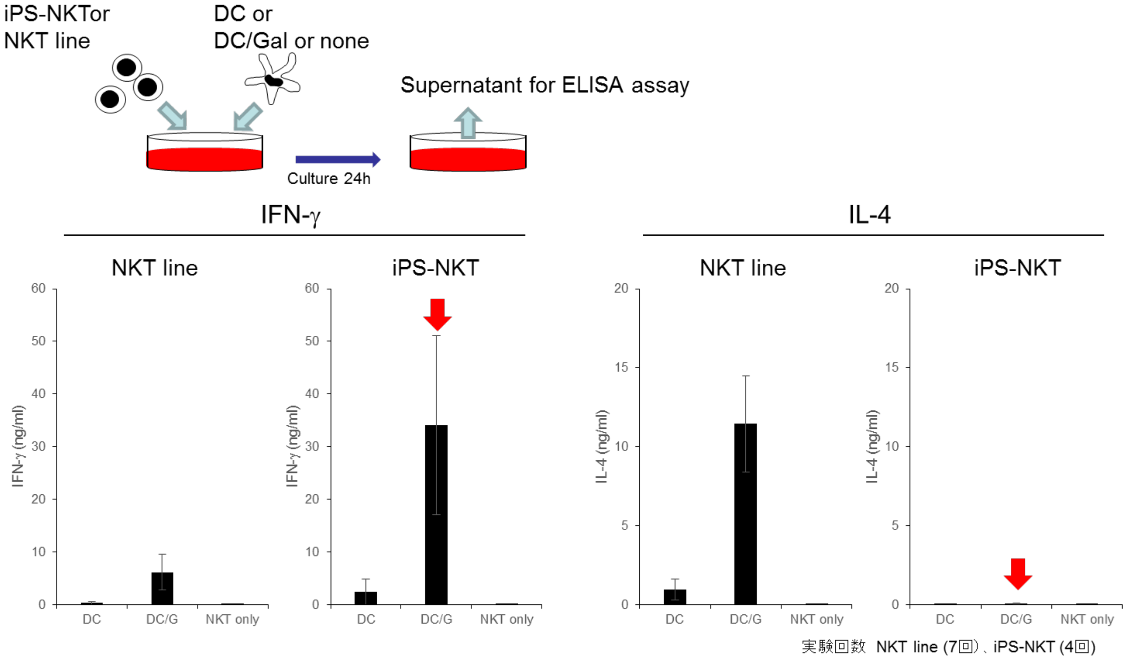

図 9　iPS-NKT 細胞の IFN-γ及び IL-4 産生能

1.2.5.2. iPS-NKT 細胞の *in vitro* 抗腫瘍効果

iPS-NKT 細胞が *in vitro* において直接的な抗腫瘍効果を持つか確認するために、iPS-NKT 細胞を腫瘍細胞 K562 及び非腫瘍コントロール細胞 Auto-PHA blast と共培養し、腫瘍細胞の殺細胞活性を観察した。その結果、iPS-NKT 細胞は、K562 に対して非常に強い殺細胞効果を示したが、Auto-PHA blast への殺細胞効果は弱かった。NKT line 細胞には殺細胞効果は認められなかった。そこで 6 種類の腫瘍細胞株（K562; ヒト白血病、NCI-H460;ヒト大細胞癌、A549; ヒト肺細胞基底上皮腺癌、HT-29;ヒト結腸癌、COLO205;ヒト大腸癌、Detroit562; ヒト咽頭癌）を用いて NKT 細胞及び iPS-NKT 細胞の抗腫瘍効果を評価した結果、iPS-NKT 細胞は 6 種類の腫瘍細胞株すべてに対して強い抗腫瘍効果を示すことが明らかとなった（図 10 iPS-NKT 細胞の抗腫瘍活性）。

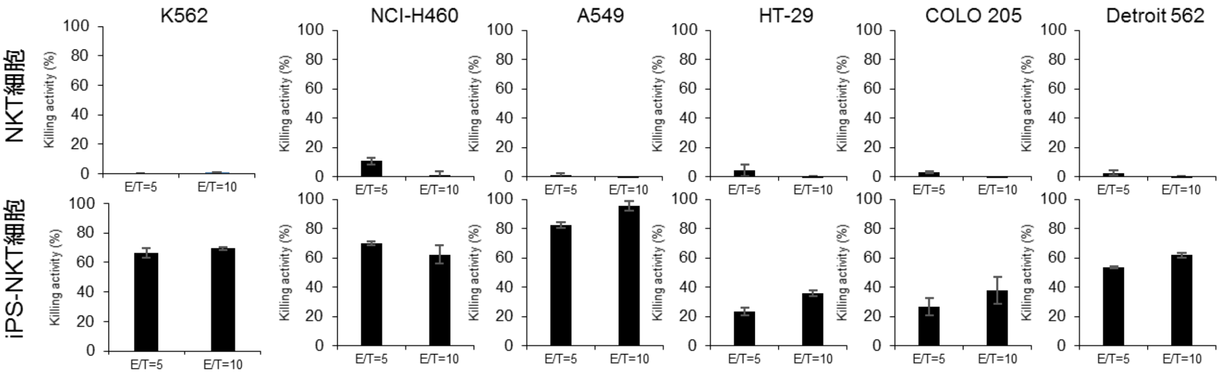

図 10　iPS-NKT 細胞の抗腫瘍活性

1.2.5.3. iPS-NKT 細胞の動態試験

マウス中においてヒト由来 NKT 細胞を検出するための試験はいまだ一般化されていないため、まず、測定法の適否及び細胞濃度推移の概略を確認するための予備試験を実施し、^{51}Cr ラベルによる動態試験を選択した。予備試験において肝臓中への集積が観察されたため、本試験においては観察期間をヒトの反復投与期間として想定している 2 週間まで延長して実施した。その結果、表 15 に示したように、iPS-NKT 細胞は免疫正常マウスに静脈内投与した場合、まず肺に一時的に集積した後、全身に分布すると考えられた。血液中の放射能は投与 48 時間後には 1%未満（0.33%）まで減少しており、その後は観察されなかったため、臓器に移行した放射能は血液中には戻ってこないことが示唆された。分布する臓器としては、脾臓、肝臓、大腿骨、腎臓及び骨髄が顕著であり、特に肝臓では投与 48 時間後においても投与放射能の 27%が残存しており、2 週間後（336 時間後）においても 21%が残存することが明らかとなった。蛍光ラベルした iPS-NKT 細胞を用いて理研内で実施した動態試験では、投与後 3 日目に肺及び脾臓において iPS-NKT 細胞が検出されているものの、肝臓からはほとんど検出されていないことを考慮すると、肝臓において観察されている放射能は、投与された iPS-NKT 細胞を貪食した宿主の免疫細胞に由来しており、iPS-NKT 細胞自身ではないと考えられた。

これを証明するために iPS-NKT 細胞を投与後のマウス臓器を摘出し、細胞を分離した後セルソーターでヒト NKT 細胞を検出する試験を実施したところ、投与後 4 時間目に血液及び肺において僅かに検出さ

れた（1 - 3 cells/サンプル、4 cells/mL 及び 31 cells/g）が、肝臓、脾臓及び腎臓からは検出されず（表 16）、またそれ以降の時点においても検出されなかった。

次に、非臨床安全性試験に使用した NOG マウスを用いて上記と同様にセルソーターを用いた動態試験を実施した結果、投与された iPS-NKT 細胞は、臓器重量（g）あたり、血液では投与後 24 時間で最大 3 個、肝臓では投与後 4 時間で最大 1 個、24 時間で最大 1 個、48 時間で最大 1 個、腎臓では投与後 48 時間で最大 3 個、肺では投与後 4 時間で最大 29 個の分布が見られたが、投与後 96 時間以降では血液及び各臓器から消失していた（表 17）。

したがって、ラベル体を用いた動態試験において観察された肝臓への分布は放射能のみで iPS-NKT 細胞の分布を反映しておらず、実際には iPS-NKT 細胞はほぼ消失していると判断した。

表 14　非臨床動態試験の総括表

試験項目名	被験物質等	試験系	投与方法	試験結果	実施状況	備考
動態試験	ヒト iPS-NKT（蛍光ラベル）	NOG マウス	静脈内投与	投与後 3 日目に肺及び脾臓で iPS-NKT 細胞を検出	終了	Non-GLP
	ヒト iPS-NKT（蛍光ラベル）	ヒト型 IL7/15 ノックイン NOG マウス	3.3×10^{8} cells/kg 静脈内投与	すべての測定点で定量限界未満	実施中	Non-GLP
	ヒト iPS-NKT（^{111}IN,^{51}Cr）	マウス（BALB/c）<br>6 週齢<br>群構成：<br>雌雄各 3 匹/群×4 群<br>（対照群，被験物質群 3 用量）	単回<br>3.3×10^{6},<br>3.3×10^{7},<br>3.3×10^{8} cells/kg<br>静脈内投与<br>（60-70 秒/匹）	肺に集積した後、脾臓、肝臓、腎臓及び骨髄に顕著に分布	終了	Non-GLP
	ヒト iPS-NKT（^{51}Cr）	マウス（BALB/c）<br>7 週齢<br>群構成：<br>雄 3 匹/群×6 群	単回<br>1.7×10^{7} cells/kg<br>静脈内投与	脾臓、肝臓、大腿骨、腎臓及び骨髄に分布<br>投与後 336 時間において，肝臓に投与放射能量の約 21%が残存<br>（**エラー！ 参照元が見つかりません。**12）	終了	信頼性基準
	ヒト iPS-NKT（セルソーターで検出）	マウス（BALB/c）<br>7 週齢<br>群構成：<br>雄 3 匹/群×3 群	単回<br>1.7×10^{7} cells/kg<br>静脈内投与	投与後 4 時間の肺で 31cell 相当/g 検出	終了	信頼性基準
	ヒト iPS-NKT（セルソーターで検出）	マウス（NOG）<br>7 週齢<br>群構成：<br>雄 3 匹/群×6 群	単回<br>3.3×10^{8} cells/kg<br>静脈内投与	投与 4 時間後に肺、血液、腎臓、肝臓に検出、96 時間までに消失	終了	信頼性基準

表 15　Percentage distribution of radioactivity in tissues （% of dose） after a single intravenous administration of [^{51}Cr]labeled iPS-NKT cell to mice

Tissue	Percentage distribution（% of dose）					
	4 h	24 h	48 h	96 h	168 h	336 h
Blood	1.89	0.69	0.33	0.00	0.00	0.00
Brain	0.01	0.00	0.00	0.00	0.00	0.00
Mandibular gland	0.03	0.02	0.03	0.03	0.01	0.02
Thymus	0.00	0.00	0.00	0.00	0.00	0.00
Heart	0.06	0.03	0.03	0.02	0.02	0.02
Lung	3.11	0.26	0.27	0.16	0.34	0.07
Liver	30.08	29.52	26.99	26.01	24.44	20.58
Kidney	2.30	2.04	1.62	1.42	0.92	0.48

Spleen	2.62	3.78	3.20	2.37	2.68	2.42
Pancreas	0.07	0.03	0.04	0.03	0.01	0.02
Skin（abdominal）	3.24	2.01	1.93	1.58	1.33	1.08
Skeletal muscle	2.32	1.00	0.99	0.72	0.60	0.52
Bone marrow	3.03	2.92	3.41	2.89	2.89	3.29
Testis	0.04	0.03	0.04	0.04	0.04	0.03
Prostate gland	0.00	0.00	0.00	0.00	0.00	0.00
Stomach	0.03	0.02	0.03	0.02	0.02	0.02
Small intestine	0.27	0.14	0.12	0.08	0.07	0.05
Large intestine	0.12	0.06	0.07	0.04	0.04	0.03

Data are expressed as the mean values of three animals.

表 16　Balb/c マウスに iPS-NKT 細胞を投与 4 時間後の臓器中 iPS-NKT 細胞数のセルソーターによる測定

臓器	動物番号	細胞数		
		イベント数	(Cells)	(Cells/g or mL)
血液	YH2M01	1	1	3
	YH2M02	1	1	4
	YH2M03	1	1	4
肝臓	YH2M01	0	0	0
	YH2M02	0	0	0
	YH2M03	0	0	0
腎臓	YH2M01	0	0	0
	YH2M02	0	0	0
	YH2M03	0	0	0
肺	YH2M01	3	7	51
	YH2M02	1	3	21
	YH2M03	1	3	21
脾臓	YH2M01	0	0	0
	YH2M02	0	0	0
	YH2M03	0	0	0

表 17　NOG マウスに iPS-NKT 細胞を投与後の臓器中 iPS-NKT 細胞数のセルソーターによる測定

投与後時間	iPS-NKT 細胞数 (cells/mL or g)			
(時間)	血液	肝臓	腎臓	肺
4	0	1	0	7
	0	0	0	29
	0	1	0	0
24	0	0	0	0
	0	0	0	0
	3	1	0	0
48	0	0	0	0
	0	0	3	0
	0	1	0	0
96	0	0	0	0
	0	0	0	0
	0	0	0	0
336	0	0	0	0
	0	0	0	0
	0	0	0	0

1.2.6. 類似製品の臨床試験（有効性と有害事象）

千葉大学医学部附属病院において、非小細胞肺癌及び頭頸部扁平上皮癌を対象に、DC/Gal 単独並びに、DC/Gal 及び NKT 細胞を用いた臨床試験が実施されており、高い安全性及び良好な有効性が確認されている[参考資料 x.~xvi.]。実施された試験の一覧を表 18、概要を図 11～図 12 頭頸部がんに対して行った NKT 細胞療法の概要に示す。

表 18 これまでに実施された NKT 細胞標的療法の一覧

試験	試験デザイン/<br>用法・用量	被験者数	主な目的	主な結果
標準治療終了後の切除不能進行・再発非小細胞肺癌を対象とした DC/Gal 療法の第 I 相試験	非盲検、用量漸増<br>DC/Gal $5x10^7$、$2.5x10^8$ 又は $1x10^9$ /m^2/週で 2 回静脈内投与後、4 週間休薬し、再度週 1 回で 2 回、計 4 回投与	9 例<br>（各用量 3 例）	安全性<br>免疫応答性<br>抗腫瘍効果	重篤な有害事象は認められなかった。細胞投与 1 又は 2 回後、高用量群において、末梢血液中の NKT 細胞数及び IFN-γの産生増強が認められた。高用量群 3 例全例において、23~26 週にわたり腫瘍の増悪が認められなかった。<br>[参考資料 xi.]
標準治療終了後の切除不能進行・再発非小細胞肺癌を対象とした DC/Gal 療法の第 I / II 相試験	非盲検、単用量<br>DC/Gal $1x10^9$ /m^2/週で 2 回静脈内投与後、4 週間休薬し、再度週 1 回で 2 回、計 4 回投与	17 例	安全性<br>免疫応答性<br>抗腫瘍効果	重篤な有害事象として、試験治療との関連のない深部静脈血栓症 1 例が認められた。<br>αGalCer 反応性 IFN- γ産生細胞数（NKT 細胞活性化）が増加した 10 例において、非増加例 7 例と比較して有意な OS の延長が認められた。<br>[参考資料 xii.]
標準治療終了後の切除不能進行・再発非小細胞肺癌を対象とした NKT 細胞療法の第 I 相試験	非盲検、用量漸増<br>NKT 細胞 $1×10^7$ 又は $5×10^7$ /body/週で 2 回静脈内投与	6 例<br>（各用量 3 例）	安全性<br>免疫応答性<br>抗腫瘍効果	重篤な有害事象は認められなかった。細胞投与 1 又は 2 回後、高用量群 3 例において、αGalCer 反応性 IFN-γ産生細胞数の増加が認められ、そのうちの 2 例において腫瘍の増大が 9 カ月及び 12 カ月の間、認められなかった。<br>[参考資料 xiii.]
標準治療終了後の切除不能進行・再発頭頸部扁平上皮癌を対象とした DC/Gal 療法の第 I 相試験	非盲検、単用量<br>DC/Gal $1x10^8$ /body/週、計 2 回鼻粘膜下投与	9 例	安全性<br>免疫応答性<br>抗腫瘍効果	重篤な有害事象は認められなかった。8/9 例において、αGalCer 反応性 IFN-γ 産生細胞数の増加が認められた。1/9 例において、部分奏効が認められた。<br>[参考資料 xiv]
標準治療終了後の切除不能進行・再発頭頸部扁平上皮癌を対象とした DC/Gal 及び NKT 細胞を用いた複合免疫療法の第 I / II 相試験	非盲検、単用量<br>DC/Gal $1x10^8$ /body/週、計 2 回鼻粘膜下投与後、NKT 細胞 $5x10^7$ /body を腫瘍栄養動脈内に単回投与	8 例	安全性<br>免疫応答性<br>抗腫瘍効果	重篤な有害事象として、Grade 3 の咽頭皮膚瘻（急速な腫瘍縮小に伴う）1 例が認められた。<br>5/8 例において、αGalCer 反応性 IFN-γ 産生細胞数の増加が認められた。3/8 例において、部分奏効が認められた。<br>[参考資料 xv]
手術可能な局所再発頭頸部扁平上皮癌を対象とした DC/Gal 及び NKT 細胞を用いた複合免疫療法の第 II 相試験	第 II 相、非盲検、単用量<br>救済手術前に DC/Gal $1x10^8$ /body を鼻粘膜下に単回投与後、1 週後に、NKT 細胞 $5x10^7$ /body を腫瘍栄養動脈内に単回投与	10 例	安全性<br>免疫応答性<br>抗腫瘍効果	重篤な有害事象は認められなかった。DC/Gal 投与 3 週後の CT 評価において、5/10 例で部分奏効が認められた。7/10 例において、腫瘍浸潤 NKT 細胞数の増加が認められた。<br>[参考資料 xvi.]
切除不能進行期ならびに再発非小細胞肺癌に対する α-Galactosylceramide パルス樹状細胞を用いた免疫細胞治療<br>―第 II 相試験―	非盲検、単用量<br>DC/Gal $1x10^9$ /m^2/週で 2 回静脈内投与後、4 週間休薬し、再度週 1 回で 2 回、計 4 回投与	35 例	OS<br>安全性<br>免疫応答性	最大の解析集団における生存期間中央値(MST)は 667 日 (95%信頼区間(CI) 451~790 日）であった。期待 MST 17 カ月、閾値 MST 8 カ月と設定して試験実施し、95%CI 下限が閾値 MST を含まず、目標達成と考えられた。無再発生存期間は 85 日 (95% CI 80～113 日）、奏効率・病勢制御率は 2.9% (95% CI 0.1 ~14.9%)、42.9% (95% CI 26.3~60.7%)であった。また細胞投与に関連する重篤

				な有害事象は認められなかった。[参考資料 xvii.]
標準治療後の進行期頭頸部扁平上皮癌症例に対するα-Galactosylceramide パルス樹状細胞を用いたアジュバント療法の有効性に関する２群間ランダム化第 II 相臨床試験	二重盲検/ DC/Gal もしくはコントロールとしての非処理 DC を 1x10^8 / body/週、計 2 回鼻粘膜下投与	23 人（予定症例数 66 人）	無再発生存期間<br>安全性<br>2 年無再発生存率<br>2 年全生存率<br>免疫応答性	無再発生存期間中央値について、実薬群(n=13)未達（95%CI 0.85-NE）、非処理群（n=9) 2.60 年（95%CI 0.35-NE）で p=0.3613 (Log-rank test) であった。2 年無再発生存率について、実薬群 69％ (95%CI 48-99%)、非処理群 56％ (95%CI 31-100%) (p=0.3676)、2 年全生存率について、実薬群 82% (95%CI 62-100%)、非処理群 71％ (95% CI 45-100%) (p=0.443) と、いずれにおいても両群間に統計学的に有意な有効性は示されなかった。<br>本細胞治療に関連すると判断された重篤な有害事象は認められず、関連が否定されない重篤な有害事象が１件（Grade5）認められた。当該事例について、効果安全性評価委員会において、臨床所見、剖検所見、免疫学的所見も含めて検討された結果、DC/Gal の鼻粘膜下投与との因果関係は支持しないが、本試験自体には相応の侵襲が存在するため、本試験との因果関係は否定できないという結論となった。

非小細胞肺癌に対して DC/Gal を投与した結果、標準的な治療法と比較して有意な生存期間延長が認められた。さらに投与した 17 例を、細胞投与後に IFN-γ産生能の増強を認めた免疫療法反応群（n＝10 例、赤線）と非反応群（n＝7 例、黒線）に群分けした時の細胞治療後の各群の生存曲線を比較した結果、IFN-γ産生能の増強を認めた免疫療法反応群の生存期間延長効果は顕著であった[参考資料 xv.]。

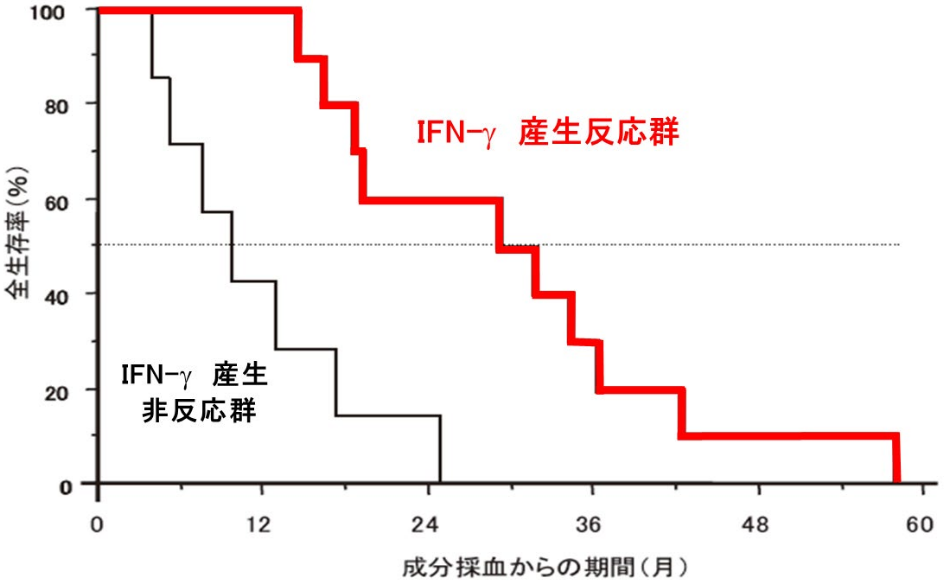

図 11　生存率と IFN-γ産生反応

頭頸部がんに対する DC/Gal 及び活性化 NKT 細胞（患者末梢血由来の細胞 5×10^7 cells/body）を用いた第II相臨床試験の結果、10 例中 5 例に奏効が認められた。末梢血中の抗腫瘍活性の誘導と抗腫瘍効果には関連は認められなかった一方で、腫瘍組織内に NKT 細胞が多く存在する患者において、より強い抗腫瘍

効果が認められる傾向があった[参考資料 xvi.]。

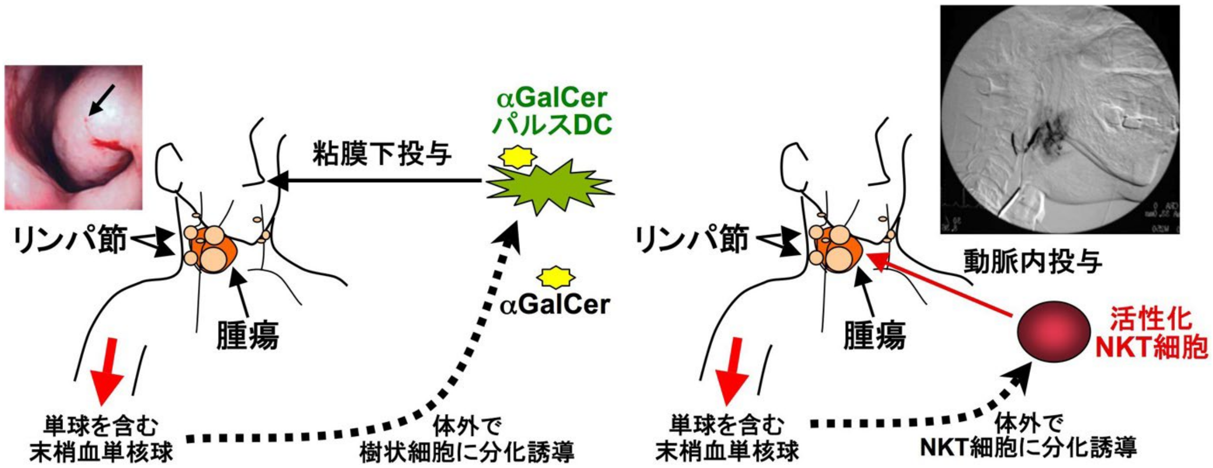

図 12　頭頸部がんに対して行った NKT 細胞療法の概要

1.2.7. 予想される副作用

DC/Gal 又は iPS-NKT 細胞に関係して予想される主たる「副作用」及び処置例を以下に示す。

① 血圧上昇：症状及び重症度に応じて降圧剤を投与する。

② 発熱：血液検査、X 線検査、血液培養を施行し、さらに感染症の可能性も考慮し、速やかな抗菌薬（原因菌に対してスペクトルを有するもの）の投与を行う。解熱剤については、熱苦痛がなければ投与は控えるが、熱苦痛が著しいときには適宜投与する。

③ 呼吸障害：速やかに酸素を投与して、酸素化の安定を図る。原因検索の一環として血液検査、X 線検査及び CT 検査（可能であれば造影）を行う。輸注中の重症呼吸障害の原因としては、アレルギー性のものと塞栓症（特に iPS-NKT の投与時）によるものとが考えられる。それぞれに対して、重症度に応じたステロイドやエピネフリンの投与、並びに塞栓症に対する抗血栓療法を行う。

④ ショック：担当医及び主治医が速やかに対応する。バイタルサインの確認、血液検査、重症度に応じたステロイドやエピネフリンの投与を行う。呼吸不全や血圧低下に対しては重症度に応じて酸素投与や昇圧剤の投与を行う。呼吸不全や血圧低下に対しては重症度に応じて酸素投与や昇圧剤の投与を行う。

⑤ 咽頭皮膚ろう：自家 NKT 細胞と DC/Gal との併用療法に関する先行研究において腫瘍縮小に伴う咽頭皮膚ろうが認められていたことから、本試験においても本有害事象に留意する必要がある。本有害事象が認められた際には、感染予防及び必要に応じて咽頭皮膚ろう閉鎖術を行う。

⑥ 脳梗塞：細胞を頸部の動脈に投与することから、カニュレーションの際に血栓が脳へ飛ぶ可能性がある。機能障害の有無などを脳神経外科に相談したうえで評価し、バイタルサインの確認や脳保護薬の投与などを行う。

⑦ 穿刺部の出血や血種形成：カニューレの動脈穿刺を行うことで発生する可能性がある。穿刺後に圧迫止血を行うが、重度の血種が生じた場合には形成外科に相談し除去術などを考慮する。軽度から中等度であれば経過観察で吸収される。

1.3. 本試験実施が妥当であると判断した理由

1.3.1. 初回投与量及び用法の妥当性

ヒト iPS-NKT 細胞の 1×10^6 及び 2×10^6 cells/body（20g）を NKT-deficient B6 マウスに Day0, 14, 28 の 3 回静脈内投与し、同日にマウス DC/Gal 1×10^9 cells/m^2 を静脈内投与した結果、いずれの群にも死亡及びその他の安全性上懸念すべき所見が認められず、マウス DC/Gal 併用時のヒト iPS-NKT 細胞の最大無毒性用量は 2×10^6 cells/body＝1×10^8 cells/kg であった。当該用量はヒトの体表面積当たりの投与量に換算すると、1×10^8 cells/kg×3≒3×10^8 cells/m^2 であることから（FDA“Guidance for Industry Estimating the Maximum Safe Starting Dose in Initial Clinical Trials for Therapeutics in Adult Healthy Volunteers”）、当該用量の 1/10 となる 3×10^7 cells/m^2 を本試験における開始用量として設定可能と考えられる。また、本試験の計画時点（2022 年 4 月）で実施中の iPS-NKT 細胞単独投与の忍容性を確認することを目的とした第 I 相試験（以下、「FIH 治験」）が実施中であり、iPS-NKT 細胞 3×10^7 cells/m^2 の忍容性が確認されている。したが

って、本試験における iPS-NKT 細胞の開始用量として、3×10^7 cells/m^2 を設定した。また、自家 DC/Gal の投与量は、先行研究において自家 NKT 細胞との併用の忍容性、安全性等が確認されている用量である 1×10^8 cells とした。

用法について、過去の自家 DC/Gal と自家 NKT 細胞の併用試験において有効性が認められたプロトコルでは、自家 DC/Gal 1 回もしくは２回投与後に自家 NKT 細胞を１回投与していることから、本試験における iPS-NKT 細胞の投与回数を 1 回と設定した。加えて、非臨床試験において実施された静脈内投与と比較して、腫瘍栄養動脈内投与では腫瘍局所への集積性が高く、より高い有効性が期待されることから、本試験における用法を腫瘍栄養動脈内投与と設定した。

1.3.2. 対象集団の妥当性

本試験は FIH 試験であることから、「抗悪性腫瘍薬の臨床評価方法に関するガイドライン」（平成 17 年 11 月 1 日付け薬食審査発第 1101001 号）に基づき、標準的治療に不応又は不耐の再発又は進行頭頸部がん患者とした。また、iPS-NKT 細胞の投与経路を腫瘍栄養動脈内と規定することから、動注投与可能かつ評価可能な病変を有する患者を選択基準として設定した。

1.3.3. 評価方法の妥当性

本試験は FIH 試験であることから、単一用量における 3+3 デザインを用いて iPS-NKT 細胞と DC/Gal を併用することの忍容性を評価することとした。すなわち、本試験で設定された用量を 3 例に順次投与し DLT の発現が認められない場合、及び DLT 発現例数が 1 例の場合は同用量を追加の 3 例に投与し、合計 6 例において DLT 発現例数が 1 例以下であっても当該用量を MTD とする。DLT 発現例数が 6 例中 2 例以上の場合は試験を中止する。

また、2 例以上の被験者に対する特定細胞加工物の同日投与は行わず、少なくとも 7 日以上の間隔をあけて次の被験者における自家 DC/Gal 及び iPS-NKT 細胞投与を開始する。

上記手順により、DLT の評価を行い、データモニタリング委員会において、DLT を含むすべての有害事象等の安全性情報を評価した上で、試験継続の可否及び MTD を決定する。

DLT 評価期間について、iPS-NKT 細胞を用いた非臨床薬物動態試験において、静脈内への単回投与後 48 時間でマウス血中の iPS-NKT 細胞濃度は投与量の 0.33%まで低下し、以降は検出されなかったことから、当該体内消失時間より十分長い投与間隔として、本試験における DLT 評価期間を iPS-NKT 細胞投与後 2 週間までと設定した。

1.3.4. 被験者安全性の確保

試験期間を通じて体重、バイタルサイン、ECOG PS、腫瘍評価、血液学的検査、血液生化学検査、凝固検査、尿検査、放射線画像検査等を実施する。加えて、DLT 評価期間（自家 DC/Gal 投与から iPS-NKT 細胞投与後 14 日目まで）及び観察期間の 1 日目は入院を原則とし、被験者の安全性を慎重にモニタリングする。また、iPS-NKT 細胞投与第 7 日目以降の検査結果及び診察において、Grade 2 以上の非血液学的毒

性及び Grade 3 以上の血液学的毒性が発現しておらず、有害事象発生時に実施医療機関及び自宅近隣医療施設へ連絡ができ、緊急時に速やかに患者と連絡が取れる体制が整っている場合にのみ、実施責任者又は分担医師の判断で外泊又は退院することも可能とする。

以上より、本試験を実施することは、倫理的、科学的に妥当であると判断した。

2. 試験の目的と必要性

iPS-NKT 細胞と自家 DC/Gal との併用療法は、非臨床試験における腫瘍増殖抑制作用及びマウスに対する一定の安全性について確認されているものの、現在までにヒトへの投与経験はない。したがって、ヒトに対する iPS-NKT 細胞と自家 DC/Gal との併用療法の忍容性、安全性及び今後の開発に向けた有効性並びに試験治療により惹起される免疫学的動態の探索が必要である。本試験は、主に投与用量における DLT 発現割合を算出することにより併用療法の忍容性を確認し、安全性プロファイル、有効性評価を副次的に確認することに加え、免疫学的動態について探索的に評価することを目的に実施する。

3. 対象患者

以下の選択基準のすべてを満たし、除外基準のいずれにも該当しない患者を適格例とする。

3.1. 選択基準

以下のすべての条件に該当する患者を対象とする。

1) 標準的治療に不応又は不耐の再発又は進行頭頸部がん患者のうち、腫瘍栄養動注投与可能かつ評価可能な病変を有する患者。
2) 前治療から試験治療開始までに 1 カ月以上が経過する見込みのある患者。前治療の種類は問わない。
3) 同意取得時の年齢が 20 歳以上 80 歳未満の患者。
4) ECOG の Performance Status（別紙 2 参照）が 2 以下の患者。
5) 以下の検査データを満たす患者
    - ヘモグロビン≧10 g/dL
    - 白血球数≧3,000/μL、血小板数≧75,000/μL
    - 血清クレアチニン≦1.5 mg/dL
    - 総ビリルビン≦1.5 mg/dL、AST （GOT）、ALT （GPT）≦施設基準値上限の 2.5 倍
    - SpO2 （room air） ≧93%
6) 3 カ月以上の予後が期待される患者。
7) 本人からの文書による同意が得られている患者。

【設定の根拠】

1）：本試験において、iPS-NKT 細胞を腫瘍栄養動脈内投与可能ながん種として頭頸部がんを選択した。また、本試験が first in human 試験であることを鑑み、「抗悪性腫瘍薬の臨床評価方法に関するガイドライン」（平成 17 年 11 月 1 日付け薬食審査発第 1101001 号）に基づき、標準的治療のない患者を本試験の対象として設定した。

2-5）：対象患者の安全性及び倫理性を考慮し、信頼性のあるデータを得るために設定した。

6）：試験実施期間を考慮し、試験治療の忍容性及び安全性を評価するために最低限必要と考えられる期間として設定した。

3.2. 除外基準

以下のいずれかの条件に該当する患者は対象としない。

1) HBs 抗原、HCV 抗体[1)]、HIV 抗体又は HTLV-1 抗体が陽性、若しくは HBs 抗原陰性であるが HBV-DNA 定量検査で HBV-DNA が検出された患者。
2) 試験治療開始前の少なくとも 2 週間以内にコルチコステロイド（メチルプレドニゾロン 10 mg/日以上又は相当量）又は免疫抑制剤を内服又は注射している患者。
3) 妊娠、授乳中（授乳を中止する場合も含む）又は本試験中に妊娠を予定している女性患者。また妊娠可能な女性[2)] 及び男性で、DC/Gal 又は iPS-NKT 細胞投与期中及び iPS-NKT 細胞最終投与後 14 日間まで医師の指導のもと、異性間の性交を禁止することに同意しない者（ただし本試験の同意を得る前に、本人又はパートナーの両側卵管閉塞術又は精管切除術を既に実施していた場合、異性間の性交禁止は該当しない）。
4) コルチコステロイドや生物学的製剤の全身投与もしくは免疫抑制療法を要する活動性の自己免疫疾患を有する患者。
5) 免疫チェックポイント阻害薬で免疫関連有害事象を生じた患者。
6) コントロール不良な糖尿病を有する患者。
7) 重症以上の肺疾患を有する患者（mMRC 息切れスケール Grade 2 以上（別紙 3 参照））、又はステロイドによる治療を要する非感染性の間質性肺疾患の既往を有する患者。
8) 重大な心疾患を有する患者（NYHA class III 以上（別紙 4 参照））。
9) 試験治療の初回投与前 2 年以内に進行中または積極的な治療を必要とする別の悪性腫瘍があることが分かっている。ただし、根治治療が実施された皮膚の基底細胞がん、皮膚の扁平上皮癌、根治的切除された非浸潤性乳管がん及び根治的切除された非浸潤性乳がん及び内視鏡的に根治切除された上部消化管癌を除く。
10) 造影剤を使用できない患者。
11) ヒト血清アルブミン製剤、又は異種由来タンパク質に対し過敏症の既往のある患者。
12) 同意取得時、他の試験又は臨床試験に参加し、他の試験製品等の投与を受けている又は当該試験製品等による有害事象の影響が残存していることが、実施責任者又は分担医師により判断される患者。
13) HLA-A、B 及び C の genotype が iPS-NKT 細胞と完全に一致する患者。[3)]
14) 成分採血禁忌（不安定狭心症、A-V block II 度以上、WPW 症候群、完全左脚ブロック、収縮期血圧 90 以下もしくは 170torr 以上）の患者。
15) 担当医が本試験への参加を不適当と判断した患者。

---

1) HCV 抗体が陽性の場合、HCV-RNA 定量検査を実施し陰性であれば組入れ可とする。

2) 妊娠の可能性のある女性とは、初潮を経験しており、不妊手術（子宮摘出術、両側卵管結紮又は両側卵巣摘出術など）を受けておらず、閉経していないすべての女性が含まれる。閉経の定義は、他の医学的理由なく、12 カ月以上連続して無月経であることとする。

3) iPS-NKT 細胞の遺伝子型は以下の通り。
A*26:01-B*35:01-C*03:03
A*26:03-B*35:01-C*03:03

【設定の根拠】

1）：iPS-NKT 細胞が静脈内投与された際に、肝臓への集積が認められている。本試験において、iPS-NKT 細胞は腫瘍栄養動脈内へ投与され、全身への循環は少ないと予測されているものの、最終的に肝臓への影響も否定されないことから、対象患者の安全を考慮し、設定した。

2, 4-5）：iPS-NKT 細胞は、対象患者における直接的及び間接的な免疫活性化を意図した製剤であり、対象患者の免疫状態に影響を及ぼす疾患及び薬剤の影響を除外するために設定した。

3）：iPS-NKT 細胞の胎児及び妊孕性への影響は現在までに確認されていないことから、対象患者への当該影響を避けるため設定した。なお、体内に投与された iPS-NKT 細胞は投与後 7 日目でほぼ体内から消失すると考えられることから、避妊期間を設定した。

6-8）：対象患者の安全性を考慮し、全身状態が芳しくない状態にある患者を除外するために設定した。

9）：頭頸部以外のがんによる、有害事象の影響を除外するために設定した。特に頭頸部がんの重複癌として頻度の高い消化器癌における根治治療後の再発率を参考に、2 年以内の積極的治療症例は再発リスクが一定程度懸念されると考え、当該期間を設定した。

10）：本試験において、対象患者の腫瘍栄養動脈を確認するために造影剤の使用は必須であることから、造影剤を使用できない患者を除外するために設定した。

11）：被験者の安全を確保するために設定した。

12）：他の試験製品等の投与による影響を除外するために設定した。

13）：当該 HLA genotype を有する患者では、患者自身の NKT 細胞と iPS-NKT細胞との弁別が不可能となるため。

14）：DC/Gal 調製のための成分採血ができない患者では、本研究の実施が不可能であるため。

15）：その他、安全に試験を実施できないことが想定されると担当医が判断した場合に除外可能となるように設定した。

4. 被験者の同意

4.1. 同意文書及びその他の説明文書の作成並びに改訂

実施責任者は、被験者から試験参加の同意を得るために用いる同意文書及びその他の説明文書を可能な限り平易な表現で作成する。また、同意文書及びその他の説明文書を改訂する必要があると認めた場合は、これらを改訂する。

実施責任者は、作成又は改訂された同意文書及びその他の説明文書を特定認定再生医療等委員会及び生命倫理審査委員会に提出し、その承認を得る。

4.2. 同意取得の時期と方法

1) 同意の取得

実施責任者又は分担医師は、特定認定再生医療等委員会及び生命倫理審査委員会の承認を得た同意文書及びその他の説明文書を被験者に手渡し、「4.3 被験者に対する説明事項」に示す内容につい

て十分な説明を行う。また、必要な場合には、試験協力者も被験者に補足的な説明を行う。被験者が試験の内容を良く理解したことを確認した上で、試験開始前（スクリーニング）検査を実施するまでに文書で自由意思による同意を取得する。

2) 説明時の被験者への対応

実施責任者又は分担医師は、同意を得る前に被験者が質問をする機会と当該試験に参加するか否かを判断するのに十分な時間を与え、被験者の質問に対しては、被験者が満足するように回答する。

3) 同意書への記入方法及び説明文書の交付

被験者の同意に際しては、説明を行った実施責任者又は分担医師が記名押印又は署名し、説明した日付を記入する。被験者は同意書に記名押印又は署名し、同意した日付を記入する。なお、試験協力者が補足的な説明を行った場合は、当該試験協力者も記名押印又は署名し、説明した日付を記入する。同意を得た後、説明文書及び同意書の写しを被験者に交付する。

4) 説明文書改訂時

実施責任者又は分担医師は、被験者の同意に関連し得る新たな情報の入手などにより同意文書及びその他の説明文書を改訂した場合、その都度、被験者に対して改訂された同意文書及びその他の説明文書を用いて改めて説明し、試験への参加継続について被験者から文書で自由意思による再同意を取得する。なお、被験者の同意に影響を与えうる新たな重要な情報が得られた場合には、直ちに当該情報を被験者に提供し、これを文書により記録するとともに、被験者が試験に継続して参加するかどうかを確認する。

4.3. 被験者に対する説明事項

実施責任者が作成する説明文書には、以下の事項を記載する。

1) 提供する再生医療等の名称及び厚生労働大臣に再生医療等提供計画を提出している旨
2) 再生医療等を提供する医療機関の名称並びに当該医療機関の管理者、実施責任者及び再生医療等を行う医師又は歯科医師の氏名（再生医療等を多施設共同研究として行う場合にあっては、代表管理者の氏名及び当該再生医療等を行う他の医療機関の名称及び当該医療機関の管理者の氏名を含む。）
3) 提供される再生医療等の目的及び内容
4) 当該再生医療等に用いる細胞に関する情報
5) 再生医療等を受ける者として選定された理由（研究として再生医療等を行う場合に限る。）
6) 当該再生医療等の提供により予期される利益及び不利益
7) 再生医療等を受けることを拒否することは任意であること。
8) 同意の撤回に関する事項
9) 再生医療等を受けることを拒否すること又は同意を撤回することにより不利益な取扱いを受けないこと。
10) 研究に関する情報公開の方法（研究として再生医療等を行う場合に限る。）

11) 再生医療等を受ける者又は代諾者の求めに応じて、研究計画書その他の研究の実施に関する資料を入手又は閲覧できる旨及びその入手又は閲覧の方法（研究として再生医療等を行う場合に限る。）
12) 再生医療等を受ける者の個人情報の保護に関する事項
13) 試料等の保管及び廃棄の方法
14) 研究に対する第八条の八第一項各号に規定する関与に関する状況（研究として再生医療等を行う場合に限る。）
15) 苦情及び問合せへの対応に関する体制
16) 当該再生医療等の提供に係る費用に関する事項
17) 他の治療法の有無及び内容並びに他の治療法により予期される利益及び不利益との比較
18) 当該再生医療等の提供による健康被害に対する補償に関する事項（研究として再生医療等を行う場合に限る。）
19) 再生医療等を受ける者の健康、子孫に受け継がれ得る遺伝的特徴等に関する重要な知見が得られる可能性がある場合には、当該者に係るその知見（偶発的所見を含む。）の取扱い
20) 再生医療等を受ける者から取得された試料等について、当該者から同意を受ける時点では特定されない将来の研究のために用いられる可能性又は他の医療機関に提供する可能性がある場合には、その旨と同意を受ける時点において想定される内容
21) 当該再生医療等の審査等業務を行う認定再生医療等委員会における審査事項その他当該再生医療等に係る認定再生医療等委員会に関する事項
22) 研究に用いる医薬品等の製造販売をし、若しくはしようとする医薬品等製造販売業者又はその特殊関係者による研究資金等の提供を受けて研究を行う場合においては臨床研究法第三十二条に規定する契約の内容（研究として再生医療等を行う場合に限る。）
23) その他当該再生医療等の提供に関し必要な事項

5. 試験の方法

5.1. 試験のデザイン

本試験は、標準的治療に不応又は不耐の再発又は進行頭頸部がん患者を対象とし、iPS-NKT 細胞と自家 DC/Gal との併用療法の忍容性、安全性、薬物動態、薬力学及び抗腫瘍効果を検討する非盲検非対照第 I 相試験である。

用法・用量は、自家 DC/Gal 1×10^8 cells 単回投与の 5 日後に iPS-NKT 細胞 3×10^7 cells/m^2 を単回腫瘍動脈内投与とし、当該用量において 2 例の DLT（「5.2. DLT」参照）が出現した場合、試験中止とする。2 例以上の被験者に対する DC/Gal 又は iPS-NKT 細胞の同日投与は行わず、少なくとも 7 日以上の間隔をあけて次の被験者における本試験治療を開始する。

上記の用量で 3+3 デザインによる忍容性の評価を行う。すなわち、設定された用量を投与された最初の 3 例において DLT の発現が認められない場合、及び 1 例 DLT 発現が認められた場合は同用量を追加の 3 例に投与し、DLT 発現例数が 6 例中 2 例以上の場合は試験を中止する。最終的に、合計 6 例において DLT 発現例数が 1 例以下であれば当該用量を MTD とする。

本試験について説明及び同意が得られた時点からスクリーニング期が開始される。スクリーニング検査結果を踏まえ、本試験への参加が適格と判断された場合に症例登録が実施される。試験治療開始 1 週間前に被験者の末梢血を採取の上、自家 DC/Gal の製造を行う（細胞準備期）。

自家 DC/Gal 投与から iPS-NKT 細胞投与後 14 日目までを DLT 評価期間とし、原則入院下で被験者の安全性を慎重にモニタリングする。ただし、iPS-NKT 細胞投与第 7 日目以降の検査結果及び診察において、Grade 2 以上の非血液学的毒性及び Grade 3 以上の血液学的毒性が発現しておらず、有害事象発生時に実施医療機関及び自宅近隣医療施設へ連絡ができ、緊急時に速やかに患者と連絡が取れる体制が整っている場合には実施責任者又は分担医師の判断で外泊又は退院することも可能とする。

実施責任者及び試験調整医師が指名するもので構成されるデータモニタリング委員会は、本試験の安全性をモニタリングし、試験継続について勧告を行うことができる。

iPS-NKT 細胞と自家 DC/Gal の併用療法の有効性を予備的に確認するため、試験治療開始前（4 週間以内）及び観察期初日（iPS-NKT 細胞の最終投与より 14 日後又は投与中止が決定された日のうち、より遅い時点）に造影 CT による腫瘍評価を行う。

有害事象は同意取得後から観察期最終日（中止時）まで観察する。有害事象の観察期間終了時に、DC/Gal 又は iPS-NKT 細胞との因果関係が否定できない有害事象が未回復の場合、当該有害事象の回復、安定又は死亡のいずれか最も早い時点まで追跡調査を行う。

試験のアウトラインを以下に示す。

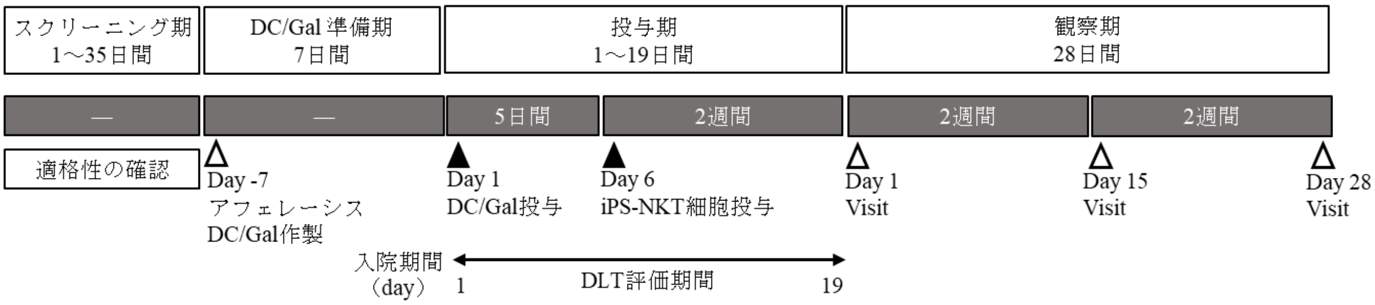

図 13　本試験のアウトライン

5.2.　用量制限毒性（DLT）

自家 DC/Gal 投与から iPS-NKT 細胞投与後 14 日目までの期間（DLT 評価期間）に観察された、DC/Gal 又は iPS-NKT 細胞との因果関係が否定できない以下の有害事象を DLT とする。Grade 判定は CTCAE ver.5.0 日本語訳 JCOG 版に従う。

- Grade 4 以上の血液毒性
- 原疾患と関連のないあらゆる輸血及び G-CSF 製剤投与を要する血液毒性
- Grade 3 以上の非血液毒性（一過性の臨床検査値異常、適切な処置により Grade 2 以下へ回復した下痢、悪心、嘔吐又はその他の管理可能な全身症状は除く）

その他、DC/Gal 又は iPS-NKT 細胞との因果関係が否定できない有害事象により、以下の状況とな

った場合、当該有害事象を DLT とする。

・　輸血療法を行った場合

実施責任者は DLT の定義に該当すると疑われる疾患、徴候が発生した際には、速やかにデータモニタリング委員会に判断の妥当性の審議を依頼し、当該患者における試験継続の可否を決定する。

DLT 以外の理由により DLT 評価期間を完了しなかった被験者は、DLT 評価可能例とみなさず、補充を行い DLT を評価する。

5.3. 目標被験者数と試験実施期間

目標被験者数：2～6 例

試験実施期間：2022 年 9 月～2024 年 8 月 31 日

症例登録期間：2022 年 10 月～2024 年 7 月 14 日

5.4. 施設登録及び症例登録

施設登録及び症例登録は、千葉大学医学部附属病院　データセンターにおける中央登録制とする。

5.4.1. 施設登録

1) 実施責任者は、当該施設の特定認定再生医療等委員会で承認が得られた後、特定認定再生医療等委員会の承認通知書の写し及び施設登録依頼書を千葉大学医学部附属病院データセンターに FAX する。
2) 千葉大学医学部附属病院データセンターは施設登録を行い、施設登録完了通知書を実施責任者に送付する。

5.4.2. 症例登録

1） 文書によって同意を取得した後、スクリーニング検査を行う。
2） 実施責任者又は分担医師は、スクリーニング検査の結果、被験者が選択基準を満たし、除外基準に抵触していないことを確認する。
3） 実施責任者又は分担医師は適格性に係る判断の妥当性について試験調整委員会を構成する 2 名以上の医師に諮問する。最終的に「適格」と判断された被験者について、実施責任者、分担医師又は試験協力者は症例登録を行う。
4） 実施責任者、分担医師又は試験協力者は症例登録に必要な情報を症例登録票に記入し千葉大学医学部附属病院データセンターに FAX する。なお、実施責任者又は分担医師は適格性判定を確認し、適格と判定された上で試験治療を開始する。一度登録された被験者の登録取り消しはされない。誤登録・重複登録が判明した際には速やかに千葉大学医学部附属病院データセンターに連絡する。

なお、DC/Gal 又は iPS-NKT 細胞の品質を含む被験者の医学的問題以外の要因（例：DC/Gal 又は iPS-

NKT 細胞の細胞数が投与予定用量に満たなかった、等）又は一時的な感染症等に伴う発熱（インフルエンザ感染など）により規定期間内に初回投与が出来ない状況において、再度必要なスクリーニング検査を実施した上で、上記 3)、4）の手順に従い、改めて登録を可能とする。

5.4.3. 症例登録先

千葉大学医学部附属病院データセンター
TEL：043-226-2667　FAX：043-226-2644

5.5. 登録されなかった被験者の取り扱い

登録において、不適格などの何らかの理由で登録が行われなかった被験者は、試験の登録症例には含めない。実施責任者又は分担医師は、当該被験者に本試験への登録が不可である旨を説明する。

5.6. 投与スケジュール及び投与量・投与方法

すべての組入れ基準を満たし、いずれの除外基準にも該当しない被験者から自家 DC/Gal を作製し投与スケジュールに則って自家 DC/Gal 及び iPS-NKT 細胞を投与する。

本試験は自家 DC/Gal 及び iPS-NKT 細胞の併用療法に関する FIH 試験であり、単用量における 3+3 デザインとする。自家 DC/Gal 投与 5 日後に iPS-NKT 細胞を投与する。投与細胞数は、自家 DC/Gal 1×10^8 cells/回、iPS-NKT 3×10^7 cells/m^2/回とする。投与回数は、自家 DC/Gal、iPS-NKT 細胞共に 1 回とする。2 例以上の被験者に対する特定細胞加工物の同日投与は行わず、少なくとも 7 日以上の間隔をあけて次の被験者における DC/Gal 又は iPS-NKT 細胞の投与を開始する。自家 DC/Gal は微量注射シリンジを用いた鼻粘膜下投与、iPS-NKT 細胞は腫瘍栄養動脈への投与とし、血管造影下で挿入されたカテーテル（セルジンガー法）を介して行う。

5.7. 投与開始基準

本試験に登録された患者に対して、DC/Gal 又は iPS-NKT 細胞投与日に、「投与中止基準」のいずれにも該当しないことを確認した上で、自家 DC/Gal の投与を開始する。

5.8. 投与延期基準

以下の基準に該当した場合、実施責任者又は分担医師は iPS-NKT 細胞投与を最長 3 日まで延期できる。

1) 実施責任者又は分担医師が DC/Gal 又は iPS-NKT 細胞の投与を不適当と認めた場合。
2) DC/Gal 又は iPS-NKT 細胞の投与後に発現したすべての有害事象が管理可能な Grade 2 若しくは Grade 1 以下又はベースラインまで回復しない場合。
3) DC/Gal 又は iPS-NKT 細胞投与前 24 時間以内に、38℃以上の発熱が認められた場合を含む治療を要

する感染症が発現した場合

5.9. 個々の症例における投与中止基準及び試験中止基準

5.9.1. 個々の症例における投与中止基準

以下のいずれかの基準に該当した場合、実施責任者又は分担医師は DC/Gal 又は iPS-NKT 細胞の投与を中止する。投与中止となった場合は、投与中止が決定された日から観察期に移行し、検査スケジュールに沿った検査・評価を継続して実施する。

1) DC/Gal 投与後に DLT が出現した場合、又は試験期間中に発生した Grade 3 以上のすべての有害事象が、DC/Gal 又は iPS-NKT 細胞投与直前までに回復若しくは Grade 1 に改善しない、又は臨床的に十分管理可能な Grade 2 に改善しない場合。
2) 治療を要する感染症が発現し、DC/Gal 又は iPS-NKT 細胞投与直前まで回復若しくは Grade 1 に改善しない、又は臨床的に十分管理可能な Grade 2 に改善しない場合。
3) SpO_2 （room air） が 90%未満で維持されている。
4) DC/Gal 又は iPS-NKT 細胞投与前の検査において mMRC 息切れスケール Grade 2 以上（別紙 3 参照）。
5) 被験者からの中止の申し出があった場合。
6) その他、実施責任者又は分担医師により被験者への DC/Gal 又は iPS-NKT 細胞の投与中止の必要性が認められた場合。

実施責任者又は分担医師は中止日、中止理由等の必要事項を記録する。

なお、DC/Gal 又は iPS-NKT 細胞の投与が中止された場合の「中止日」は、中止の理由となる事象が発現した日又は判明した日ではなく、実施責任者又は分担医師が中止を判断した日とする。

5.9.2. 個々の症例における試験中止基準

以下のいずれかの基準に該当した場合、実施責任者又は分担医師は当該症例における試験を中止する。

1) 実施責任者又は分担医師の判断により、併用禁止薬又は併用療法を必要とする原疾患の進行が認められた場合。
2) 被験者からの中止の申し出があった場合。
3) 症例登録後、被験者が対象として不適格であることが判明した場合。
4) その他、実施責任者又は分担医師が被験者の DC/Gal の投与が不可能と判断した場合。

実施責任者又は分担医師は、DC/Gal 又は iPS-NKT 細胞投与開始後に中止基準に該当する被験者が判明した場合には、当該被験者にその旨を説明して試験を中止し、適切な処置を行う。

有害事象（臨床検査値の異常変動を含む）により中止した場合、退院又は転院が可能と判断できるまで、必要な検査・観察を実施し、DC/Gal 又は iPS-NKT 細胞との因果関係が否定出来ない有害事象について

は、原則として症状が回復又は軽快するまで可能な限り観察継続する。

実施責任者又は分担医師は中止日、中止理由等の必要事項を記録する。また、中止時の規定項目を可能な範囲で行う。

なお、個々の症例における試験が中止された場合の「中止日」は、中止の理由となる事象が発現した日又は判明した日ではなく、実施責任者又は分担医師が中止を判断した日とする。

5.10. 併用薬及び併用療法

5.10.1. 併用可能薬・併用可能療法

- 原疾患に対する緩和療法及び支持療法
- 以下の場合、試験中、局所疼痛管理のための緩和的放射線療法を実施することができる。
  - 照射範囲が骨髄全体の 10%以下である場合
  - 照射野に評価病変が含まれない場合。
- DC/Gal 又は iPS-NKT 細胞に起因すると考えられる有害事象への対応としてのあらゆる輸血療法
  当該有害事象については DLT とする。

5.10.2. 併用薬・併用療法の記録

DC/Gal 又は iPS-NKT 細胞の投与後から最終観察終了日（中止時）までに使用した 5.10.3.に記載する薬剤及び併用療法について、薬剤名（商品名若しくは一般名）、投与期間、投与目的を記録する。併用療法については、療法名、治療期間、治療目的を記録する。

5.10.3. 併用禁止薬及び併用禁止療法

本試験において、DC/Gal 又は iPS-NKT 細胞は単独投与とし下記の薬剤・療法の併用は認めない。臨床上使用する必要がある場合には、緊急の危険を回避するための逸脱として取扱い、これを記録及び医療機関の長に報告する。

- コルチコステロイド（メチルプレドニゾロン 10 mg/日以上又は相当量）又は免疫抑制剤の内服又は注射、他の抗悪性腫瘍剤、放射線治療又は腫瘍切除を含む手術。
- あらゆる輸血療法（ただし、5.10.1 に記載されている場合を除く）。

5.11. 試験中止又は終了後の対応

試験を中止又は終了した被験者には、退院又は転院が可能と医学的に判断できるまで、必要な検査・観察を実施し、必要に応じて適切な処置を講じる。

6. 特定細胞加工物

6.1.1. iPS-NKT 細胞の包装・表示

iPS-NKT 細胞を封入したガス交換可能なプラスチック試験管は、CO_2 の供給が可能なプラスチック製パック（アネロパック®CO_2）に収納され、さらに輸送時の保温のために 37℃に保温可能な輸送箱（TACPack®37）で外装される。また、iPS-NKT 細胞を封入したプラスチック製パックには、下記を明記している。

1） 試験用である旨
2） 自ら試験を実施する者の氏名及び職名並びに住所
3） 構成細胞、導入遺伝子又は識別記号
4） 製造番号又は製造記号
5） 貯蔵方法、有効期間（定める必要があるもの）

iPS-NKT 細胞ラベル（見本）

**試験用　iPS-NKT**
製造番号：NKT-　　　　　（融解日）
包装形態・本数：50 mL チューブ　　　本
構成細胞：ヒト iPS 細胞由来 NKT 細胞
有効期限：出荷から 6 時間かつ調剤後 3 時間以内
出荷日時：20　　年　　　月　　　日　　　　時　　分
保存条件：5 % CO_2 供給下　37℃（許容範囲 33℃～38℃）
実施責任者　飯沼　智久・助教
千葉大学医学部附属病院　〒260-8677 千葉県千葉市中央区亥鼻１－８－１

6.1.2. DC/Gal の包装・表示

DC/Gal を封入した微量注射シリンジは袋に入れられ、さらに輸送時の温度管理のために室温（4~25℃）に維持可能な輸送箱（TACPack®0208Re-2A-SC）で外装される。また、DC/Gal を封入した袋には、下記を明記している。

1） 製造番号　:　A2023xxx-000x-DC
2） 製造日時　:　YYYY 年 MM 月 DD 日　　時　　分
3） 投与可能期間　:　出荷より２時間
4） 試験用である旨

ラベル見本

試験用　A2023xxx-000x-DC<br>包装形態・本数：微量注射シリンジ　1 本<br>出荷日時：YYYY 年 MM 月 DD 日　　時　　分<br>投与可能期間：出荷より 2 時間

6.2. 管理・調剤方法

6.2.1. iPS-NKT の管理・調剤方法

1) 実施責任者は、症例登録後、理化学研究所へ投与日、投与量等の連絡をする。
2) 理化学研究所は、実施責任者からの投与日、投与量等の連絡を受け、投与前に iPS-NKT 細胞を実施医療機関の実施責任者へ納入する。
3) 試験実施医療機関の特定細胞加工物（iPS-NKT 細胞）管理者は、実施責任者から試験実施医療機関の長を通じて提供された「特定細胞加工物の管理に関する手順書」に従って管理する。
4) 調剤の時期及び方法については、別途定める手順書に従う。

6.2.2. DC/Gal の管理方法

1) 実施責任者は、症例登録後、未来開拓センター製造部門へ投与日、投与量等の連絡をする。
2) 未来開拓センター製造部門は、実施責任者からの投与日、投与量等の連絡を受け、投与前に DC/Gal を実施責任者へ納入する。
3) 試験実施医療機関の特定細胞加工物（DC/Gal）管理者は、実施責任者から試験実施医療機関の長を通じて提供された「特定細胞加工物の管理に関する手順書」に従って管理する。

7. 観察・検査・評価項目、方法及び実施時期

7.1. 実施スケジュールと手順

観察・検査・評価の実施スケジュールを表 19 に示す。実施責任者又は分担医師は、スケジュールに従って観察・検査等を実施する。なお、被験者背景の調査や臨床検査など、試験協力者が実施可能な項目については、実施責任者の管理下で試験協力者が実施してもよい。

表 19　試験スケジュール

		スクリーニング期	細胞準備期	試験治療期						観察期 i)			中止時
Day<br>※試験治療開始日を Day 1 とする		－42～<br>－8	-7	1	6	7	8	13	19	1	15	28	―
許容範囲（日）				+3	＋3	―	―		±3	±3	±7	±7	＋7
同意取得	●												
被験者背景		●											
既往・合併症		●											
前治療歴		●											
選択除外基準		●											
妊娠検査 a)		●										●	●
感染症検査 b)		●											
胸部 X 線検査		●						●		●	●	●	●
12 誘導心電図検査		●											
造影 CT 検査（血管・腫瘍評価）		●									●h)		●
自家 DC/Gal 作成			●										
投与開始基準の確認				●	●								
自家 DC/Gal 鼻粘膜下投与				●									
iPS-NKT 投与経路確保 c)					●								
iPS-NKT 投与					●								
血液学的検査及び血液生化学検査		●		●	●			●		●	●	●	●
凝固検査		●			●								
尿検査		●		●	●					●	●	●	●
バイタルサイン d)		●	●	←	―	―	―	―	→				
体重		●		●	●			●		●	●	●	●
ECOG PS		●		●	●					●	●	●	●
mMRC				●	●								
PK/PD 用採血 e)					●	●		●					
免疫細胞分画検査 f)				●	●		●	●		●			
有害事象評価		←	―	―	―	―	―	―	―	―	―	―	→
DLT 評価期間				←	―	―	―	―	→				
入院 g)				←	―	―	―	―	―	→			
HLA 検査		●											
抗 HLA 抗体検査		●			●					●			●

a）妊娠可能な女性のみ行う。尿定性検査を実施する。

b）感染症検査には、HIV 抗体、HTLV-1 抗体、HBs 抗原、HBs 抗体、HBc 抗体及び HCV 抗体を含み、HBs 抗原陰性かつ HBs 抗体検査又は HBc 抗体検査が陽性の場合、HBV-DNA 定量検査を実施する。また、HCV 抗体陽性の場合、HCV-RNA 定量検査を実施する。

c）投与法の項に記載があるとおり、iPS-NKT 細胞の投与法（投与経路確保の方法）としてセルジンガー法を用いる。iPS-NKT 細胞投与当日に IVR 室において腫瘍栄養動脈にカテーテル先端を誘導し、iPS-NKT 細胞を直接投与する。

d）バイタルサインとは、体温、脈拍数、呼吸数、SpO_2、血圧（収縮期血圧及び拡張期血圧）を含むものとし、実施責任者又は分担医師の判断に基づき必要に応じて検査（胸部 X 線検査、血液学的・血清学的検査、血液ガス検査等）を追加することができる。

e）PK/PD 測定の採血スケジュールは表 20 に示すとおりとする。

f) 採血検体から PBMCs を分離し、細胞表面マーカーを染色する。

g）DLT 評価期間（自家 DC/Gal 投与から iPS-NKT 細胞投与後 14 日目までの期間）及び観察期間の 1 日目は入院を原則とし、被験者の安全性を慎重にモニタリングする。また、iPS-NKT 細胞投与第 7 日目（試験治療期 Day 14）以降の検査結果及び診察において、Grade 2 以上の非血液学的毒性及び Grade 3 以上の血液学的毒性が発現しておらず、有害事象発生時に実施医療機関及び自宅近隣医療施設へ連絡ができ、緊急時に速やかに患者と連絡が取れる体制が整っている場合にのみ、実施責任者又は分担医師の判断で外泊又は退院することも可能とする。なお、患者都合等により DC/Gal 投与前日の入院は可能とする。

h）試験治療開始前（4 週間以内）、及び観察期 15 日目（iPS-NKT 細胞の最終投与より 28 日後又は投与中止が決定された日から 15 日目のうち、より遅い時点）に造影 CT による腫瘍評価を行う。

i）iPS-NKT 細胞が投与中止となった場合、投与中止が決定された日から観察期開始となる。

表 20　PK/PD 測定の採血スケジュール

	DC/Gal<br>投与前	iPS-NKT<br>投与前	投与後		
実施時点（時間）	—	—	1	24	168
許容範囲（分）	—	—	±15	±30	±60
PK/PD 用採血	—	●	●	●	●

7.1.1. スクリーニング検査

同意取得後、スクリーニング検査を開始する。実施責任者又は分担医師は以下のスクリーニング検査を行い、選択基準を満たし、除外基準に抵触しない患者を登録する。検査項目は以下に記載のとおりとする。なお、実施責任者または試験担当医師の判断で、安全性上等の必要に応じて検査（胸部X線、血液学的および血清学的検査、血液ガス検査等）を追加する場合がある。

- 体重
- バイタルサイン[4)]
- ECOG PS
- 血液学的検査
- 血液生化学検査（HbA1c を含む）
- 凝固検査
- 尿検査
- 妊娠検査（妊娠可能な女性のみ）
- 感染症検査（HBs 抗原、HBs 抗体、HBc 抗体、HCV 抗体、HIV 抗体又は HTLV-1 抗体）
- 12 誘導心電図検査
- 胸部 X 線検査
- 造影 CT 検査（血管・腫瘍評価）
- HLA 検査（Luminex 法によって第 2 区域まで）
- 抗 HLA 抗体検査

7.1.2. 被験者の情報

同意取得時又はスクリーニング検査時に、以下の被験者情報を記録する。

1) 同意取得日
2) 被験者識別コード
3) 性別
4) 年齢
5) 身長
6) 既往・合併症
7) 診断名
8) 組織型
9) 病期分類
10) 体表面積[6)]
11) アレルギーの有無
12) 過去のがん治療歴及び手術歴

---

4) バイタルサインとは、体温、脈拍数、呼吸数、SpO_2、血圧（収縮期血圧及び拡張期血圧）を含むものとする。
6) 藤本式（体表面積（m^2）=体重（kg）$^{0.444}$×身長（cm）$^{0.663}$×88.83÷10000）を用いる。

13) アルコール・喫煙歴

7.1.3. 観察・検査・評価項目

各タイミングにおける検査項目を以下に記す。さらに、入院期間中は毎日、バイタルサイン、有害事象の確認を実施する。臨床検査項目の詳細は、表 21 に示すとおりとする。また、なお、実施責任者又は分担医師の判断で、安全性上等の必要に応じて検査（胸部 X 線、血液学的及び血清学的検査、血液ガス検査等）を追加することは可能とする。

表 21　臨床検査項目

感染症検査<br>（※スクリーニング時のみ）	HBs 抗原、HBs 抗体、HBc 抗体、HCV 抗体、HIV 抗体又は HTLV-1 抗体、HBV-DNA 定量（※HBs 抗原陰性かつ HBs 抗体又は HBc 抗体陽性時に追加）、HCV-RNA 定量（※HCV 抗体陽性時に追加）
HLA 検査<br>（※スクリーニング時のみ）	HLA 遺伝子（HLA-A、B、C、DRB1、DQB1、DPB1）
血液学的検査	赤血球、ヘモグロビン、ヘマトクリット値、血小板数、白血球数、<br>白血球分画（好中球、リンパ球、単球、好酸球、好塩基球）
血液生化学検査<br>（※HbA1c についてはスクリーニング時のみ）	総蛋白、アルブミン、総ビリルビン、AST、ALT、ALP、LDH、BUN、クレアチニン、尿酸、Na、K、Cl、Ca、P、アミラーゼ、HbA1c
凝固検査	PT-INR、aPTT
尿検査	pH、糖、蛋白、ウロビリノーゲン、ケトン、潜血
免疫細胞分画検査	T 細胞数（個/mL）<br>NK 細胞数（個/mL）<br>NKT 細胞（個/mL）<br>CD4+ T 細胞数（個/mL）<br>CD8+ T 細胞数（個/mL）<br>ナイーブ CD4+ T 細胞数（個/mL）<br>セントラルメモリーCD4+ T 細胞数（個/mL）<br>エフェクターメモリーCD4+ T 細胞数（個/mL）<br>エフェクターCD4+ T 細胞数（個/mL）<br>ナイーブ CD8+ T 細胞数（個/mL）<br>セントラルメモリーCD8+ T 細胞数（個/mL）<br>エフェクターメモリーCD8+ T 細胞数（個/mL）<br>エフェクターCD8+ T 細胞数（個/mL）<br>PD-1+CD4+T 細胞割合（CD4+CD3+ T 細胞中）<br>PD-1+CD8+T 細胞割合（CD8+CD3+ T 細胞中）<br>PD-1+NKT 細胞割合（NKT 細胞中の%）<br>PD-1+NK 細胞割合（NK 細胞中の%）<br>WBC 数（個/mL）<br>リンパ球数（個/mL）
抗 HLA 抗体検査	Class I・II 判定、Class I・II 陽性抗体、DSA

7.1.3.1. 細胞準備期（Day -7）

DC/Gal 投与の 1 週間前に以下のバイタルサインを確認した上でアフェレーシスを行い自家 DC/Gal を作成する。

7.1.3.2. 試験治療期

Day1 の DC/Gal 投与前に以下を実施する。
- 体重
- バイタルサイン
- ECOG PS
- mMRC
- 血液学的検査
- 血液生化学検査
- 尿検査
- 免疫細胞分画検査※
- 前治療から 1 カ月以上経過していることの確認

Day6 の iPS-NKT 細胞投与前に以下を実施する。
- 体重
- バイタルサイン
- ECOG PS
- mMRC
- 血液学的検査
- 血液生化学検査
- 凝固検査
- 尿検査
- 免疫細胞分画検査※
- 抗 HLA 抗体検査
- PK/PD 用採血（iPS-NKT 細胞投与 1 時間後）

Day7（iPS-NKT 細胞投与から 24 時間後）に PK/PD 用採血を行う。

Day8 に免疫細胞分画検査※用採血を行う。

Day13 に以下の検査を実施する。
- 体重
- バイタルサイン

・　胸部 X 線検査
・　血液学的検査
・　血液生化学検査
・　免疫細胞分画検査※
・　PK/PD 用採血

※免疫細胞分画検査：採血検体から PBMCs を分離し、細胞表面マーカーを染色する。主に T 細胞分画、NKT 細胞マーカー等がん関連免疫細胞の分画について、フローサイトメトリーを用いて末梢血中の濃度及び割合を算出する。

・　有害事象評価
（DLT 評価期間中、実施責任者は DLT の定義（5.2. 用量制限毒性（DLT）参照）に該当すると疑われる疾患、徴候が発生した際には、速やかにデータモニタリング委員会に判断の妥当性の審議を依頼し、当該患者における試験継続の可否を決定する。）

7.1.3.3. 観察期

観察期の Day 1、15、及び 28 に、以下を実施する。
・　体重
・　ECOG PS
・　血液学的検査
・　血液生化学検査
・　尿検査
・　妊娠検査（妊娠可能な女性のみ、観察期 Day28 に実施）
・　胸部 X 線検査
・　有害事象評価
・　造影 CT 検査（腫瘍評価）（観察期 Day1 のみ実施）
・　免疫細胞分画検査（観察期 Day1 のみ実施）
・　抗 HLA 抗体検査（観察期 Day1 のみ実施）

7.1.3.4. 試験中止時

実施責任者又は分担医師により試験の中止を判断された日から 7 日以内に可能な範囲で以下を実施する。ただし、後治療を開始する場合は、後治療開始前に以下を実施する。
・　体重
・　ECOG PS
・　血液学的検査

・　血液生化学検査
・　尿検査
・　妊娠検査（妊娠可能な女性のみ）
・　胸部 X 線検査
・　有害事象評価
・　造影 CT 検査（腫瘍評価）
・　抗 HLA 抗体検査

7.1.3.5. 腫瘍評価

副次評価項目として、腫瘍に対する DC/Gal 又は iPS-NKT 細胞の効果判定を画像検査で実施する。

スクリーニング時には、頭頸部から骨盤部までの病変の有無を造影 CT により検索する。

iPS-NKT 細胞投与開始後も、頭頸部から骨盤部までの病変を、観察期初日（iPS-NKT 細胞投与から 28 日後又は投与中止が決定された日から 15 日目のうち、より遅い時点）及び中止時に画像検査で確認する。スクリーニング時に頭部及び四肢に病変が認められなかった被験者においても、臨床症状に応じて脳転移及び骨転移の有無を画像検査で確認する。

本試験で実施される造影 CT は、経時的評価を可能とするために可能な限り同一のモダリティ設定で撮影する。

有効性評価は、RECIST1.1 版日本語訳 JCOG 版に従って行う（別紙 1 参照）。有効性評価のための画像診断が実施された場合、実施責任者又は分担医師は速やかに評価を行い、評価結果を記録する。

7.1.3.6. 妊娠の転帰調査

女性被験者又は男性被験者のパートナーが試験期間中に妊娠が判明した場合には、試験期間によらず妊娠の転帰が判明するまで追跡調査を行うこととする。

7.1.3.7. 研究終了後のフォローアップ

本試験終了後、通常診療における範囲内で被験者の診療を行う。

8. 有害事象・品質不良等発生時の取り扱い

8.1. 有害事象・品質不良の定義

有害事象とは DC/Gal 又は iPS-NKT 細胞が投与されたのちに生じる、好ましくない、若しくは意図しない徴候（臨床検査値の異常変動を含む）症状又は疾病のことであり、DC/Gal 又は iPS-NKT

細胞との因果関係を問わない。

既存の病態（試験期間よりも以前に存在した合併症）については、試験期間内に増悪又は発症頻度の上昇がないかぎり、有害事象として扱わない。ただし、DC/Gal 又は iPS-NKT 細胞の投与後に合併症が悪化した場合、有害事象として取扱い、悪化が確認された日を有害事象の発現日とする。

品質不良とは、被験製品等の機能の不全、細胞が人体に及ぼす副作用等広く具合のよくないことをいい、製造・交付・保管又は使用のいずれの段階によるものであるかを問わない。

8.2. 疾病等の定義

疾病等とは、再生医療等の提供に起因するものと疑われる疾病、障害若しくは死亡又は感染症の発生（以下「疾病等の発生」という。）を指す。なお、特定認定再生医療等委員会および厚生労働大臣へ報告の必要な疾病等は、「9.3 疾病等（重篤な有害事象）報告の手順」に従い報告を行う。

8.3. 重篤な有害事象の定義

重篤な有害事象とは、次のいずれかに該当するものとする。

1) 死亡
2) 死亡につながるおそれのあるもの
3) 治療のために病院又は診療所への入院又は入院期間の延長が必要とされるもの
4) 障害
5) 障害につながるおそれのあるもの
6) 3）～5）までに掲げる症例に準じて重篤であるもの
7) 後世代における先天性の疾病又は異常

なお、3）の「入院」には、再検査、追跡調査のための入院又は入院期間の延長、及び試験開始前より予定していた治療又は検査を試験中に実施することのみを目的とした入院（予定手術や検査等）は含まれない。（ただし、その入院中新たに発生したものは有害事象として取り扱う。）

8.3. 有害事象又は品質不良の発生時の被験者への対応

実施責任者又は分担医師は、有害事象又は品質不良の発生を認めたときは、直ちに適切な処置を行うとともに、DC/Gal 又は iPS-NKT 細胞の投与を中止した場合や、有害事象に対する治療が必要となった場合には、被験者にその旨を伝える。なお、実施責任者又は分担医師により、より専門的な診察が必要と判断される有害事象又は症状が認められた場合は、速やかに適切な専門医と連携の上、必要に応じて入院期間の延長を含む対応を可能とする。

試験終了・中止時に DC/Gal 又は iPS-NKT 細胞との因果関係が否定できない有害事象が未回復の場合は、原則として回復又は軽快するまで可能な限り観察を継続する。ただし、実施責任者又は分担医師が本試験の影響は消失しており、被験者の安全性は十分確保され、それ以上の追跡調査は必要ないと判断

した場合はこの限りではない。

8.4. 報告の対象となる有害事象及び品質不良

1) 同意取得日から観察期最終日（中止時）までに発生したすべての有害事象は DC/Gal 又は iPS-NKT 細胞との因果関係の有無に関わらず報告し、有害事象から回復するか観察期最終日（中止時）まで観察する。また、DC/Gal 又は iPS-NKT 細胞との因果関係があると判断された有害事象については、試験期間終了時まですべて報告する。
2) 試験期間中に発生した品質不良情報は、すべて報告する。

8.5. 有害事象及び品質不良発生時の報告手順

上記期間に発生したすべての有害事象及び品質不良について、実施責任者又は分担医師は、カルテ及び症例報告書に齟齬なく記載する。

8.6. 有害事象及び品質不良の評価に必要な記載内容

8.6.1. 有害事象

1) 有害事象の名称
   有害事象名は、原則として診断名・疾患名（病名）で記録する。診断名・疾患名が特定できない場合や実施責任者又は分担医師より診断名・疾患名としないことが妥当と判断された場合には、臨床症状又は徴候を有害事象名とする。
2) 発現日
3) 消失日
4) 転帰：回復、軽快、回復したが後遺症あり、未回復、死亡、不明
5) 処置（DC/Gal 又は iPS-NKT 細胞の投与）：変更なし、中止、投与延期、該当せず
6) その他処置：なし、薬物治療、その他
7) 重篤度：非重篤、重篤
8) 重症度：程度は CTCAEv5.0 日本語訳 JCOG 版に基づき判定する
9) DC/Gal 又は iPS-NKT 細胞との因果関係：関連なし、関連が否定できない

8.6.2. 品質不良

1) 品質不良名
2) 品質不良の確認日
3) 発生日
4) 品質不良が発生した DC/Gal 又は iPS-NKT 細胞の製造番号

5) 品質不良が発生したと考えられる原因及び品質不良状況
6) 品質不良に対する処置
7) 当該品質不良による有害事象の有無
①有の場合：有害事象名
②無の場合：重篤な有害事象発生のおそれの有無

8.6.3. 有害事象の回復性と DC/Gal 又は iPS-NKT 細胞との因果関係

有害事象の回復とは、有害事象がない状態、又は投与前の状態への回復とする。有害事象における DC/Gal 又は iPS-NKT 細胞との因果関係の判定に際しては、被験者の全身状態、合併症、併用薬・併用療法、時間的関係を勘案して判断する。

因果関係なし : 下記以外、又は他の原因による有害事象であることがより強く示唆されている場合。

因果関係が否定できない : DC/Gal 又は iPS-NKT 細胞の投与後、当該事象が発現するまでの時間的関連性がみられ、DC/Gal 又は iPS-NKT 細胞の投与後、時間の経過とともに当該事象が減弱するが、その後の再投与とともに当該事象が再発又は悪化する場合、又は、被験者の全身状態、合併症、併用薬及び併用療法などの影響や交絡するリスク因子の存在が否定的である場合など。

8.7. 疾病等の発生の場合の措置

1) 分担医師は、DC/Gal 又は iPS-NKT 細胞によるものと疑われる疾病等の発生を知ったときは、速やかに実施責任者及び試験調整医師にその旨を報告する。
2) 実施責任者は、別紙様式-C（院内様式）を用いて病院長にその旨を報告する。
3) 病院長又は実施責任者は、分担医師に対し、当該再生医療等の中止その他の必要な措置を講ずるよう指示する。
4) 病院長又は実施責任者は、DC/Gal 及び iPS-NKT 細胞を製造した特定細胞加工物製造事業者（未来開拓センター及び理化学研究所）に対し発生した事態及び講じた措置について速やかに通知する。

8.8. 特定認定再生医療等委員会及び厚生労働大臣への疾病等報告

1) 病院長は以下の疾病等を知ったときは、表 1 に従い特定認定再生医療等委員会及び厚生労働大臣に報告する。
2) 特定認定再生医療等委員会へは、「疾病等報告書」別紙様式第一（省令第三十五条関係）を用いて報告する。また厚生労働大臣へは、jRCT を通じて報告する。

表 1

疾病等の重篤性等	報告先	報告期限
① 死亡<br>② 死亡につながるおそれのある症例	特定認定再生医療等委員会<br><br>厚生労働大臣	7 日
① 治療のために医療機関への入院又は入院期間の延長が必要とされる症例<br>② 障害<br>③ 障害につながるおそれ<br>④ 重篤である症例<br>⑤ 後世代における先天性の疾病又は異常	特定認定再生医療等委員会<br><br>厚生労働大臣	15 日
① 再生医療等の提供によるものと疑われる又は当該再生医療等の提供によるものと疑われる感染症による疾病等の発生	特定認定再生医療等委員会	再生医療等提供計画を厚生労働大臣に提出した日から起算して 60 日ごとに当該期間満了後 10 日以内

8.9. 特定認定再生医療等委員会及び厚生労働大臣への定期報告

1) 病院長は、再生医療等提供計画を厚生労働大臣に提出した日から起算して、1 年ごとに、当該期間満了後 90 日以内に、以下の項目について特定認定再生医療等委員会に報告する。
   - 当該再生医療等を受けた者の数
   - 当該再生医療等に係る疾病等の発生状況及びその後の経過
   - 当該再生医療等の安全性及び科学的妥当性についての評価
   - 当該再生医療等に対する第八条の八第一項各号に規定する関与に関する事項
   - 当該再生医療等に係るこの省令又は再生医療等提供計画に対する不適合の発生状況及びその後の対応
2) 病院長は、特定認定再生医療等委員会に意見を聴いた後、再生医療等提供計画を厚生労働大臣に提出した日から起算して、1 年ごとに、当該期間満了後 90 日以内に、以下の項目について厚生労働大臣に報告する。
   - 特定認定再生医療等委員会の名称
   - 当該特定認定再生医療等委員会による当該再生医療等の継続の適否に係る意見
   - 当該再生医療等を受けた者の数

8.10. 厚生労働大臣への重大事態報告

1) 特定細胞加工物製造事業者は、特定細胞加工物の安全性の確保に重大な影響を及ぼすおそれがある事態（重大事態）が生じた場合には、必要な措置を講じるとともに、その旨を速やかに提

供先の再生医療等提供機関（千葉大学医学部附属病院）及び厚生労働大臣に報告しなければならない。

2) 重大事態が生じた特定細胞加工物を保管する場合においては、当該特定細胞加工物を区分して一定期間保管した後、適切に処理しなければならない。

8.11. 不適合の管理

病院長又は実施責任者は、本試験が再生医療等法又は再生医療等提要計画に適合していない状態であると知ったときは、速やかにその旨を病院長に報告する。

また、病院長又は実施責任者は特に重大な不適合が判明した場合、速やかに特定認定再生医療等委員会の意見を聴くこととする。

9. 評価項目

9.1. 主要評価項目

投与用量における用量制限毒性（DLT）発現割合

【主要評価項目の設定根拠】<br>本試験の目的は、DC/Gal と iPS-NKT 細胞との併用療法の忍容性を評価することであり、医薬品等の第 I 相試験における代表的な評価項目である DLT 発現割合に基づく評価を行う。

9.2. 副次評価項目

【有効性の副次評価項目】
- 奏効割合（RECIST ver.1.1）
- 病勢コントロール割合（RECIST ver.1.1 に基づき CR、PR 又は SD と評価された患者の割合）

【安全性の副次評価項目】
- 有害事象の発現状況（種類、頻度及び重症度等）
- 臨床検査値の推移

【有効性の副次評価項目の設定根拠】

本試験は DC/Gal 及び iPS-NKT 細胞の併用療法を用いた悪性腫瘍に対する FIH 試験であることから、有効性に関する評価を副次評価項目として設定した。

【安全性の副次評価項目の設定根拠】

DC/Gal 及び iPS-NKT 細胞の併用療法がヒトに与えうる有害事象について網羅的に確認するため、DLT 評価の他に、本試験で発現したすべての有害事象について可能な限り詳細に評価を行うために、安全性の副次評価項目を設定した。

9.3. 探索的評価項目

- iPS-NKT 細胞投与後 1 週における末梢血内 iPS-NKT 細胞の残存有無
- 動態測定（iPS-NKT 細胞投与後 7 日目における末梢血中 iPS-NKT 細胞濃度）

【探索的評価項目の設定根拠】

iPS-NKT 細胞のヒト体内における、作用機序及び iPS-NKT 細胞が被験者の免疫動態・機能に及ぼす影響等を明らかにする目的として、探索的評価項目を設定した。

9.4. 免疫学的評価項目

免疫学的検査用採血を用いて下記の免疫学的解析を行う。

- 免疫細胞分画
- 末梢血免疫細胞における Omics 解析（遺伝学的検査を含む）

iPS-NKT 細胞を頭頸部がん患者の腫瘍栄養動脈に投与することにより、末梢血免疫細胞の分化、機能に与える分子生物学的影響を探索的に検討するために、末梢血免疫細胞における Omics 解析（以下、「Omics 解析」）を行う。

Omics 解析は、各被験者から Omics 解析に関する同意を取得した上で、末梢血の薬物動態及び免疫細胞分画評価のために採取された血液検体（約 12 mL）の残余検体を用いて行う。当該検体の解析に際し、個人を特定できる情報を削除し、新たな ID を付与し匿名化した上で、必要に応じて千葉大学及び理化学研究所にて解析を行う。解析により明らかとなった遺伝情報について、研究開始時点において遺伝情報の有用性は明らかでないことから、被験者には原則非開示とする。しかし、遺伝情報の有用性が明らかになった場合、及び偶然に他の重大な病気との関係が見つかり、被験者や血縁者の希望や有益性を考慮した上で、生命倫理審査委員会の審議を経て、遺伝情報を開示することがある。なお、試料及びデータの取扱いについて、「16.1. 試料の保存」及び「16.2. 検体の廃棄」に従うものとする。

10. 統計学的事項

本試験の統計解析計画の概要を以下にまとめた。なお、統計解析計画の詳細は、統計解析計画書に記載する。統計解析計画書において本研究計画書の概要を修正することがあるが、主要評価項目の定義や解析方法が変更される場合には、本研究計画書を改訂する。

10.1. 解析対象集団

10.1.1. 安全性解析対象集団及び DLT 評価対象集団

本試験に登録され、DC/Gal を投与された症例を安全性解析対象集団とする。ただし、再生医療等法不遵守例は安全性解析対象集団から除外する。また、安全性解析対象集団のうち、DC/Gal 又は iPS-NKT 細胞と関連する毒性以外の理由で DLT 評価期間中の評価が適切に実施できなかった被験者を除くすべての被験者を DLT 評価対象集団とする。解析対象から除外された対象者が発生した場合には新たな被験者を当該コホートに追加する。

10.1.2. 最大の解析対象集団（full analysis set：FAS）

本試験に登録され、1 回以上 DC/Gal 又は iPS-NKT 細胞を投与され、有効性データがあるすべての被験者を最大の解析対象集団（FAS）とする。ただし、ベースラインのデータが取得されなかった被験者及び、重大な研究計画書違反　（同意未取得、等）の被験者については除外する。

10.1.3. 研究計画書に適合した対象集団（per protocol set：PPS）

FAS から、試験方法や併用療法など研究計画書の規定に対して、以下の重大な違反があった症例を除いた被験者とする。

選択基準違反、除外基準違反、併用禁止薬違反、併用禁止療法違反

10.2. 目標症例数と設定根拠

解析対象例数：2 名～6 名

【設定根拠】<br>本試験は、自家 DC/Gal と iPS-NKT 細胞の併用療法の忍容性の検討並びに安全性及び有効性を探索的に評価することを目的としているため、検証目的とした有意差検定は実施しない。単一用量に対して 3+3 デザインで実施される本試験の最大の解析対象例数は 6 例と設定した。また、最小症例数は DLT が 2 例ずつ発現した場合となることから 2 例と設定した。

10.3. 症例の取り扱い

原則として登録された症例については、試験調整医師及び統計解析責任者が協議の上、症例の取り扱いを決定する。新たな問題が起こった場合の症例の取り扱いについても、試験調整医師及び解析責任者が協議の上、決定する。

10.4. データの取り扱い

データ集計・解析時におけるデータの取り扱いについては、原則として以下に示すとおりとする。疑義が生じた場合は、統計専門家と試験調整医師が協議の上データ固定前に決定する。

欠測値に対しては補完を行わない。

10.5. 統計解析項目及び解析計画

すべての症例において DC/Gal 又は iPS-NKT 細胞の投与が終了し、データが固定された後に解析を行う。

安全性の解析は、DLT 評価対象集団を対象として DLT 発現について、MedDRA/J の器官別大分類（SOC）及び基本語（PT）ごとに頻度表を作成する。また、副次評価として、安全性解析対象集団における下記の解析を実施する。

有効性評価において、最大の解析対象集団 （FAS） 及び研究計画書に合致した解析対象集団（PPS） における解析を行う。

統計解析の詳細はデータ固定前に別途作成する統計解析計画書に規定する。

主要評価項目

- 設定された単一用量における用量制限毒性（DLT）発現割合

副次評価項目

【安全性の副次評価項目】

- 有害事象の発現状況（種類、頻度及び重症度等）
- 臨床検査値の推移

【有効性の副次評価項目】

- 奏効割合（RECIST ver.1.1）
- 病勢コントロール割合（RECIST ver.1.1）

探索的評価項目

- 末梢血中 iPS-NKT 細胞濃度推移
- 免疫細胞分画（T 細胞分画、NKT 細胞マーカー等）
- 末梢血免疫細胞における Omics 解析

10.5.1. 被験者背景の解析

各解析対象集団における被験者背景データの分布及び要約統計量を算出する。名義変数については、カテゴリの頻度及び割合を群ごとに示す。連続変数については要約統計量を算出する。

10.5.2. 安全性及び有効性の解析

10.5.2.1. 主たる解析

DLT 評価の対象集団において、DLT の定義に基づき投与量ごとの DLT 発現例数及びその割合と95%信頼区間を算出する。

10.5.2.2. 副次解析

主たる解析結果を補足する考察を行う目的で以下の副次評価項目の解析を行う。

安全性の評価項目について、DC/Gal 又は iPS-NKT 細胞投与後に発現したすべての有害事象について、MedDRA/J の器官別大分類（SOC）及び基本語（PT）ごとに頻度表を作成する。用量別、重篤度別、DC/Gal 又は iPS-NKT 細胞との因果関係別、CTCAE Grade 別に、同様の頻度表を作成する。

臨床検査値に関しては、要約統計量を算出する。また、ベースラインと各時点との差の要約統計量を算出する。また、臨床検査値の推移図も作成する。

有効性の評価項目について、RECIST v.1.1 に基づく実施責任者又は分担医師評価による奏効割合と病勢コントロール割合に関して、該当した被験者の人数とその割合を算出し、それらの割合の95%信頼区間を算出する。本試験は探索的試験であるため、統計解析により得られる仮説検定の P 値及び信頼区間は、意味のある変動を把握し推定することを意図するものであり、効果の有無等を結論づけられるものではない。その他の項目及び詳細については統計解析計画書に記載する。

10.5.3. 中間解析

本試験では中間解析は行わない。

10.6. データモニタリング委員会

データモニタリング委員会は、実施責任者から独立した関係にある悪性腫瘍の専門家から構成され、実施責任者からの報告内容につき、以下の項目について審議する。

1) 実施責任者の求めに応じて、発生した有害事象と DC/Gal 又は iPS-NKT 細胞との因果関係の有無や、既知／未知の判断の妥当性について審議し意見を述べる。特に、発現した有害事象が

本製品によるものか、原疾患の悪化によるものか等の DLT への該当性に係る最終的な判定は、本委員会にて行う。

2) DLT を含むすべての有害事象等の安全性情報を評価した上での、試験継続の可否の判断。
3) 上記事項に対応した研究計画書改正・改訂の要否、説明同意文書改訂の要否の判断。

データモニタリング委員会への審議依頼及び審議方法、審議結果の通知方法は別に作成されるデータモニタリング委員会標準業務手順書に拠る。

10.7. 最終解析

追跡期間終了後、データが得られ症例が固定された後に解析を行う。統計解析責任者が「解析報告書」をまとめ、試験調整医師及び実施責任者に提出する。試験調整医師は解析報告書の内容を総括し、試験全体の結論、問題点、結果の解釈及び考察、今後の方針等を主として臨床的観点からまとめた「総括報告書」を作成し、実施責任者の承認を得る。

11. 研究計画書の遵守及び逸脱

1) 実施責任者又は分担医師は、本研究計画書を遵守して試験を実施する。
2) 実施責任者又は分担医師は、研究計画書から逸脱した場合、すべての逸脱の詳細及び理由を記録する。
3) 被験者の緊急の危機を回避するためその他医療上やむを得ない理由により実施計画書から逸脱した場合、実施責任者は、逸脱の内容及びその理由を記載した文書を実施医療機関の長に直ちに提出するとともに、当該文書の内容を実施医療機関の長を経由して特定認定再生医療等委員会に速やかに報告する。

12. 研究計画書、症例報告書又は解析計画に関する変更

12.1. 研究計画書及び症例報告書の改訂

研究計画書及び症例報告書を改訂する場合には、以下の手順により行う。

1) 実施責任者は、DC/Gal 又は iPS-NKT 細胞の品質、有効性及び安全性に関する事項、その他試験を適正に行うために重要な情報を知ったときは、必要に応じて当該研究計画書を改訂する。また、改訂の際には改訂履歴を作成し、それを保存する。
2) 実施責任者は、必要に応じ研究計画書の改訂に併せて又は他の理由により症例報告書を改訂する。また、改訂の際には改訂履歴を作成し、それを保存する。
3) 実施責任者は研究計画書改訂版及び症例報告書用紙改訂を速やかに実施医療機関の長に提出し、実施医療機関の長を経由して速やかに特定認定再生医療等委員会及び生命倫理審査委員会に諮る。
4) 特定認定再生医療等委員会及び生命倫理審査委員会の意見に基づく実施医療機関の長の指示が実施責任者の許容できる範囲内で、研究計画書及び症例報告書用紙を修正する場合も同様の

手順とする。

12.2. 統計解析計画の変更

統計解析責任者は、統計解析計画書の内容を変更した場合、変更内容をすべて本試験の統計解析報告書に記載する。なお、統計解析計画書の変更は、その経緯を記録に残す。

13. 試験の中止、中断又は終了

13.1. 試験全体での中止又は中断の基準

実施責任者は、以下のいずれかの情報が得られ、試験全体の続行が困難であると考えられる時には、試験調整医師と試験全体の中止又は中断を決定する。

1) DC/Gal 又は iPS-NKT 細胞に関する新たな安全性情報又は重篤な有害事象の情報が得られる等、試験の安全性確保が困難になった場合。
2) 実施医療機関が、研究計画書からの重大な逸脱を行い、改善が見られない場合。
3) 2 例以上に DLT が発生した場合。DLT に該当しない被験者については、速やかに観察期に移行し、すべての被験者の試験期間が終了し次第、試験を終了する。
4) その他、試験実施中に試験の中止・中断が必要と考えられる新たな情報が得られた場合。

13.2. 試験全体での中止又は中断する場合の手続き

実施責任者は、DMC などへの諮問又は協議により試験全体を中止又は中断する場合には、実施医療機関の長にその旨とその理由を詳細に速やかに文書で通知する。また、投与中の被験者に対して速やかにその旨を伝え、適切な治療への変更等の適切な処理を行うとともに被検者の安全性を確保する検査などを実施する。

13.3. 試験の終了

実施責任者は、試験終了後、実施医療機関の長に試験が終了した旨を文書で通知し、試験結果の概要を文書で報告する。

14. データマネジメント

14.1. データマネジメントの手順

データマネジメントに関する詳細な手順については、データマネジメント計画書に記載する。

14.2. データの収集

実施責任者又は分担医師は、症例報告書を作成する。実施責任者又は分担医師は、症例報告書の記載内容の変更、修正又は追記に当たっては、症例報告書を用いてデータセンターに連絡し、すべて電子情報として記録する。なお、実施責任者は、分担医師が症例報告書を作成した場合並びに試験協力者が原資料（原データ）から症例報告書に転記した場合には、当該症例報告書が提出される前にその内容について点検し、問題がないことを確認する。実施責任者は、最終的に電子症例報告書を電子媒体（例 : CD-R 等）にて保存する。実施責任者は、電子症例報告書の見読性、保存性を担保する。

EDC システムの使用にあたり、実施医療機関は EDC のトレーニングを受講し、入力方法の詳細は別途入力マニュアルを参照する。

14.3. 症例報告書の直接記載され、かつ原資料（原データ）を解すべき資料の特定

本試験においては、以下の文書などを原資料（原データ）とする。

1) 被験者の同意及び被験者への情報提供に関する記録、診療録、看護記録、臨床検査データ及び画像検査フィルム等症例報告書作成の基となった記録。なお、電子カルテに格納されたデータも原資料とみなす。
2) DC/Gal 又は iPS-NKT 細胞投与に関する記録
3) 本試験に関連する指針上必要な試験に係る文書又は記録

症例報告書に記載されたデータのうち、以下に示す項目は症例報告書の記載をもって原資料（原データ）とする。ただし、診療録等に記載のある場合は、当該診療録等を原資料（原データ）とみなす。

1) 併用薬・併用療法の目的
2) 有害事象の程度、転帰（追跡調査時の結果を含む）、重篤度、DC/Gal 又は iPS-NKT 細胞との因果関係の判定
3) 被験者の試験中止理由
4) 実施責任者又は分担医師のコメント

15. 原資料及びその他の記録の保存

15.1. 試験実施医療機関による記録の保存

再生医療等法に規定される試験実施医療機関において保存すべき試験に係る文書又は記録は、病院長が次の日のうちいずれかの遅い日までの期間保存する。

1) DC/Gal 又は iPS-NKT 細胞提供者が被験製品に係る再生医療等製品についての製造販売の承認を受ける日（開発が中止された場合には、開発中止が決定されてから 3 年が経過した日）
2) 試験の中止又は終了後 30 年が経過した日

実施責任者は、試験実施医療機関又は特定認定再生医療等委員会及び生命倫理審査委員会が保存すべき記録について保存が不要となった場合、試験実施医療機関に通知する。

15.2. 実施責任者による記録の保存

再生医療等法及び省令に規定される実施責任者が保存すべき試験に係る文書又は記録は、適切と判断される保管場所において、試験の中止又は終了後 30 年が経過した日までの期間保存する。

16. 採取した細胞の一部等と再生医療等に用いた細胞加工物の一部の保存期間

16.1. 試料の保存

試料は試験終了後 10 年間、千葉大学医学部附属病院未来開拓センターに保存する。保存方法は液体窒素での凍結保存とする。保存場所のセキュリティーは大学病院出入口及び未来開拓センターロック付きドアで保護される。

16.2. 検体の廃棄

被験者から同意の撤回があった場合、検体の取り違えや混入が起きるか又はそれらが強く疑われる場合、その他廃棄の必要性を認めた場合には、匿名化番号などを削除したうえで廃棄する。

16.3. データの利用

本試験で得られた情報は、共同研究先である理化学研究所と共有する。また、次相以降の開発のため、本試験で得られた情報を営利企業と共有する場合がある。

16.4. 試料及びデータの再利用

余剰検体は 16.1 に示したとおり保存され、試料や情報の 2 次利用（千葉大学におけるプロテオーム解析、トランスクリプトーム解析、ゲノム解析及びエピゲノム解析を含む）を行うことがある。その場合の倫理審査及び同意取得の方法については、対応する倫理指針等に従うものとする。

17. 原資料の直接閲覧

試験実施医療機関の長及び実施責任者は、モニタリング、監査、特定認定再生医療等委員会及び生命倫理審査委員会又は規制当局による調査の際に、原資料等すべての記録を閲覧できることを保証する。試験が適切に実施されていること及びデータの信頼性が十分に確保されていることを確認する。直接閲覧の方法、実施時期についてはモニタリング計画書に別途定める。

18. 試験の品質管理及び品質保証

18.1. 品質管理

1) 本研究計画書からの逸脱した行為があった場合は、実施責任者又は分担医師は、本実施計画書に定めるところに従う。
2) 実施責任者又は分担医師は、症例報告書を本研究計画書に従って作成する。
3) 実施責任者は、症例報告書に記載されたすべてのデータ及びその他の記録が正確及び完全であることを確認する。
4) 症例報告書に記載されたデータのうち、原資料と何らかの矛盾がある場合は、実施責任者はその理由を説明する記録を作成・保存する。
5) 実施責任者は、当該モニタリングの対象となる実施医療機関において当該試験に従事していない者をモニターとして指名し、モニタリング標準業務手順書に従いモニタリングを実施させる。モニターは、下記の事項を確認する。
   － 被験者の人権、安全及び福祉が保護されている
   － 再生医療等法及び施行規則、最新の研究計画書及び当該試験に係る標準業務手順書を遵守して実施している。
   － 実施責任者又は分担医師から報告されたデータ等が正確かつ完全で原資料等の試験関連記録と照らし検証できる
6) データマネジメント責任者は、別途定めた標準業務手順書に従ってデータマネジメント計画を立案し、データの取扱い各段階で品質管理を行い、その品質を確保する。

18.2. 品質保証

実施責任者は、監査に関する計画書及び業務に関する標準業務手順書を作成し、当該計画書及び標準業務手順書に従って監査を実施させる。なお、監査担当者は、当該監査に係る DC/Gal 又は iPS-NKT 細胞の開発を担当する者でなく、また、当該監査に係る試験を実施する医療機関において当該試験の実施（その準備及び管理を含む。）及びモニタリングに従事していない者とする。

19. 倫理及び再生医療等法

本試験の実施に際しては、「ヘルシンキ宣言」及び再生医療等法に従って行われる。また、本研究計画書及び関連する標準業務手順書を遵守して施行される。

また、実施責任者又は分担医師は、被験者の選定にあたって、人権保護の観点並びに選択基準と除外基準に基づいて被験者の健康状態、症状、年齢、性別、同意能力、実施責任者等との依存関係、他の試験を含む臨床試験への参加の有無を十分に考慮したうえで、試験への参加を求めることの適否を慎重に検討する。

加えて、本試験において試験参加中の被検者のための苦情及び問い合わせを受け付ける窓口を設置し、いつでも問い合わせを受け付けることができる体制を整えている。

20. 審査する委員会

本試験の実施に先立ち、実施医療機関の特定認定再生医療等委員会及び生命倫理審査委員会は、本試験の倫理的、科学的及び医学的妥当性を審査する。本試験は、特定認定再生医療等委員会及び生命倫理審査委員会の承認を得た後に実施する。特定認定再生医療等委員会及び生命倫理審査委員会の審議結果が「修正の上で承認する」であった場合には、審議結果に基づいて実施計画書又は症例報告書、同意説明文書等を修正し承認された後、本試験を実施する。また、特定認定再生医療等委員会及び生命倫理審査委員会は少なくとも 1 年に 1 回以上の頻度で本試験が適切に実施されているか否かを継続的に審査する。

21. 健康被害補償及び保険

本試験に参加した結果として被験者に健康被害が生じた場合、実施責任者はその治療に関する医療体制の提供など必要かつ適切な処置を行う。

本試験では、試験期間中の試験行為に起因して被験者に健康被害（死亡を含む。）が生じた場合に、損害てん補を受けられる臨床研究保険に加入する。なお、保険期間は、西暦 2022 年 9 月 1 日から西暦 2024 年 8 月 31 日までの試験期間に 1 年の観察期間が加わった西暦 2025 年 8 月 31 日までである。損害てん補を受けられる被保険者は、実施責任者、分担医師及び試験実施機関などこの試験に携わる者となる。

臨床研究保険によって補償されるのは、被験者に説明し、同意を取得した健康被害の補償内容に基づき実施責任者又は試験実施機関が被験者に支払うべき補償金である。ただし、実施責任者・分担医師が試験行為上の過失等により、被験者に対して法律上の賠償責任を負担する場合には、補償金はこの損害賠償金に充当される。

22. 試験に関する費用負担

本試験に係わる費用は下記のとおりとする

(1) 本試験で使用される DC/Gal は千葉大学医学部附属病院未来開拓センターより提供され、iPS-NKT 細胞は国立研究開発法人　理化学研究所より提供される。

(2) 試験に係る診療費用はすべて研究者が負担する。

(3) 被験者の負担軽減費は、本試験で別途定める「被験者への支払いに関する資料」に基づき、試験実施医療機関が負担する。

23. 研究資金及び利益相反

本試験は、国立研究開発法人日本医療研究開発機構（AMED）再生医療実用化研究事業の以下の研究費にて実施する。

事業名：多能性幹細胞（iPS／ES 細胞）、体性幹細胞等を用いて、再生医療等安全性確保法に従って実施する臨床研究（再生医療実用化研究事業）

研究代表者：国立研究開発法人　理化学研究所　古関　明彦

千葉大学医学部附属病院は、理化学研究所と再委託契約を締結し、試験を実施する。iPS-NKT 細胞は理化学研究所より提供される。

また、特定認定再生医療等委員会及び生命倫理審査委員会審議前に利益相反管理が適切になされているか審議が行われ、実施責任者又は分担医師及び試験協力者は利益相反状態になっていないことが確認される。

24. 試験のデータベース登録

本試験は、最初の被験者からの同意取得前に臨床研究実施計画・研究概要公開システム（jRCT）（https://jrct.niph.go.jp/）に登録を行う。

25. 試験実施体制

本試験の実施体制は、別紙 5 参照。

26. 参考資料・文献リスト

1. Barbara B, Kevin JH, Richard G et al. Pembrolizumab alone or with chemotherapy versus cetuximab with chemotherapy for recurrent or metastatic squamous cell carcinoma of the head and neck (KEYNOTE-048): a randomised, open-label, phase 3 study. The Lancet. 2019; 10212: 1915-28.
2. Ferris RL, Blumenschein G Jr, Fayette J, Guigay J, Colevas AD, Licitra L, Harrington K, Kasper S, Vokes EE, Even C, Worden F, Saba NF, Iglesias Docampo LC, Haddad R, Rordorf T, Kiyota N, Tahara M, Monga M, Lynch M, Geese WJ, Kopit J, Shaw JW, Gillison ML. Nivolumab for Recurrent Squamous-Cell Carcinoma of the Head and Neck. N Engl J Med. 2016; 375: 1856-1867.
3. Taniguchi M, Harada M, Kojo S, Nakayama T, Wakao H. The regulatory role of Vα14 NKT cells in innate and acquired immune response. Annu Rev Immunol. 2003; 21: 483-513.
4. Kawano T, Cui J, Koezuka Y, Toura I, Kaneko Y, Motoki K, Ueno H, Nakagawa R, Sato H, Kondo E, Koseki H, Taniguchi M. CD1d-restricted and TCR-mediated activation of Vα14 NKT cells by glycosylceramides. Science. 1997; 278: 1626-9.
5. Kawano T, Nakayama T, Kamada N, Kaneko Y, Harada M, Ogura N, Akutsu Y, Motohashi S, Iizasa T, Endo H, Fujisawa T, Shinkai H, Taniguchi M. Antitumor cytotoxicity mediated by ligand-activated human Vα24 NKT cells. Cancer Res. 1999; 59: 5102-5.
6. Taniguchi M, Seino K, Nakayama T. The NKT cell system: bridging innate and acquired immunity. Nat Immunol. 2003; 4: 1164-5.
7. Fujii S, Shimizu K, et al. Innate Vα14+ natural killer T cells mature dendritic cells, leading to strong adaptive immunity. Immunol Rev 2007; 220: 183-98.
8. Ishikawa A, Motohashi S, et al. A phase I study of α-galactosylceramide （KRN7000）-pulsed dendritic cells in patients with advanced and recurrent non-small cell lung cancer. Clin Cancer Res 2005; 11: 1910-7.
9. Motohashi S, Ishikawa A, Ishikawa E, Otsuji M, Iizasa T, Hanaoka H, Shimizu N, Horiguchi S, Okamoto Y, Fujii S, Taniguchi M, Fujisawa T, Nakayama T. A phase I study of in vitro expanded natural killer T cells in patients with advanced and recurrent non-small cell lung cancer. Clin Cancer Res. 2006; 12: 6079-86.

10. Kobayashi K, et al. The effect of radiotherapy on NKT cells in patients with advanced head and neck cancer. Cancer Immunol Immunother 2010; 59: 1503-9.
11. Horiguchi S, Matsuoka T, Okamoto Y et al. Migration of Tumor Antigen-Pulsed Dendritic Cells After Mucosal Administration in the Human Upper Respiratory Tract. J Clin Immunol 2007; 27: 598-604.
12. Kurosaki M, et al. Migration and immunological reaction after the administration of α-GalCer-pulsed antigen-presenting cells into the submucosa of patients with head and neck cancer. Cancer Immunol Immunother. 2011; 60: 207-15.
13. Uchida T, Horiguchi S, Tanaka Y et al. Phase I Study of α-galactosylceramide-pulsed antigen presenting cells administration to the nasal submucosa in unresectable or recurrent head and neck cancer. Cancer Immunol Immunother. 2008; 57: 337-345.
14. Kunii N, Horiguchi S, Motohashi S et al. Combination therapy of in vitro-expanded natural killer T cells and α-galactosylceramide-pulsed antigen-presenting cells in patients with recurrent head and neck carcinoma. Cancer Sci. 2009; 100: 1092-8.
15. Yamasaki K, et al. Induction of NKT cell-specific immune responses in cancer tissues after NKT cell-targeted adoptive immunotherapy. Clin Immunol. 2011; 138: 255-65.

---

i 国立研究開発法人理化学研究所. iPS-090-001,002　製造工程フロー図 （version 3）. （2018）.

ii 国立研究開発法人理化学研究所.バリデーションポイントの設定. （2017）.

iii 金光 弘幸. 最終報告書: iPS-NKT 細胞のヌードマウスを用いた単回静脈内投与毒性試験. 株式会社ボゾリサーチセンター. （2015） N-TT150002.

iv 望月 秀美. 最終報告書：ヒト iPS-NKT 細胞のマウスを用いた用量設定試験. 株式会社イナリサーチ. （2017） NB16320.

v 望月 秀美. 最終報告書：ヒト iPS-NKT 細胞の NOG マウスを用いた一般毒性試験. 株式会社イナリサーチ. （2018） NB17321.

vi 伊藤浩太. 最終報告書：α-ガラクトシルセラミドパルス樹状細胞（Chiba-NKT）の 2 回および 4 回投与による全身毒性試験. 株式会社化合物安全性研究所 2016. SR15350

vii 伊藤 雅彦. 最終報告書：軟寒天コロニー形成試験法を用いた iPS-NKT 細胞の造腫瘍性試験. 株式会社ボゾリサーチセンター. （2015） N-BT150002

viii 伊藤 格試. 最終報告書: ヒト NKT-iPS 細胞の NOG マウスを用いた造腫瘍性評価試験. 株式会社日本バイオリサーチセンター.（2017） 370292

ix 六角 香. 最終報告書: ヒト NKT-iPS 細胞の NOG マウスを用いた造腫瘍性評価試験. 日精バイリス株式会社.(2020) 10300.

x 望月 雅裕. 経過報告書: ヒト iPS-NKT 細胞の NOG マウスを用いた造腫瘍性評価試験. 株式会社ボゾリサーチセンター. （2018） OT-170003.

xi Ishikawa, S. Motohashi, E. Ishikawa et al. A Phase I Study of α-Galactosylceramide （KRN7000）– Pulsed Dendritic Cells in Patients with Advanced and Recurrent Non–Small Cell Lung Cancer. Clin. Cancer Res. （2005） 11: 1910-1917.

xii Motohashi S, Nagato K, Kunii N, et al. A Phase I-II Study of α-Galactosylceramide-Pulsed IL-2/GM-CSF-Cultured Peripheral Blood Mononuclear Cells in Patients with Advanced and Recurrent Non-Small Cell Lung Cancer. J. Immunol （2009） 182: 2492-2501

xiii Motohashi S, Ishikawa A, Ishikawa E, et al. A phase I study of in vitro expanded natural killer T cells in patients with advanced and recurrent non-small cell lung cancer. Clin Cancer Res. （2006） 15; 12: 6079-86

xiv Uchida T, Horiguchi S, Tanaka Y, et al. Phase I study of α-galactosylceramide-pulsed antigen presenting cells administration to the nasal submucosa in unresectable or recurrent head and neck cancer. Cancer Immunol. Immunother. （2008） 57: 337-345.

xv Kunii N, Horiguchi S, Motohashi S, et al. Combination therapy of in vitro-expanded natural killer T cells and α-galactosylceramide-pulsed antigen-presenting cells in patients with recurrent head and neck carcinoma. Cancer Sci. （2009） 100: 1092-1098.

xvi Yamasaki K, Horiguchi S, Kurosaki M, et.al. Induction of NKT cell- specific immune responses in cancer tissues after NKT cell-targeted adoptive immunotherapy. Clin. Immunol. 2011; 138: 255–265.

xvii Toyoda, T, Kamata, T, Tanaka, K, et al. Phase II study of α-galactosylceramide-pulsed antigen-presenting cells in patients with advanced or recurrent non-small cell lung cancer. J. Immunother. Cancer 2020; 8(1): e000316.
