# Supplementary material for: A phase I clinical trial to evaluate the tolerability and safety of an allogeneic iPSC-derived iNKT cell and α-GalCer-pulsed autologous DC combination therapy for patients with recurrent and advanced head and neck cancer: A study protocol
Source: PLoS One. 2026 Feb 26;21(2):e0342387. doi: 10.1371/journal.pone.0342387 (PMC12944769; doi:10.1371/journal.pone.0342387)
Supplement: S6 File — (PDF) [file pone.0342387.s006.pdf]

Study Protocol

A Phase I clinical trial to evaluate the tolerability, safety and efficacy of iPS-NKT cell intra-arterial administration and autologous DC/Gal combination therapy for patients with recurrent and advanced Head and Neck Cancer

Type I Regenerative medical technology

Protocol ID：CUH_ iPSNKT_DCGal_001

**Version**	**1.8**
**Date of issuance**	**March 7, 2023**

Amendment History

Date of Issue	version	Detail
03/22/2022	1.0	Create new
03/28/2022	1.1	Correction of errors and addition of clinical trial results of similar products
04/16/2022	1.2	Deletion of port method in administration method, unification of terminology, and improvement of description
06/01/2022	1.3	Addition of nonclinical efficacy data, change in number of the iPS-NKT cells administered, unification of terminology, correction of errors
06/12/2022	1.4	Unification of unit descriptions, correction of errors
08/29/2022	1.5	Correction of errors
10/17/2022	1.6	Revision in response to suggestions from the Regenerative Medicine Evaluation Subcommittee
2/6/2023	1.7	Revision in response to suggestions from the Regenerative Medicine Evaluation Subcommittee
3/7/2023	1.8	Revision in response to suggestions from the Regenerative Medicine Evaluation Subcommittee

＜**Table of Contents**＞

0. Synopsis 11
1. Background 17
1.1. Study background 17
1.1.1. Head and Neck Cancer and its therapeutic strategies 17
1.1.2. NKT cell immunotherapy 17
1.1.3. Application of iPS cell technology 18
1.1.4. Combination of iPS-NKT cells and autologous DC/Gal 18
1.2. iPS-NKT cell and auto-DC/Gal 19
1.2.1. Summary of iPS-NKT cell and auto-DC/Gal 19
1.2.1.1. Summary of iPS-NKT 19
1.2.1.2. Summary of auto-DC/Gal 20
1.2.2. iPS-NKT cell and DC/Gal Manufacturing 21
1.2.2.1. iPS-NKT cell Manufacturing 21
1.2.2.2. Standardized donor screen test for iPS-NKT cells (Table 1) 23
1.2.2.3. NKT-iPS cell screen test (Table 2) 23
1.2.2.4. NKT-iPS Cell MCB/WCB Standardized Test (Table 3 and 4) 24
1.2.2.5. iPS-NKT cell (frozen) Specification test (Table 5) 25
1.2.2.6. iPS-NKT cell (product) specification test (Table 6) 26
1.2.2.7. The stability of iPS-NKT cell (product) (Table 8) 28
1.2.2.8. Auto-DC/Gal manufacturing 32
1.2.2.9. Specification test on the auto-DC/Gal 33
1.2.2.10. Stability test on auto-DC/Gal 34
1.2.3. Efficacy on preclinical study 34
1.2.3.1. Anti-tumor effect of iPS-NKT cell (K562 cell) 34
1.2.3.2. Anti-tumor effect of iPS-NKT cell against (FaDu cell) 35
1.2.3.3. NK cell activation potential of iPS-NKT cells (adjuvant activity) 36
1.2.3.4. Combination use of iPS-NKT cell and DC/Gal 36
1.2.3.5. In vivo anti-tumor effect and intra-tumor immune cell analysis (DC/Gal combination, human lung cancer PDX) 37
1.2.4. General toxicity and tumorigenicity studies 38
1.2.4.1. General toxicity test on iPS-NKT cell 38
1.2.4.2. General toxicity test on DC/Gal 39
1.2.4.3. Toxicity study of combination dose (NKT-deficient B6, human IL7/15 expressing NSG mice) 39
1.2.4.4. Tumorigenic test 40
1.2.5. Pharmacological and kinetic studies 43

1.2.5.1. Productivity of IFN- γ and IL-4 of iPS-NKT cell 43
1.2.5.2. Anti-tumor effect of iPS-NKT cell (*in vitro*) 43
1.2.5.3. Pharmacodynamic test of iPS-NKT cell 44
1.2.6. Clinical study related to NKT and DC/Gal (Efficacy and Safety) 46
1.2.7. Expected Side Effects 50
1.3. Rationale 50
1.3.1. Dosing 50
1.3.2. Target population 51
1.3.3. Endpoint 51
1.3.4. Safety management of subjects 51
2. Objective and necessity of Study 52
3. Study Subjects 52
3.1. Inclusion Criteria 52
3.2. Exclusion Criteria 53
4. Informed Consent 55
4.1. Preparation and Revision of Consent and Other Explanatory Documents 55
4.2. Timing and Method on Informed Consent 55
4.3. Contents of Explanation for subjects 56
5. Study Method 57
5.1. Design 57
5.2. Dose Limiting Toxicity (DLT) 58
5.3. Sample size and Study Duration 59
5.4. Registration of Study Site and Subject 59
5.4.1. Study site 59
5.4.2. Subjects 59
5.4.3. Responsible department for registration 60
5.5. Handling of Patients Who Are Not Registered 60
5.6. Dosing schedule and route 60
5.7. Criteria for administration 60
5.8. Criteria for postpone 60
5.9. Criteria of treatment and study discontinuation for each subject 61
5.9.1. treatment discontinuation 61
5.9.2. Study discontinuation 61
5.10. Concomitant drugs and therapy 62
5.10.1. Permitted concomitant drug/therapy 62
5.10.2. Record on concomitant drug/therapy 62

5.10.3. Prohibited drug/therapy 62
5.11. Actions to be taken after discontinuation or termination of the study 63
6. Specified cell processed product 64
6.1.1. Packaging/label of iPS-NKT cell 64
6.1.2. Packaging/label of auto-DC/Gal 64
6.2. Administration and dispensing methods 65
6.2.1. Administration and dispensing methods of iPS-NKT cell 65
6.2.2. Management method of auto-DC/Gal 65
7. Method and Timing of Observations/tests/assessments 65
7.1. Study Schedule and Procedure 65
7.1.1. Screening test 63
7.1.2. Subject Information 63
7.1.3. Observation, test, assessment measurements 64
7.1.3.1. Cell preparation period (Day -7) 65
7.1.3.2. Treatment period 65
7.1.3.3. Observation Period 66
7.1.3.4. Study Discontinuation 66
7.1.3.5. Tumor Evaluation 67
7.1.3.6. Pregnancy Outcome Study 67
7.1.3.7. Follow-up after study completion 67
8. Handling of Adverse Events, Quality Defects, etc. 68
8.1. Definition of Adverse Event and Quality Defect 68
8.2. Definition of Disease, etc. 68
8.3. Definition of Serious Adverse Event 68
8.3. Response to Subjects in occurrence of the Adverse Events or Quality Defects 69
8.4. Reportable Adverse Events and Quality Defects 69
8.5. Reporting Procedures for Adverse Events and Quality Defects 69
8.6. Description required for evaluation of adverse events and quality defects 69
8.6.1. Adverse event 69
8.6.2. Quality defects 70
8.6.3. Causal relationship between adverse events and DC/Gal or iPS-NKT cells, and recoverability of adverse events 70
8.7. Measures to be taken in case of outbreak of disease, etc. 71
8.8. Reporting of disease, etc. to the Specified Authorized Regenerative Medicine Committee and the Minister of Health, Labour, and Welfare 71

8.9. Annual reports to the Specified Authorized Committee for Regenerative Medicine and the Minister of Health, Labour and Welfare 72
8.10. Report of Serious Incidents to the Minister of Health, Labor and Welfare 73
8.11. Management of incompliance issue 73
9. Endpoints 73
9.1. Primary endpoint 73
9.2. Secondary endopoints 74
9.3. Exploratory endopoints 74
9.4. Exploratory Immunological evaluation 74
10. Statistical Considerations 75
10.1. Population to be analyzed 75
10.1.1. Safety analysis population and DLT evaluation population 75
10.1.2. Largest analyzed population (full analysis set：FAS） 75
10.1.3. Subject population conforming to the research protocol (per protocol set：PPS） 75
10.2. Sample size and Rationale 76
10.3. Case Handling 76
10.4. Data Handling 76
10.5. Statistical analysis items and analysis plan 76
10.5.1. Subject Background Analysis 77
10.5.2. Safety and efficacy analysis 77
10.5.2.1. Primary analysis 77
10.5.2.2. Secondary analysis 77
10.5.3. The interim analysis 78
10.6. Independent Data Monitoring Committee 78
10.7. Final Analysis 78
11. Compliance and deviation from the research protocol 79
12. Changes to the research protocol, case report form, or analysis plan 79
12.1. Revision of research protocols and case report forms 79
12.2. Change in statistical analysis plan 79
13. Study Discontinuation, suspension, or termination 80
13.1. Criteria for discontinuation or suspension of the study 80
13.2. Procedures for discontinuation or suspension of the study 80
13.3. Study completion 80
14. Data Management 80
14.1. Data management procedure 80
14.2. Data collection 81

14.3. Specification of materials that are directly described in the case report and that should be interpreted as source documents (source data) 81
15. Preservation of source documents and other records 81
15.1. Record keeping by the study site 81
15.2. Record keeping by the PI 82
16. Storage period of a part of harvested cells, etc. and a part of cellular processed products used for regenerative medicine 82
16.1. Storage of samples 82
16.2. Disposal of samples 82
16.3. Extent of the data use 82
16.4. Secondary use of samples and data 83
17. Direct access to source documents 83
18. Quality Control and Quality Assurance 83
18.1. Quality Control 83
18.2. Quality Assurance 84
19. Ethics and the Act on the Safety of Regenerative Medicine (ASRM) 84
20. Review Committee 84
21. Compensation and Insurance 84
22. Study Costs 85
23. Research Funding and Conflicts of Interest 85
24. Study Registration 85
25. Study structure 85
26. Refferences 86

Abbreviations

Abbreviation	Definition
ALP	alkaline phosphatase
ALT	alanine aminotransferase
AMED	Japan Agency for Medical Research and Development
aPTT	activated partial thromboplastin time
AST	aspartic aminotransferase
BUN	blood urea nitrogen
CDDP	cisplatin
CI	confidence interval
Cmab	cetuximab
CMV	cytomegalovirus
CT	computed tomography
CTCAE	common terminology criteria for adverse events
DC	dendritic cell
DC/Gal	αGalCer pulsed DC
CTL	Cytotoxic T lymphocyte
DLT	dose limiting toxicity
EBV	Epstein-Barr virus
ECOG	Eastern Cooperative Oncology Group
EDC	electronic data capture
ELISA	enzyme-linked immune-sorbent assay
ELISpot	enzyme-linked immunoSpot
ER/ES	electronic records/electronic signature
FACS	fluorescence-activated cell sorter
FAS	full analysis set
FDG	2-Deoxy-2-[18F]fluoroglucose
FIH	first in human
G-CSF	granulocyte-colony stimulating factor
GLP	Good Laboratory Practice
GVHD	graft versus host disease
HBV	hepatitis B virus
HCV	hepatitis C virus
HIV	human immunodeficiency virus
HLA	human leukocyte antigen
HNSCC	head and neck squamous cell carcinoma
HTLV	human T-lymphotropic virus
IFN	interferon
IL	interleukin

Abbreviation	Definition
iPS	induced pluripotent stem cells
iPS-NKT	
JCOG	Japan Clinical Oncology Group
LDH	lactate dehydrogenase
MCB	master cell bank
MedDRA/J	Medical Dictionary for Regulatory Activities
MEM	Minimum Essential. Medium
MHC	major histocompatibility complex
mMRC	modified Medical Research Council
MTD	maximum tolerated dose
NE	Not evaluable
NK	natural killer （cells）
NKR	natural killer receptor
NKT	natural killer T （cells）
NKT-iPS	
NOG	NOD.Cg-Prkdcscid Il2rgtm1Sug/Jic
NYHA	New York Heart Association
OS	overall survival
ParvoB19	Parvovirus B19
PBMC	peripheral blood mononuclear cells
PBS	phosphate buffered saline
PCR	polymerase chain reaction
PD	pharmacodynamics
PET	positron emission tomography
PI	Principal investigator
PK	pharmacokinetics
PPS	per protocol set
PS	performance status
PT	preferred terms
PT-INR	prothrombin time-international normalized ratio
PTT	partial thromboplastin time
QOL	quality of life
RECIST	response evaluation criteria in solid tumors
SI	Sub-investigator
SOC	system organ class
TCR	T-cell receptor
TD	toxicodynamic
TK	toxicokinetic

Abbreviation	Definition
WCB	working cell bank
WNV	West Nile Virus
αGalCer	α-Galactosylceramide
ASRM	The Act on the Safety of Regenerative Medicine

0. Synopsis

Title	Phase I Study of Tolerability, Safety, and Efficacy of iPS-NKT Cell Injection Therapy and Autologous DC/Gal Combination Therapy in Patients with Recurrent or Advanced Head and Neck Cancer
Study Objective	To evaluate the tolerability of iPS-NKT cell intra-tumoral administration and autologous DC/Gal combination therapy, as well as the safety and efficacy of the therapy, in patients with recurrent or advanced head and neck cancer after standard treatment who are refractory to radical therapy.
Study Design	Single site, unblinded, uncontrolled study
Study Phase	Phase I
Specified cell processed material	iPS derived NKT cell (iPS-NKT)<br>αGalCer activated autologous dendric cell (auto-DC/Gal)
Inclusion criterion	Patients who meet all the following criteria will be eligible.<br>1) Patients with recurrent or advanced head and neck cancer, refractory or intolerant to standard of care and who have evaluable lesions that can be treated with intra-arterial infusion to tumor.<br>2) Patients who have not been on previous therapy within 1 month. Any type of previous treatment is acceptable.<br>3) Patients must be at least 20 years old but less than 80 years<br>4) Patients must have an ECOG Performance Status of 2 or less.<br>5) Patients who meet the following laboratory data. Hemoglobin ≥ 10 g/dL, White blood cell count ≥ 3000/μL and platelet count ≥ 75,000/μL, Serum creatine ≤ 1.5 mg/dL, Total bilirubin ≤ 1.5 mg/dL, AST (GOT), ALT (GPT) < 2.5 times the upper limit of facility reference values, SpO2 (under room air) ≥ 93%.<br>6) Patients who are expected to have a prognosis of 3 months or more.<br>7) Patients for whom written consent has been obtained from the individual.

Exclusion criterion	Patients who meet any of the following conditions are not eligible.<br>1) Patients who are HBs, HCV, HIV or HTLV-1 antibody-positive or HBs antibody-negative but have HBV-DNA detected by HBV-DNA quantitative testing.<br>2) Patients who have been taking or injecting corticosteroids (methylprednisolone 10 mg/day or higher or equivalent) or immunosuppressive drugs within at least 2 weeks prior to the start of the study product.<br>3) Women who are pregnant, lactating, or planning to become pregnant during the study and men who do not agree with using any of effective contraceptive methods under the guidance of a physician during the study period or up to 14 days after the last dose of the study product.<br>4) Patients with active autoimmune disease requiring systemic or immunosuppressive therapy with corticosteroids or biologic agents.<br>5) Patients who have experienced immune-related adverse events with immune checkpoint inhibitors.<br>6) Patients with poorly controlled diabetes mellitus.<br>7) Patients with severe lung disease (mMRC Breathlessness Scale Grade 2 or higher) or with a history of non-infectious interstitial lung disease requiring steroid treatment.<br>8) Patients with significant cardiac disease (NYHA class III or greater).<br>9) Patients with concurrent multiple cancers.<br>10) Patients who are unable to use contrast agents in radiograph (e.g., allergy, kidney disfunction).<br>11) Patients with a history of hypersensitivity to human serum albumin products or proteins of foreign origin.<br>12) Patients who, at the time of consent acquisition, are participating in other clinical trials or clinical studies and are receiving other investigational products or are judged by the PI or SI to have residual effects of adverse events caused by such products.<br>13) Patients with completely identical genotypes of HLA-A, B and C to the investigational product.<br>14) Patients who are prohibited with blood apheresis because of comorbidities such as unstable angina, A-V block class 2 or greater, WPW syndrome, complete left bundle branch block, systolic blood pressure of 90 mmHg or less, or 170 mmHg or more.<br>15) Patients who are judged to be unsuitable to participate in the study.

Endpoints	Primary Endpoint<br>The occurrence rate of the Dose Limiting Toxicity (DLT)<br>Secondary Endpoint<br>【Efficacy】<br>・ Response rate (RECIST ver.1.1)<br>・ Disease control rate (RECIST ver.1.1)<br>【Safety】<br>・ Adverse events (type, frequency and severity, etc.)<br>・ Blood Biochemistry Test values<br>Exploratory Endpoints<br>・ Concentration of iPS-NKT cells in peripheral blood<br>・ Immune cell fractionation (T cell fractionation, NKT cell markers, etc.)<br>・ Omics analysis of peripheral blood immune cells
Administration dose/method	1) We assess the DLTs of the first three subjects as the first step of this study. If any of the first three patients experienced the DLT, we add the extra three patients. If two or more patients have DLT, we stop this study. If one or fewer of the six patients have DLT, the dose can be considered as tolerable. Then, we can continue the study to assess the second dose with the same manner. Finally, if one or fewer of the six patients have DLT in the second dose cohort, the IDMC will define the dose as the MTD.<br>2) The iPS-NKT cell is administered 5 days after administration of auto-DC/Gal.<br>3) The number of auto-DC/Gal is 1×10^8 cells/body, and iPS-NKT is 3.0×10^7 cells/m^2<br>4) The number of administration of the auto-DC/Gal and the iPS-NKT is once each<br>5) The combination therapy should not be administered to more than 2 subjects on the same day, and the next subject should start the study treatment after an interval of at least 7 days<br>6) Auto-DC/Gal should be administered submucosa of the nasal concha using a microinjection syringe, and the iPS-NKT cells should be administered into tumor feeding artery via super-selective arterial infusion.

DLT definition	We defined the DLTs as the following AEs related to the auto-DC/Gal or the iPS-NKT cell product observed during the treatment period, same as the DLT assessment period. The Grade assessment is evaluated by the CTCAE ver.5.0 Japanese version JCOG edition. 1) Grade 4 or higher hematological toxicities, 2) any non-disease-related blood toxicity requiring any transfusion or G-CSF administration, 3) grade 3 or higher non-hematological toxicities (excluding transient clinical laboratory abnormalities, diarrhea, nausea, vomiting, or other manageable systemic symptoms that recovered to grade 2 or below under appropriate treatments), and 4) AEs lead to blood transfusion. If the PI suspects the occurrence DLTs, the PI can consult the Independent Data Monitoring Committee (IDMC), and the IDMC determines the trial's continuation for the patient.<br>DLT assessment will be evaluated step by step. We assess the DLTs of the first three subjects as the first step of this study. If any of the first three patients experienced the DLT, we add the extra three patients. If two or more patients have DLT, we stop this study. If one or fewer of the six patients have DLT, the IDMC will define the dose as the MTD. DLT assessment will be conducted based on the evaluation of all the safety information, including DLT, the IDMC decides to proceed with the next dose level cohort or the MTD.
Criteria for administration	For patients enrolled in the study, administration of the auto-DC/Gal or the iPS-NKT cells will be initiated after confirming that none of the "Criteria for Discontinuation of Administration" are met on the date of DC/Gal or iPS-NKT cell administration.
Criteria for deferral of administration	In cases where any of the following criteria are met, the PI or SI may postpone the administration of the auto-DC/Gal or the iPS-NKT cells for up to 3 days.<br>1) The PI or SI determines that administration of the DC/Gal or iPS-NKT cells is inappropriate.<br>2) All adverse events occurring after the administration of the auto-DC/Gal or the iPS-NKT cells do not recover to a manageable Grade 2 or Grade 1 or below, or to baseline.<br>3) 3) Infectious disease requiring treatment develops within 24 hours prior to the administration of iPS-NKT cells, including the development of fever of 38°C or higher.

Criteria for drug suspension	If any of the following criteria are met, the PI or SI will discontinue administration of DC/Gal or iPS-NKT cells.If administration is discontinued, the patient will be shifted to the observation period from the date when the decision to discontinue administration was made, and testing and evaluation will be continued in accordance with the testing schedule.<br>1) DLT occurs after the auto-DC/Gal administration, or all adverse events of Grade 3 or higher that occurred during the study period do not recover or improve to Grade 1 or clinically manageable Grade 2 by the time immediately prior to the auto-DC/Gal or the iPS-NKT cell administration.<br>2) Infectious disease requiring treatment develops and does not recover or improve to Grade 1 or clinically manageable Grade 2 by the time immediately prior to administration of DC/Gal or iPS-NKT cells.<br>3) SpO_2 (room air) is maintained below 90 %<br>4) Grade 2 or higher on the mMRC shortness of breath scale in the examination before the auto-DC/Gal or the iPS-NKT cell administration<br>5) Discontinuation request from the subject<br>6) Otherwise in which the need for discontinuation of the auto-DC/Gal or the iPS-NKT cell administration to the subject by the PI or SI.
Criteria for study discontinuation of subject	1) When progression of the primary disease requiring concomitant use of prohibited drugs or therapy is recognized, as judged by the PI or SI.<br>2) Discontinuation requests from the subject<br>3) When the subject is found to be ineligible after enrollment.<br>4) Otherwise in which the PI or SI determines that the subject is unable to receive the auto-DC/Gal
Prohibited concomitant drug and therapy	・ Corticosteroids, immunosuppressive drugs, other antineoplastic drugs, radiation therapy, or surgery including tumor resection.<br>・ Any transfusion therapy (however, if the above drugs or therapies are necessary in response to an adverse event thought to be caused by DC/Gal or iPS-NKT cells, the above drugs or therapies may be used with the adverse event as DLT)
Duration of study participation for each subject	The study period for each subject will be from the date consent is obtained to the end of the last observation. If the subject requests to discontinue the study or if it becomes difficult to observe or conduct the study due to transfer to a different hospital or other reasons, the date of discontinuation and the date of completion of the final observation shall be the date of discontinuation and the date of completion of the final observation.
Sample size	2～6
Study duration	After completion of the study notification to MHLW – 08/31/2024

Number of study site	Single site: Chiba University Hospital
Principal Investigator (PI)	Tomohisa Iinuma<br>Assistant professor, Otolaryngology, Head and Neck surgery, Graduate school of Medicine, Chiba University
Study Coordinating physician	Shin-ichiro Motohashi (Board Chair)<br>Professor, Medical Immunology, Graduate school of Medicine, Chiba University<br>Toyoyuki Hanazawa<br>Professor, Otolaryngology, Head and Neck surgery, Graduate school of Medicine, Chiba University<br>Hideki Hanaoka<br>Professor, Clinical Research Center, Chiba University Hospital
Provider of study products	Auto-DC/Gal: The Center for Advanced Medicine, Chiba University Hospital<br>iPS-NKT cell: The Center for Integrative Medical Sciences, institute of physical and chemical research (RIKEN)
Ethics and Regulation	In conducting this study, the ethical principles based on the "Declaration of Helsinki," the "The Act on the Safety of Regenerative Medicine" and other relevant regulatory requirements shall be observed
Review Committee	Prior to the implementation of this study, the Specified Authorized Regenerative Medicine Committee of the study site, the Bioethics Review Committee, and the Health Sciences Council (Regenerative Medicine Evaluation Subcommittee) will review the ethical, scientific, and appropriateness of the study. This study will be conducted after obtaining approval from the Specified Authorized Regenerative Medicine Committee, the Bioethics Review Committee, and the Health Sciences Council (Regenerative Medicine Evaluation Subcommittee). If the deliberation results of the Specified Authorized Committee for Regenerative Medicine, etc. and the Bioethics Review Committee are "Approval with modifications," the study will be conducted after the protocol, case report form, informed consent form, etc. are modified and approved based on the deliberation results. In addition, the Specified Authorized Regenerative Medicine Committee and the Bioethics Review Committee shall continuously review whether the study is being conducted appropriately at least once a year.

1. Background

1.1. Study background

1.1.1. Head and Neck Cancer and its therapeutic strategies

According to estimates from the regional cancer registries of the Japan National Cancer Center, the incidence of head and neck cancer is 8.6 per 100,000 population for oral and pharyngeal cancer and 2.8 for laryngeal cancer, and head and neck cancer as a whole is thought to account for about 5% of all cancers in Japan. The squamous cell carcinoma is the dominant pathology in head and neck cancer, and it is becoming possible to treat non-advanced cancers with oral surgery and intensity-modulated radiation therapy without reducing quality of life while maintaining high treatment outcomes. For advanced cancer, concurrent chemoradiotherapy and salvage surgery if necessary, or radical resection surgery and postoperative chemoradiotherapy are the first-line treatment options, but even with these highly invasive treatments, the 5-year survival rate for most advanced head and neck cancers remains around 40-50%. Even in patients who achieve complete response to these therapies, there is no effective prophylaxis against local recurrence or distant metastatic recurrence. In the KEYNOTE-048 trial, pembrolizumab alone or in combination with pembrolizumab, platinum, and 5-FU significantly improved overall survival (OS) compared with conventional chemotherapy [1] and is considered the standard of care for these patients. However, the median OS is 2.0-4.7 months, and the drug is not indicated for all patients with head and neck cancer. In the CHECKMATE-141 study in head and neck cancer patients who were refractory to platinum-based chemotherapy, nivolumab, an immune checkpoint inhibitor, demonstrated a median overall survival of 2.4 months compared to other chemotherapy agents [2]. Nivolumab is approved for the treatment of head and neck cancer with recurrent and/or distant metastases in many countries around the world. However, the response rate of nivolumab in the CHECKMATE-141 trial was limited to 13%, and there is a need to develop new immunotherapy for head and neck cancer patients.

1.1.2. NKT cell immunotherapy

NKT cells are unique lymphocytes that express both T cell antigen receptor (TCR) and NK receptor (NKR) on their cell surface [3]. The αGalCer has been identified as a ligand [4]. They directly injure cancer cells by releasing perforin and granzymes, and indirectly injure cancer cells by activating NK cells and CTLs via IFN-γ production [5, 6, 7].

In a clinical study of the safety and efficacy of intravenous administration of activated NKT cells and dendritic cells pulsed with αGalCer (DC/Gal) in non-small cell lung cancer, no serious adverse events were observed and induction of anti-tumor immune response derived from NKT cells was confirmed [8, 9].

In a clinical study on patients with head and neck cancer, it was also found that the decrease in peripheral blood NKT cell count generally observed in cancer patients was not observed in patients with head and neck cancer. Also, the NKT cells, unlike normal T cells, are resistant to radiation therapy, which is widely used as a standard treatment for head and neck cancer [10]. According to these evidences, the NKT cell immunotherapy is expected to be useful in the treatment of head and neck cancer. In clinical studies conducted so far at Chiba University Hospital, DC/Gal was

found to migrate to cervical lymph nodes and act to induce activated NKT cells when administered submucosally in the nasal concha [11, 12]. Therefore, a phase I study of nasal submucosal administration of DC/Gal was conducted in patients with recurrent or advanced head and neck cancer not indicated for curative treatment. No serious adverse events, the increased anti-tumor immune activity and tumor shrinkage were observed [13]. A phase II study of DC/Gal nasal submucosal administration is currently being analyzed as a double-blind, randomized, controlled trial aimed at evaluating the efficacy of NKT cell immunotherapy in suppressing recurrence in patients with HNSCC after curative treatment.

In addition to the nasal submucosal administration, intraarterial administration of tumor nutrient arteries is also being investigated as NKT cell immunotherapy. Most head and neck cancers are controlled by the terminal circulation of the external carotid artery, and it is possible to administer antineoplastic agents into the tumor-trophoblastic artery (hereinafter referred to as "super-selective infusion"). Compared to intravenous administration, super-selective infusion of antineoplastic agents is expected to be more effective locally and to reduce systemic side effects. Two clinical trials have been conducted using this technology in combination with submucosal administration of DC/Gal and super-selective infusion of activated NKT cells in patients with local recurrence after standard treatment. Since December 2007, submucosal DC/Gal and activated NKT cells into the arteries before salvage surgery in patients with locally recurrent head and neck cancer (Phase II clinical study) was conducted in 10 patients to confirm the efficacy and safety of the treatment method, and tumor shrinkage was observed in 5 patients and tumor growth was suppressed in 5 patients.

1.1.3. Application of iPS cell technology

The therapeutic efficacy of NKT cells is limited by the small number of NKT cells (0.01% to 0.1% of leukocytes) and the high variability among patients. Therefore, it is expected that many NKT cells differentiated, matured, and proliferated in vitro using iPS cells can be administered to patients with head and neck cancer by super-selective infusion, thereby demonstrating even greater clinical efficacy.

In a preclinical study using iPS-NKT cells being developed in collaboration with RIKEN, six cancer cell lines in vitro (K562; human leukemia, NCI-H460; human large cell carcinoma, A549; human lung cell basal epidermoid adenocarcinoma, HT-29; human colon cancer, COLO205; human colon cancer, Detroit562; human pharyngeal carcinoma), showed the micro- and macroscopic inhibition of tumor growth in transplanted mice with human head and neck cancer, without any safety concerns. Based on these results, a Phase I study has undergone as an investigator-initiated clinical trial (jRCT2033200116) to evaluate the tolerability and safety of super-selective iPS-NKT cell transfusion in patients with recurrent or advanced HNSCC patients who are refractory to curative treatment.

1.1.4. Combination of iPS-NKT cells and autologous DC/Gal

As described in 1.1.2 above, stimulation by DC/Gal is important for the activation of NKT cells, and therefore, the combination therapy of iPS-NKT cells and autologous DC/Gal (auto-DC/Gal) is expected to be more

effective than the treatment of iPS-NKT cells alone, based on the same activation mechanism for iPS-NKT cells. In preclinical studies in mice, iPS-NKT cells were found to be more effective than auto-DC/Gal cells alone. In a preclinical study in mice, anti-tumor efficacy was observed with auto-DC/Gal combination therapy in tumors derived from patients who had failed to show anti-tumor efficacy with iPS-NKT cells alone. In a heterologous mouse model, there were no serious adverse events associated with auto-DC/Gal combination therapy.

Based on these results, we have planned to conduct a phase I study to evaluate the tolerability and safety of combination therapy with iPS-NKT cells and auto-DC/Gal in patients with recurrent or advanced head and neck cancer who are refractory to curative treatment, as in the physician-led clinical trial.

1.2. iPS-NKT cell and auto-DC/Gal

1.2.1. Summary of iPS-NKT cell and auto-DC/Gal

1.2.1.1. Summary of iPS-NKT

1) Name of the specific cellular processed product (code name): iPS-NKT
2) Ingredient name or expected generic name of the specific cellular processed product: Human iPS cell derived NKT cell
3) Raw materials for specific cellular processed products: NKT cells derived from healthy adult donor peripheral blood

   Dosage form, specification, appearance and composition of the specified cellular processed products:

   Dosage form: Injection

   Specification: See Table 6

   Appearance: light red colored suspension

   Composition: αMEM (20% human AB blood serum, 5 ng/mL IL-7, 10 ng/mL IL-15 included)
4) Raw material collection site: Chiba University Hospital

   1-8-1 Inohana, Chuo-ku, Chiba-shi, Chiba 260-8677 Japan
5) Cell culture processing site for specific cell processed products:

   RIKEN Center for Integrative Medical Sciences

   1-7-22 Suehiro-cho, Tsurumi-ku, Yokohama-shi, Kanagawa 230-0045 Japan
6) Cell Characteristics: This specific cell process is a CD3-positive lymphocyte-like cell characterized by expression of NKT-specific TCR (Vα24, Vβ11)
7) Cell purity: Population of cells that are CD3 positive and express NKT-specific TCR (Vα24, Vβ11) (CD3, TCR Vα24, TCR Vβ11 positive cells)
8) Expected function in the body: iPS-NKT cells are expected to directly injure cancer cells in tumor tissues by selective intra-arterial administration to tumor-feeding artery. It is also expected to exert anti-tumor effects by activating NK cells and CTLs through adjuvant action by DC-mediated activation. Some cells may circulate throughout the body but are expected to be eliminated early because of alien cell rejection.

9) Lifespan: Although the lifespan in the human body is unknown, it is considered that the cells are not viable because they are alien cells.
10) Effect on other cells: This specific cell process is a human iPS cell-derived NKT cell, which has self-renewal ability but has a proliferation limit. The possibility of nonspecific damage to host cells by GVHD was not observed in studies using immunocompetent humanized mice. Therefore, the possibility of damage to other cells by GVHD is considered low.
11) Cell safety: see section 1.2.4.

1.2.1.2. Summary of auto-DC/Gal

1) Name of the specific cellular processed product (code name): DC/Gal
2) Ingredient name or expected generic name of the specific cellular processed product: αGalCer pulsed dendric cell
3) Raw materials for specific cellular processed products: autologous peripheral mononuclear cell
4) Dosage form, specification, appearance and composition of the specified cellular processed products:
   Dosage form: Injection
   Specification: See Table 11
   Appearance: light yellow colored suspension
   Composition: saline solution (2.5% human albumin included)

5) Raw material collection site: Chiba University Hospital
   1-8-1 Inohana, Chuo-ku, Chiba-shi, Chiba 260-8677 Japan
6) Cell culture processing site for specific cell processed products:
   Chiba University Hospital Center of advanced Medicine
   1-8-1 Inohana, Chuo-ku, Chiba-shi, Chiba 260-8677 Japan
7) Cell Characteristics: This specific cell process is a group of CD45-positive mononuclear cells characterized by expression of CD86.
8) Cell purity: Population of cells that are CD45 positive and express at least 15% of CD86, a co-stimulatory molecule for the T cell antigen receptor (CD45, CD86 positive cells)
9) Expected function in the body: According to Kurosaki, M. (Cancer Immunol Immunother, 2011) et al., when administered to the nasal mucosa, a portion of DC/Gal is expected to migrate to the cervical lymph nodes and exhibit anti-tumor effects by activating NKT cells and iPS-NKT cells(2011). Some of the cells also circulate throughout the body and are expected to exhibit anti-tumor effects by activating iPS-NKT cells.
10) Lifespan: The exact lifespan in the human body is unknown, but according to Kurosaki, M. (Cancer Immunol Immunother, 2011) et al., it is detectable even one week after nasal submucosal administration.
11) Effect on other cells: DC/Gal is an antigen-presenting cell derived from autologous peripheral blood mononuclear cells and is thought to be activated by NKT cells recognizing GalCer presented on the cells and exerts an antitumor effect. The possibility of GVHD and effect on other cells induced by DC/Gal administration

is considered unlikely because DC/Gal is an autologous cell and was not observed in a study using DC/Gal in combination with iPS-NKT cells in immune system humanized mice

12) Cell Safety: See section 1.2.4.

1.2.2. iPS-NKT cell and DC/Gal Manufacturing

1.2.2.1. iPS-NKT cell Manufacturing

An outline of each manufacturing process (production of key intermediates, induction culture and frozen stock preparation, and purification culture), the timing of specification tests (NKT-iPS cell specification test, iPS-NKT in-process control test, and iPS-NKT specification test) at each stage, and control items during manufacturing are shown in Fig. 1. A detailed manufacturing process flow diagram is shown in Figure 2 [Ref. i.]. The establishment of validation points in the manufacturing process is attached in Reference ii.

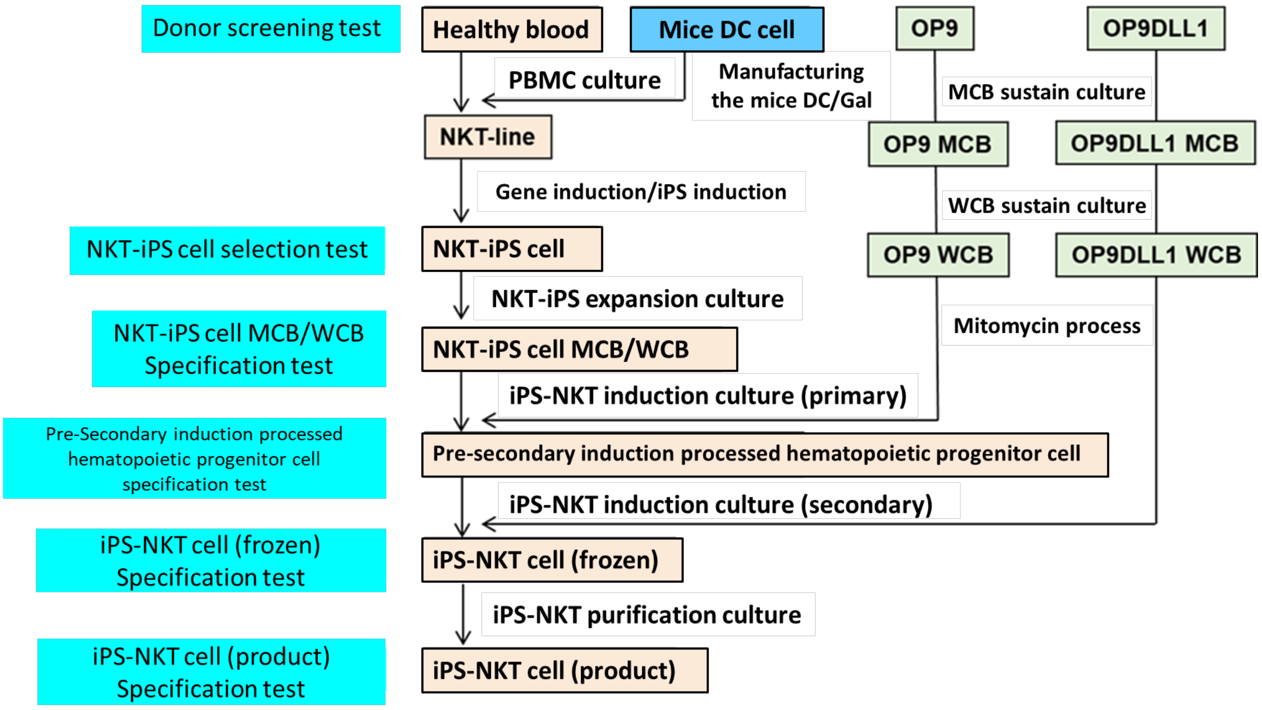

Fig.1. Summary and elements of iPS-NKT cell manufacturing

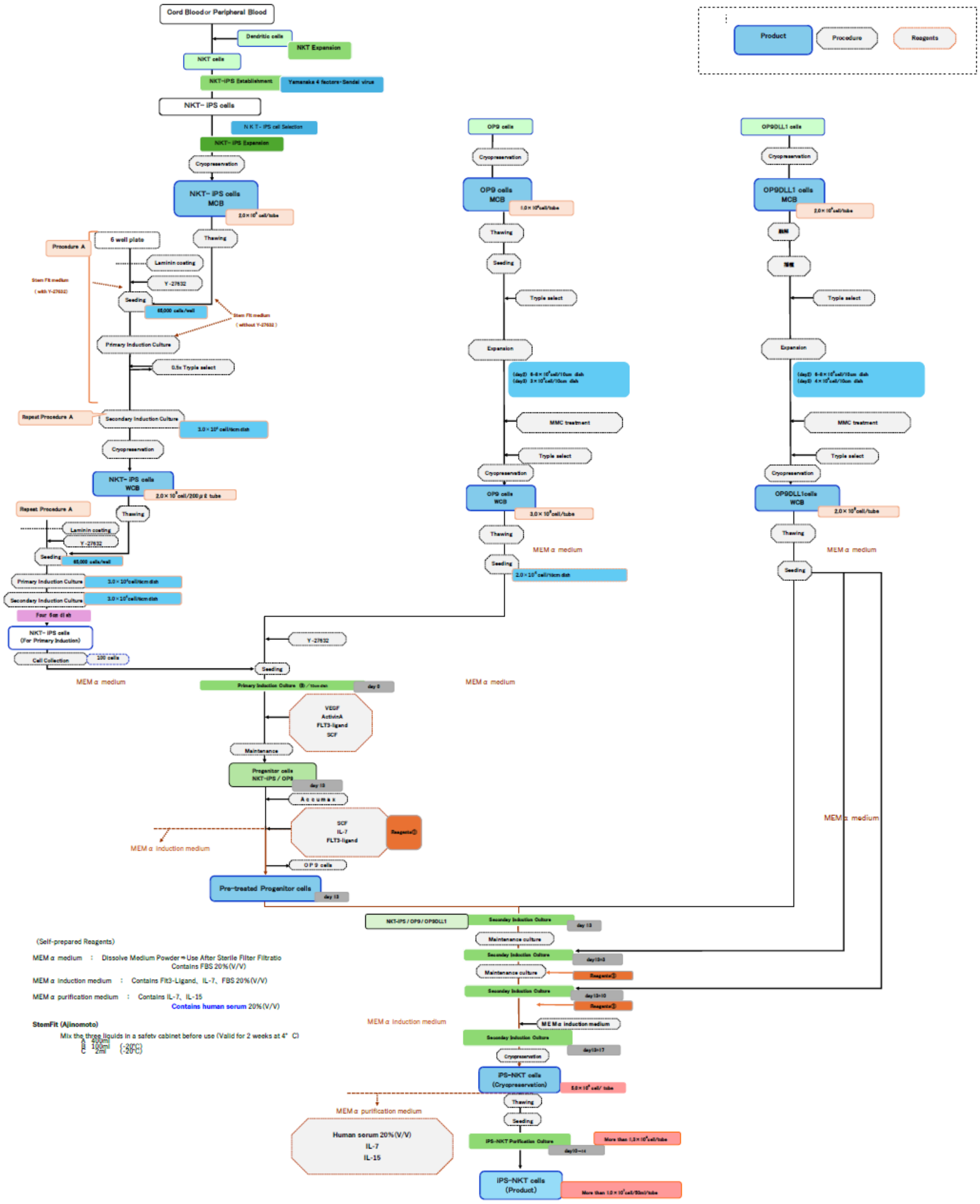

Fig. 2. Detailed summary and elements of iPS-NKT manufacturing

1.2.2.2. Standardized donor screen test for iPS-NKT cells (Table 1)

Study specimens will be peripheral blood mononuclear cells (PBMCs) and NKT cells isolated from the blood of healthy individuals who have given written consent. Donor eligibility will be confirmed by physician interview, medical examination, blood tests, hematology, Blood Biochemistry Test, aseptic technique, mycoplasma negative test, as well as tests for specific bacteria and viruses listed in Table 6, in accordance with the Human Cell and Tissue Source Criteria. These specific bacteria and viruses should be re-tested after a window period of 3~4 months to reconfirm that they are negative.

Table 1 List of Donor screening test

Test category	Detail	Method	Tentative Evaluation Standard Values
General status	Medical checkup by physician	Medical interview, medical checkup, and test	Comprehensive judgment (health) based on medical history, blood tests, Blood Biochemistry Test, etc.
	Bacteria/Virus clearance	PCR, Serological, Culture	Sterile, mycoplasma negative, syphilis treponema, HIV, HTLV, HCV, HBV, ParvoB19, EBV, CMV, WNV negative (retest after 3-4 months, window period)
NKT cell characteristics	NKT cell amplification test	Cell culture	Amplified to more than 1 x 10^6 cells by ligand (DC/G) stimulation
Genome analysis	HLA analysis	NSG 法	Detection of HLA-A, -B, -C, -DRB1

1.2.2.3. NKT-iPS cell screen test (Table 2)

The test samples are NKT-iPS cell suspensions after culture. While for cell function measurement, iPS-NKT cells after purified culture (cell counting) and iPS-NKT cells in purified culture on day 33 (d33) and day 43 (d43) after the start of induction culture are used.

Table 2 List of NKT-iPS cell screening and characterization tests

Test category	Detail	Method	Tentative Evaluation Standard Values
Screening tests			
General Characteristics	cell morphology	microscopic observation	Colony formation equivalent to pluripotent stem cells
	Viable cell count	Cell counting	2 x 10^5 or more per frozen tube
	NKT-specific TCR reconstruction	PCR	Confirm the NKT-specific TCR Vα24 gene reconstitution
Cell function	Ability to differentiate into NKT cells	Cell counting	Induction of more than 1 x 10^6 NKT cells per 100 clamps/10 cm dish using OP9 and OP9-DLL1 which passed the specification test. The cells must be able to proliferate under purified culture conditions.
		FACS analysis	(d33) The viable cells must contain CD45+CD3+ cells.<br>(d43)At least 80% of the CD45+ cells express CD3, and CD3+ cells express NKT-specific TCR (Vα24, Vβ11).
Safety	Sterility test	Cell culture	Fungi and common bacteria Negative
	Mycoplasma	PCR	Negative
	Endotoxin	Colorimetric and turbidimetric methods	Less than the standard value (0.3 EU/mL)
	Sendai virus denial	PCR	Negative
Characterization tests			
Pluripotency	Gene expression	Pluripotency marker gene expression	Actb 617-618, Oct 367-368, Sox2 369-370, Klf4 359-360, c-Myc 566-567, Nanog 363-364 Positive
	Cell surface marker expression	Immunostaining	SSEA3, SSEA4, TRA1-60, TRA1-81 Positive

1.2.2.4. NKT-iPS Cell MCB/WCB Standardized Test (Table 3 and 4)

The test samples will be NKT-iPS cell master cell bank (MCB) and working cell bank (WCB) suspensions after culture. Virus testing and characterization studies will be performed only on MCBs. For cell function measurement, iPS-NKT cells after purified culture (cell counting) and iPS-NKT cells in purified culture on day 33 (d33) and day 43 (d43) after the start of induction culture are used.

Table 1 List of the standardization and the characteristic test NKT-iPS cell MCB

<table>
<tr><td colspan="4">Specification test</td></tr>
<tr><td>Test category</td><td>Detail</td><td>Method</td><td>Tentative Evaluation Standard Values</td></tr>
<tr><td rowspan="3">General Characteristics</td><td>Cell Morphology</td><td>Microscopic observation</td><td>Colony formation equivalent to pluripotent stem cells</td></tr>
<tr><td>Total number of cells</td><td>Cell counting</td><td>2 x 10^5 or more per frozen tube</td></tr>
<tr><td>Number of viable cells (or viability)</td><td>Cell/Viability counting</td><td>1.6×10^5 or more per frozen tube during thawing</td></tr>
<tr><td rowspan="2">Cell Function</td><td rowspan="2">Ability to differentiate into NKT cells</td><td>Cell counting</td><td>Induction of more than 1 x 10^6 NKT cells per 100 clamps/10 cm dish using OP9 and OP9-DLL1 which passed the specification test. The cells must be able to proliferate under purified culture conditions.</td></tr>
<tr><td>FACS</td><td>(d33) The viable cells must contain CD45+CD3+ cells.
(d43) At least 80% of the CD45+ cells express CD3, and CD3+ cells express NKT-specific TCR (Vα24, Vβ11).</td></tr>
<tr><td rowspan="13">Safety</td><td>Sterility test</td><td>Cell culture</td><td>Fungi and common bacteria negative</td></tr>
<tr><td>Mycoplasma</td><td>PCR</td><td>Negative</td></tr>
<tr><td>Endotoxin</td><td>Colorimetric and turbidimetric methods</td><td>Less than the standard value (0.3 EU/mL)</td></tr>
<tr><td rowspan="10">Virus denial (only MCB)</td><td>Infectivity test</td><td>Negative</td></tr>
<tr><td>Electron Microscope Test</td><td>Negative</td></tr>
<tr><td>reverse transcriptase activity test</td><td>Negative</td></tr>
<tr><td>Human Virus Testing</td><td>Negative</td></tr>
<tr><td>in vitro test</td><td>Negative</td></tr>
<tr><td>in vivo test</td><td>Negative</td></tr>
<tr><td>Bovine and porcine virus test</td><td>Negative</td></tr>
<tr><td>Mouse Infectivity Test</td><td>Negative</td></tr>
<tr><td>Mouse antibody production test</td><td>Negative</td></tr>
<tr><td>mouse virus test</td><td>Negative</td></tr>
<tr><td colspan="4">Characterization Tests</td></tr>
<tr><td rowspan="2">Pluripotency marker expression</td><td>Gene expression</td><td>PCR</td><td>Actb 617-618, Oct 367-368, Sox2 369-370, Klf4 359-360, c-Myc 566-567, Nanog 363-364 Positive</td></tr>
<tr><td>Cell surface marker expression</td><td>FACS</td><td>SSEA4 TRA-1-81 60</td></tr>
<tr><td rowspan="2">Genome analyses</td><td>karyotype stability</td><td>Chromosomal nuclear stability test</td><td>Normal karyotype is more than 90% and stable after more than 1 month (more than 4 passages)</td></tr>
<tr><td>Exosome (cancer-related gene)</td><td>Deep sequencing of approximately 600 cancer-related gene regions (x1000)</td><td>No genetic mutations in known cancer-related gene regions</td></tr>
</table>

Table 2 List of the specification test for NKT-iPS cell WCB

Specification test			
Test category	Detail	Method	Tentative Evaluation Standard Values
General Characteristics	Cell Morphology	Microscopic observation	Colony formation equivalent to pluripotent stem cells
	Total number of cells	Cell counting	2 x 10^5 or more per frozen tube
	Number of viable cells (or viability)	Cell/Viability counting	1.6×10^5 or more per frozen tube during thawing
Cell Function	Ability to differentiate into NKT cells	Cell counting	Induction of more than 1 x 10^6 NKT cells per 100 clamps/10 cm dish using OP9 and OP9-DLL1 which passed the specification test. The cells must be able to proliferate under purified culture conditions.
		FACS	(d33) The viable cells must contain CD45+CD3+ cells.<br>(d43) At least 80% of the CD45+ cells express CD3, and CD3+ cells express NKT-specific TCR (Vα24, Vβ11).
Safety	Sterility test	Cell culture	Fungi and common bacteria negative
	Mycoplasma	PCR	Negative
	Endotoxin	Colorimetric and turbidimetric methods	Less than the standard value (0.3 EU/mL)

1.2.2.5. iPS-NKT cell (frozen) Specification test (Table Table 3)

The test samples are iPS-NKT cell (frozen) suspensions after culture. Among the specified values, the evaluation of cell functions (in vitro IFNγ productivity and in vitro anti-tumor effect) was conducted on a mini scale compared to the manufacturing of the product, and it is not yet clear whether the above tentative evaluation standard values are applicable to the manufacturing of the product for clinical research. Since it was not possible to test a sufficient amount of lots before the start of the clinical research, the values will be treated as target values or reference values as of December 17, 2021, and further investigation will be conducted through the manufacturing of lots for clinical research to verify whether they can be applied as provisional standard values.

Table 3 List of the standardization and the characteristic tests for iPS-NKT cell (frozen)

Specification test			
Test category	Detail	Method	Tentative Evaluation Standard Values
General characteristic (Day 33)	Viable cell count	Cell counting	2~5×10^6 cells/frozen tube
	Cell surface marker expression	FACS	The viable cells must contain CD45+CD3+ cells.
Safety	Sterility test	Cell culture	Fungi and common bacteria negative
	Mycoplasma	PCR	Negative
	Endotoxin	Colorimetric and turbidimetric methods	Less than the standard value (0.3 EU/mL)
	Cell purity	PCR	Undifferentiated marker (LIN28)-expressing cells negative
Cell function	in vitro IFNγ productivity	ELISA	IFNγ level (target value: 1.7 times the average of negative samples)
	in vitro anti-tumor effect	K562 cell killing potential	Confirmation of anti-tumor efficacy (target: 7%)
Characteristic test			
Cell function	proliferative potential	Living cell counting	Cell proliferation during the purification culture process
	Cell surface marker expression	FACS	CD45+ cells of which CD3+ cells express NKT-specific TCRs (Vα24 and Vβ11)

1.2.2.6. iPS-NKT cell (product) specification test (TableTable 4)

The specimens are iPS-NKT cells (product) after purification culture. Sterility tests were conducted using the rapid method and the Japanese Pharmacopoeia (JP) method (anaerobic bacteria) by sampling the product during manufacturing 2 to 3 days prior to shipment, and were used as shipment judgment tests, while sterility tests using the JP method were also conducted during shipment. The rapid method shown in Table 7 can detect aerobic bacteria and fungi by 48 hours of incubation, so information on sterility can be obtained prior to administration to patients by evaluating samples 2 to 3 days prior to shipment. On the other hand, since anaerobic bacteria can also be tested by the JP method, testing is performed by the JP method. Therefore, before shipment, the shipment of aerobic bacteria, fungi, and anaerobic bacteria is determined by the rapid method and the JP method, and an agreement is made with the study site on what to do if bacteria are detected by the rapid method and the JP method after administration. With regard to cellular function, since there are many variations in interferon γ (IFN-γ) production capacity related to adjuvant effect and direct in vitro anti-tumor effect, and the accuracy of measurement is insufficient as of December 17, 2021, iPS-NKT cells (Therefore, as with iPS-NKT cells (frozen), no reference value will be established, but a target value will be set and verified again after the completion of product manufacturing.)

In addition, since mouse cells are used as feeder cells and FBS is used as a medium additive during differentiation induction, we will measure foreign proteins in the administered formulation after washing.

Table 4 List of specification test on iPS-NKT cell (product)

Specification test			
Test category	Detail	Method	Tentative Evaluation Standard Values
General characteristic	Appearance	Seeing	No adhesion of foreign matter, peeling of labels, etc.<br>No liquid leakage
			No abnormality in color tone and no foreign matter
	Cell morphology	Microscopic observation	T cell-like morphology (floating cell)
	Viable cell count (product)	Cell counting	More than 1×10^{7} cells/50mL tube
	Viability	FACS	More than 90% of viability (7AAD-) in the lymphocyte gate
	Cell surface marker expression	FACS	At least 80% of the CD45+ cells express CD3, and CD3+ cells express NKT-specific TCR (Vα24, Vβ11)
Safety	Cell purity	PCR	Undifferentiated marker (LIN28)-expressing cells negative
	Sterility	Culture method	Fungi and common bacteria negative
		Rapid method	Fungi and common bacteria negative (except anaerobic bacteria)
	Mycoplasma denial	PCR	Negative
	Endotoxin	Colorimetric and turbidimetric methods	Less than the standard value (0.3 EU/mL)
Cell function	in vitro IFNγ productivity	ELISA	IFNγ level (Target value: : 1.7 times the average of negative samples)
	in vitro anti-tumor effect	K562 cell killing potential	Confirmation of anti-tumor effect (Target value: 7%)
Characteristic test			
Safety (impurity)	BSA	ELISA	<50 ng/dose (25 mL) in final product
	Mouse genome	PCR	Below detection limit in final product

Table 5 Detection of test strains by microcolony method

Test strains		Bacteria Amount added (Calculated value)	Culture Medium*	Time									
				12		24		48		72		120	
				Number of Colonies (average)	Recovering rate (%)**	Number of Colonies (average)	Recovering rate (%)	Number of Colonies (average)	Recovering rate (%)	Number of Colonies (average)	Recovering rate (%)	Number of Colonies (average)	Recovering rate (%)
Aerobic bacteria	S.aureus	10 / filter	SCD	3.5	17.5	20.0	**100.0**	20.0	**100.0**	20.0	**100.0**	20.0	100.0
	P.aeruginosa			0.0	NA	25.0	**100.0**	25.0	**100.0**	25.0	**100.0**	25.0	100.0
	B.subtilis			4.5	90.0	5.0	**100.0**	5.0	**100.0**	5.0	**100.0**	5.0	100.0
Anaerobic bacteria	C.sporogenes			0.0	NA	0.0	NA	1.0	33.3	3.0	**100.0**	3.0	100.0
Fungi	C.albicans		SD	0.0	NA	1.0	66.7	1.5	**100.0**	1.5	**100.0**	1.5	100.0
	A.brasiliensis			0.0	NA	0.0	NA	1.0	**100.0**	1.0	**100.0**	1.0	100.0

n=2、32.5℃（S.aureus, P.aeruginosa, B.subtilis, C.sporogenes）、22.5℃（C.albicans, A.brasiliensis）
*SCD：Soybean casein digest agar medium, SD: Sabouraud glucose agar medium
** Recovery rate is relative to the number of colonies after 120 hours as 100%.

1.2.2.7. The stability of iPS-NKT cell (product) (Table Table 6)

After the purification culture process, the product is suspended in the culture medium and shipped. However, the number of viable cells and activity of the product decline over time due to aggregation by sedimentation and inactivation of cells, so the stable period after shipment is short and long-term stability cannot be guaranteed. Therefore, in order to confirm the extent to which this product can be used after being shipped to a study site, an experiment was conducted assuming that the product is first shipped to the study site while in culture and then stored in a CO_2 incubator at the site. In this experiment, after the product was shipped, it was cultured in a CO_2 incubator in the same way as it was cultured in a roller culture device, or it was left in the incubator, and the number of viable cells, in vitro IFNγ productivity, and in vitro anti-tumor effect were measured the day after shipment to determine whether the product could be used. The purity of the cells was not evaluated in this experiment because it has been confirmed that there is no change in the purity of the cells even if the incubation period is extended in previous studies. The sterility, mycoplasma negativity, and endotoxin tests were not performed in this experiment because the containers were not opened and there was no risk of contamination during shipment. The impurities were not conducted in this experiment because they are to be measured at the point of administration and validation was conducted in another washing test.

As a result, as shown in Table 9, 24 hours after shipment, the number of viable cells was below the standard value, although cell function was maintained, and it was determined that use on the day after delivery was not desirable. Therefore, we estimated the time required to transport the cells from the production facility (RIKEN) to the study site (Chiba University) and to dispense them by means of a transport test and found that 3 hours was sufficient. The estimated time from arrival of the product at Chiba University to administration was also found to be sufficient, with an estimated time of 3 hours. Therefore, a stability study of the product and the post-dispensing formulation was conducted, assuming 3 hours for transportation and dispensing of the product and 3 hours for storage of the post-dispensing formulation. This experiment was conducted to determine whether the product could be stored in a transport box at 37°C under a CO_2 supply and dispensed with a dosage formulation after 3 hours to obtain a post-dispensing formulation at the dosage concentration in the study and whether the post-dispensing formulation would retain stability for the period required for administration. The cell count of the post-dispensing formulation was calculated as the number of cells per 50 mL from the cell count (2×10^6 cells/mL) and volume after dispensing.

As shown in Table 10, the cell functionality of a post-dispensing formulation was maintained 3 hours after shipment of the product with a target concentration (2.0×10^6 cells/mL). It was also clear that cell function was maintained to some extent at both 4°C and 25°C at 3 hours after dispensing, but that the number of viable cells was insufficient in some samples when stored at 25°C, and that the in vitro antitumor activity may be below the target value. Therefore, we determined that 4°C was the preferable storage condition after preparation.

Based on these results, the stability of this product was determined to be within 6 hours after shipment and within 3 hours after dispensing. The storage temperature was set at 4°C (3°C ± 2°C).

Table 6 The stability of the product (under CO_2 incubator)

Standardized test				Stability test (CO_2 provided, 37°C)					
Test category	Detail	Method	Tentative evaluation standard value	At the point of shipment		24 hr (2hrs after shipment, roller culture)		24 hr (2hrs after shipment, left in the incubator)	
				Lot.1	Lot.2	Lot.1	Lot.2	Lot.1	Lot.2
General characteristic	Appearance	Seeing	No adhesion of foreign matter, peeling of labels, etc. No liquid leakage	No label because of internal test	No label because of internal test	No label because of internal test	No label because of internal test	No label because of internal test	No label because of internal test
			No abnormality in color tone and no foreign matter	NA	NA	NA	NA	NA	NA
	Cell morphology	Microscopic observation	T cell-like morphology (floating cell)	T-cell type	T-cell type	T-cell type	T-cell type	T-cell type	T-cell type
	Viable cell count	Cell counting	More than $1×10^7$ cells/50mL tube	$3.5×10^7$	$3.6×10^7$	$0.22×10^7$	$0.19×10^7$	$0.49×10^7$	$0.56×10^7$
	Viability	FACS	More than 90% of viability (7AAD-) in the lymphocyte gate	99.9%	Not conducted*1	99.7%	Not conducted *1	92.1%	Not conducted *1
	Cell surface marker expression	FACS	At least 80% of the CD45+ cells express CD3, and CD3+ cells express NKT-specific TCR (Vα24, Vβ11)	92.9%	Not conducted*1	94.9%	Not conducted *1	93.0%	Not conducted *1
Cell function	in vitro IFNγ productivity	ELISA	IFNγ level (Target value: : 1.7 times the average of negative samples)	8143pg/mL 9.42 times	5953 pg/mL 17.54 times	3305 pg/mL 6.09 times	6726 pg/mL 20.37 times	1366 pg/mL 4.88 times	6712 pg/mL 7.92 times
	in vitro anti-tumor effect	K562cell-killing potential	Confirmation of anti-tumor effect (Target value: 7%)	31.6%	23.1%	17.0%	26.0%	10.7%	20.2%

*1: Not conducted because of malfunction of equipment (FACS)

Table 7 Stability of the product and the post-dispensing formulation

Standardized test				Stability test					
Test category	Detail	Method	Tentative Evaluation Standard Values	出荷時(本品)			3hr(調剤後製剤)		
				Lot.2	Lot.1	Lot.3	Lot.2	Lot.1	Lot.3
General condition	Appearance	Seeing	No adhesion of foreign matter, peeling of labels, etc. No liquid leakage	No label because of internal test			No label because of internal test		
			No abnormality in color tone and no foreign matter	NA			NA		
	Cell morphology	Microscopic observation	T cell-like morphology (floating cell)	T cell type			T cell type		
	Viable cell count (product)	Cell counting	More than $1×10^7$ cells/50mL tube	$3.6×10^7$	$3.5×10^7$	$2.1×10^7$	$3.0×10^7$	$3.0×10^7$	$1.5×10^7$
	Viability	FACS	More than 90% of viability (7AAD-) in the lymphocyte gate	99.9	Not conducted *1	99.2	99.9	Not conducted *1	97.6
	Cell surface marker expression	FACS	At least 80% of the CD45+ cells express CD3, and CD3+ cells express NKT-specific TCR (Vα24, Vβ11)	92.9	Not conducted *1	88.1	91.1	Not conducted *1	96.3
Cell function	in vitro IFNγ productivity	ELISA	IFNγ level (Target value: : 1.7 times the average of negative samples)	5953 pg/mL 17.54 times	8143 pg/mL 9.42 times	11289 pg/mL 29.46 times	1663 pg/mL 9.76 times	3174 pg/mL 13.35 times	3784 pg/mL 9.93 times
	in vitro anti-	K562 cell	Confirmation of anti-tumor	23.1%	31.6%	19.4%	20.1%	12.0%	22.7%

	tumor effect	killing potential	effect (Target value: 7%)						

*1: Not conducted because of malfunction of equipment (FACS)

Table 8 Stability of the post-dispensing formulation

Standardized test				Stability test														
Category	Detail	Method	Tentative evaluation standard value	At the point of adjustment (3hrs after shipment)			3 hr (6hrs after shipment)						21 hr (24hrs after shipment)					
							4°C			25°C			4°C			25°C		
				Lot.2	Lot.1	Lot.3	Lot.2	Lot.1	Lot.3	Lot.2	Lot.1	Lot.3	Lot.2	Lot.1	Lot.3	Lot.2	Lot.1	Lot.3
General characteristic	Appearance	Seeing	No adhesion of foreign matter, peeling of labels, etc. No liquid leakage	No label because of internal test														
			No abnormality in color tone and no foreign matter	NA														
	Cell morphology	Microscopic observation	T cell-like morphology (floating cell)	T cell type														
	Viable cell count	Cell counting	More than 1×10^7 cells/50 mL tube	3.0×10^7	3.0×10^7	1.5×10^7	1.4×10^7	1.5×10^7	1.0×10^7	1.0×10^7	1.2×10^7	0.9×10^7	1.1×10^7	1.0×10^7	0.76×10^7	0.86×10^7	0.52×10^7	0.5×10^7
	Viability	FACS	More than 90% of viability (7AAD-) in the lymphocyte gate	99.9	Not conducted*1	97.6	99.3	Not conducted*1	99.3	99.5	Not conducted*1	99.2	99.3	Not conducted*1	Not conducted*1	96.1	Not conducted*1	Not conducted*1
	Cell surface marker expression	FACS	At least 80% of the CD45+ cells express CD3, and CD3+ cells express NKT-specific TCR	91.1	Not conducted*1	96.3	90.4	Not conducted*1	94.7	90.2	Not conducted*1	95.6	89.5	Not conducted*1	Not conducted*1	92.0	Not conducted*1	Not conducted*1

			(Vα24, Vβ11)															
Cell function	in vitro IFNγ productivity	ELISA	IFNγ level (Target value: : 1.7 times the average of negative samples)	1663 pg/mL 9.76倍	3174 pg/mL 13.35倍	3784 pg/mL 9.93倍	1099 pg/mL 17.31倍	3192 pg/mL 18.73倍	1786 pg/mL 7.54倍	793 pg/mL 19.20倍	3670 pg/mL 17.01倍	2185 pg/mL 9.49倍	1116 pg/mL 3.36倍	1019 pg/mL 5.82倍	1047 pg/mL 11.29倍	108 pg/mL 2.24倍	684 pg/mL 9.84倍	317 pg/mL 9.66倍
	in vitro anti-tumor effect	K562cell-killing potential	Confirmation of anti-tumor effect (Target value: 7%)	20.1%	12.0%	22.7%	9.5%	11.2%	13.9%	7.6%	2.9%	6.9%	13.6%	0%	3.5%	0%	0%	0%

*1: Not conducted because of malfunction of equipment (FACS)

1.2.2.8. Auto-DC/Gal manufacturing

Outline each step of the manufacturing process and indicate when specification tests are conducted at each stage.

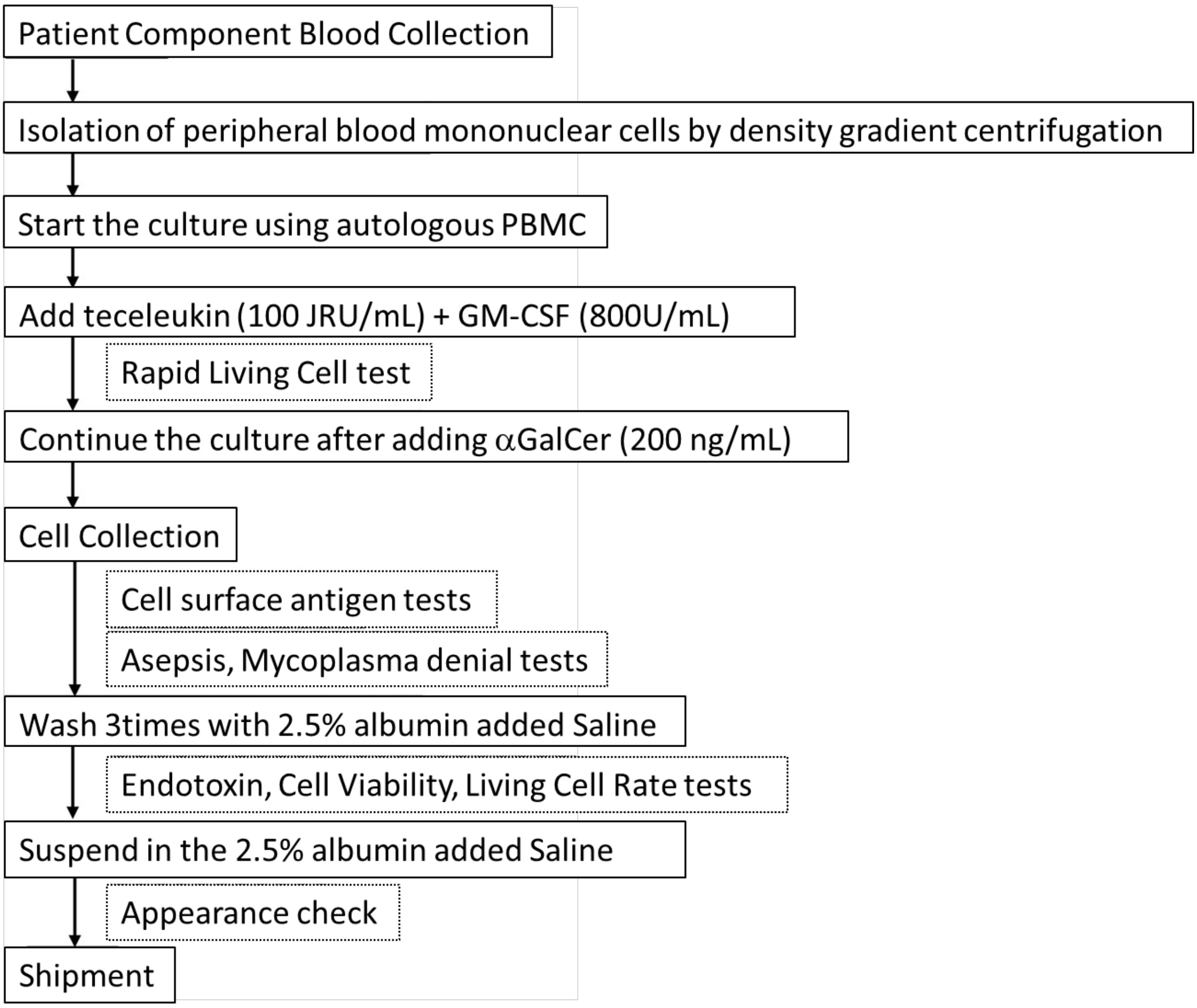

Fig 3 Diagram of DC/Gal manufacturing

1.2.2.9. Specification test on the auto-DC/Gal

The following is a list of inspections that are performed at the time of manufacture and shipment of DC/Gal and the timing of these inspections. Table 11 shows the test items and their specified values/judgment criteria.

(1) Living cell count: at the time of initial blood collection of mononuclear cell fraction and before administration

(2) Cell viability: before administration

(3) Endotoxin (simple method): at shipping on the day of administration

(4) Cell surface antigen assay (FACS): at the time of shipment on the day of administration

(5) Inspection of foreign matter in formulation: at the time of manufacture and at the time of shipment

(6) Inspection of the appearance of the container of the drug product: At the time of shipment (direct confirmation of the container and packaging)

(7) Rapid viable bacterial count test: submitted for inspection 3 days prior to administration

(8) Endotoxin test, sterility test, mycoplasma negativity test: To be submitted for inspection on the day of administration. If a positive test result is found after cell administration, the responsible person should be notified immediately, and strict follow-up should be conducted regarding the patient in question.

Table 9 List of specification tests on auto-DC/Gal

Test category	Method	Specified value/judgement criteria
Living cell count	Measurement using a blood cell counting plate	More than 1×10^8
Cell viability	Measured using a trypan blue stained blood cell calculator	More than 60%
Rapid viable bacterial test	Membrane filter method/rapid fluorescent staining	Negative
Sterility test	Membrane filter method	Negative
Endotoxin test	Gelation method/limit test method	< 0.25 EU/mL
Mycoplasma denial test	NAT Method	Negative
Endotoxin test (simplified test)	kinetic colorimetry (Simplified method)	< 1 EU/mL
Cell surface antigen assay	FACS	Of CD45+ cells, at least 15% of CD86+ cells
Impurity test on product	Macro- and Microscopic observation	No foreign matter
Appearance test on product container	Seeing	No abnormalities on the container and package

1.2.2.10. Stability test on auto-DC/Gal

After a 7-day incubation process, the product is shipped in suspension in saline with human albumin for rapid nasal submucosal administration. Due to the significantly high cell concentration at the time of shipment, it is expected that the number of viable cells will decrease over time, and long-term stability cannot be guaranteed. On the other hand, the time required for cell transportation and administration from the cell manufacturing facility (Chiba University Hospital Center for Advanced Medicine) to the administration site (Chiba University Hospital Otorhinolaryngology-Head and Neck Surgery Unit) is estimated to be 1 hour. Therefore, in order to confirm how long the product can be used after being left at room temperature after shipment, we measured changes over time in cell count and cell surface antigen when the product was left at room temperature (around 20~25℃ in Chiba University Hospital) under the condition of 1×10^8 cells/200 μ L, which is the cell concentration at the time of shipment.

As a result, as shown in Table 12, the cell count at the time of shipment (1.0×10^8 cells/200 μL) was obtained even 4 hours after shipment of this product, while maintaining the cell surface antigen.

Based on these results, the stability of this product was determined to be within 2 hours after shipment, ensuring a safe range. The preferable temperature for shipment was set at room temperature.

Table 10 Stability of post-dispensing formulation

Specification test				Stability test											
Category	Detail	Method	Standardized value	At the time of arrangement		1h		2h		3h		4h		24h	
				Lot.1	Lot.2	Lot.1	Lot.2	Lot.1	Lot.2	Lot.1	Lot.2	Lot.1	Lot.2	Lot.1	Lot.2
General characteristic	Living cell count	Cell counting	1×10^8 /200μL	1.0×10^8	1.0×10^8	1.0×10^8	9.1×10^7	9.2×10^7	7.6×10^7	1.3×10^8	7.5×10^7	1.1×10^8	7.5×10^7	n.d.	1.6×10^7
	Viability	Cell counting	Trypan blue unstained cells >60%.	93.0	95.8	93.5	98.9	87.8	98.1	95.6	97.7	92.3	99.[illegible]	n.d.	20.9
	Cell surface marker expression	FACS	Of CD45+ cells, at least 15% of CD86+ cells	n.d.	51.0	n.d.	60.3	n.d.	64.7	n.d.	66.6	n.d.	68.3	n.d.	67.2

1.2.3. Efficacy on preclinical study

1.2.3.1. Anti-tumor effect of iPS-NKT cell (K562 cell)

To confirm the anti-tumor effect of iPS-NKT cells, K562-luc cells, a cell line derived from human leukemia and highly sensitive to NK cells, were inoculated into the abdominal cavity of NOG mice, hyper immunodeficient mice. After 5 days, 3×10^6 iPS-NKT cells were injected intraperitoneally every 2 days until day 13, and the proliferation of K562-luc cells was observed from outside the body by fluorescence. As shown in Figure 4, the inhibition of K562-luc cell proliferation was observed in mice treated with iPS-NKT cells.

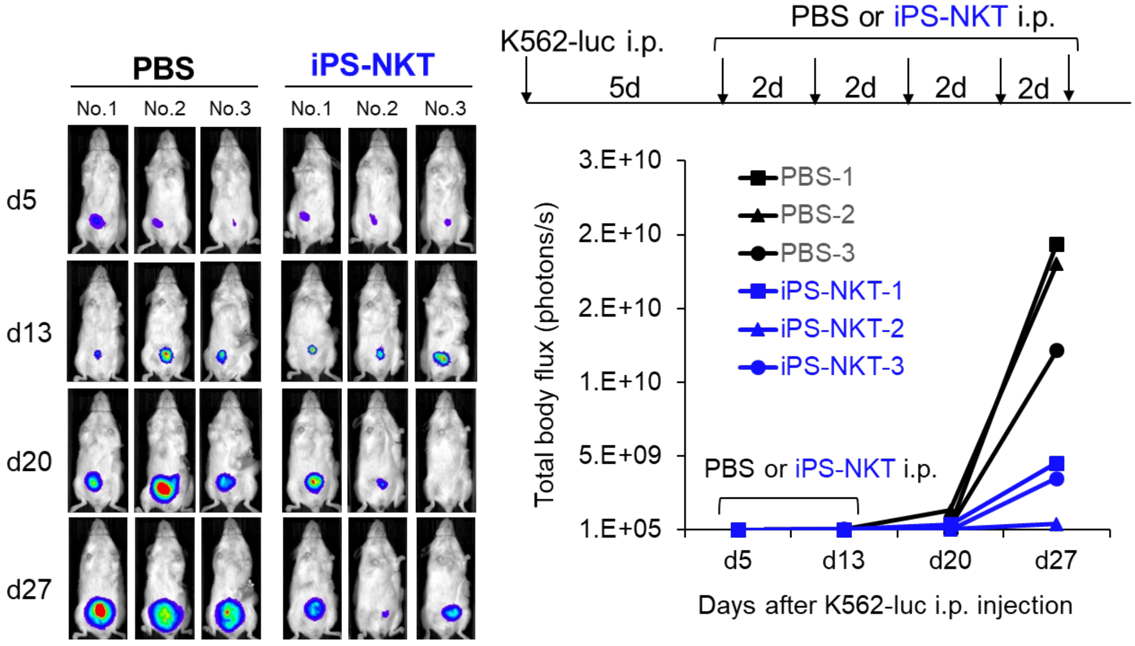

Figure 4 Anti-tumor effect of iPS-NKT cell against K-562-luc cell

1.2.3.2. Anti-tumor effect of iPS-NKT cell against (FaDu cell)

To confirm the anti-tumor effect of iPS-NKT cells, 5×10^5 FaDu-luc cells, in which the luciferase gene was introduced into FaDu cells, a cell line derived from human head and neck cancer, were inoculated under the skin of NSG mice, hyper immunodeficient mice. The tumor size on day 13 was set as 1, and the subsequent changes in tumor size were observed. As shown in Figure 5, the inhibition of tumor growth was observed in mice treated with iPS-NKT cells.

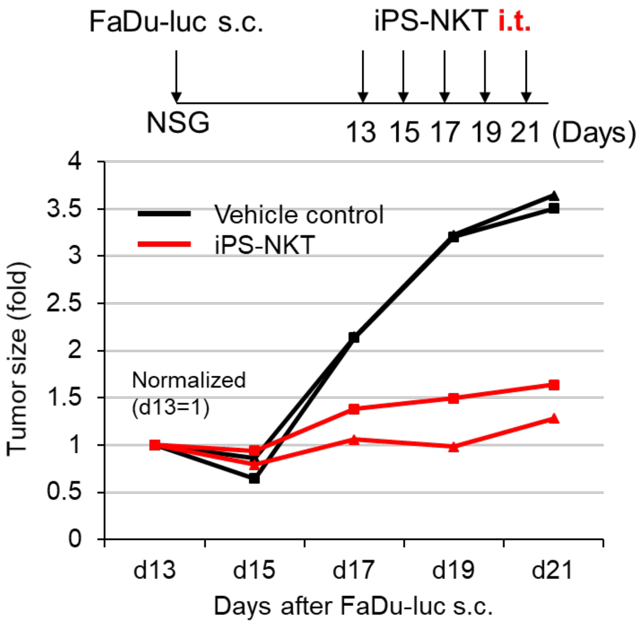

Figure 5 Anti-tumor effect of iPS-NKT cell against FaDu-luc cell

1.2.3.3. NK cell activation potential of iPS-NKT cells (adjuvant activity)

To confirm whether iPS-NKT cells can activate human NK cells, iPS-NKT cells were administered together with DC/Gal to hyper immunodeficient mice (NOG mice) that had been treated with human peripheral blood mononuclear cells, and NK cell activation was observed. As shown in Figure 6, the activation of human NK cells was observed only when iPS-NKT cells were administered with DC/Gal to NOG mice that had been treated with peripheral blood mononuclear cells, confirming NK cell activation (adjuvant activity) by iPS-NKT cells.

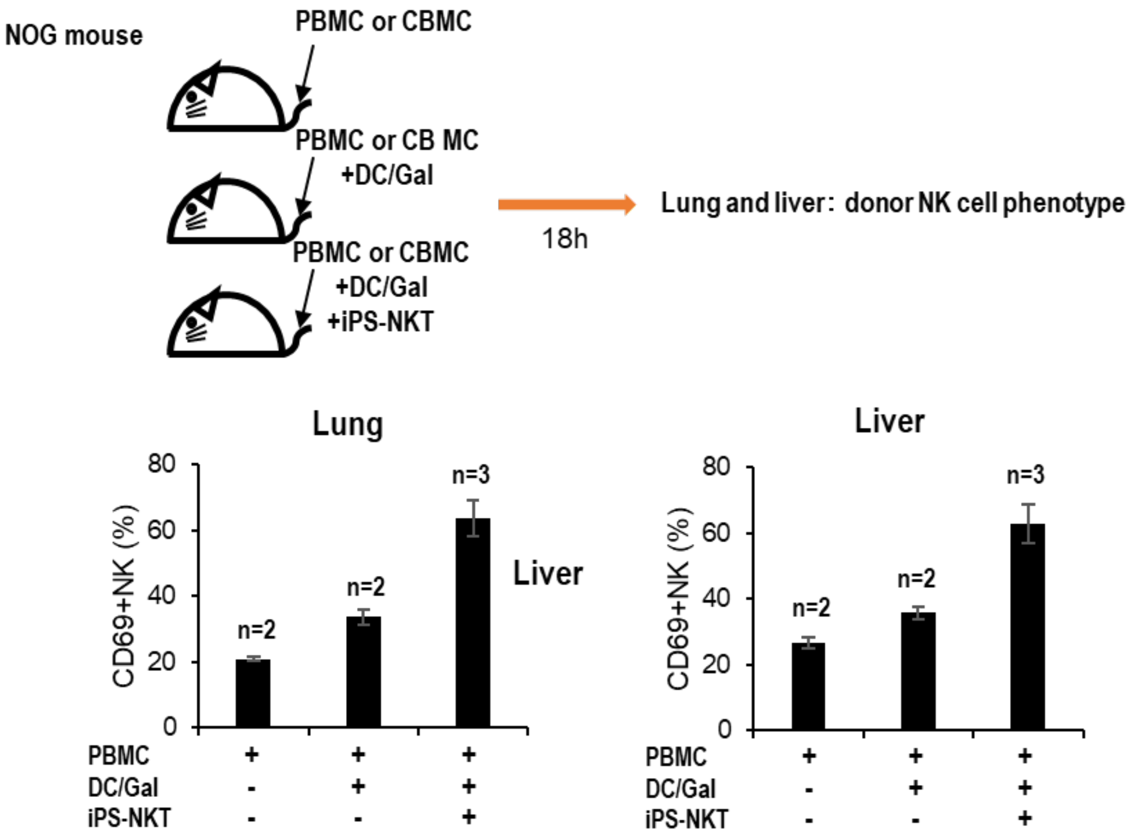

Figure 6 NK cell activation potential of iPS-NKT cells

1.2.3.4. Combination of iPS-NKT cell and DC/Gal

To investigate whether the combination of iPS-NKT cells and DC/Gal enhances the in vivo antitumor effect, cancer cells derived from human lung cancer patients were inoculated subcutaneously (PDX) in human IL7/15 knock-in NSG mice, and iPS-NKT cells 3× 10^6 cells (1 × 10^8 cells/kg) were injected into the tumor every 2 days from day 0 to day 8 (green arrow). In the combination treatment group, 1×10^6 mouse DC/Gal cells (3.3×10^7 cells/kg) were similarly administered into the tumor in addition to iPS-NKT cells on the 8th day after the start of treatment (red arrow). As shown in Fig. 7, inhibition of tumor growth was observed in mice treated with iPS-NKT cells and DC/Gal. The expression of cytotoxic factors was observed on days 8, 9, and 10 after the start of treatment, and the expression

of cytotoxic molecules such as FASLG, GZMA, and TNF, as well as genes related to adjuvant effect such as GNLY, CCL3, and CCL4 were enhanced (Fig. 7). This confirmed that the iPS-NKT+DC/Gal combination group showed significant anti-tumor effects compared to the iPS-NKT alone group.

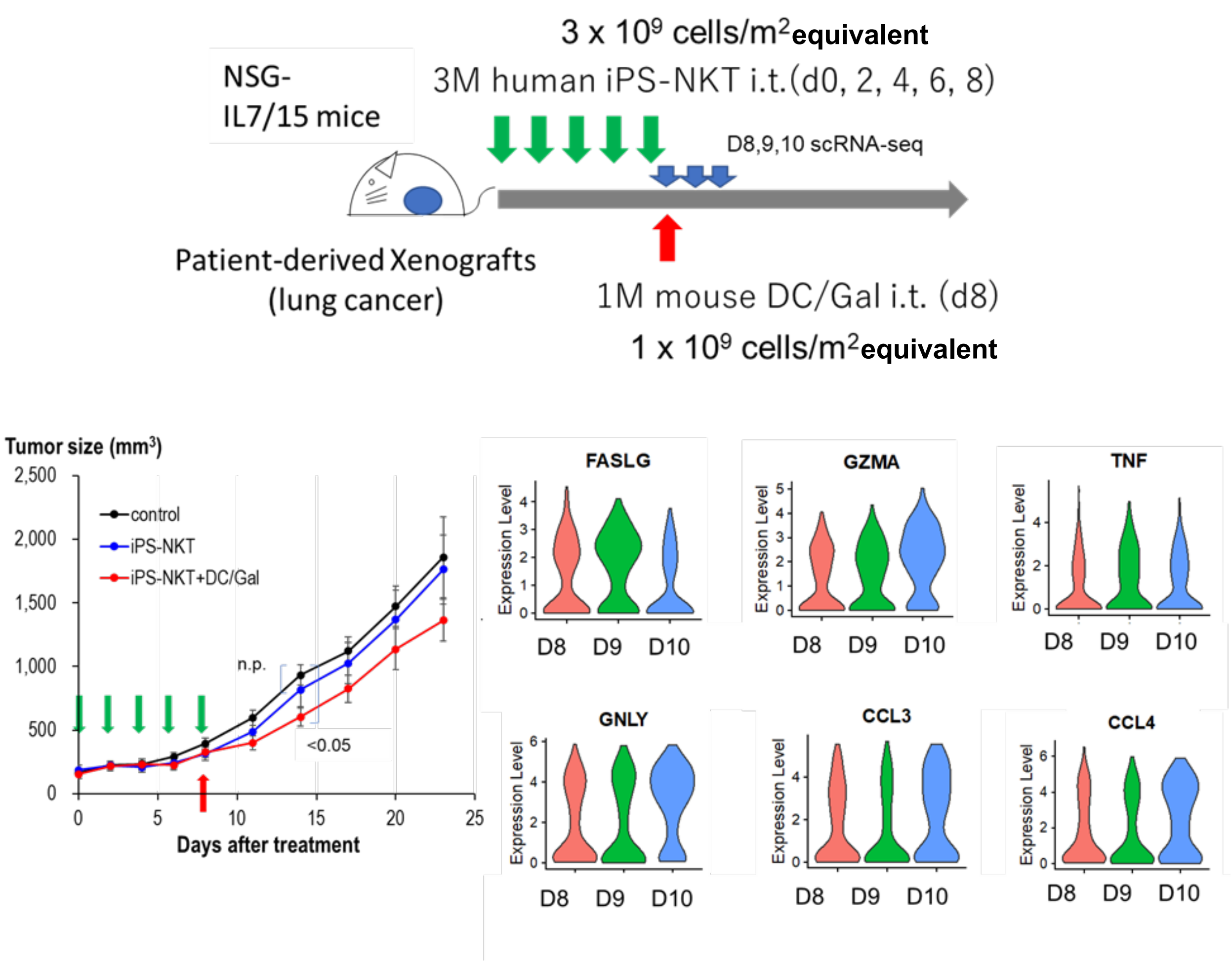

Figure 7 Anti-tumor effect of iPS-NKT cells and DC/Gal combination on human lung cancer PDX

1.2.3.5. In vivo anti-tumor effect and intra-tumor immune cell analysis (DC/Gal combination, human lung cancer PDX)

In order to confirm how the combination of iPS-NKT cells and DC/Gal exerts anti-tumor effects in vivo, we investigated in a model mouse in which the involvement of human immune cell population can also be evaluated. Cancer cells derived from human lung cancer patients were inoculated subcutaneously into human IL7/15 knock-in NSG mice (PDX), and human peripheral blood mononuclear cells (PBMCs) were injected into the tumor on day 0. For mice with confirmed tumor growth, 3 x 10^6 iPS-NKT cells (1 x 10^8 cells/kg) were administered into the tumor on day 1. In the combination group, 1 x 10^6 mouse DC/Gal cells (3.3 x 10^7 cells/kg) were administered into the tumor on the same day (day 1) in addition to iPS-NKT cells (Fig. 8). As a result, inhibition of tumor growth was observed only in mice treated with iPS-NKT cells and DC/Gal in addition to PBMCs (Fig. 8, right bottom). The analysis of immune cells remaining in the tumor on day 14 showed that most of them were T cells, which was thought to be due

to the adjuvant effect induced by the combined administration of iPS-NKT cells and DC/Gal (Fig. 8, left bottom). This confirmed that the iPS-NKT+DC/Gal combination group showed significant anti-tumor effects compared to the iPS-NKT alone group.

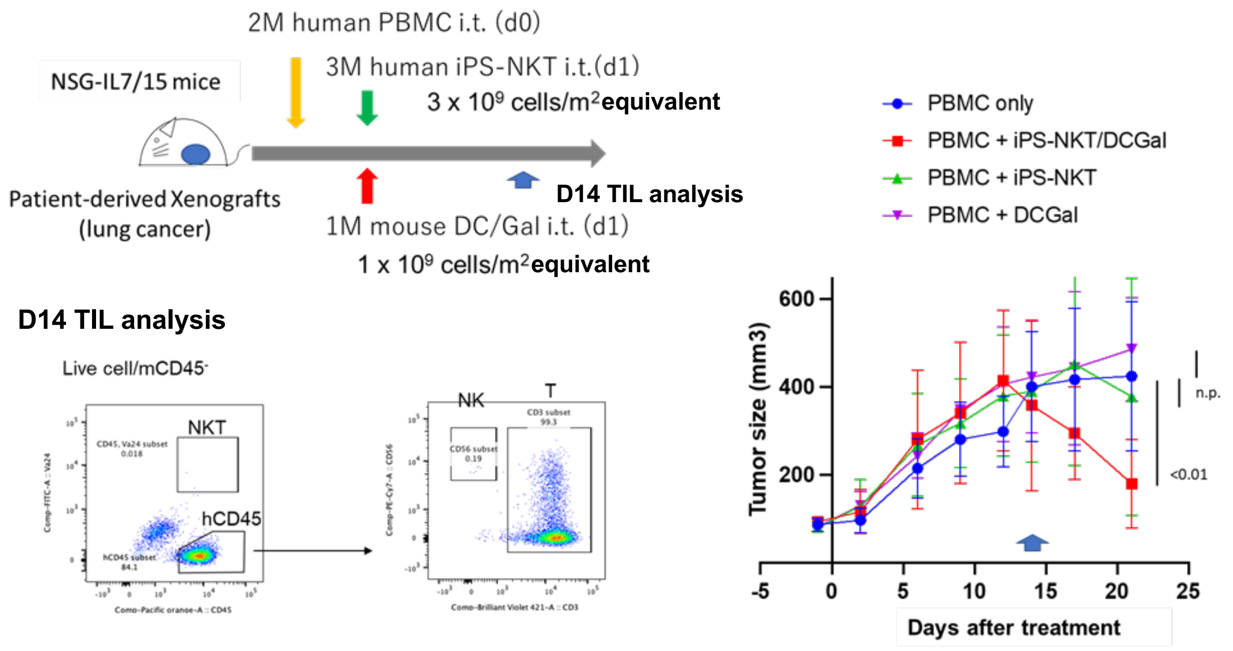

Figure 8 Anti-tumor effect of PBMC, iPS-NKT cells and DC/Gal combination treatment on human lung cancer PDX

1.2.4. General toxicity and tumorigenicity studies

1.2.4.1. General toxicity test on iPS-NKT cell

In the trial for iPS-NKT cells alone, we planned to administer iPS-NKT cells at a dose of 1×10^7 to 1×10^8 cells/m^2 via the tumor feeding artery of HNSCC three times biweekly. In this trial of iPS-NKT cell and DC/Gal combination administration, the iPS-NKT cell dose is also planned to be 1×10^8 cells/m^2 at the maximum. The tumor feeding artery administration is expected to distribute iPS-NKT cells to tumors in the head and neck region, where capillaries are well developed. Although the tumor feeding artery of the tumor cannot be selected as the route of administration for the toxicity study in mice, iPS-NKT cells administered intravenously in the preliminary kinetic study were distributed in the liver, kidney, and spleen, where capillaries are well developed, and it is expected that cells not retained in the tumor will not be retained in the tumor feeding artery administration in the head and neck tumor. Based on these considerations, intravenous administration was selected as the route of administration to mice, and the dosage was set at 1×10^7 to 1×10^9 cells/m^2, which is up to 10 times the clinical dosage. This dose per human body surface area was converted to a dose per mouse body weight of 3.3×10^6 cells/kg to 3.3×10^8 cells/kg, which is close to the limit of the dose for mice that was discussed with the contract research organization, so we considered this dose to be appropriate for the non-clinical safety study. Therefore, we conducted a single-dose toxicity study using 3.3×10^8 cells/kg as the maximum dose, and found no deaths, and no abnormalities in general condition, general symptoms and behavioral observation using Irwin's variant method, body weight, food intake, hematological

examination, blood biochemical examination, urinalysis, necropsy, organ weight, and histopathological examination. Therefore, the maximum non-toxic dose of iPS-NKT cells was considered to be 3.3×10^8 cells/kg for both males and females.

Since iPS-NKT cells were administered only once in this clinical study, a repeated dose toxicity study was not conducted.

1.2.4.2. General toxicity test on DC/Gal

A single nasal submucosal administration of 1.0×10^8 cells/body of DC/Gal to humans is planned. Since nasal submucosal administration of the DC/Gal is technically difficult in mice, we decided to investigate the toxicity when administered systemically via intravenous route. The α-galactosylceramide-pulsed dendritic cells (DC/Gal) were obtained from peripheral blood mononuclear cells derived from component blood samples of healthy human blood donors, cultured in medium supplemented with IL-2 and GM-CSF for 1 week and 2 weeks, respectively. The cells were administered intravenously to male and female nude rats (F344/NJcl-rnu/ rnu) at 21 to 23 hours after shipping and evaluated for subacute and sub-chronic systemic toxicity. The administered dose was about $8.5 \times 10^7 \sim 1.7 \times 10^8$ cells/body, which corresponds to more than 50 times the number of administered cells expected in clinical practice and was converted to the body surface equivalent according to the approximate body weight gain, and the number of administered cells was determined. As a result, no changes related to the DC/Gal administration were observed in the general condition, body weight, food intake, ophthalmological examination, urinalysis, hematological examination, blood chemical examination, necropsy findings, organ weights, and histopathological examination in both male and female DC/Gal groups at 4 and 13 weeks of necropsy. Based on the above results, it was concluded that α-galactosylceramide pulsed dendritic cells were not toxic under the conditions of this clinical trial.

1.2.4.3. Toxicity study of combination dose (NKT-deficient B6, human IL7/15 expressing NSG mice)

To evaluate the effects on host immune cells, iPS-NKT cells were administered at doses of $1 \sim 2 \times 10^6$ cells/mouse ($0.5\text{-}1 \times 10^8$ cells/kg) and mouse DC/Gal at 1×10^6 cells/mouse (0.5×10^8 cells/kg) to NKT-deficientB6 (Ja18KO) mice intravenously three times every 2 weeks (day 0, 14, and 28) and then observed until day 42. Control groups were administered iPS-NKT cells alone and DC/Gal (derived from wild-type B6 mice) alone. No animal deaths were observed in all treatment groups throughout the treatment period, and the iPS-NKT cells alone and the DC/Gal in combination group didn't show higher levels of the proinflammatory cytokines interferon γ, IL-6, IL-64, and IL-64 than the wild-type B6 mice DC/Gal alone group.

To evaluate the presence of graft-versus-host disease by iPS-NKT cells, iPS-NKT cells were administered to human IL7/15-expressing NSG mice, which can maintain human NKT cells for a long time, at a dose of 2×10^6 cells/mouse (1×10^8 cells/kg) and mouse DC/Gal at 1×10^6 cells/mouse (0.5×10^8 cells/kg), and observed from the first administration to day 70. The control groups were administered iPS-NKT cells alone, DC/Gal alone, and T cells alone. The iPS-NKT cells alone group and DC/Gal in combination group showed no drug-induced toxicity changes in general condition, body weight, autopsy, or histological examination.

Based on the results above, the non-toxic dose when iPS-NKT cells and DC/Gal were administered together was estimated to be greater than 2×10^6 cells/mouse (1×10^8 cells/kg).

1.2.4.4. Tumorigenic test

As for the tumorigenicity test, the soft agar colony test was first conducted as an in vitro test, but no proliferation was observed. In addition, regarding genomic findings of concern for tumorigenicity, karyotyping, tumor-related genes (COSMIC CGC Tier 1 and Sibata list), and structural abnormalities (abnormal copy number) were examined in the raw material NKT-iPS cells and iPS-NKT cells produced from them, but no abnormalities were found in any of the genomic findings. Since human iPS-NKT cells cannot be maintained in mice with normal immune function due to rejection, NOG mice, which are highly immunodeficient animals and have been used for drug efficacy studies, were used for the in vivo tumorigenicity study. Tumors derived from iPS-NKT cell preparations that would form in mice may include teratomas derived from undifferentiated iPS cells that remain in iPS-NKT cells and tumors derived from tumor cells contained in iPS-NKT cells or transformed by iPS-NKT cells, so we used two types of iPS cells and iPS-NKTcell preparations for the tests. As for the route of administration, iPS-NKT cells are relatively small cells and accumulate in the liver and kidney after intravenous administration, so intravenous administration was used to confirm tumorigenicity in these organs.

The iPS cells in the iPS-NKT cell preparation can detect 0.01% contamination by measuring LIN28, an undifferentiated marker. This indicates that at the maximum expected clinical dose of 1×10^8 cells/m^2 (3.3×10^7 cells/kg of body weight in mice), 1×10^4 cells/m^2 (3.3×10^3 cells/kg of body weight in mice) of iPS cells may be present. Therefore, the amount of iPS cells that are expected to be contaminated is 1×10^4 cells/m^2. Therefore, if teratomas were not formed after iPS cells administration in larger doses than expected, it could be proved that teratomas were not formed with the amount of iPS cells that may be present in the iPS-NKT cell preparation. Therefore, we set the dose as 3.3×10^8 cells/kg, which was confirmed safe in a preliminary single-dose toxicity study of iPS-NKT cells. Since the dose was 100,000 times the expected contamination dose, the observation period was 12 weeks in accordance with WHO TRS-878 section B.2.3.7. As a result, no tumorigenicity was observed in the NKT-iPS cells and no residual human cells were observed. Therefore, it was proved that no teratoma was formed with the amount of iPS cells that may be mixed in the iPS-NKT cell preparation. Additionally, based on the face-to-face meeting with PMDA (held on March 1, 2017, Re- Strategic consultation #P61-2), the same study was conducted on the newly established test iPS cell line (HV#2), and the results showed no evidence of tumorigenicity or residual human cells. Therefore, we believe that teratomas do not form with the amount of iPS cells that may be present in the iPS-NKT cell preparation.

Regarding the tumorigenicity of malignant transformed cells derived from iPS-NKT cells, it is required to administer as many cells as possible and observe them for as long as possible. Therefore, we set the dose at 3.3×10^8 cells/kg, which was confirmed to be safe in a preliminary single-dose toxicity study of iPS-NKT cells. However, some contract research organization have conducted long-term rearing of NOG mice for up to one year, and we have information that only one mouse per 20 mice died at the end of the long-term rearing period, while the other mice were healthy. Therefore, we assumed the proper observation period to be 1 year. The number of animals used in the

study was 14 mice per arm, with one additional animal added, because the expected minimum survival rate at the 99.9% confidence interval was calculated from the survival rate after one year, and it was expected that the use of 13 or more mice per arm would ensure the survival of at least 10 mice after one year. As a result, no tumorigenic changes attributable to the test substance administration were observed in all animals (including dead/near-death necropsied animals) at the histopathological examination after 52 weeks of treatment. In addition, anti-human nuclear antibody-positive cells were found in the lungs of one male iPS-NKT-treated animal, but no cells that tested positive for anti-Ki-67 antibody were found. Therefore, we concluded that 3.3 x 10^8 cells/kg of human iPS-NKT cells had no tumorigenic potential.

Since the indication for iPS-NKT cells is cancer, oncogenicity, genotoxicity, and reproductive toxicity studies will not be conducted. Also, since the plan is to administer iPS-NKT cells into the nutrient arteries of cancer, local irritation tests will not be conducted.

A summary of the toxicity and tumorigenicity studies conducted to date is shown in Table 13.

Table 11 Summary of non-clinical safety test

Category	Test substances	Subjects	Dosing and Method	Results	Status	Miscellaneous
single-dose toxicity study (Preliminary examination)	Human iPS-NKT	Nude mice (BALB/c-nu)<br>6 weeks old<br>Group composition<br>5 each male and female animals/group x 3 groups (media control group, 2 doses of test substance group)<br>Observation period: 15 days	single round<br>1×10^6 ,<br>1×10^7 cells/kg, intravenous administration	No deaths, no abnormalities in general condition, weight, hematology, blood chemistry, organ weights and autopsy	end	Non-GLP<br>[Ref. iii.].
	Human iPS-NKT	Mouse (BALB/c)<br>6 weeks old<br>Group composition<br>3 females/group x 5 groups of 3 males each<br>(media control group, 3 doses of test substance group)<br>Observation period: 8 days	single round<br>3.3 x 10^6 ,<br>3.3 x 10^7 ,<br>3.3×10^8 cells/kg intravenous administration (60-70 seconds/animal)	No fatalities.<br>General condition, weight, and autopsy were normal.	end	Non-GLP<br>[Ref. iv.].
single-dose toxicity study (Expansion type, core battery test built-in)	Human iPS-NKT preparation	Mouse (NOG)<br>6 weeks old<br>Group composition<br>10 females/group x 4 groups<br>(media control group, 3 doses of test substance group)<br>Observation period: 15 days	single round<br>3.3 x 10^6 ,<br>3.3 x 10^7 ,<br>3.3×10^8 cells/kg intravenous administration	No deaths, no abnormalities in general condition, general symptoms and behavioral observations using Irwin's variant, body weight, food intake, hematology, blood biochemistry, urinalysis, autopsy, organ weights and histopathology	end	Regenerative Medicine Products GLP<br>[Ref. v.].
Toxicity test on repetitive administration	Human DC/Gal	nude rat<br>(F344/NJcl-*rnu*/ *rnu*)	8.5×10^7 ~ 1.7×10^8 /body<br>Intravenous administration on day 0, 7, 42, 49	No abnormal sign on general status, body weight, feeding, ophthalmological test, urinalysis, blood test, Blood Biochemistry Test, autopsy findings, organ weight, and pathological assessment	end	Regenerative Medicine GLP<br>[Ref. vi.].

Toxicity test on combination administration	Human iPS-NKT<br>Mice DC/Gal	NKT-deficient B6 (Ja18KO)	1×10^9,<br>2×10^9 cells/m^2 (iPS-NKT)<br>1×10^9 cells/m^2 (DC/Gal)<br>Intravenous administration on day 0, 14, 28	No death, abnormal weight gain, physical abnormality, inflammatory cytokine until day 42	end	Non-GLP
	Human iPS-NKT<br>Mouse DC/Gal	Human IL7/15 expressing NSG mice	2×10^9 cells/m^2 (iPS-NKT)<br>1×10^9 cells/m^2 (DC/Gal)<br>Intravenous administration on 0, 14, 28	No weight loss and GVHD indication until day 70	end	Non-GLP
Soft Agar Colony Test	Human iPS-NKT	soft agar-agar medium	1×10^6 cells/well<br>Sowing in	Scaffold-independent growth potential no	end	Regenerative Medicine GLP [Ref. vii.].
Genome test	Human NKT-iPS and iPS-NKT	Karyotype analysis	50 cells	No karyotype abnormality	End	Non-GLP
		G-Band Method	1×10^6 cells	No pathogenic mutation	End	Non-GLP
		Exosome analysis for genetic mutations based on COSMIC CGC Tier1 and Shibata list	1×10^6 cells	No copy number abnormality	end	Non-GLP
Tumorigenic test	Human NKT-iPS	Mouse (NOG)<br>6 weeks old<br>Group composition<br>10 animals/group x 2 groups of 10 females/group x 2 groups of 10 males/group<br>(media control group, one dose of test substance group)<br>Observation period: 12 weeks	single round<br>3.3×10^8 cells/kg intravenous administration	No tumorigenicity<br>No human cells remain	end	Regenerative Medicine Products GLP [Ref. viii.].
	Human NKT-iPS<br>(Newly acquired shares HV #2)	Mice (NOG)<br>6 weeks old<br>Group composition<br>10 animals/group x 2 groups of 10 females/group x 2 groups of 10 males/group<br>(Media control group, 1 dose of test substance group)<br>Observation period: 12 weeks	single round<br>3.3×10^8 cells/kg intravenous administration	No tumorigenicity<br>No human cells remain	end	Regenerative Medicine Products GLP [Ref. ix.].
	Human iPS-NKT preparation	Mice (NOG)<br>6 weeks old<br>Group composition<br>14 animals/group of each sex x 2 groups<br>(media control group, one dose of test substance group)<br>Observation period: 1 year	single round<br>3.3×10^8 cells/kg intravenous administration	No tumorigenicity<br>1 case positive for anti-human nuclear antibody, negative for anti-Ki-67 antibody	end	Regenerative Medicine Products GLP [Ref. x.].

1.2.5. Pharmacological and kinetic studies

1.2.5.1. Productivity of IFN-γ and IL-4 of iPS-NKT cell

To compare the production patterns and levels of cytokines, which are considered important indicators of adjuvant action of iPS-NKT cells, with those of normal NKT cells (NKT line cells), iPS-NKT cells and NKT line cells were co-cultured with DC or α-GalCer-pulsed dendritic cells (DC/Gal), a known ligand for NKT cells, and the amount of cytokines (IFN-γ and IL-4) in the supernatant was measured 24 hours later. The results showed that iPS-NKT cells produced more IFN-γ, which activates immunity, and less IL-4, which also suppresses immunity, than normal NKT line cells (Fig. 9 IFN-γand IL-4 production capacity of iPS-NKT cells). Therefore, iPS-NKT cells are expected to be more effective in activating immunity than NKT line cells.

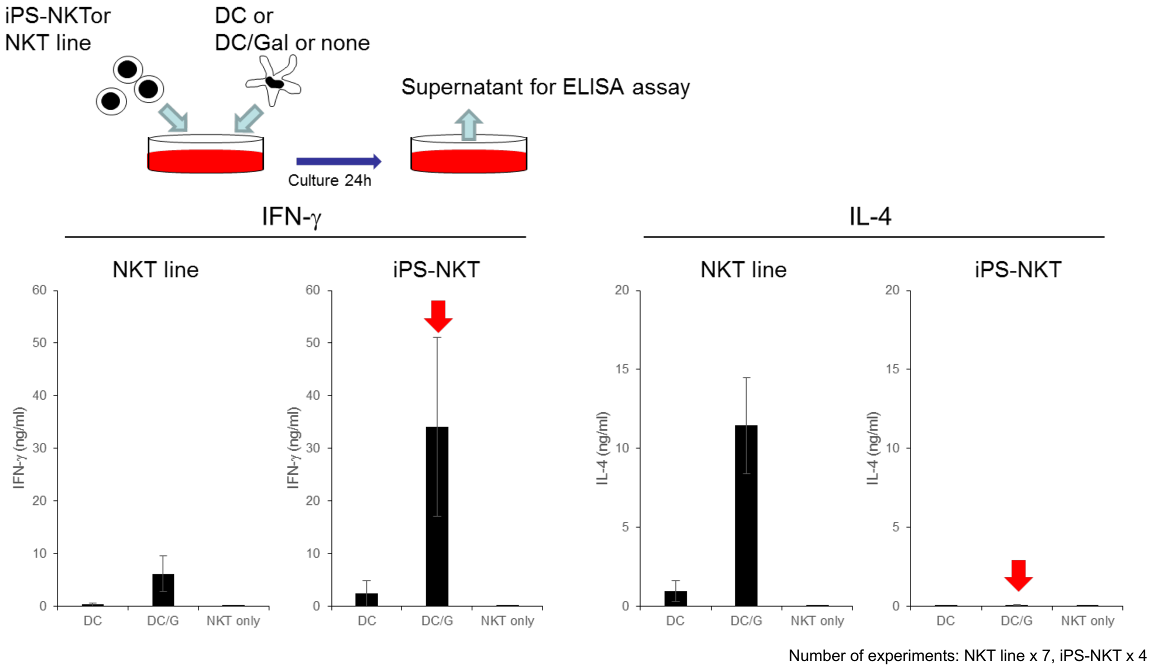

Figure 9 Productivity of IFN-γ and IL-4 of iPS-NKT cell

1.2.5.2. Anti-tumor effect of iPS-NKT cell (*in vitro*)

To confirm whether iPS-NKT cells have direct antitumor effects in vitro, iPS-NKT cells were co-cultured with tumor cell K562 and non-tumor control cell Auto-PHA blast, and tumor cell killing activity was assessed. As a result, iPS-NKT cells showed a very strong cell-killing effect on K562, but a weak cell-killing effect on Auto-PHA blast; no cell-killing effect was observed on NKT line cells. Therefore, we evaluated the antitumor effects of NKT cells and iPS-NKT cells using six tumor cell lines (K562; human leukemia, NCI-H460; human large cell carcinoma, A549; human lung cell basal epidermoid adenocarcinoma, HT-29; human colon cancer, COLO205; human colon cancer, Detroit562; human pharyngeal cancer). As a result, iPS-NKT cells showed strong antitumor activity against all six tumor cell lines (Fig. 10 Antitumor activity of iPS-NKT cells).

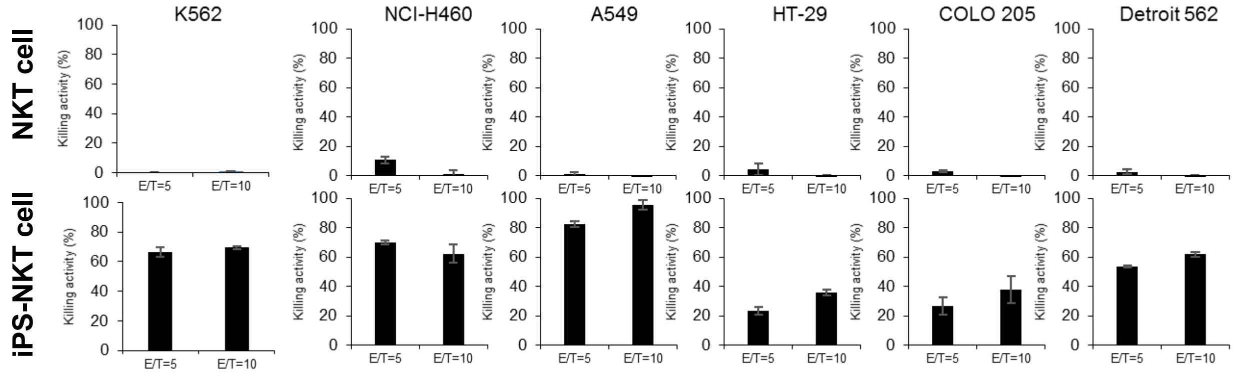

Figure 8 Anti-tumor effect of iPS-NKT cell

1.2.5.3. Pharmacodynamic test of iPS-NKT cell

Since the test for detecting human-derived NKT cells in mice has not yet been standardized, we first conducted a preliminary study to confirm the suitability of the assay method and the outline of the cell concentration transition, and selected the ^{51}Cr-labeled kinetic study. Since accumulation in the liver was observed in the preliminary study, the observation period was extended to 2 weeks, which is assumed to be the period of repeated administration in humans, in this study. As shown in Table 15, when iPS-NKT cells were intravenously administered to immunocompetent mice, they first accumulated temporarily in the lungs and then distributed throughout the body. The radioactivity in the blood decreased to less than 1% (0.33%) 48 hours after administration and was not observed thereafter, suggesting that the radioactivity that migrated to the organs does not return to the blood. The spleen, liver, femur, kidney, and bone marrow were the most prominent organs of distribution. 27% of the administered radioactivity remained in the liver at 48 hours post-dose, and 21% remained at 2 weeks (336 hours post-dose). While considering that iPS-NKT cells were detected in the lungs and spleen on day 3 post-dose in the kinetic study conducted in RIKEN using fluorescent-labeled iPS-NKT cells but hardly detected in the liver, the radioactivity observed in the liver was thought to be derived from host immune cells that phagocytosed the iPS-NKT cells and not from the iPS-NKT cells themselves.

To prove this, mouse organs were harvested after iPS-NKT cell administration, cells were isolated, and a cell sorter was used to detect human NKT cells./g), but not in the liver, spleen, or kidney (Table 16), nor at any later time point.

Next, a kinetic study using the cell sorter was conducted in the same manner as above using the NOG mice used in the nonclinical safety study. The results showed that per organ weight (g), iPS-NKT cells administered were distributed in the blood up to 3 cells at 24 hours after administration, in the liver up to 1 cell at 4 hours after administration, 1 cell at 24 hours after administration, 1 cell at 48 hours after administration, and 29 cells at 48 hours after administration. In the kidney, up to 3 cells were distributed at 48 hours post-dose, and in the lung, up to 29 cells were distributed at 4 hours post-dose, but they disappeared from the blood and each organ after 96 hours post-dose (Table 17).

Therefore, we concluded that the distribution in the liver observed in the kinetic study using the labeled body did not reflect the distribution of iPS-NKT cells due to radioactivity alone, and that the iPS-NKT cells had in

fact almost disappeared.

Table 12 Summary of non-clinical pharmaco-dynamics study

Category	Test substance	Subject etc.	Dose and method	Results	Status	Miscellaneous
Dynamics study	Human iPS-NKT (Fluorescent label)	NOG mice	intravenous administration	iPS-NKT cells detected in lung and spleen on day 3 after treatment	end	Non-GLP
	Human iPS-NKT (Fluorescent label)	Human IL7/15 knock-in NOG mice	3.3×10^8 cells/kg intravenous administration	Below the limit of quantitation at all measurement points	being carried out	Non-GLP
	Human iPS-NKT (^{111} IN,^{51} Cr)	Mice (BALB/c) 6 weeks old 3 females/arm x 4 arms of 3 males each (control arm, 3 doses of test substance arm)	single round 3.3 x 10^6 , 3.3 x 10^7 , 3.3×10^8 cells/kg intravenous administration (60-70 seconds/animal)	After accumulating in the lungs, it is prominently distributed in the spleen, liver, kidney, and bone marrow	end	Non-GLP
	Human iPS-NKT (^{51} Cr)	Mice (BALB/c) 7 weeks old 3 males/arm x 6 arms	single round 1.7×10^7 cells/kg intravenous administration	Distributed in spleen, liver, femur, kidney and bone marrow Approximately 21% of the administered radioactivity remained in the liver at 336 hours post-dose (Table 15)	end	Reliability Criteria
	Human iPS-NKT (Detected by cell sorter)	Mice (BALB/c) 7 weeks old 3 males/arm x 3 arms	single round 1.7×10^7 cells/kg intravenous administration	31 cells equivalent/g detected in lungs 4 hours after administration	end	Reliability Criteria
	Human iPS-NKT (detected by cell sorter)	NOG mice 7 weeks old 3 males/arm x 6 arms	single round 3.3×10^8 cells/kg intravenous administration	Detected in lungs, blood, kidneys, and liver 4 hours after administration; resolved by 96 hours	end	Reliability Criteria

Table 13 Percentage distribution of radioactivity in tissues （% of dose） after a single intravenous administration of [^{51}Cr]labeled iPS-NKT cell to mice

Tissue	Percentage distribution（% of dose）					
	4 h	24 h	48 h	96 h	168 h	336 h
Blood	1.89	0.69	0.33	0.00	0.00	0.00
Brain	0.01	0.00	0.00	0.00	0.00	0.00
Mandibular gland	0.03	0.02	0.03	0.03	0.01	0.02
Thymus	0.00	0.00	0.00	0.00	0.00	0.00
Heart	0.06	0.03	0.03	0.02	0.02	0.02
Lung	3.11	0.26	0.27	0.16	0.34	0.07
Liver	30.08	29.52	26.99	26.01	24.44	20.58
Kidney	2.30	2.04	1.62	1.42	0.92	0.48
Spleen	2.62	3.78	3.20	2.37	2.68	2.42
Pancreas	0.07	0.03	0.04	0.03	0.01	0.02
Skin（abdominal）	3.24	2.01	1.93	1.58	1.33	1.08
Skeletal muscle	2.32	1.00	0.99	0.72	0.60	0.52
Bone marrow	3.03	2.92	3.41	2.89	2.89	3.29

Testis	0.04	0.03	0.04	0.04	0.04	0.03
Prostate gland	0.00	0.00	0.00	0.00	0.00	0.00
Stomach	0.03	0.02	0.03	0.02	0.02	0.02
Small intestine	0.27	0.14	0.12	0.08	0.07	0.05
Large intestine	0.12	0.06	0.07	0.04	0.04	0.03

Data are expressed as the mean values of three animals.

Table 14 iPS-NKT cell counts calculated by cell sorter in various organs detected 4 hours after administration of iPS-NKT cells to Balb/c mouse

Organ	ID of animal	Cell count		
		Number of events	Cells	Cells/g or mL
Blood	YH2M01	1	1	3
	YH2M02	1	1	4
	YH2M03	1	1	4
Liver	YH2M01	0	0	0
	YH2M02	0	0	0
	YH2M03	0	0	0
Kidney	YH2M01	0	0	0
	YH2M02	0	0	0
	YH2M03	0	0	0
Lung	YH2M01	3	7	51
	YH2M02	1	3	21
	YH2M03	1	3	21
Spleen	YH2M01	0	0	0
	YH2M02	0	0	0
	YH2M03	0	0	0

Table 15 Measurement of the number of iPS-NKT cells in organs after administration of iPS-NKT cells by cell sorter

Time after administration	The number of iPS-NKT cell (cells/mL or g)			
(Hour)	Blood	Liver	Kidney	Lung
4	0	1	0	7
	0	0	0	29
	0	1	0	0
24	0	0	0	0
	0	0	0	0
	3	1	0	0
48	0	0	0	0
	0	0	3	0
	0	1	0	0
96	0	0	0	0
	0	0	0	0
	0	0	0	0
336	0	0	0	0
	0	0	0	0
	0	0	0	0

1.2.6. Clinical study related to NKT and DC/Gal (Efficacy and Safety)

Clinical studies using DC/Gal alone and DC/Gal and NKT cells have been conducted for non-small cell

lung cancer and HNSCC at Chiba University Hospital, and safety and efficacy have been confirmed [Ref.x.~xvi.]. A list of the studies conducted is shown in Table 18 and an overview of NKT cell therapy for HNSCC is shown in Figures 11 through 12.

Table 18 List of NKT cell-targeted therapies implemented to date

Title	Test design/ Dosage and Administration	Number of subjects	Main Purpose	Main Results
Phase I Study of Alpha GalCer DC Therapy in Unresectable Advanced or Recurrent Non-Small Cell Lung Cancer after Completion of Standard Therapy	Open-label, dose escalation αGalCer DC $5x10^7$, $2.5x10^8$ or $1x10^9$ /m^2 /week twice intravenously, followed by a 4-week rest period and again twice weekly for a total of 4 doses	9 cases (3 cases for each dose)	safety immunoreactivity antitumor effect	No serious adverse events were observed. After 1 or 2 cell doses, enhanced NKT cell counts and IFN- production in the peripheral blood were observed in the high-dose group. No tumor progression was observed in all 3 patients in the high-dose group for 23~26 weeks. [Ref. x.].
Phase I/II Study of Alpha GalCer DC Therapy in Unresectable Advanced or Recurrent Non-Small Cell Lung Cancer after Completion of Standard Therapy	Open-label, single dose αGalCer DC $1x10^9$ /m^2 / intravenously twice a week, followed by a 4-week rest period, then again twice a week for a total of 4 doses	17 cases	safety immunoreactivity OS	Serious adverse events included one case of deep vein thrombosis unrelated to study treatment. Significant OS prolongation was observed in 10 patients with increased α GalCer-reactive IFN- producing cell counts (NKT cell activation) compared to 7 non-increased cases. [Ref. xi.].
Phase I study of NKT cell therapy in unresectable advanced or recurrent non-small cell lung cancer after completion of standard therapy	Open-label, dose escalation NKT cells 1 x 10^7 or 5 x 10^7 /body/week twice intravenously	6 cases (3 cases for each dose)	safety immunoreactivity antitumor effect	No serious adverse events were observed. After 1 or 2 cell doses, an increase in the number of α GalCer-reactive IFN-γ-producing cells was observed in 2 patients in the high-dose group, and no tumor progression was observed over a 9~12 month period. [Ref. xii.].
Phase I study of αGalCer DC therapy in unresectable advanced or recurrent squamous cell carcinoma of the head and neck after completion of standard therapy	Open-label, single dose αGalCer DC $1x10^8$ /body/week, total of 2 times nasal submucosal administration	9 cases	safety immunoreactivity antitumor effect	No serious adverse events were observed. Increased number of αGalCer-reactive IFN-γ-producing cells was observed in 8/9 patients; partial response was observed in 1/9 patients. [Ref. xiii].
Phase I/II study of combined immunotherapy using αGalCer DC and NKT cells in patients with unresectable advanced or recurrent squamous cell carcinoma of the head and neck after completion of standard therapy	Open-label, single dose αGalCer DC $1x10^8$ /body/week, total of 2 subnasal submucosal doses, followed by a single dose of NKT cells $5x10^7$ /body in the tumor-feeding artery	8 cases	safety immunoreactivity antitumor effect	One Grade 3 case of pharyngeal skin thinning (associated with rapid tumor shrinkage) was observed as a serious adverse event. Increased number of αGalCer-reactive IFN-γ-producing cells was observed in 5/8 patients; partial response was observed in 3/8 patients. [Ref. xiv, xv.].
Phase II study of combined immunotherapy using αGalCer DC and NKT cells in operable locally recurrent squamous cell carcinoma of the head and neck	Phase II, open-label, single dose One week after a single dose of αGalCer DC $1x10^8$ /body in the nasal submucosa before salvage surgery, a single dose of NKT cells $5x10^7$ /body was administered in the tumor-feeding artery	10 cases	safety exemption response antitumor effect	No serious adverse events were observed. In CT evaluation after 3 weeks of αGalCer DC treatment, partial response was observed in 5/10 patients. 7/10 patients showed an increase in the number of tumor-infiltrating NKT cells. [Ref. xvi.].
DC/Gal-based immuno-cell therapy	Unblinded, single dose DC/Gal $1x10^9$ /m^2/week	35 cases	OS Safety	The median survival (MST) in the Full analysis set was 667 days (95%

for unresectable advanced or recurrent non-small cell lung cancer<br>-A Phase II Trial	After 2 intravenous administrations, the drug is withdrawn for 4 weeks, and then administered again twice a week for a total of 4 doses.		Immuno-response	confidence interval (CI) 451~790 days). The study was conducted with an expected MST of 17 months and a threshold MST of 8 months, and the lower 95% CI did not include the threshold MST, which was considered to be on target. Recurrence-free survival was 85 days (95% CI 80-113 days), and response and disease control rates were 2.9% (95% CI 0.1 ~14.9%) and 42.9% (95% CI 26.3~60.7%).No serious adverse events related to cell administration were observed.[Ref. xvii.]
A two-arm randomized phase II clinical trial on the efficacy of adjuvant therapy with DC/Gal in patients with advanced squamous cell carcinoma of the head and neck after standard therapy	Double blinded,<br>DC/Gal or control (non-treated DC) $1x10^8$ / body/week, administered twice totally in submucosa of nasal concha	23 cases (sample size was 66 cases)	Recurrence-free survival<br>Safety<br>2-year recurrence-free survival<br>2-year OS<br>Immune response	Median recurrence-free survival was not reached (95%CI: 0.85-NE) vs 2.60 years (95%CI: 0.35-NE) in the control group (n=9), p=0.3613 (Log-rank test) in treatment arm (N=13) and control arm (N=9) (p=0.3613, Log-rank test), respectively. The 2-year recurrence-free survival was 69% (95%CI: 48-99) vs 56% (95%CI: 31-100) in the treatment arm and control arm, respectively. The 2-year overall survival rate was 82% (95%CI: 62-100) vs 71% (95% CI 45-100%) in the treatment arm and control arm (p=0.443, Log-rank test), respectively, showing no statistically significant benefit between the two arms.<br>There were no serious adverse events judged to be related to this cell therapy, and one serious adverse event (Grade 5) was observed that was not ruled out to be related. The efficacy and safety evaluation committee reviewed the case, including clinical, autopsy, and immunological findings, and concluded that although they did not support a causal relationship with the submucosal administration of DC/Gal, a causal relationship with the study cannot be ruled out because of the presence of a reasonable amount of invasiveness in the study itself.

DC/Gal treatment for non-small cell lung cancer resulted in a significant survival benefit compared to standard of therapy. Furthermore, when the 17 patients treated were divided into two groups: the immunotherapy response group (n = 10, red line) and the non-response group (n = 7, black line), which showed enhanced IFN-γ productivity after cell administration, the survival curves of each group after cell therapy were compared. IFN-γ productivity was enhanced in the immunotherapy response group, which showed a remarkable survival benefit [Ref. xv].

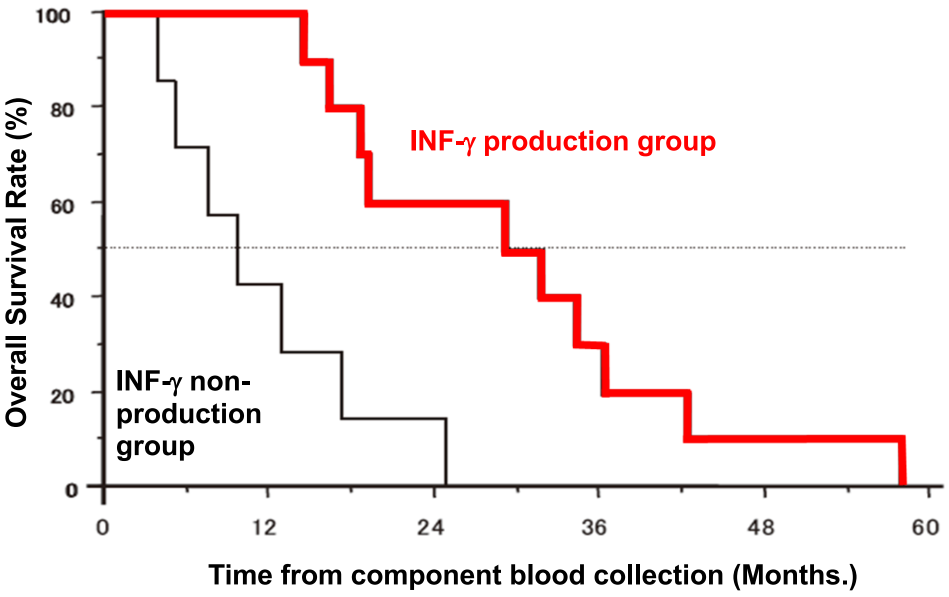

Figure 9 Overall survival rate and INF-γ productivity

Results of a phase II clinical trial using DC/Gal and activated NKT cells (5×10^7 cells/body of cells derived from patients' peripheral blood) for head and neck cancer showed responses in 5 out of 10 patients. Although there was no relationship between induction of antitumor activity in the peripheral blood and antitumor efficacy, there was a trend toward stronger antitumor efficacy in patients with more NKT cells in the tumor tissue [Ref. xvi.].

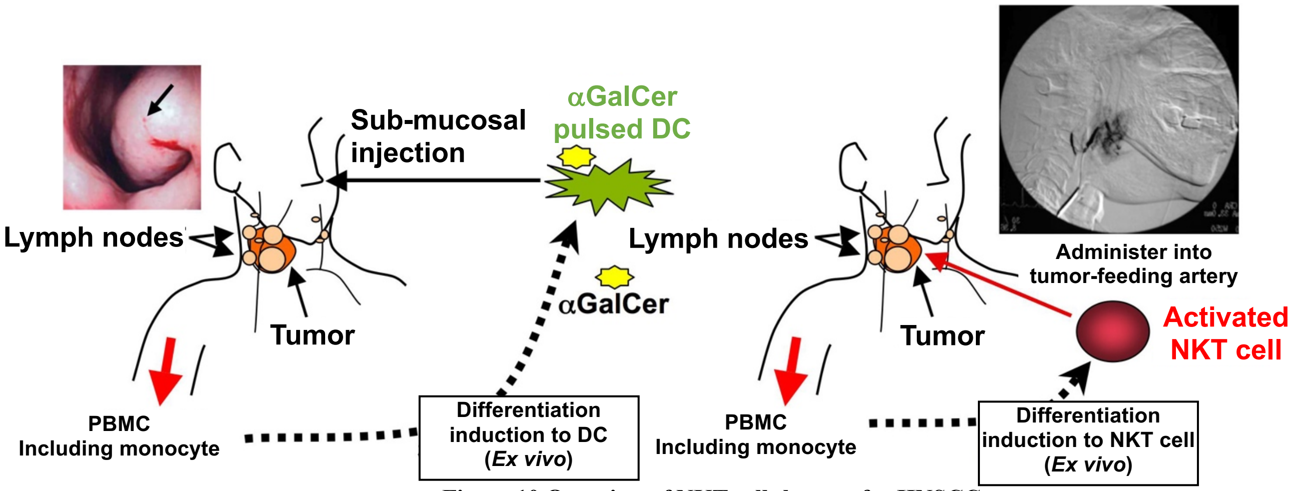

Figure 10 Overview of NKT cell therapy for HNSCC

1.2.7. Expected Side Effects

The main "side effects" expected in relation to DC/Gal or iPS-NKT cells and examples of treatments are listed below.

① Elevated blood pressure: Antihypertensive agents should be administered according to symptoms and severity.

② Fever: Blood test, chest X-ray, blood culture should be performed. Regarding the possibility of infectious disease, antibiotic agents should immediately be administered. Antipyretics should not be administered if there is no febrile distress but should be given as needed if febrile distress is marked.

③ Respiratory distress: Administer oxygen promptly to stabilize blood oxygenation. Blood tests, X-rays and CT scan (contrast if possible) should be performed as part of the screening for the diagnosis. Possible causes of severe respiratory failure during infusion include allergic and embolic events (especially when iPS-NKT is administered). Steroids and epinephrine should be administered according to the severity of the disease, as well as antithrombotic therapy for embolism.

④ Shock: The physician in charge and the attending physician will respond promptly. Check vital signs, perform blood tests, and administer steroids or epinephrine according to the severity of the illness. Administer oxygen and pressor agents for respiratory failure and hypotension, depending on the severity of the illness.

⑤ Pharyngeal fistula: Since pharyngeal fistula associated with tumor shrinkage was observed in a previous study of combination therapy with autologous NKT cells and DC/Gal, this adverse event should be kept in mind in this study as well. If this adverse event is observed, infection prophylaxis and, if necessary, pharyngeal fistula closure should be performed.

⑥ Cerebral infarction: Because the cells are administered into the arteries of the neck, there is a possibility of blood clots flowing into the brain during cannulation. The patient should be evaluated after consulting a neurosurgeon for functional impairment, checking vital signs, and administering cerebro-protective drugs.

⑦ Bleeding and hematoma at the puncture site: This may occur with arterial puncture of the cannula. If severe hematochezia develops, consult a plastic surgeon and consider removal of hematochezia. Mild to moderate hematomas can be absorbed with observation.

1.3. Rationale

1.3.1. Dosing

The results of intravenous administration of 1×10^6 and 2×10^6 cells/body (20 g) of human iPS-NKT cells to NKT-deficient B6 mice three times on Days 0, 14, and 28, and intravenous administration of mouse DC/Gal 1×10^9 cells/m^2 on the same days, showed no death or other safety concerns in either group. The No Observable Adverse Effect Level (NOAEL) of human iPS-NKT cells in combination with mouse DC/Gal was 2×10^6 cells/body = 1×10^8 cells/kg. This dose is equivalent to 1×10^8 cells/kg $\times 3 \fallingdotseq 3 \times 10^8$ cells/m^2 according to FDA “Guidance for Industry Estimating the Maximum Safe Starting Dose in Initial Clinical Trials.” Then assuming a safety margin as 1/10, we set 3×10^7 cells/m^2 as the starting dose in this study. In addition, a Phase I study aimed at confirming the

tolerability of iPS-NKT cells alone is ongoing at the time of planning this study (April 2022), and the tolerability of 3×10^7 cells/m^2 of iPS-NKT cells has been confirmed. Regarding this result from the Phase I study, 3×10^7 cells/m^2 of iPS-NKT cells is appropriate as the starting dose of iPS-NKT cells in this study. The dose of auto-DC/Gal was set at 1×10^8 cells, a dose that has been confirmed to be tolerable and safe in combination with autologous NKT cells in previous studies.

Regarding the dosage, the number of administrations of iPS-NKT cell in this study was set to be at once, since the protocol that showed efficacy in previous combination studies of auto-DC/Gal and autologous NKT cells involved one or two doses of auto-DC/Gal followed by one dose of autologous NKT cells. In addition, compared to the intravenous administration in the non-clinical study, the tumor-feeding artery administration is expected to be more effective because of its higher local tumor concentration.

1.3.2. Target population

Since this study is an FIH study, patients with recurrent or advanced head and neck cancer who were refractory or intolerant to standard treatment are included in the study based on the "Guidelines for Clinical Evaluation Methods of Antineoplastic Agents" (Pharmaceutical and Food Safety Bureau of Japan, March 31, 2021, #033101). In addition, since the route of iPS-NKT cell administration is defined as tumor-feeding arterial administration, patients with lesions amenable to intravenous infusion and evaluable are set as the selection criteria.

1.3.3. Endpoint

Since this is an FIH study, we decided to evaluate the tolerability of the combination of iPS-NKT cell and auto-DC/Gal using a 3+3 design with single dose cohort. If the number of DLT cases was 1, the same dose was administered to 3 additional cases, and if the number of DLT cases was 2 or more out of 6, the study was terminated. Finally, the MTD will be defined as the maximum dose at which one or fewer DLTs occur in a total of six subjects. In addition, the auto-DC/Gal and iPS-NKT should not be administered to more than 2 subjects on the same day, and auto-DC/Gal and iPS-NKT cell administration in the next subject should be started after an interval of at least 7 days. The above procedure will be used to evaluate DLT, and the Independent Data Monitoring Committee will decide whether to continue the study and the MTD after evaluating all adverse events and other safety information including DLT.

In a preclinical pharmacokinetic study using iPS-NKT cell, the concentration of iPS-NKT cell in mouse blood decreased to 0.33% of the dose at 48 hours after a single intravenous administration and was not detected thereafter. Therefore, two weeks after administration of iPS-NKT cell is reasonable to assess the Dose Limiting Toxicity.

1.3.4. Safety management of subjects

Weight, vital signs, ECOG PS, tumor evaluation, hematology, blood biochemistry, coagulation, urinalysis,

and radiological imaging will be performed throughout the study period. In addition, the DLT assessment period (from auto-DC/Gal administration to day 14 after iPS-NKT cell administration) and day 1 of the observation period will, in principle, require hospitalization to carefully monitor subject safety. The principal investigator (PI) or sub-investigator (SI) may leave the hospital at his/her own discretion only if the patient does not develop non-hematologic toxicity or hematologic toxicity of Grade 2 or higher and Grade 3 or higher on or after Day 7 of iPS-NKT cell administration, and be able to contact the study site and home doctor of the subjects, if any, in the event of an adverse event.

Based on the above, we have determined that it is ethically and scientifically appropriate to conduct this study.

2. Objective and necessity of Study

Although the combination therapy of iPS-NKT cell and auto-DC/Gal has been confirmed to inhibit tumor growth in preclinical studies and to be safe to a certain degree in mice, there has been no experience with human administration to date. Therefore, it is necessary to explore the tolerability, safety, and efficacy of the combination therapy of iPS-NKT cell and auto-DC/Gal in human for future development, as well as the immunological kinetics induced by experimental therapy. This study will be conducted primarily to confirm the tolerability of the combination therapy by assessing the DLT at the administered dose, and secondarily to confirm the safety profile and efficacy evaluation, as well as to explore the immunokinetic of the combination therapy.

3. Study Subjects

Patients who meet all of the following selection criteria and none of the exclusion criteria will be considered eligible subjects.

3.1. Inclusion Criteria

Patients who meet all the following criteria will be eligible.

1) Patients with recurrent or advanced head and neck cancer, refractory or intolerant to standard of care and who have evaluable lesions that can be treated with intra-arterial infusion to tumor.
2) Patients who have not been on previous therapy within 1 month. Any type of previous treatment is acceptable.
3) Patients must be at least 20 years old but less than 80 years
4) Patients must have an ECOG Performance Status of 2 or less.
5) Patients who meet the following laboratory data. Hemoglobin ≥ 10 g/dL, White blood cell count ≥ 3000/μL and platelet count ≥ 75,000/μL, Serum creatine ≤ 1.5 mg/dL, Total bilirubin ≤ 1.5 mg/dL, AST (GOT), ALT (GPT) < 2.5 times the upper limit of facility reference values, SpO2 (under room air) ≥ 93%.

6) Patients who are expected to have a prognosis of 3 months or more.
7) Patients for whom written consent has been obtained from the individual.

【Rationale】
1) Patients with recurrent or advanced head and neck cancer who were refractory or intolerant to standard treatment are included in the study based on the "Guidelines for Clinical Evaluation Methods of Antineoplastic Agents" (Pharmaceutical and Food Safety Bureau of Japan, March 31, 2021, #033101). In addition, since the route of iPS-NKT cell administration is defined as tumor-feeding arterial administration, patients with lesions amenable to intravenous infusion and evaluable are set as the selection criteria.
2-5) Considering the safety and ethics of the subject, and data reliability
6): Considering the duration of the study, it was set as the minimum period considered necessary to evaluate the tolerability and safety of the study treatment.

3.2. Exclusion Criteria

Patients who meet any of the following conditions are not eligible.

1) Patients who are HBs, HCV[1)], HIV or HTLV-1 antibody-positive or HBs antibody-negative but have HBV-DNA detected by HBV-DNA quantitative testing.
2) Patients who have been taking or injecting corticosteroids (methylprednisolone 10 mg/day or higher or equivalent) or immunosuppressive drugs within at least 2 weeks prior to the start of the study product.
3) Women who are pregnant[2)], lactating, or planning to become pregnant during the study and men who do not agree with using any of effective contraceptive methods under the guidance of a physician during the study period or up to 14 days after the last dose of the study product.
4) Patients with active autoimmune disease requiring systemic or immunosuppressive therapy with corticosteroids or biologic agents.
5) Patients who have experienced immune-related adverse events with immune checkpoint inhibitors.
6) Patients with poorly controlled diabetes mellitus.
7) Patients with severe lung disease (mMRC Breathlessness Scale Grade 2 or higher) or with a history of non-infectious interstitial lung disease requiring steroid treatment.
8) Patients with significant cardiac disease (NYHA class III or greater).
9) Patients with concurrent multiple cancers.
10) Patients who are unable to use contrast agents in radiograph (e.g., allergy, kidney disfunction).
11) Patients with a history of hypersensitivity to human serum albumin products or proteins of foreign origin.
12) Patients who, at the time of informed consent, are participating in other clinical trials or clinical studies and are receiving other investigational products or are judged by the PI or SI to have residual effects of adverse events

1) If HCV antibody is positive, HCV-RNA quantification test is performed and negative result is acceptable for inclusion.
2) Women of childbearing potential include all women who have experienced menarche, have not undergone sterilization procedures (hysterectomy, bilateral tubal ligation or bilateral oophorectomy, etc.), and have not reached menopause. Menopause is defined as no menstruation for at least 12 consecutive months without any other medical reason.

caused by such products.

13) Patients with completely identical genotypes[3)] of HLA-A, B and C to the investigational product.

14) Patients who are prohibited with blood apheresis because of comorbidities such as unstable angina, A-V block class 2 or greater, WPW syndrome, complete left bundle branch block, systolic blood pressure of 90 mmHg or less, or 170 mmHg or more.

15) Patients who are judged to be unsuitable to participate in the study.

【Rationale】

1) When iPS-NKT cells are administered intravenously, they have been found to accumulate in the liver. In this study, iPS-NKT cells were administered intravenously into tumor-feeding arteries, and although it is expected that there will be little systemic circulation, the eventual effect on the liver cannot be ruled out, and therefore, this setting was determined based on consideration of the safety of the subjects.

2, 4-5) The iPS-NKT cells are intended for direct and indirect immune activation in the target patients and were set up to exclude the influence of diseases and drugs that affect the immune status of the target patients.

3) Since the effects of iPS-NKT cells on the fetus and fertility have not been confirmed to date, the dose was set to avoid such effects in the target population. Since iPS-NKT cells administered into the body are considered to be almost completely eliminated from the body on the seventh day after administration, a contraceptive period was established.

6-8) Considering the safety of the subjects.

9) This was established to exclude the impact of adverse events due to cancers other than head and neck. The time period was set based on the recurrence rate after curative treatment of gastrointestinal cancer, which is a particularly frequent overlapping cancer of the head and neck, and because the risk of recurrence is considered to be high within 2 years.

10) Since the use of contrast enhancements is mandatory in this study to confirm tumor-feeding arteries in the subject

11) Considering the subjects' safety

12) To exclude the effects by other experimental agent on other clinical trials.

13) Considering the PK/PD analysis, we need to detect the iPS-NKT cells with different genotype from subjects one.

14) Because it is impossible to conduct this study in patients who are unable to have component blood samples taken for DC/Gal preparation.

15) Other settings were made to allow for exclusions when the investigators judged that the study could not be safely conducted.

[3)] The genotype of iPS-NKT cell used in this study is as follows:
A*26:01-B*35:01-C*03:03
A*26:03-B*35:01-C*03:03

4. Informed Consent

4.1. Preparation and Revision of Consent and Other Explanatory Documents

The PI shall prepare the consent document and other explanatory documents used to obtain consent for participation in the clinical trial from the subject, using plain language as much as possible. If it is deemed necessary to revise the consent document and other explanatory documents, these documents shall be revised.

The PI shall submit the prepared or revised consent document and other explanatory documents to the Clinical Trial Review Committee for its approval.

4.2. Timing and Method on Informed Consent

1) Obtaining Consent

PI or SI should hand the informed consent form and related documents approved by IRB etc., and should carefully explain the contents described below in “4.3. Contents of Explanation for subjects” to the candidate patients. And CRCs can supplementally explain for them if needed. PI or SI should perform the Screening tests after confirming the subjects’ comprehension and obtaining the written, free and voluntary consent.

2) Explanation

The PI or SI will provide the subject with an opportunity to ask questions and sufficient time to decide whether or not to participate in the study before obtaining consent, and will answer the subject's questions to the subject's satisfaction.

3) Filling out a Consent Form and Delivery of the Explanation Document

When consenting, the PI or SI who provided the explanation should affix his/her name and seal or sign the consent form and fill out the date of the explanation. The subject shall affix his/her name and seal or sign the consent form and fill out the date of consent. If the study coordinator provided supplementary explanations, he/she should also affix his/her name and seal or sign the consent form and fill out the date of the explanation. After obtaining consent, a copy of the explanation document and the informed consent form should be given to the subject.

4) Amendment of Informed Consent Form

Whenever the PI or SI revises the informed consent form or other explanatory documents due to the acquisition of new information that may be relevant to the subject's consent, the PI or SI will explain the revised informed consent form or other explanatory documents to the subject and obtain the subject's written, free and voluntary consent to continue participating in the study. The subject's consent for continued participation in the study shall be obtained in writing. If new important information is obtained that could affect the subject's consent, this information will be immediately provided to the subject, recorded in writing, and the subject's continued participation in the study will be confirmed.

4.3. Contents of Explanation for subjects

The following items should be included in the explanatory document prepared by the PI:

1) The name of the regenerative medicine to be provided and the fact that a regenerative medicine provision plan has been submitted to the Minister of Health, Labour and Welfare.
2) The name of the medical institution providing the regenerative medicine, etc., and the names of the administrator, PI, and physician or dentist providing the regenerative medicine, etc. at the medical institution (In the case where regenerative medicine, etc. is conducted as a multi-center collaborative study, the name of the PI and the names of other medical institutions where the regenerative medicine, etc. is conducted and the name of the administrator of the medical institutions.)
3) Purpose and details of the regenerative medicine, etc. to be provided
4) Information on the cells to be used for regenerative medicine, etc.
5) Reason for selection as a recipient of regenerative medicine, etc. (limited to cases where regenerative medicine, etc. is to be provided as part of research)
6) Anticipated benefits and risks of the regenerative medical care, etc.
7) The refusal to receive regenerative medicine, etc. is voluntary.
8) Withdrawal of consent.
9) The patient shall not be treated disadvantageously by refusing to receive regenerative medicine, etc. or by withdrawing consent.
10) The method of disclosing information on the research (limited to cases where regenerative medicine, etc. is conducted as research).
11) The fact that research protocols and other materials related to the implementation of the research may be obtained or inspected upon request by the person receiving regenerative medicine, etc. or a surrogate, and the method of obtaining or inspecting such materials (limited to cases where regenerative medicine, etc. is performed as part of the research).
12) The protection of personal information of persons receiving regenerative medicine, etc.
13) Methods of storage and disposal of samples, etc.
14) Status of involvement in research as prescribed in each item of Article 8-8(1) (limited to cases where regenerative medicine, etc. is performed as research)
15) System for responding to complaints and inquiries
16) The costs involved in the regenerative medicine, etc.
17) Existence and details of other treatment methods, and comparison with the expected benefits and risks of other treatments.
18) Compensation for damage to health resulting from the provision of the regenerative medicine, etc. (limited to cases where regenerative medicine, etc. is provided as part of research).
19) Handling of the important findings (including incidental findings) that may be obtained concerning the health of a subject who receives regenerative medicine, etc., or genetic characteristics that can be passed on to offspring,

etc., if there is a possibility.

20) If there is a possibility that a sample, etc. obtained from a subject who receives regenerative medicine, etc., will be used for future research that is not specified at the time consent is obtained from the subject or will be provided to other medical institutions, a statement to that effect and the details of such research that are assumed at the time consent is obtained.
21) Matters to be examined by the Specified Authorized Regenerative Medicine Committee, that conducts the examination of the regenerative medicine etc., and other matters related to the Specified Authorized Regenerative Medicine Committee, related to the regenerative medicine, etc.
22) In the case where research is conducted with the provision of research funds, etc. by a manufacturer or seller of pharmaceuticals, etc. who manufactures and sells or intends to manufacture and sell pharmaceuticals, etc. to be used in the research or their specifically related entities, the details of the contract prescribed in Article 32 of the Act on the Safety of Regenerative Medicine (limited to the case where regenerative medicine, etc. is conducted as research).
23) Other matters necessary for the provision of the regenerative medicine, etc.

5. Study Method

5.1. Design

This is an open-label, single arm, Phase I study to evaluate the tolerability, safety, pharmacokinetics, pharmacodynamics, and antitumor effects of iPS-NKT cell in combination with auto-DC/Gal in patients with recurrent or advanced head and neck cancer who are refractory or intolerant to standard of therapy.

The dosing regimen involves both a submucosal injection of auto-DC/Gal at 1.0×10^8 cells (fixed dose) followed by tumor-feeding arterial administration of the iPS-NKT cell at one dose levels: 3.0×10^7 cells/m^2, administered at once. The iPS-NKT cell is administered at once five days after the DC/Gal injection. We avoid same-day administration of the investigational product to more than two subjects, and there must be a minimum interval of at least seven days before administering the investigational product to the following subject. We employed the three-by-three (3+3) design for dose escalation in this trial. Ultimately, the maximum tolerated dose (MTD) will be determined based on a total of six subjects with one or less DLT occurrences.

The screening period begins after obtaining the informed consent from each subjects. Based on the results of the screening tests, eligible subjects are registered in this study. One week prior to the start of study treatment, the subject's peripheral blood is collected, and auto-DC/Gal is manufactured (auto-DC/Gal preparation period).

DLT assessment period starts from the injection of auto-DC/Gal to 14 days after the iPS-NKT cell administration. During DLT assessment period, subjects will be carefully monitored under hospitalization. The PI or SI may leave the hospital at his/her own discretion only if the patient does not develop non-hematologic toxicity or hematologic toxicity of Grade 2 or higher and Grade 3 or higher on or after Day 7 of iPS-NKT cell administration, and be able to contact the study site and home doctor of the subjects, if any, in the event of an adverse event.

The Independent Data Monitoring Committee, consisting of members designated by the PI and study

coordinating physicians, monitors the safety of the study and may make recommendations regarding the continuation of the study.

To preliminarily confirm the efficacy of the combination of iPS-NKT cells and auto-DC/Gal, tumor evaluation by contrast-enhanced CT will be performed prior to the start of study treatment (within 4 weeks) and on the first day of the observation period (14 days after the last administration of iPS-NKT cell or on the later of the date when the decision to discontinue administration is made).

Adverse events are observed after obtaining the informed consent until the last day of the observation period (at the time of discontinuation). If, at the end of the observation period for adverse events, an adverse event with an undeniable causal relationship to auto-DC/Gal or iPS-NKT cell has not yet recovered, the patient will be followed until the earliest point of recovery, stabilization, or death of the adverse event, whichever occurs first.

The study flow is shown below (Fig.13).

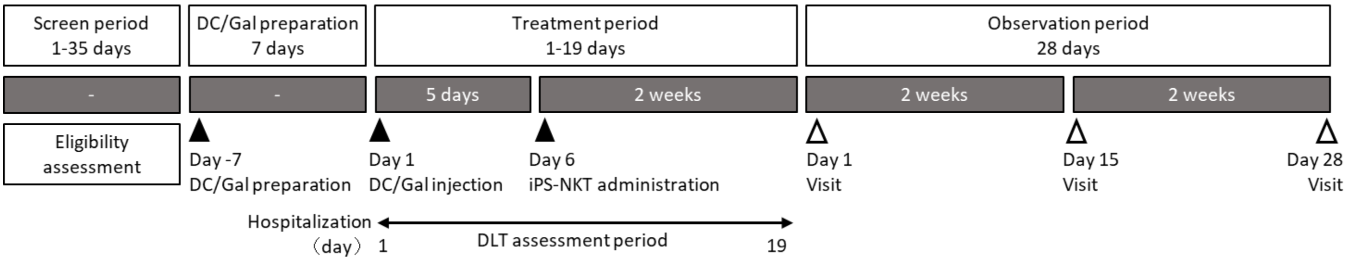

Figure 11 Study Flow

5.2. Dose Limiting Toxicity (DLT)

DLT is defined as the following adverse events observed during the DLT assessment period, in which a causal relationship with auto-DC/Gal or iPS-NKT cell cannot be denied. Grading is determined according to the CTCAE ver.5.0 translated in Japanese by the Japan Clinical Oncology Group (JCOG).

1) Grade 4 or higher hematological toxicities
2) any non-disease-related blood toxicity requiring any transfusion or granulocyte-colony stimulating factor (G-CSF) administration
3) grade 3 or higher non-hematological toxicities (excluding transient clinical laboratory abnormalities, diarrhea, nausea, vomiting, or other manageable systemic symptoms that recovered to grade 2 or below under appropriate treatments)
4) AEs lead to blood transfusion.

If the PI suspects the occurrence of DLTs, the PI can consult the Independent Data Monitoring Committee (IDMC), and the IDMC determines the continuation or termination for the patient. Subjects who do not complete the DLT assessment period for reasons other than DLT will not be considered DLT evaluable cases and then another subject will be supplemented in the same dose cohort.

5.3. Sample size and Study Duration

Sample Size: 2～6 cases

Study Duration: September 2022 – 08/31/2024

Recruitment: October 2022 – 07/14/2024

5.4. Registration of Study Site and Subject

The study sites and subjects will be registered centrally at the Data Center of Chiba University Hospital.

5.4.1. Study site

1) After approval is obtained from the Specified Authorized Regenerative Medicine Committee of the study site, the PI shall fax a copy of the approval letter from the Specified Authorized Regenerative Medicine Committee and a request form for site registration to the Chiba University Hospital Data Center.
2) Chiba University Hospital Data Center will register the study site and send a notification of completion of study site registration to the PI.

5.4.2. Subjects

1） After obtaining written consent, a screening test will be performed.
2） The PI or SI will confirm that subjects meet the selection criteria and do not violate the exclusion criteria regarding the screening test results.
3） The PI or SI will consult with at least two physicians comprising the study coordinating committee regarding the appropriateness of the judgment regarding eligibility. If the subject is finally judged "eligible," the PI, SI, or study coordinator will enroll the subject in the study.
4） The PI, SI, or study coordinator fills in the information necessary for subject registration and faxes it to the Chiba University Hospital Data Center. The PI or SI will confirm the eligibility and start the study treatment once the subject is determined to be eligible. Once a subject has been registered, the registration will not be cancelled. In the event that an error or duplicate registration is discovered, the Chiba University Hospital Data Center will be notified immediately.

In the event that the first dose cannot be administered within the specified period due to factors other than the subject's medical problems including fever caused by temporary infection (e.g., influenza infection), or the quality of DC/Gal or iPS-NKT cells (e.g., the cell count of DC/Gal or iPS-NKT cells was less than the expected dose). In such cases, the subject may be re-registered according to the procedures described in 3) and 4) above after conducting the necessary screening tests again.

5.4.3. Responsible department for registration

Chiba University Hospital Data Center
TEL：043-226-2667　FAX：043-226-2644

5.5. Handling of Patients Who Are Not Registered

Subjects who are not registered for any reason, such as ineligibility, will not be included in the study subject. The PI or SI will explain to the subject that he/she is not eligible for enrollment in the study.

5.6. Dosing schedule and route

Auto-DC/Gal and iPS-NKT cell will be administered according to the dosing schedule from subjects who meet all inclusion and exclusion criteria.

This is a FIH study on the combination therapy of auto-DC/Gal and iPS-NKT cell and will be a 3+3 design with single dose cohort. iPS-NKT cell will be administered 5 days after auto-DC/Gal injection. The number of cells administered will be 1×10^8 cells/treatment of auto-DC/Gal and 3×10^7 cells/m^2/treatment of iPS-NKT cells. The number of dosing should be once for both auto-DC/Gal and iPS-NKT cell, and the dosing of the auto-DC/Gal or iPS-NKT cell in the next subject should be started after an interval of at least 7 days. Auto-DC/Gal should be administered submucosal using a microinjection syringe, and iPS-NKT cell should be administered via a catheter inserted under angiography (Selsinger method) into the tumor-feeding artery.

5.7. Criteria for administration

For subjects enrolled in this study, auto-DC/Gal will be started after confirming that none of the "Criteria for Discontinuation of Administration" are met on the day of auto-DC/Gal or iPS-NKT cell administration.

5.8. Criteria for postpone

If any of the following criteria are met, the PI or SI may postpone the administration of iPS-NKT cells for up to 3 days.

1) When the PI or SI determines that administration of auto-DC/Gal or iPS-NKT cell is inappropriate.
2) All adverse events occurring after the administration of DC/Gal or iPS-NKT cell do not recover to a manageable Grade 2 or Grade 1 or below, or to baseline.
3) Infectious disease requiring treatment develops within 24 hours prior to administration of DC/Gal or iPS-NKT cell, including cases of fever of 38°C or higher.

5.9. Criteria of treatment and study discontinuation for each subject

5.9.1. treatment discontinuation

If any of the following criteria are met, the PI or SI will discontinue administration of DC/Gal or iPS-NKT cell. If administration is discontinued, the subject will be shifted to the observation period from the date when the decision to discontinue administration was made, and testing and assessment will be continued in accordance with the study schedule.

1) DLT occurs after administration of DC/Gal, or all adverse events of Grade 3 or higher that occurred during the study period do not recover or improve to Grade 1 or clinically manageable Grade 2 by the time immediately before administration of DC/Gal or iPS-NKT cell.
2) Subject develops an infectious disease requiring treatment that does not recover or improve to Grade 1, or does not improve to a clinically manageable Grade 2 by the time immediately prior to administration of DC/Gal or iPS-NKT cell.
3) SpO_2 (room air) is maintained below 90%.
4) Grade 2 or higher on the mMRC shortness of breath scale (see Appendix 3) in the examination before administration of DC/Gal or iPS-NKT cells.
5) Requirement for the discontinuation from the subject.
6) Other cases in which the need to discontinue the administration of DC/Gal or iPS-NKT cell to the subject are recognized by the PI or SI.

The PI or SI should record the date of discontinuation, reason for discontinuation, and other necessary information.

In the case of discontinuation of DC/Gal or iPS-NKT cell administration, the "date of discontinuation" is not the date when the event causing the discontinuation occurred or became known, but the date when the PI or SI decided to discontinue.

5.9.2. Study discontinuation

If any of the following criteria are met, the PI or SI will discontinue the study in the subject.

1) When progression of the primary disease requiring concomitant use of prohibited drugs or concomitant therapy is recognized, as judged by the PI or SI.
2) When the subject requests discontinuation of the study.
3) When a subject is found to be ineligible after his/her registration.
4) Other cases in which the PI or SI determines that the subject is unable to receive DC/Gal.

When a subject who meets the criteria for discontinuation is identified after the start of DC/Gal or iPS-NKT cell administration, the PI or SI should explain this to the subject, discontinue the study, and take appropriate

measures.

In the case of discontinuation due to adverse events (including abnormal changes in laboratory test values), necessary examination and observation should be conducted until it is determined that the subject can be discharged or transferred to another hospital. For adverse events for which a causal relationship with DC/Gal or iPS-NKT cell cannot be denied, observation should be continued as much as possible until symptoms recover or become mild, in principle.

The PI or SI should record the date of discontinuation, reason for discontinuation, and other necessary information. In addition, the PI or SI should perform the stipulated items at the time of discontinuation as possible.

In the case of discontinuation, the "date of discontinuation" shall be the date when the PI or SI decides to discontinue the study, not the date when the event that is the reason for discontinuation occurs or becomes known.

5.10. Concomitant drugs and therapy

5.10.1. Permitted concomitant drug/therapy

- Palliative and supportive care for the primary disease
- Palliative radiation therapy for local pain management may be administered during the study if:
  - ➢ The irradiated area covers less than 10% of the total bone marrow
  - ➢ The irradiation field does not include the evaluation lesion
- Any transfusion therapy as a response to adverse events thought to be caused by DC/Gal or iPS-NKT cell. Such adverse events shall be considered DLT.

5.10.2. Record on concomitant drug/therapy

For concomitant drugs and therapies listed in 5.10.3. that were used from the administration of DC/Gal or iPS-NKT cell until the end of the last observation (at the time of discontinuation), record the drug name (trade name or generic name), duration of administration, and purpose of administration. For concomitant therapy, record the name of the therapy, duration of therapy, and purpose of therapy.

5.10.3. Prohibited drug/therapy

In this study, DC/Gal or iPS-NKT cell will not be administered in combination with the following drugs or therapies. If it is necessary to use them clinically, it should be treated as a deviation to avoid immediate risk and reported to the record and the head of the medical institution.

- Corticosteroids (methylprednisolone 10 mg/day or more or equivalent) or immunosuppressive drugs taken or injected, other antineoplastic agents, radiation therapy, or surgery including tumor resection
- Any transfusion therapy (except as noted in 5.10.1).

5.11. Actions to be taken after discontinuation or termination of the study

Subjects who discontinue or terminate the study will be subjected to necessary examination and observation, and appropriate measures will be taken as necessary, until it can be medically determined that the subject can be discharged or transferred to another hospital.

6. Specified cell processed product

6.1.1. Packaging/label of iPS-NKT cell

Gas-exchangeable plastic test tubes containing iPS-NKT cell are stored in a plastic pack capable of supplying CO_2 (AnaeroPack®CO_2) and are further encased in a transport box (TACPack®37) that can be kept at 37°C for heat insulation during transport. In addition, the plastic pack containing the iPS-NKT cell is clearly stated with the following information.

1) For experimental use only
2) Name, title, and address of the person conducting the study
3) Cells, transgenes, or identification code
4) Serial number or serial symbol
5) Storage method and validity period (if necessary to be specified)

An example of iPS-NKT cell product label

iPS-NKT for Clinical Trial
Serial number: NKT-   (Date of melting)
Packaging form/number of tubes (50 mL tubes):
Component cells: Human iPS cell-derived NKT cells
Expiration date: 6 hours after shipment and within 3 hours after preparation
Date of shipment: yyyy/mm/dd, hh:mm
Storage conditions: 37°C under 5% CO_2 (Allowance: 33 - 38℃)
PI: Tomohisa Iinuma, Assistant Professor
Chiba University Hospital 1-8-1 Inohana, Chuo-ku, Chiba-shi, Chiba 260-8677, Japan

6.1.2. Packaging/label of auto-DC/Gal

The auto-DC/Gal-filled micro-injectable syringe is placed in a bag and further packaged in a shipping box (TACPack®0208Re-2A-SC) that can be maintained at room temperature (4~25°C) for temperature control during transportation. In addition, the following is clearly stated on the bag containing the auto-DC/Gal.

1) Serial number: A2023xxx-000x-DC
2) Manufacturing Date and Time: yyyy/mm/dd, hh:mm
3) Expiration for administration: 2hrs after shipment
4) For experimental use only

Example of label

For use only on clinical trial A2023xxx-000x-DC<br>Packaging form/number of tubes: Micro-injectable syringe/ 1<br>Date of shipment: YYYY/MM/DD, HH:MM<br>Expiration for use: 2hours after shipment

6.2. Administration and dispensing methods

6.2.1. Administration and dispensing methods of iPS-NKT cell

1) After subject registration, the PI notifies RIKEN of the administration date, dose, etc.
2) RIKEN delivers the iPS-NKT cell to the PI of the study site prior to the administration of the iPS-NKT cell, based on the notification from the PI of the administration date, dosage, etc.
3) The administrator of the iPS-NKT cell line at the study site will manage the iPS-NKT cell in accordance with the "Procedure for Management of Specified Cell Processed Products" provided by the PI through the head of the study site
4) The timing and method of dispensing shall be in accordance with the separately prescribed procedure.

6.2.2. Management method of auto-DC/Gal

1) After the subject registration, the PI informs the Manufacturing Department of Chiba University Hospital Center for Advanced Medicine of the administration date, dose, etc.
2) The Manufacturing Department of the Chiba University Hospital Center for Advanced Medicine delivers auto-DC/Gal to the PI prior to administration, based on the information from the PI regarding the administration date, dosage, etc.
3) The administrator of the auto-DC/Gal at the study site manages the auto-DC/Gal in accordance with the "Procedures for the Management of Specified Cell Processed Products" provided by the PI through the head of the study site.

7. Method and Timing of Observations/tests/assessments

7.1. Study Schedule and Procedure

The schedule for conducting observations, tests, and assessments is shown in Table 19. The PI or SI should conduct the observations, tests, etc. according to the schedule. Procedures that can be performed by study coordinators, such as subject background investigation and blood tests, may be performed by the study coordinators under the supervision of the PI.

Table 16 Study Calendar

		Screening	DC/Gal Prep.	Treatment Period						Observation Period h)			Termination
Day		−42～−8	-7	1	6	7	8	13	19	1	15	28	—
Allowance (days)				+3	+3	—	—		±3	±3	±7	±7	+7
Informed consent	●												
Demographics		●											
Eligibility		●											
Pregnancy test a)		●										●	●
Infection test b)		●											
Chest X ray		●						●		●	●	●	●
ECG		●											
Enhanced CT		●									●i)		●
DC/Gal preparation			●										
DC/Gal injection				●									
iPS-iNKT cell administration c)					●								
CBC/Laboratory test		●		●	●			●		●	●	●	●
Coagulation test		●			●								
Urology		●		●	●					●	●	●	●
Vital sign d)		●	●	←									→
Body weight		●		●	●			●		●	●	●	●
ECOG PS		●		●	●					●	●	●	●
mMRC				●	●								
PK/PD e)					●	●		●					
Immune cell assessment f)				●	●		●	●		●			
AEs		←											→
DLTs under hospitalization g)				←					→				
HLA test		●											
Anti-HLA antibody		●			●					●			●

(a) Perform only in women of childbearing potential. Perform a urine qualitative test.

(b) Infectious disease tests include HIV antibody, HTLV-1 antibody, HBs antigen, HBs antibody, HBc antibody, and HCV antibody. If the HBs antigen is negative and the HBs antibody test or HBc antibody test is positive, the HBV-DNA quantitative test is performed. If the HCV antibody test is positive, the HCV-RNA quantitative test is performed.

(c) Secure the route of administration of the investigational product 1 week prior to the first administration date (allowance based on 1 week prior to the first administration date: -14 to +7 days). As described in the Administration Method section, there are two methods of administering the investigational product (method of securing the route of administration): the port method (e.g., for maxillary cancer) or the Selsinger method. In the case of the port method, a port is implanted under local anesthesia in an IVR room, and the investigational product is administered through the implanted port after confirming that there are no signs of infection in the wound. In the case of the Selsinger method (tongue cancer, etc.), the tip of the catheter is guided into the tumor-feeding artery in the IVR room, and the investigational product is administered directly.

(d) Vital signs shall include body temperature, pulse rate, respiratory rate, SpO_2 , and blood pressure (systolic and diastolic) , and additional tests (chest X-ray, hematological and serological tests, blood gas tests, etc.) may be added as necessary based on the judgment of the PI or SI .

(e) The blood collection schedule for PK/PD and blood cytokine measurements will be as shown in **Table** 20

(f) Isolate PBMCs from blood samples and stain for cell surface markers.

(g) During the DLT evaluation period (from the first dose to Cycle 2 Day 14) and for 7 days after the third dose of the investigational product, the subject will be carefully monitored for safety by hospitalization in principle. In addition, the results of laboratory tests and medical examinations after the seventh day of administration of the investigational product (Day 8 of each cycle) should show that no Grade 2 or higher non-hematological toxicity or Grade 3 or higher hematological toxicity has occurred, and that the site and home health care facility can be contacted in the event of an adverse event and that the patient can be contacted promptly in an emergency. The PI or SI may stay overnight or leave the hospital at his/her own discretion only when a system is in place to contact the site and home health care facility in the event of an adverse event and to contact the patient promptly in an emergency.

(h) Cycle 1 Day 14 contrast CT scan (tumor evaluation) will be performed prior to Cycle 2 transition.

(i) If the administration of the investigational product is discontinued, the observation period will begin at the later of the end of the cycle of administration of the investigational product immediately prior to discontinuation (2 weeks after the last administration of the investigational product) or the date on which the decision to discontinue administration was made.

Table 17 Blood collection schedule for PK/PD measurement

	Before DC/Gal injection	Before iPS-NKT administration	Post-dose		
Time point (hour)	—	—	1	24	168
Allowance (min)	—	—	±15	±30	±60
Blood collection for PK/PD	—	●	●	●	●

7.1.1. Screening test

After obtaining consent, the screening tests are initiated. The PI or SI will perform the following screening tests and enroll patients who meet the selection criteria and do not violate the exclusion criteria. The test items shall be as described below.

- Body weight (usu. one's own)
- Vital signs[4)]
- ECOG PS
- Hematological examination
- Blood biochemical tests (including HbA1c)
- Coagulation test
- Urinalysis
- Pregnancy test (for women of childbearing potential only)
- Infectious disease test (HBs antigen, HBs antibody, HBc antibody, HCV antibody, HIV antibody or HTLV-1 antibody)
- 12-lead ECG test
- Chest X-ray
- Contrast-enhanced CT scan (vascular and tumor evaluation)
- HLA testing (up to area 2 by Luminex method)
- Anti-HLA antibody test

7.1.2. Subject Information

The following subject information should be recorded at the time consent is obtained or at the time of the screening test.

1) Date Consent was obtained
2) Subject Identification Code
3) Gender
4) Age
5) Stature
6) Previous history/complications
7) Diagnostic name
8) Organizational type
9) Staging
10) Body surface[6)]
11) Allergies

---

4) Vital signs shall include temperature, pulse rate, respiratory rate, SpO_2 , and blood pressure (systolic and diastolic).

6) Fujimoto formula (Body surface area (m^2) = Weight $(kg)^{0.444}$ x Height $(cm)^{0.663}$ x 88.83/10000) is used

12) Past cancer treatment and surgery

13) Alcohol and smoking history

7.1.3. Observation, test, assessment measurements

Blood Biochemistry Tests at each timing are listed below. In addition, vital signs and adverse events will be checked daily during the hospitalization period. Details of measurements are shown in Table 21. Additional tests (chest X-ray, hematological and serological tests, blood gas test, etc.) may be added as necessary for safety or other reasons at the discretion of the PI or SI.

Table 18 List of laboratory test measurement

infectious disease test<br>(*only at screening)	HBs antigen, HBs antibody, HBc antibody, HCV antibody, HIV antibody or HTLV-1 antibody, or<br>HBV-DNA quantification (*Added when HBs antigen negative and HBs or HBc antibody positive)<br>HCV-RNA quantification (*Additional when HCV antibody positive)
HLA test<br>(*only at screening)	HLA genes (HLA-A, B, C, DRB1, DQB1, DPB1)
hematological examination	Red blood cell, hemoglobin, hematocrit, platelet count, white blood cell count, and Leukocyte fractions (neutrophils, lymphocytes, monocytes, eosinophils, basophils)
Blood Biochemistry Test<br>(*HbA1c only at screening)	Total protein, albumin, total bilirubin, AST, ALT, ALP, LDH, BUN, creatinine, uric acid, Na, K, Cl, Ca, P, amylase, HbA1c
coagulation test	PT-INR, aPTT
urinalysis	pH, sugar, protein, urobilinogen, ketones, occult blood
Blood cytokine assay<br>(Using Bio-Plex Pro human cytokine GI 8-Plex A panel)	GM-CSF, IFN-γ, IL-2, IL-4, IL-6, IL-8, IL-10, TNF-α
Immuno-cell fractionation test	T-cell count (cells/mL)<br>NK cell count (cells/mL)<br>NKT cells (cells/mL)<br>CD4+ T cell count (cells/mL)<br>CD8+ T cell count (cells/mL)<br>Naive CD4+ T cell count (cells/mL)<br>Central memory CD4+ T cell count (cells/mL)<br>Number of effector memory CD4+ T cells (cells/mL)<br>Number of effector CD4+ T cells (cells/mL)<br>Naive CD8+ T cell count (cells/mL)<br>Central memory CD8+ T cell count (cells/mL)<br>Number of effector memory CD8+ T cells (cells/mL)<br>Number of effector CD8+ T cells (cells/mL)<br>PD-1+ CD4+ T cell percentage (in CD4+CD3+ T cells)<br>PD-1+CD8+ T cell percentage (in CD8+CD3+ T cells)<br>PD-1+NKT cell percentage (% in NKT cells)<br>PD-1+ NK cell percentage (% in NK cells)<br>WBC count (pcs/mL)<br>Lymphocyte count (cells/mL)
Anti-HLA antibody test	Class I and II determination, Class I and II positive antibodies, DSA

7.1.3.1. Cell preparation period (Day -7)

One week prior to DC/Gal administration, apheresis is performed after checking vital signs to create autologous DC/Gal.

7.1.3.2. Treatment period

Before the injection of auto-DC/Gal on Day1, the following are performed

- Body weight
- Vital signs
- ECOG PS
- mMRC
- Hematological test
- Blood Biochemistry Test
- Urinalysis
- Immune cell repertoire analysis
- Confirmation that at least one month has passed since the previous treatment

Day 6, the following are performed before iPS-NKT cell administration

- Body Weight
- Vital signs
- ECOG PS
- mMRC
- Hematological test
- Blood Biochemistry Test
- Coagulation test
- Urinalysis
- Immune cell repertoire analysis※
- Anti-HLA antibody test
- PK/PD blood collection (1 hour after iPS-NKT administration)

Day7 (24 hours after iPS-NKT cell administration) : PK/PD blood collection.

Blood samples for immune cell repertoire analysis* will be taken on Day 8.

The following will be performed on Day13.

- Body wight
- Vital sign

- Chest X-ray
- Hematological
- Blood Biochemistry Test
- Immune cell repertoire analysis※
- PK/PD

※Immune cell repertoire test: PBMCs are isolated from blood samples and stained for cell surface markers. Concentration and percentage of cancer-related immune cell fractions, mainly T cell fractions and NKT cell markers, in peripheral blood are calculated using flow cytometry.

- Adverse event evaluation
  (During the DLT evaluation period, if any disease or symptom is suspected to fall under the definition of DLT (see 5.2. Dose Limiting Toxicity (DLT)), the PI will immediately request the Independent Data Monitoring Committee to review the appropriateness of the decision and decide whether the study can continue in the patient.)

7.1.3.3. Observation Period

On Days 1, 15, and 28 of the observation period, the following will be performed

- Body Weight
- ECOG PS
- Hematology
- Blood Biochemistry Test
- Urinalysis
- Pregnancy test (for women with childbearing potential only, on Day 28)
- Chest X-ray
- Adverse event evaluation
- Contrast-enhanced CT scan (tumor evaluation, on Day 15)
- Anti-HLA antibody test (only on Day 1)
- Immune cell repertoire analysis (only on Day 1)

7.1.3.4. Study Discontinuation

Within 7 days of the decision by the PI or SI to discontinue the study, the following will be performed as possible. However, if post-treatment is to be initiated, the following must be performed prior to the start of post-treatment.

- Body Weight

- ECOG PS
- Hematology
- Blood Biochemistry Test
- Urinalysis
- Pregnancy test (for women with childbearing potential only)
- Chest X-ray
- Adverse event evaluation
- Contrast-enhanced CT scan (tumor evaluation)
- Anti-HLA antibody test

7.1.3.5. Tumor Evaluation

As a secondary endpoint, imaging studies will be performed to determine the efficacy of combination use of DC/Gal and iPS-NKT cells on the tumor.

At screening period, contrast-enhanced CT will be performed to detect the presence of evaluable tumor from the head and neck to the pelvic region.

After the start of iPS-NKT cell administration, target lesions from the head and neck to the pelvic region will be confirmed by imaging examination on the first day of the observation period (28 days after iPS-NKT cell administration or 15 days after the decision to discontinue administration, whichever comes later) and at the time of discontinuation. Even in subjects with no target lesions in the brain or extremities at screening period, the presence or absence of brain or bone metastases will be evaluated by imaging tests according to clinical symptoms.

Contrast-enhanced CT performed in this study will be performed in the same modality setting as much as possible for evaluation over time.

Efficacy evaluation will be performed according to the RECIST 1.1 Japanese translation of the JCOG version (see Appendix 1). When imaging for efficacy evaluation is performed, the PI or SI should promptly perform the evaluation and record the evaluation results.

7.1.3.6. Pregnancy Outcome Study

If a female subject or male subject's partner is found to be pregnant during the study period, she will be followed until the outcome of the pregnancy is known, regardless of the study period.

7.1.3.7. Follow-up after study completion

After the completion of the study, the subjects will be treated in the standard clinical practice.

8. Handling of Adverse Events, Quality Defects, etc.

8.1. Definition of Adverse Event and Quality Defect

Adverse events are any unwanted or unintended symptoms (including abnormal changes in laboratory values) or illnesses that occur after DC/Gal or iPS-NKT cells have been administered, regardless of causal relationship to DC/Gal or iPS-NKT cells.

Preexisting conditions (complications that existed prior to the study period) will not be categorized as adverse events unless they worsen or increase in frequency during the study period. However, if a complication worsens after the administration of DC/Gal or iPS-NKT cells, it shall be categorized as an adverse event, and the date when the worsening is confirmed shall be recorded as the date of occurrence of the adverse event.

Quality defect refers to the failure of the function of the test product, etc., or adverse effects of the cells on the human body, or any other widely unfavorable conditions, regardless of whether the failure is due to the product itself, delivery, storage, or use of the product.

8.2. Definition of Disease, etc.

Disease, etc. means any disease, disability, or death or outbreak of infectious disease that is suspected to be caused by the provision of regenerative medicine, etc. (hereinafter referred to as "occurrence of disease, etc."). Diseases, etc. that need to be reported to the Specified Authorized Regenerative Medicine Committee and the Minister of Health, Labour and Welfare should be reported according to "8.8. Reporting of diseases, etc. to the Specified Authorized Regenerative Medicine Committee and the Minister of Health, Labour and Welfare".

8.3. Definition of Serious Adverse Event

A serious adverse event is defined as any of the following:

1) Death
2) Those that may lead to death
3) In need of hospitalization or prolonged hospitalization for treatment
4) Disability
5) Those that may lead to disability
6) Those that are as serious as those listed in 3) to 5) above.
7) Congenital diseases or anomalies in later generations

Note that "hospitalization" in 3) does not include reexamination, hospitalization for follow-up or extended hospitalization, and hospitalization solely for the purpose of performing treatment or tests that were planned prior to the start of the study (e.g., scheduled surgery or tests) during the study. (However, any new occurrence

during such hospitalization will be treated as an adverse event.)

8.3. Response to Subjects in the occurrence of the Adverse Events or Quality Defects

When an adverse event or quality defect is observed, the PI or SI will immediately take appropriate measures and inform the subject if the administration of DC/Gal or iPS-NKT cells is discontinued or if treatment for the adverse event should be taken. If an adverse event or symptom is recognized by the PI or SI as requiring more specialized consultation, the PI or SI will promptly collaborate with an appropriate specialist and can take measures including extension of the hospitalization period, if necessary.

If an adverse event with an undeniable causal relationship to DC/Gal or iPS-NKT cells has not yet recovered at the time of completion or discontinuation of the study, observation should be continued as much as possible until recovery or attenuation of symptoms, in principle. However, this does not apply when the PI or SI determines that the influence of the study have disappeared, the subject's safety is sufficiently ensured, and further follow-up is not necessary.

8.4. Reportable Adverse Events and Quality Defects

1) All adverse events occurring from the date of consent to the last day of the observation period (at the time of discontinuation) should be reported regardless of whether or not they are causally related to DC/Gal or iPS-NKT cells, and the patients should be observed until they recover from the adverse events or the last day of the observation period (at the time of discontinuation). All adverse events judged to be causally related to DC/Gal or iPS-NKT cells should be reported until the end of the study period.
2) All information on quality defects occurring during the study period shall be reported.

8.5. Reporting Procedures for Adverse Events and Quality Defects

All adverse events and quality defects occurring during the above period shall be documented by the PI or SI without discrepancy in the medical record and case report form.

8.6. Description required for evaluation of adverse events and quality defects

8.6.1. Adverse event

1) Description of the adverse event
   Generally, the name of the adverse event should be recorded by the name of the diagnosis and the name of the disease (illness). If the name of the diagnosis or disease cannot be identified, or if it is deemed appropriate not to use the name of the diagnosis or disease by the PI or SI, the clinical symptoms or signs should be used as the name of the adverse event.

2) The date of occurrence
3) The date of outcome
4) Outcome: recovery, mild recovery, recovered but with sequelae, not recovered, death, unknown
5) Measurement (administration of DC/Gal or iPS-NKT cell): no change, discontinue, pending, not applicable
6) Other response: none, drug therapy, others
7) Seriousness: Non-serious, serious
8) Severity: Severity is judged based on CTCAEv5.0 JCOG version.
9) Causal relationship with DC/Gal or iPS-NKT cells: No relation, relation cannot be ruled out

8.6.2. Quality defects

1) The type of quality defect
2) The date the quality defect is confirmed
3) The date of the incident
4) Serial number of DC/Gal or iPS-NKT cells in which quality defects occurred
5) Possible causes and the circumstance of quality defects
6) Measures against quality defects
7) Whether there are any adverse events due to the quality defects
   ① Yes: type of the adverse event
   ② No: Possibility of the occurrence of severe adverse event

8.6.3. Causal relationship between adverse events and DC/Gal or iPS-NKT cells, and recoverability of adverse events

Recovery from an adverse event is defined as the absence of the adverse event or restoration to the state prior to administration. The causal relationship between the adverse event and DC/Gal or iPS-NKT cells should be determined by taking into consideration the subject's general condition, complications, concomitant medications/adjunctive therapies, and time-related factors.

No causal relationship:

when there is a stronger indication that the adverse event is due to other causes other than those listed below or other causes.

A causal relationship cannot be ruled out:

A temporal relationship is observed between the administration of DC/Gal or iPS-NKT cells and the onset of the event, and the event attenuates with time after the discontinuation of DC/Gal or iPS-NKT cells, but recurs or worsens with subsequent re-administration, or no obvious confounding risk factors such as the subject's general condition, complications, concomitant medications, or

concomitant therapies.

8.7. Measures to be taken in case of outbreak of disease, etc.

1) When investigators become aware of an outbreak of disease suspected to be caused by DC/Gal or iPS-NKT cells, he/she shall promptly report it to the PI and the study coordinating physician.
2) The PI shall report to the hospital director using Appendix Form-C (hospital form).
3) The hospital director or the study coordinating physician shall instruct the PI to take necessary measures such as discontinuation of the regenerative medicine, etc.
4) The hospital director or the PI shall promptly notify the specified cell-processing manufacturer (Chiba University Hospital Center for Advanced Medicine and RIKEN) that produced the DC/Gal and iPS-NKT cells of the occurrence of the situation and the measures taken.

8.8. Reporting of disease, etc. to the Specified Authorized Regenerative Medicine Committee and the Minister of Health, Labour, and Welfare

1) When a hospital director becomes aware of any of the following diseases, etc., he/she shall report it to the Specified Authorized Regenerative Medicine Committee and the Minister of Health, Labour and Welfare in accordance with Table 1.
2) Report to the Specified Authorized Regenerative Medicine Committee using Appendix Form 1 of "Report on Diseases, etc." (related to Article 35 of the Ministerial Ordinance). Reports shall also be submitted to the Minister of Health, Labour and Welfare through jRCT.

Table 1

Severity of disease etc.	Entity to be reported	Deadline
① Death<br>② Case that may lead to death	The Specified Authorized Regenerative Medicine Committee<br>The Minister of Health, Labour, and Welfare	Within 7 days
① Cases requiring admission to a medical institution for treatment or prolonged hospitalization<br>② Disability<br>③ Cases that may lead to disability<br>④ Cases that are serious<br>⑤ Congenital disease or abnormality in later generation	The Specified Authorized Regenerative Medicine Committee<br>The minister of health, labour, and welfare	Within 15 days
① Occurrence of disease, etc. due to an infectious disease that is suspected to be caused by the provision of regenerative medicine, etc.	The Specified Authorized Regenerative Medicine Committee	Every 60 days from the date of submission of the plan for providing regenerative medicine to the Minister of Health, Labour and Welfare, and within 10 days after the expiration of designated period

8.9. Annual reports to the Specified Authorized Committee for Regenerative Medicine and the Minister of Health, Labour and Welfare

1) The hospital director shall report to the Specified Authorized Regenerative Medicine Committee on the following items annually, starting from the date of submission of the plan for the provision of regenerative medicine to the Minister of Health, Labour and Welfare, within 90 days after the expiration of the designated period.
   - Number of subjects who have received this regenerative medical treatment
   - The occurrence and progress of diseases, etc. related to this regenerative medical treatment
   - Evaluation of the safety and scientific validity of this regenerative medicine
   - Matters concerning involvement in this regenerative medicine, etc. as prescribed in each item of Article 8-8(1) on the Act on the Safety of Regenerative Medicine (ASRM)
   - Status of occurrence of incompliance to this Ministerial Ordinance or the plan for providing regenerative

medicine, and subsequent actions taken

2) After hearing opinions from the Specified Authorized Regenerative Medicine Committee, the hospital director shall report to the Minister of Health, Labour and Welfare on the following items annually, starting from the date of submission of the plan for provision of regenerative medicine within 90 days after the expiration of designated period.
    - Name of the Specified Authorized Regenerative Medicine Committee
    - Opinion of the Specified Authorized Regenerative Medicine Committee, on the appropriateness of continuation of this study.
    - Number of subjects who have received this regenerative medical treatment

8.10. Report of Serious Incidents to the Minister of Health, Labor and Welfare

1) In the event of a situation that may have a significant impact on ensuring the safety of the specified cellular processed products (a serious incident), the specified cellular processed product manufacturer must take the necessary measures and promptly report to the study site (Chiba University Hospital) and the Minister of Health, Labour and Welfare.
2) When storing Specified Cellular Processed Products in which a serious incident has occurred, the Specified Cellular Processed Products must be classified and stored for a certain period of time, and then properly processed.

8.11. Management of incompliance issue

When the Hospital Director or the PI becomes aware that the study is not incompliance with the Act on the Safety of Regenerative Medicine (ASRM) or the Regenerative Medicine Provision Plan, he/she shall promptly report to the Hospital Director to that effect.

In addition, when a particularly serious incompliance issue is found, the Hospital Director or the PI shall promptly obtain the opinion of the Specified Authorized Committee for Regenerative Medicine.

9. Endpoints

9.1. Primary endpoint

Dose Limiting Toxicity (DLT)

【Rationale】
The primary purpose of this study is to evaluate the tolerability of the combination therapy of DC/Gal and iPS-NKT cells, based on the DLT expression rate, which is a typical endpoint in Phase I studies of drugs.

9.2. Secondary endpoints

【Efficacy】

・ Response rate (RECIST ver.1.1)

・ Disease control rate (Subjects reached on CR, PR, or SD based on RECIST ver.1.1)

【Safety】

・ Adverse event (Type, frequency, and severity)

・ Changes in Laboratory Values

【Rationale on secondary efficacy endpoints】

Since this is an FIH study of DC/Gal and iPS-NKT cell combination therapy for malignant tumors, the evaluation of efficacy was set as a secondary endpoint.

【Rationale on secondary safety endpoints】

Secondary safety endpoints are established to evaluate in as much detail as possible all adverse events that occurred in this study, in addition to the DLT evaluation.

9.3. Exploratory endpoints

- Remaining iPS-NKT cells in peripheral blood at 1 week after iPS-NKT cell administration
- Dynamics measurement (iPS-NKT cell concentration in peripheral blood on day 7 after iPS-NKT cell administration)

【Rationale】

The exploratory endpoints were established to elucidate the mechanism of action of iPS-NKT cells in the human body and the effects of iPS-NKT cells on the immunokinetic and function of the subject.

9.4. Exploratory Immunological evaluation

The following immunological analyses will be performed using blood samples for immunological testing.

・ Immune cell repertoire analysis

・ Omics analysis (including genetic testing) in peripheral blood immune cells

Omics analysis in peripheral blood immune cells ("Omics analysis") will be performed to explore the molecular biological effects of iPS-NKT cells on the differentiation and function of peripheral blood immune cells when administered into the tumor-trophoblastic artery of head and neck cancer patients.

Omics analysis will be performed using residual blood samples (approximately 12 mL) collected for

pharmacokinetic and immune cell repertoire analysis of peripheral blood after obtaining consent for the Omics analysis from each subject. When analyzing the specimens, personal identifiable information will be deleted, new IDs will be assigned, and the specimens will be anonymized, and analyzed at Chiba University and RIKEN, as necessary. The genetic information revealed by the analysis will not be disclosed to the subjects in principle, since the usefulness of the genetic information is not clear at the time the research is initiated. However, genetic information may be disclosed after deliberation by the Bioethics Review Committee if the usefulness of the genetic information becomes clear, or if a relationship to other serious diseases is discovered by chance, and after considering the wishes and benefits of the subject and their relatives. The handling of samples and data shall be in accordance with "16.1. Storage of Samples" and "16.2. Disposal of Specimens.

10. Statistical Considerations

The statistical analysis plan for this study is summarized below. The details of the statistical analysis plan are described in the statistical analysis plan. The outline of this study plan may be revised in the statistical analysis plan if the definition of the primary endpoints or the analysis methods are changed.

10.1. Population to be analyzed

10.1.1. Safety analysis population and DLT evaluation population

Subjects those who registered in the study and treated with the auto-DC/Gal will be the target population for safety analysis. However, cases of non-compliance with the Regenerative Medicine Act will be excluded from the safety analysis population. Of the safety analysis population, all subjects who could not be appropriately evaluated during the DLT evaluation period for reasons other than the auto-DC/Gal or the iPS-NKT cell-related toxicity will be excluded from the DLT evaluation population. In the event that a subject is excluded from the analysis, a new subject will be added to the cohort.

10.1.2. Largest analyzed population (full analysis set：FAS)

All subjects those who registered in the study and have received at least one dose of the auto-DC/Gal or the iPS-NKT cells and for whom efficacy data are available will be the Full Analysis Set (FAS). However, subjects for whom baseline data were not obtained and subjects with serious violations of the study protocol (e.g., failure to obtain consent) will be excluded.

10.1.3. Subject population conforming to the research protocol (per protocol set：PPS)

Subjects without the following serious violations of the provisions of the research protocol, including

study methods and concomitant therapies.

Violation of selection criteria, exclusion criteria, concomitant use of prohibited drugs, or concomitant use of prohibited therapies

10.2. Sample size and Rationale

Sample size：2～6

【Rationale】

Since the purpose of this study is to evaluate the tolerability of the combination therapy of the auto-DC/Gal and the iPS-NKT cells, as well as to explore the safety and efficacy of the combination therapy, a significance test for validation purposes will not be performed. We employed a 3+3 design for this study, and the maximum number of patients to be analyzed in this study is set at 6 patients. The minimum number of cases was set at 2, since the study was terminated due to the occurrence of two consecutive cases of DLT in the first dose.

10.3. Case Handling

In principle, the study coordinating physician and the statistical analyst will decide on the handling of the registered cases after consultation with the study coordinating physician and the statistical analyst. The study coordinating physician and the statistical analyst will also discuss and decide on the handling of cases in the event of new problems.

10.4. Data Handling

In principle, the handling of data during data tabulation and analysis shall be as follows. In case of doubt, the statistical expert and the study coordinating physician will discuss and decide before fixing the data. Missing values will not be supplemented.

10.5. Statistical analysis items and analysis plan

Analysis will be performed after the auto-DC/Gal or the iPS-NKT cell administration is completed in all cases and data are fixed.

For the safety analysis, a frequency table will be created for each System Organ Class (SOC) and the Preferred Term (PT) in MedDRA/J for the occurrence of DLT in the DLT evaluation target population. In addition, the following analyses in the safety analysis target population will be conducted as secondary evaluations.

For the efficacy evaluation, analyses will be performed on the Full Analysis Set (FAS) and on the analysis population consistent with the study protocol (PPS).

Details of the statistical analysis will be specified in a separate statistical analysis plan to be prepared prior to data fixation.

Primary endpoint

- Dose-Limiting Toxicity (DLT) Incidence Rate

Secondary endpoints

【Safety】

- Occurrence of adverse events (type, frequency, severity, etc.)
- Changes in laboratory values

【Efficacy】

- Response rate (RECIST ver. 1.1)
- Disease control rate (RECIST ver. 1.1)

Exploratory endpoints

- Concentration of iPS-NKT cells in peripheral blood
- Immune cell fractionation (T cell fractionation, NKT cell markers, etc.)
- Omics analysis of peripheral blood immune cells

10.5.1. Subject Background Analysis

The distribution and summary statistics of the subject background data in each analyzed population are calculated. For nominal variables, the frequency and percentage of categories are shown for each group. For continuous variables, summary statistics are calculated.

10.5.2. Safety and Efficacy Analysis

10.5.2.1. Primary Analysis

In the target population for DLT evaluation, the number of cases of DLT occurrence per dose and its percentage and 95% confidence interval will be calculated based on the definition of DLT.

10.5.2.2. Secondary analysis

The following secondary endpoints will be analyzed for the purpose of providing supplementary discussion to the main analysis results. For the safety secondary endpoints, frequency tables will be created for

all adverse events that occur after the auto-DC/Gal or the iPS-NKT cell administration for each the system organ class (SOC) and the preferred term (PT) in MedDRA/J. Similar frequency tables will be created by dose, severity, causal relationship to the auto-DC/Gal or the iPS-NKT cells, and the CTCAE Grade. Summary statistics will be calculated for Blood Biochemistry Test results. In addition, summary statistics of the difference between baseline and each time point will be calculated. A trend chart of laboratory values will also be created. For the efficacy secondary endpoints, the response rate and disease control rate based on RECIST v.1.1 evaluated by the PI or SI will be calculated, and the 95% confidence interval for these rates will also be calculated. Because this is an exploratory study, the P-values and confidence intervals for hypothesis testing obtained from statistical analysis are intended to identify and estimate meaningful variation and are not conclusive as to the presence or absence of efficacy. Other items and details are described in the statistical analysis plan.

10.5.3. The interim analysis

No interim analysis will be performed in this study.

10.6. Independent Data Monitoring Committee

The Independent Data Monitoring Committee shall consist of malignancy experts who are independent from the project sponsor, and shall deliberate on the following items regarding the contents reported by the project sponsor.

1. At the request from the PI, the committee will discuss and provide opinions on the presence or absence of a causal relationship between the adverse event and the auto-DC/Gal or the iPS-NKT cells, and on the validity of the known/unknown judgment. In particular, the Committee will make the final judgment regarding the applicability of the adverse event to DLT, such as whether the adverse event is caused by the product or by aggravation of the underlying disease.
2. After evaluating all adverse events and other safety information, including DLT, the Committee will decide on whether or not to continue the study.
3. Determine whether the research protocol needs to be amended or revised, and whether the Informed Consent Form needs to be revised, in response to the above matters.

The method of requesting the Data Monitoring Committee to deliberate, the method of deliberation, and the method of notification of the results of deliberation will be based on the Data Monitoring Committee Standard Operating Procedures to be developed separately.

10.7. Final Analysis

After the follow-up period of the last subject, analysis will be conducted after the data are obtained

and all data are fixed. The statistical analyst will compile an "Analysis Report" and submit it to the study coordinating physician and the project manager. The study coordinator will summarize the contents of the analysis report and prepare a "summary report" summarizing the overall conclusions of the study, problems, interpretation and discussion of the results, and future policies mainly from a clinical perspective, which will be approved by the PI.

11. Compliance and deviation from the research protocol

1) The PI or SI shall conduct the study in compliance with the study protocol.
2) In case of deviation from the research protocol, the PI or SI shall record the details of all deviations and the reasons.
3) When deviating from the protocol in order to avoid immediate emergency to subjects or for other unavoidable medical reasons, the PI shall immediately submit a document describing the details of the deviation and the reasons for it to the head of the study site. Also, the contents of the document shall be promptly reported to the Specified Authorized Committee for Regenerative Medicine via the head of the study site

12. Changes to the research protocol, case report form, or analysis plan

12.1. Revision of research protocols and case report forms

When revising the research protocol and case report form, the following procedure shall be followed.

1) When the PI becomes aware of matters concerning the quality, efficacy, and safety of the auto-DC/Gal or the iPS-NKT cells, or other information important for the proper conduct of the study, the PI shall revise the relevant study protocol, as necessary. Revision history should be recorded and retained at the time of revision.
2) The PI shall revise the case report form as necessary in conjunction with the revision of the study protocol or for other reasons. Revision history should be recorded and retained at the time of revision.
3) The PI shall promptly submit the revised study protocol and the revised case report form to the head of the study site, and promptly submit to the Specified Authorized Regenerative Medicine Committee and the Bioethics Review Committee via the head of the study site.
4) The same procedure should be followed when the study protocol and case report form are revised within the scope acceptable to the PI, as directed by the head of the study site based on the opinions of the Specified Authorized Regenerative Medicine Committee and the Review Committee.

12.2. Change in statistical analysis plan

If the statistical analyst changes the content of the statistical analysis plan, the statistical analyst shall

include all changes in the statistical analysis report of this study. In addition, the circumstances of any changes made to the statistical analysis plan shall be recorded.

13. Study Discontinuation, suspension, or termination

13.1. Criteria for discontinuation or suspension of the study

The PI will decide with the study coordinating physician to discontinue or suspend the study when any of the following information is obtained and it is considered difficult to proceed with the study.

1) If it becomes difficult to ensure the safety of the study due to new safety information or information on serious adverse events concerning the auto-DC/Gal or the iPS-NKT cells.
2) The site of study seriously deviates from the study protocol and no improvement is seen.
3) If DLT occurs in two or more subjects; subjects who do not have DLT will be promptly transferred to the observation period and the study will be terminated as soon as all subjects have completed all of the study procedures.
4) If any other new information is obtained during the course of the study that may require discontinuation or suspension of the study.

13.2. Procedures for discontinuation or suspension of the study

If the PI decides to discontinue or suspend the study after consultation or discussion with the IDMC, etc., the PI shall promptly notify the head of the study site in a document with the reasons for the discontinuation or suspension in detail. In addition, the subjects undergoing treatment will be promptly informed, and appropriate measures such as changing to an appropriate treatment will be taken, and tests to ensure the safety of the subjects will be conducted.

13.3. Study completion

Upon completion of the study, the PI shall notify the head of the study site in document that the study has been completed and report a summary of the study results in document.

14. Data Management

14.1. Data management procedure

Detailed procedures for data management shall be described in the data management plan.

14.2. Data collection

The PI and SI shall prepare the case report form. When making changes, corrections, or additions to the contents of the case report form, the PI or SI should contact the Data Center using the case report form, and record all such changes, corrections, or additions as electronic information. When a case report form is prepared by a SI or when a study collaborator transcribes a case report form from the source documents (source data), the PI shall check the contents of the case report form before it is submitted to the Data Center. The PI shall save the final electronic case report form on an electronic medium (e.g., CD-R, etc.). The PI shall ensure the legibility and preservation of the electronic case report form.

When using the EDC system, the site should receive training in EDC and should refer to a separate input manual for details on how to input data.

14.3. Specification of materials that are directly described in the case report and that should be interpreted as source documents (source data)

The following documents and others shall be considered source documents (source data) in this study.

1) Records related to consent of subjects and provision of information to subjects, medical records, nursing records, clinical laboratory data, imaging films, and other records used as the basis for preparing case reports. Data stored in electronic medical records are also considered as source documents.
2) Records related to the auto-DC/Gal or the iPS-NKT cell administration
3) Documents or records related to the study that are necessary for complying the applicable guidelines.

The following items of data described in the case report form shall be considered source documents (source data). However, if the data are recorded in the medical record, the medical record shall be regarded as source documents (original data).

1) Purpose of concomitant medications/therapy
2) Determination of the extent of adverse events, outcome (including results at follow-up), severity, and causal relationship with the auto-DC/Gal or the iPS-NKT cells
3) Reasons for discontinuation of the subject's study
4) Comments of the PI or SI

15. Preservation of source documents and other records

15.1. Record keeping by the study site

Documents or records pertaining to a study to be preserved at the study site as prescribed in the Regenerative Medicine Act shall be preserved by the hospital director until the later of the following dates.

1) The date on which the auto-DC/Gal or the iPS-NKT cell donor receives approval for manufacture

and marketing of the regenerative medicine product pertaining to the test product (if development is discontinued, the date on which 3 years have passed since the decision to discontinue development was made)

2) The date on which 30 years have elapsed since the discontinuation or termination of the study
3) The PI shall notify the study site or the Specified Authorized Regenerative Medicine Committee and the Bioethics Review Committee when it is no longer necessary to retain the records that should be retained by the study site or the Specified Authorized Regenerative Medicine Committee and the Bioethics Review Committee.

15.2. Record keeping by the PI

Documents or records pertaining to a study that should be preserved by the PI as stipulated in the Regenerative Medicine Act and the Ministerial Ordinances shall be preserved at a storage location deemed appropriate until 30 years have elapsed after the study is discontinued or terminated.

16. Storage period of a part of harvested cells, etc. and a part of cellular processed products used for regenerative medicine

16.1. Storage of samples

Samples will be stored at Chiba University Hospital Center for Frontier Medical Sciences for 10 years after the completion of the study. The storage method is freezing by liquid nitrogen. The storage location will be secured by a locked door at the entrance of the University Hospital and at the Center for Frontier Sciences.

16.2. Disposal of samples

If a subject withdraws consent, if a specimen is mistaken or contaminated or is strongly suspected of being mistaken or contaminated, or if the sample disposal is otherwise needed, the samples will be disposed after deletion of the identical number from the samples.

16.3. Extent of the data use

The information obtained from this study will be shared with RIKEN institute, with whom we are collaborating. In addition, information obtained from this study may be shared with industries for the development of the next and subsequent development of the product.

16.4. Secondary use of samples and data

Surplus specimens will be stored as indicated in 16.1 and may be used for secondary use of samples and information (including proteome, transcriptome, genome and epigenome analyses at Chiba University). In such cases, ethical review and methods for obtaining consent shall follow the corresponding ethical guidelines.

17. Direct access to source documents

The head of the study site and the PI ensure the direct access to all records, including source documents, during monitoring, audits, and investigations by the Specified Authorized Regenerative Medicine Committee and the Bioethics Review Committee or regulatory authorities for ensuring that the study is being conducted appropriately and that the data are sufficiently reliable. The method and timing of direct access will be specified separately in the monitoring plan.

18. Quality Control and Quality Assurance

18.1. Quality Control

1) In the event of any deviation from this study protocol, the PI or SI shall follow the provisions of this study protocol.
2) The PI or SI shall prepare the case report form in accordance with this study protocol.
3) The PI shall ensure that all data and other records in the case report form are accurate and complete.
4) If any of the data in the case report form is inconsistent in any way with the original data, the PI shall prepare and maintain a record explaining the reason for the inconsistency.
5) The PI shall designate a person who is not engaged in the study at the site subject to the monitoring as a monitor and have him/her conduct the monitoring in accordance with the Standard Operating Procedures for Monitoring. The monitor shall confirm the following:
    - The human rights, safety and well-being of the subjects are protected
    - The study is conducted in compliance with the ASRM and its implementing regulations, the latest research protocol, and the Standard Operating Procedures for the study.
    - The data reported by the PI or SI are accurate and complete, and can be verified against the source documents and other study-related records.

The person in charge of data management shall formulate a data management plan in accordance with the separately established standard operating procedures, and shall ensure the quality of the data through quality control at each stage of data handling.

18.2. Quality Assurance

The PI shall make the plan and the SOPs for audit and have responsible person conduct audit in accordance with these documents. The auditor must not be involved in this study, development of the auto-DC/Gal or the iPS-NKT cell product, site staff who conducts this study, or monitor.

19. Ethics and the Act on the Safety of Regenerative Medicine (ASRM)

This study will be conducted in accordance with the "Declaration of Helsinki" and the Act on the Safety of Regenerative Medicine (ASRM). In addition, this study will be conducted in compliance with the study protocol and related SOPs.

In selecting subjects, the PI or SI will carefully consider the appropriateness of asking subjects to participate in the study from the viewpoint of human rights protection and based on the inclusion and exclusion criteria, taking into consideration the subjects' health condition, symptoms, age, gender, ability to consent, dependency on the investigators, etc. The appropriateness of requesting a patient to participate in a clinical trial, including other trials, will be carefully considered.

In addition, a complaint and inquiry desk has been established for subjects in the study, and a system is in place to receive inquiries at any time.

20. Review Committee

Prior to the implementation of this study, the Specified Authorized Regenerative Medicine Committee of the study site, the Bioethics Review Committee, and the Health Sciences Council (Regenerative Medicine Evaluation Subcommittee) will review the ethical, scientific, and appropriateness of the study. This study will be conducted after obtaining approval from the Specified Authorized Regenerative Medicine Committee, the Bioethics Review Committee, and the Health Sciences Council (Regenerative Medicine Evaluation Subcommittee). If the deliberation results of the Specified Authorized Committee for Regenerative Medicine, etc. and the Bioethics Review Committee are "Approval with modifications," the study will be conducted after the protocol, case report form, informed consent form, etc. are modified and approved based on the deliberation results. In addition, the Specified Authorized Regenerative Medicine Committee and the Bioethics Review Committee shall continuously review whether the study is being conducted appropriately at least once a year.

21. Compensation and Insurance

If a subject suffers health problems because of participating in this study, the PI will take necessary and appropriate measures, including the provision of a medical care system for treatment.

The PI shall enroll to a clinical research insurance that will cover any damage to the subject's health (including death) resulting from the study activities during the study period. The insurance period is from

September 1, 2022 to August 31, 2025, including an additional one-year observation period. Insureds eligible for coverage include the PI, SIs, and others involved in the study site.

The clinical research insurance compensates in accordance with the explanation in the informed consent form. However, if the PI or SI is legally liable for compensation to the subject due to negligence in the conduct of the study, the compensation will be applied to the compensation for damages.

22. Study Costs

The costs associated with this study are as follows:

1. The auto-DC/Gal used in this study will be provided by Chiba University Hospital Center for Advanced Medicine, and the iPS-NKT cells will be provided by RIKEN.
2. All medical fees related to the study will be borne by the PI.
3. The medical institution conducting the study will bear the cost of reducing the burden on subjects, based on the "Documents Concerning Payment to Subjects" separately stipulated in the study.

23. Research Funding and Conflicts of Interest

This study will be conducted with the following research funds from the Japan Agency for Medical Research and Development (AMED) Research Project for Practical Application of Regenerative Medicine.

Project name: Clinical research using pluripotent stem cells (iPS/ES cells), somatic stem cells, etc., conducted in accordance with the Act on the Safety of Regenerative Medicine (ASRM), etc. (Research Project for Practical Application of Regenerative Medicine)

Research Head： Akihiko Koseki (National Institute of Physical and Chemical Research (RIKEN))

Chiba University Hospital has signed a subcontract agreement with RIKEN to conduct the study. iPS-NKT cells will be provided by RIKEN.

Before the deliberations of the Specified Authorized Regenerative Medicine Committee and the Bioethics Review Committee, deliberations will be conducted to ensure that conflicts of interest are properly managed, and it will be confirmed that the PIs or SIs and collaborators are not in a state of conflict of interest.

24. Study Registration

The study will be registered in the registry system known as Japan registry of Clinical Trials (jRCT) (https://jrct.niph.go.jp/) prior to obtaining consent from the first subject.

25. Study structure

See Appendix 5 for the structure of this study.

26. References

1. Barbara B, Kevin JH, Richard G et al. Pembrolizumab alone or with chemotherapy versus cetuximab with chemotherapy for recurrent or metastatic squamous cell carcinoma of the head and neck (KEYNOTE-048): a randomized, open-label, phase 3 study. The Lancet. 2019; 10212: 1915-28.
2. Ferris RL, Blumenschein G Jr, Fayette J, Guigay J, Colevas AD, Licitra L, Harrington K, Kasper S, Vokes EE, Even C, Worden F, Saba NF, Iglesias Docampo LC, Haddad R, Rordorf T, Kiyota N, Tahara M, Monga M, Lynch M, Geese WJ, Kopit J, Shaw JW, Gillison ML. Nivolumab for Recurrent Squamous-Cell Carcinoma of the Head and Neck. N Engl J Med. 2016; 375: 1856-1867.
3. Taniguchi M, Harada M, Kojo S, Nakayama T, Wakao H. The regulatory role of Vα14 NKT cells in innate and acquired immune response. Annu Rev Immunol. 2003; 21: 483-513.
4. Kawano T, Cui J, Koezuka Y, Toura I, Kaneko Y, Motoki K, Ueno H, Nakagawa R, Sato H, Kondo E, Koseki H, Taniguchi M. CD1d-restricted and TCR-mediated activation of Vα14 NKT cells by glycosylceramides. Science. 1997; 278: 1626-9.
5. Kawano T, Nakayama T, Kamada N, Kaneko Y, Harada M, Ogura N, Akutsu Y, Motohashi S, Iizasa T, Endo H, Fujisawa T, Shinkai H, Taniguchi M. Antitumor cytotoxicity mediated by ligand-activated human Vα24 NKT cells. Cancer Res. 1999; 59: 5102-5.
6. Taniguchi M, Seino K, Nakayama T. The NKT cell system: bridging innate and acquired immunity. Nat Immunol. 2003; 4: 1164-5.
7. Fujii S, Shimizu K, et al. Innate Vα14+ natural killer T cells mature dendritic cells, leading to strong adaptive immunity. Immunol Rev 2007; 220: 183-98.
8. Ishikawa A, Motohashi S, et al. A phase I study of α-galactosylceramide （KRN7000）-pulsed dendritic cells in patients with advanced and recurrent non-small cell lung cancer. Clin Cancer Res 2005; 11: 1910-7.
9. Motohashi S, Ishikawa A, Ishikawa E, Otsuji M, Iizasa T, Hanaoka H, Shimizu N, Horiguchi S, Okamoto Y, Fujii S, Taniguchi M, Fujisawa T, Nakayama T. A phase I study of in vitro expanded natural killer T cells in patients with advanced and recurrent non-small cell lung cancer. Clin Cancer Res. 2006; 12: 6079-86.
10. Kobayashi K, et al. The effect of radiotherapy on NKT cells in patients with advanced head and neck cancer. Cancer Immunol Immunother 2010; 59: 1503-9.
11. Horiguchi S, Matsuoka T, Okamoto Y et al. Migration of Tumor Antigen-Pulsed Dendritic Cells After Mucosal Administration in the Human Upper Respiratory Tract. J Clin Immunol 2007; 27: 598-604.
12. Kurosaki M, et al. Migration and immunological reaction after the administration of α-GalCer-pulsed antigen-presenting cells into the submucosa of patients with head and neck cancer. Cancer Immunol Immunother. 2011; 60: 207-15.
13. Uchida T, Horiguchi S, Tanaka Y et al. Phase I Study of α-galactosylceramide-pulsed antigen presenting cells administration to the nasal submucosa in unresectable or recurrent head and neck cancer. Cancer Immunol Immunother. 2008; 57: 337-345.
14. Kunii N, Horiguchi S, Motohashi S et al. Combination therapy of in vitro-expanded natural killer T cells

and α-galactosylceramide-pulsed antigen-presenting cells in patients with recurrent head and neck carcinoma. Cancer Sci. 2009; 100: 1092-8.

15. Yamasaki K, et al. Induction of NKT cell-specific immune responses in cancer tissues after NKT cell-targeted adoptive immunotherapy. Clin Immunol. 2011; 138: 255-65.

---

i The National Institute for physical and chemical research. iPS-090-001,002 Manufacturing Diagram (version 3). (2018).

ii The National Institute for physical and chemical research. Setting for the validation point. (2017).

iii Hiroyuki Kanemitsu. Final Report: Toxicity study of single intravenous administration of iPS-NKT cells in nude mice. Bozo Research Center Inc. (2015) N-TT150002.

iv Hidemi Mochizuki. Final Report: Dose-Setting Study of iPS-NKT Cells in Mice. Ina Research Inc. (2017) NB16320.

v iv Hidemi Mochizuki. Final Report: General toxicity study of human iPS-NKT cells using NOG mice. Ina Research Inc. (2018) NB17321.

vi Kota Ito. Final Report: Systemic Toxicity Study of α-Galactosylceramide Pulsed Dendritic Cells (Chiba-NKT) in Two and Four Doses. Research Institute for Compound Safety Inc. 2016. SR15350

vii Masahiko Ito. Final Report: Tumorigenic potential of the iPS-NKT cells tested by soft agar colony formation assay. Bozo Research Center Inc. (2015) N-BT150002

viii Kakushi Ito. Final Report: Evaluation study of tumorigenicity of human NKT-iPS cells using NOG mice. Japan Bioresearch Center Inc. (2017) 370292

ix Kaori Rokkaku. Final Report: Evaluation of tumorigenicity of human NKT-iPS cells using NOG mice. Nissei Vilis Corporation. (2020) 10300.

x Masahiro Mochizuki. Progress Report: Evaluation study of tumorigenicity of human NKT-iPS cells using NOG mice. Bozo Research Center Inc. (2018) OT-170003.

xi Ishikawa, S. Motohashi, E. Ishikawa et al. A Phase I Study of α-Galactosylceramide （KRN7000）– Pulsed Dendritic Cells in Patients with Advanced and Recurrent Non–Small Cell Lung Cancer. Clin. Cancer Res. （2005） 11: 1910-1917.

xii Motohashi S, Nagato K, Kunii N, et al. A Phase I-II Study of α-Galactosylceramide-Pulsed IL-2/GM-CSF-Cultured Peripheral Blood Mononuclear Cells in Patients with Advanced and Recurrent Non-Small Cell Lung Cancer. J. Immunol （2009） 182: 2492-2501

xiii Motohashi S, Ishikawa A, Ishikawa E, et al. A phase I study of in vitro expanded natural killer T cells in patients with advanced and recurrent non-small cell lung cancer. Clin Cancer Res. （2006） 15; 12: 6079-86

xiv Uchida T, Horiguchi S, Tanaka Y, et al. Phase I study of α-galactosylceramide-pulsed antigen presenting cells administration to the nasal submucosa in unresectable or recurrent head and neck cancer. Cancer Immunol. Immunother. （2008） 57: 337-345.

xv Kunii N, Horiguchi S, Motohashi S, et al. Combination therapy of in vitro-expanded natural killer T cells and α-galactosylceramide-pulsed antigen-presenting cells in patients with recurrent head and neck carcinoma. Cancer Sci. （2009） 100: 1092-1098.

xvi Yamasaki K, Horiguchi S, Kurosaki M, et.al. Induction of NKT cell- specific immune responses in cancer tissues after NKT cell-targeted adoptive immunotherapy. Clin. Immunol. 2011; 138: 255–265.

xvii Toyoda, T, Kamata, T, Tanaka, K, et al. Phase II study of α-galactosylceramide-pulsed antigen-presenting cells in patients with advanced or recurrent non-small cell lung cancer. J. Immunother. Cancer 2020; 8(1): e000316.
