# Supplementary material for: A phase I clinical trial to evaluate the tolerability and safety of an allogeneic iPSC-derived iNKT cell and α-GalCer-pulsed autologous DC combination therapy for patients with recurrent and advanced head and neck cancer: A study protocol
Source: PLoS One. 2026 Feb 26;21(2):e0342387. doi: 10.1371/journal.pone.0342387 (PMC12944769; doi:10.1371/journal.pone.0342387)
Supplement: Attachment — Submitted filename: PONE-D-25-33703_Comments_to_the_authors.pdf [file pone.0342387.s007.pdf]

Kurokawa et al. present a study investigating off-the-shelf iPSC-derived invariant NKT (iPSC-iNKT) cells in combination with α-galactosylceramide-pulsed autologous dendritic cells (DC/Gal). The study's design is both innovative and timely, given the growing interest in off-the-shelf cellular therapies and the clinical application of iPSC-derived products. Its findings are expected to make a significant contribution to the field of cancer immunotherapy, with broad implications for the development of allogeneic or off-the-shelf cellular therapies, while also advancing the translation of iNKT cell–based therapies.

At the same time, several aspects of the manuscript could be improved to enhance clarity and readability. Below, I provide detailed suggestions to strengthen the presentation and ensure that the study's rationale, methodology, and outcomes are communicated clearly and effectively.

Major Revisions

Lines 129-132:
During the treatment period, we evaluated the dose limiting toxicity (DLT) from the DC/Gal injection to 14 days after iPSC-iNKT cell administration under hospitalization. The 28-day observation period starts 14 days after administration of the iPSC-iNKT cells, including three visits for the safety evaluation (Fig. 1/2).

> As patients are monitored throughout both treatment and peri-treatment phases, it would be helpful to clarify at the outset that the monitoring period consists of two phases: a **treatment period**, during which patients are hospitalized and closely monitored for dose-limiting toxicities (DLTs), and an **observation period**, during which patients are followed as outpatients with three visits over 28 days.

Lines 213-215:
The outline of this study plan may be revised in the statistical analysis plan if the primary endpoints or the analysis methods are changed.

> This sentence may be perceived as not methodologically nor regulatory-compliant as it stands. I would suggest revising as follows: "Details of the statistical methods may be further specified in the Statistical Analysis Plan (SAP). If any changes to the primary endpoints or analysis methods are required, they will be implemented through a protocol amendment, and the SAP will be updated accordingly"

Lines 223-225:
The purpose of this study is to evaluate the tolerability, safety, and efficacy of the DC/Gal and iPSC-iNKT cell combination therapy. Therefore, a significance test for validation purposes will not be performed.

> **Efficacy**: in the abstract, the authors state that the study aims to "explore the immunological changes that occur following this combination treatment". Therefore, the term "efficacy" could be reconsidered to better reflect the exploratory objective focused on immunological or immune correlates, rather than efficacy assessment per se. Suitable alternatives may include "immune effects" and "tumor response."
>
> **Significance test**: the protocol is meant to describe a non-comparative, exploratory, early-phase study. It is indeed acceptable not to perform formal statistical significance

testing. However, I would suggest stating it more clearly and scientifically, for example: “This study is designed as an exploratory, early-phase evaluation of the safety, tolerability, and exploratory immune changes following treatment with DC/Gal and iPSC-iNKT cell combination therapy. Therefore, no formal hypothesis testing will be performed, and analyses will be descriptive and exploratory in nature.”

Lines 244-247:

This paragraph includes language about CIs and hypothesis testing that may sounds in contradiction with the stated non-comparative design of the study. To improve clarity and use neutral language, I would suggest "This is an exploratory, early-phase study primarily designed to evaluate safety and tolerability. Therefore, no formal hypothesis testing for efficacy is planned and the study is not powered to demonstrate efficacy. Any P values or confidence intervals derived from these analyses will be reported as nominal and exploratory and will not be used to draw definitive conclusions about efficacy."

Lines 250-255:

Please clarify the manufacturing process. The phrase “*was previously manufactured*” implies that no further steps are required beyond product release. In contrast, the phrases “*The PI will order the manufacturing*” and “*incubated for purification*” suggest that additional procedures are needed and that manufacturing prior to the trial start may not have been completed.

Lines 296-298:

These lines would benefit from clarification. Do the authors mean the following: “DLTs are defined as dose-limiting adverse events possibly related to the DC/Gal or iPSC-iNKT cell product that occur during the period referred to as the treatment period. Other adverse events possibly related to the DC/Gal or iPSC-iNKT cell product, occurring during or outside this period, will be summarized separately.”

Line 382:

There are few or no examples

Please clarify whether no or only a few examples have been reported, and provide references as appropriate.

Lines 389-391:

Additionally, this trial protocol can inform future clinical trials using immunological agents in combination with patient-derived adjuvant agents.

This sentence appears to repeat the information stated in lines 386–387. Immunological (non-cell) agents are in fact under investigation in trials combined with patient-derived adjuvant agents (e.g., NCT03294954).

Lines 411-414:

because iPSC-iNKT cells exhibited cytotoxic activity against six tumor cell lines, we did not restrict the tumor pathology type in this trial. Although only head and neck cancer cases were included, iPSC-iNKT cells could be applied to other malignant tumors with accessible tumor-feeding arteries.

The current phrasing is a bit contradictory: it says the trial did not restrict tumor pathology type, but then notes that only head and neck cancers were included. Consider rewording this passage, for example: “Furthermore, as iPSC-iNKT cells demonstrated cytotoxic activity against six distinct tumor cell lines, their mechanism of action is expected to be broadly applicable across malignancies. Nonetheless, for this first-in-human trial, enrollment is restricted to patients with head and neck cancer, but in future studies this approach could be applied to other tumors with readily accessible tumor-feeding arteries for targeted administration.”

Lines 418-419:
Regenerative medicine products are challenging to store long-term, so study materials must be expanded from the working cell bank after each participant’s enrollment.

For clarity, it may be helpful to explain what is meant by the bank. For example: “[from the working cell bank] consisting of intermediate cell products [after each participant’s enrollment]”.

Lines 434-484:
The section begins by stating that there are “several limitations,” but the text that follows largely reads as explanations or justifications rather than clearly stated limitations. To frame the limitations explicitly, they should be presented as constraints of the study design or interpretability, followed by brief reasoning or mitigation, which the authors already provide effectively. Consider including the following before the mitigation statements:

- Limitation 1: This trial will not assess the tolerability, safety, or efficacy of repeated dosing, restricting evaluation of long-term or repeated combination therapy.
- Limitation 2: The study design limits the generalizability of the findings to other malignancies.

Minor Revisions:
General Note:
Across the manuscript, there are several typographical errors and inconsistent use of tenses. Adopting the present tense consistently may improve clarity.

Lines 74-78:
Please consider the following to improve readability:
Unlike other T cell types, iNKTs recognize glycolipids such as alpha-galactosylceramide (αGalCer). When presented by antigen-presenting cells via CD1d, a monomorphic MHC-like molecule, the glycolipid induces rapid iNKT cell activation. Activated iNKT cells can then recognize CD1d-presented lipid antigens on cancer cells, exerting direct cytotoxicity against $CD1d^{+}$ tumor cells regardless of their major histocompatibility complex (MHC) expression, including through release of perforin and granzymes. They can also promote broader antitumor immune responses indirectly harm cancer cells by activating NK cells and cytotoxic T lymphocytes (CTLs) through release of interferon (IFN)-γ and other cytokines.

Lines 78-79:
It appears that the word **“**autologous**”** is missing before “activated iNKT cells.” For accuracy, the passage should read: “In clinical trials targeting non-small cell lung cancer,

intravenously administering autologous activated iNKT cells or αGalCer-pulsed autologous dendritic cells (DC/Gal)”

Lines 85-89

iNKT cells have durable INF- γ productivity and proliferative capacity against conventional radiation therapy.

Consider: “iNKT cells also maintain durable interferon-γ production and proliferative capacity following conventional radiation therapy”

Lines 101-105:

Please include references

Line 108:

Please include references

Lines 126-129

We avoid same-day administration of the investigational product to more than two participants, with a minimum interval of at least seven days before administering the investigational product to the following participant.

For clarity, it would be helpful to indicate the reason behind this, whether it is to allow for safety evaluation prior to subsequent dosing, to ensure adequate monitoring, or for operational feasibility.

Lines 138-139

with one or fewer

consider “≤1” or “no or one”

Lines 203-204:

Patients with completely identical genotypes of HLA-A, HLA-B, and HLA-C to the investigational product

Clarify why, as explained in lines 394-396

Lines 205:

Consider replacing “*patients who are prohibited with blood apheresis because of comorbidities*” with “Patients who are prohibited from undergoing blood apheresis due to comorbidities”

Lines 230-232:

In the target population for DLT evaluation, the number of cases of DLT occurrence per cohort, along with its percentage and 95% confidence interval (CI), will be calculated.

Consider replacing with: “For each cohort, the number and proportion of patients experiencing a DLT, with corresponding 95% confidence intervals, will be calculated”

Lines 265 (Table 1):

Consider rewording “*Characteristic test*” to more specifically reflect the manufacturing requirements of iPSC-iNKTs. As currently written, it may appear redundant with the safety tests listed under the “*Specification test*” category.

Lines 279-280:

Please clarify the rationale for using different methods to assess Mycoplasma and for applying different thresholds when assessing Endotoxin levels in Table 2.

Lines 300:

Grade 4 or higher.

The authors may wish to specify “Grade 4 hematological toxicities” rather than “Grade 4 or higher,” since Grade 5 corresponds to death.

Lines 312-313:

If two or more patients have the DLT, then we will stop this study.

For improved readability, consider rewording as: “If two or more patients experience a DLT at the first dose cohort, the dose will not be escalated and the study will be stopped”

Line 361:

participants need to be hospitalized during the DLT period with intensive observation

For clarity and consistency, it may be helpful to use the terms “treatment period” and “observation period” as previously defined, without introducing a new “*DLT period”*. The line could be reworded as: “Participants need to be hospitalized during the treatment period for intensive observation and prompt identification of DLTs”
