# Supplementary material for: A phase I clinical trial to evaluate the tolerability and safety of an allogeneic iPSC-derived iNKT cell and α-GalCer-pulsed autologous DC combination therapy for patients with recurrent and advanced head and neck cancer: A study protocol
Source: PLoS One. 2026 Feb 26;21(2):e0342387. doi: 10.1371/journal.pone.0342387 (PMC12944769; doi:10.1371/journal.pone.0342387)
Supplement: Attachment — Submitted filename: PONE-D-25-33703R1_Comments_to_the_authors.pdf [file pone.0342387.s009.pdf]

COMMENTS TO THE AUTHORS (PONE-D-25-33703R1)

Kurokawa et al. have addressed all the comments and revised their study protocol accordingly.

We acknowledge and appreciate the authors' efforts to incorporate the suggested revisions. The revised manuscript has improved notably in both clarity for readers and accuracy.

We would suggest three additional final edits to ensure that all sections are sufficiently clear and detailed for readers to appreciate the quality and impact of this important study.

1) Lines 79-81.
   Please insert "*that*" after "*responses*". The complete sentence will read as: They can also promote broader antitumor immune responses *that* indirectly harm cancer cells by activating NK cells and cytotoxic T lymphocytes (CTLs) through release of interferon (IFN)-γ and other cytokines [12-14]

2) Tables 1-2.
   We thank the authors for providing the rationale for the different methods and thresholds used to assess Mycoplasma and Endotoxin. This information may help readers appreciate the implications of generating and applying two distinct products produced under different conditions and in different facilities. We would suggest including this information in the manuscript as brief table footnotes. For example:

   [Table 2 footnote]
   Mycoplasma testing methods are equivalent across products.

   [Table 1 and Table 2 footnote]
   Endotoxin thresholds reflect the validated limit of detection at the manufacturing site.

3) Line 441-443.
   Please complete the sentence with a main clause. One suitable option is provided below: Because the DC/Gal and iPSC-iNKT 442 cells are administered at one time, *this trial will not assess the tolerability, safety, or efficacy of repeated dosing,* restricting evaluation of long-term or repeated 443 combination therapy.

With these final improvements, we recommend the manuscript for publication.
