# Supplementary material for: A phase I clinical trial to evaluate the tolerability and safety of an allogeneic iPSC-derived iNKT cell and α-GalCer-pulsed autologous DC combination therapy for patients with recurrent and advanced head and neck cancer: A study protocol
Source: PLoS One. 2026 Feb 26;21(2):e0342387. doi: 10.1371/journal.pone.0342387 (PMC12944769; doi:10.1371/journal.pone.0342387)
Supplement: Attachment — Submitted filename: Response to Reviewers_the journal requirements.docx [file pone.0342387.s008.docx]

Response to the Reviewers’ Comments and Journal Requirements

Journal Requirements:

When submitting your revision, we need you to address these additional requirements.

1. Please ensure that your manuscript meets PLOS ONE's style requirements, including those for file naming. The PLOS ONE style templates can be found at

https://journals.plos.org/plosone/s/file?id=wjVg/PLOSOne_formatting_sample_main_body.pdf and

https://journals.plos.org/plosone/s/file?id=ba62/PLOSOne_formatting_sample_title_authors_affiliations.pdf

<Response>

We have again confirmed that our manuscript meets PLOS ONE’s style requirements, as described in the templates.

2. Thank you for stating the following in the Competing Interests section:

H. Koseki and S. Motohashi received grants from BrightPath Biotherapeutics Co., Ltd. All other authors declare that they have no relevant conflicts of interest.

Please confirm that this does not alter your adherence to all PLOS ONE policies on sharing data and materials, by including the following statement: "This does not alter our adherence to PLOS ONE policies on sharing data and materials.” (as detailed online in our guide for authors http://journals.plos.org/plosone/s/competing-interests). If there are restrictions on sharing of data and/or materials, please state these. Please note that we cannot proceed with consideration of your article until this information has been declared.

Please include your updated Competing Interests statement in your cover letter; we will change the online submission form on your behalf.

<Response>

We revised the Competing Interests statement in the cover letter as follows:

H. Koseki and S. Motohashi received grants from BrightPath Biotherapeutics Co., Ltd. All other authors declare that they have no relevant conflicts of interest. This does not alter our adherence to PLOS ONE policies on sharing data and materials.

3. We note that you have indicated that there are restrictions to data sharing for this study. For studies involving human research participant data or other sensitive data, we encourage authors to share de-identified or anonymized data. However, when data cannot be publicly shared for ethical reasons, we allow authors to make their data sets available upon request. For information on unacceptable data access restrictions, please see http://journals.plos.org/plosone/s/data-availability#loc-unacceptable-data-access-restrictions.

Before we proceed with your manuscript, please address the following prompts:

a) If there are ethical or legal restrictions on sharing a de-identified data set, please explain them in detail (e.g., data contain potentially identifying or sensitive patient information, data are owned by a third-party organization, etc.) and who has imposed them (e.g., a Research Ethics Committee or Institutional Review Board, etc.). Please also provide contact information for a data access committee, ethics committee, or other institutional body to which data requests may be sent.

b) If there are no restrictions, please upload the minimal anonymized data set necessary to replicate your study findings to a stable, public repository and provide us with the relevant URLs, DOIs, or accession numbers. Please see http://www.bmj.com/content/340/bmj.c181.long for guidelines on how to de-identify and prepare clinical data for publication. For a list of recommended repositories, please see https://journals.plos.org/plosone/s/recommended-repositories. You also have the option of uploading the data as Supporting Information files, but we would recommend depositing data directly to a data repository if possible.

Please update your Data Availability statement in the submission form accordingly.

<Response>

We will adhere to the data sharing policy on your journal, and already stated as follows in the “Data Availability” section:

No datasets were generated or analyzed during the current study. De-identified research data will be made publicly available upon completion and publication of the study.

4. If the reviewer comments include a recommendation to cite specific previously published works, please review and evaluate these publications to determine whether they are relevant and should be cited. There is no requirement to cite these works unless the editor has indicated otherwise.

<Response>

We added one citation as “[21]” on the previous phase I study using iPSC-iNKT cells which has been published just recently.

Reviewer #1:

From a statistical perspective this is very well written protocol.

The protocol indicates (Page 8, lines 135-139) the use of (3 + 3) dose escalating design with two dose levels (Dose 1 and Dose 2. Thus provided the first three patients experience acceptable levels of dose limiting toxicity (DLT) on Dose 1, the next 3 patients will receive Dose 2. This is carefully set out in Figure 3.

The protocol describes carefully (Page 13, lines 244-247) the sensible use of a Confidence Interval at the interpretation stage. I am not sure the P-value will be of much use, but this is a very trivial remark.

<Response>

We thank the reviewer for the valuable comment regarding the P-value. We agree that the confidence interval provides the primary information for interpreting study results. However, we have included P-values as supplementary information to express the degree of incompatibility between the null hypothesis and the data obtained through our study. We believe that presenting both confidence intervals and P-values will make our results accessible to a broader readership while maintaining statistical rigor.

Reviewer #2:

The submitted protocol adheres to international standards for first-in-human studies and provides insight into testing a promising modern cell therapy. I have only a few minor recommendations.

The introduction should explain how iPSC-iNKTs are an improvement over standard peripheral blood iNKTs and how donor variability is reduced. iPSCs come from a healthy donor, so the text should reference published data showing that standardized preparation consistently produces iPSC-iNKTs with a similar phenotype and function.

<Response>

We recently published an article on the previous phase I study using iPSC-iNKTs. In the paper, the specifications of the iPSC-iNKTs were described. So, we will add some description on the specification of iPSC-iNKTs with reference in the introduction part.

At this time, the data has not been published in a paper. However, the relevant data have been compiled as part of the study, including product quality and nonclinical data, in the protocol (Supporting Information, S4). Therefore, we put the S4 as a reference here.

It is unnecessary to list the names of commercial cell lines. Mentioning the broad effect and referencing the publication is sufficient.

<Response>

As mentioned above, the data have not been published in a paper. We will also revise the main text to eliminate redundant descriptions of cell lines and refer readers to the supporting information describing the in vitro tumor suppressive effects.

Within the protocol, I would appreciate a mention that cytokine release syndrome and tumor lysis syndrome are monitored. Although these reactions are not expected, they should be mentioned, along with the markers and how treatment is set up in the case of CRS. Feel free to mention that it is set up according to CAR-T therapy guidelines.

<Response>

As you mentioned, our monitoring plan includes care for CRS and TLS by vigilant monitoring in the hospital during the first 14 days after administration of study treatment. We will consider including a mention on CRS and TLS in the next protocol amendment.

The protocol contains references to tables that do not exist (e.g., Table 20). I believe these are typos in the protocol revisions, but I recommend checking them in future revisions.

<Response>

Thank you for pointing out the typos. We’ll revise the tables in the next protocol amendment.

Overall, the protocol and the study are of high quality.

<Response>

We appreciate your review and mindful advice.

Reviewer#3

**Major Revisions**

Lines 129-132:

During the treatment period, we evaluated the dose-limiting toxicity (DLT) from the DC/Gal

injection to 14 days after iPSC-iNKT cell administration under hospitalization. The 28-day

observation period starts 14 days after administration of the iPSC-iNKT cells, including three

visits for the safety evaluation (Fig. 1/2).

As patients are monitored throughout both treatment and peri-treatment phases, it would be helpful to clarify at the outset that the monitoring period consists of two phases: a treatment period, during which patients are hospitalized and closely monitored for dose-limiting toxicities (DLTs), and an observation period, during which patients are followed as outpatients with three visits over 28 days.

<Response>

I agree with your suggestion. It can be more straight forward.

Lines 213-215:

The outline of this study plan may be revised in the statistical analysis plan if the primary

endpoints or the analysis methods are changed.

This sentence may be perceived as not methodologically nor regulatory-compliant as it

stands. I would suggest revising as follows: “Details of the statistical methods may be

further specified in the Statistical Analysis Plan (SAP). If any changes to the primary

endpoints or analysis methods are required, they will be implemented through a protocol

amendment, and the SAP will be updated accordingly”

<Response>

I agree with your suggestion. It can be more accurate.

Lines 223-225:

The purpose of this study is to evaluate the tolerability, safety, and efficacy of the DC/Gal and iPSC-iNKT cell combination therapy. Therefore, a significance test for validation purposes will not be performed.

Efficacy: in the abstract, the authors state that the study aims to “explore the immunological changes that occur following this combination treatment”. Therefore, the term “efficacy” could be reconsidered to better reflect the exploratory objective focused on immunological or immune correlates, rather than efficacy assessment per se. Suitable alternatives may include “immune effects” and “tumor response.”

Significance test: the protocol is meant to describe a non-comparative, exploratory, early-phase study. It is indeed acceptable not to perform formal statistical significance testing. However, I would suggest stating it more clearly and scientifically, for example:

“This study is designed as an exploratory, early-phase evaluation of the safety,

tolerability, and exploratory immune changes following treatment with DC/Gal and iPSCiNKT cell combination therapy. Therefore, no formal hypothesis testing will be performed, and analyses will be descriptive and exploratory in nature.”

<Response>

I agree and replace the language as you suggested.

Lines 244-247:

This paragraph includes language about CIs and hypothesis testing that may sounds in contradiction with the stated non-comparative design of the study. To improve clarity and use neutral language, I would suggest "This is an exploratory, early-phase study primarily designed to evaluate safety and tolerability. Therefore, no formal hypothesis testing for efficacy is planned and the study is not powered to demonstrate efficacy. Any P values or confidence intervals derived from these analyses will be reported as nominal and exploratory and will not be used to draw definitive conclusions about efficacy."

<Response>

I agree and replace the language as you suggested.

Lines 250-255:

Please clarify the manufacturing process. The phrase “was previously manufactured”

implies that no further steps are required beyond product release. In contrast, the

phrases “The PI will order the manufacturing” and “incubated for purification” suggest

that additional procedures are needed and that manufacturing prior to the trial start may not have been completed.

<Response>

As you mentioned, the word “manufactured” here is inappropriate. We will delete the word.

Lines 296-298:

These lines would benefit from clarification. Do the authors mean the following:

“DLTs are defined as dose-limiting adverse events possibly related to the DC/Gal or

iPSC-iNKT cell product that occurs during the period referred to as the treatment period. Other adverse events possibly related to the DC/Gal or iPSC-iNKT cell product, occurring during or outside this period, will be summarized separately.”

<Response>

Adverse events other than DLTs are not separately summarized, but will be included as “all AEs” regardless of causality, which also includes DLTs. Therefore, we would like to use the first sentence of your proposal. The description on “all AEs” is described in the “Safety measurement” section (Lines 322-325).

Line 382:

There are few or no examples

Please clarify whether no or only a few examples have been reported, and provide

references as appropriate.

<Response>

Thank you for pointing out the misleading part. The intention of this part was to highlight the challenges of clinical trials using regenerative medicines, specifically regarding the combination of autologous cells and allogeneic regenerative therapy. It’s also challenging to determine the prevalence of such a type of clinical trial. Therefore, we’ll replace the sentence as follows:

The iPSC-iNKT cells were established from a healthy volunteer and are stocked as a master cell bank at the RIKEN Institute, while αGalCer/DCs are prepared from participants’ peripheral blood in the study site. The combination use of autologous and allogeneic cells requires careful consideration for a feasible study flow.

Lines 389-391:

Additionally, this trial protocol can inform future clinical trials using immunological agents in combination with patient-derived adjuvant agents.

This sentence appears to repeat the information stated in lines 386–387. Immunological (non-cell) agents are in fact under investigation in trials combined with patient-derived adjuvant agents (e.g., NCT03294954).

<Response>

We intended to highlight that this study protocol would provide insights into organizing challenging clinical trials using separately prepared immune cells. Therefore, we’d like to clarify our intention as follow (underlined: amended language):

Additionally, this trial protocol can contribute to future clinical trials using separately prepared immune cell combinations, such as iPS cell-derived immune cell products and patient-derived adjuvant agents.

Lines 411-414:

because iPSC-iNKT cells exhibited cytotoxic activity against six tumor cell lines, we did not restrict the tumor pathology type in this trial. Although only head and neck cancer cases were included, iPSC-iNKT cells could be applied to other malignant tumors with accessible tumor-feeding arteries.

The current phrasing is a bit contradictory: it says the trial did not restrict tumor

pathology type, but then notes that only head and neck cancers were included. Consider rewording this passage, for example: “Furthermore, as iPSC-iNKT cells demonstrated cytotoxic activity against six distinct tumor cell lines, their mechanism of action is expected to be broadly applicable across malignancies. Nonetheless, for this first-inhuman trial, enrollment is restricted to patients with head and neck cancer, but in future studies this approach could be applied to other tumors with readily accessible tumorfeeding arteries for targeted administration.”

<Response>

I agree and replace the language as you suggested.

Lines 418-419:

Regenerative medicine products are challenging to store long-term, so study materials must be expanded from the working cell bank after each participant’s enrollment.

For clarity, it may be helpful to explain what is meant by the bank. For example: “[from the working cell bank] consisting of intermediate cell products [after each participant’s enrollment]”.

<Response>

I agree and add the language as you suggested.

Lines 434-484:

The section begins by stating that there are “several limitations,” but the text that follows largely reads as explanations or justifications rather than clearly stated limitations. To frame the limitations explicitly, they should be presented as constraints of the study design or interpretability, followed by brief reasoning or mitigation, which the authors already provide effectively. Consider including the following before the mitigation statements:

• Limitation 1: This trial will not assess the tolerability, safety, or efficacy of repeated

dosing, restricting evaluation of long-term or repeated combination therapy.

• Limitation 2: The study design limits the generalizability of the findings to other

malignancies.

<Response>

We agree with your suggestion. We’ll explicitly insert the language on restriction as follows (underlined: amended language):

Line 440-441: Because the GalCer/DCs and iPSC-iNKT cells are administered at one time, restricting evaluation of long-term or repeated combination therapy.

Line 444: The study design limits the generalizability of the findings to other

malignancies. Nevertheless, the iPSC-iNKT cells showed a killing effect not only…

**Minor Revisions:**

General Note:

Across the manuscript, there are several typographical errors and inconsistent use of tenses. Adopting the present tense consistently may improve clarity.

<Response>

I appreciate your careful proofreading. I revised the typos and tenses accordingly.

Lines 74-78:

Please consider the following to improve readability:

Unlike other T cell types, iNKTs recognize glycolipids such as alpha-galactosylceramide (αGalCer). When presented by antigen-presenting cells via CD1d, a monomorphic MHC-like molecule, the glycolipid induces rapid iNKT cell activation. Activated iNKT cells can then recognize CD1d-presented lipid antigens on cancer cells, exerting direct cytotoxicity against CD1d⁺ tumor cells regardless of their major histocompatibility complex (MHC) expression, including through release of perforin and granzymes. They can also promote broader antitumor immune responses indirectly harm cancer cells by activating NK cells and cytotoxic T lymphocytes (CTLs) through release of interferon (IFN)-γ and other cytokines.

<Response>

Thank you for clarifying the anti-tumor mechanism of iNKTs. We’ll adopt your suggestion.

Lines 78-79:

It appears that the word “autologous” is missing before “activated iNKT cells.” For accuracy, the passage should read: “In clinical trials targeting non-small cell lung cancer,intravenously administering autologous activated iNKT cells or GalCer-pulsed

autologous dendritic cells (DC/Gal)”

<Response>

Thank you for pointing this out. We'll put "autologous" before "activated iNKT cells" in the sentence.

Lines 85-89

iNKT cells have durable INF- γ productivity and proliferative capacity against conventional radiation therapy.

Consider: “iNKT cells also maintain durable IFN-γ production and proliferative capacity following conventional radiation therapy”

<Response>

I agree with your suggestion. Thank you very much.

Lines 101-105:

Please include references

<Response>

At this time, the data has not been published in a paper. However, the relevant data have been compiled as part of the study, including product quality and nonclinical data, in the study protocol (Supporting Information, S4). Therefore, we put the S4 as a reference here.

Line 108:

Please include references

<Response>

We have recently been published the results of the preceding clinical trial in Nature Communications. So, we add the reference in the revision.

Lines 126-129

We avoid same-day administration of the investigational product to more than two participants, with a minimum interval of at least seven days before administering the investigational product to the following participant.

For clarity, it would be helpful to indicate the reason behind this, whether it is to allow for safety evaluation prior to subsequent dosing, to ensure adequate monitoring, or for operational feasibility.

<Response>

This provision is established in the IND in Japan to prevent multiple individuals from experiencing acute fatal adverse events on the same day. Regarding the tragic events that occurred in March 2006 during a first-in-human clinical trial for TGN1412, a mAb targeting the T-cell surface protein CD28, PMDA has been requiring to avoid induction of study treatment for more than one participant at the same day on the first-in-human clinical trial. So, we will add the sentence as follows:

This provision is to prevent multiple individuals from experiencing acute fatal adverse events on the same day.

Lines 138-139

with one or fewer

consider “≤1” or “no or one”

<Response>

I will replace the language to “no or one”

Lines 203-204:

Patients with completely identical genotypes of HLA-A, HLA-B, and HLA-C to the investigational product

Clarify why, as explained in lines 394-396

<Response>

We’ll add the sentence as follows (underlined: amended language):

Patients with completely identical genotypes of HLA-A, HLA-B, and HLA-C to the investigational product to distinguish the study product from participants’ iNKT cells.

Lines 205:

Consider replacing “patients who are prohibited with blood apheresis because of

comorbidities” with “Patients who are prohibited from undergoing blood apheresis due to comorbidities”

<Response>

We'll replace the language as you suggested.

Lines 230-232:

In the target population for DLT evaluation, the number of cases of DLT occurrence per cohort, along with its percentage and 95% confidence interval (CI), will be calculated.

Consider replacing with: “For each cohort, the number and proportion of patients

experiencing a DLT, with corresponding 95% confidence intervals, will be calculated”

<Response>

We'll replace the language as you suggested.

Lines 265 (Table 1):

Consider rewording “Characteristic test” to more specifically reflect the manufacturing

requirements of iPSC-iNKTs. As currently written, it may appear redundant with the

safety tests listed under the “Specification test” category.

<Response>

We appreciate the reviewer's valuable suggestion regarding the clarity and specificity of the heading "Characteristic test." We agree that this term, as currently placed under the broader "Specification test" category, may appear redundant with the general "Safety" tests and does not adequately reflect the specific nature of the analyses performed.

The tests currently listed under "Characteristic test" are BSA ELISA and Mouse genome PCR. These tests are specifically designed to assess residual impurities that originate from the manufacturing process, such as bovine serum albumin (BSA) used in the cell culture medium and mouse genomic DNA contamination derived from the potential use of mouse feeder cells during the initial establishment or maintenance of iPSCs. These process-related safety assessments are distinct from the general product safety criteria (e.g., sterility, mycoplasma denial, endotoxin levels) already listed under the general "Safety" category.

To enhance the precision and clarity of Table 1 by focusing on the manufacturing requirements of the iPSC-iNKT product, we have revised the heading as follows:

Change:

• Original Heading: Characteristic test

• Revised Heading: Process-Related Impurity Testing

Lines 279-280:

Please clarify the rationale for using different methods to assess Mycoplasma and for

applying different thresholds when assessing Endotoxin levels in Table 2.

<Response>

The iPS-NKT and DC/Gal are products produced in different cell processing facilities.

1. Since the mycoplasma quantification methods for iPS-NKT and DC/Gal are essentially identical, we would like to unify the name to the NAT, Nucleic acid Amplification Test, method.

2. Endotoxin testing is validated at each facility, and the detection limit varies from facility to facility. Although the thresholds shown in the tables differ, both represent the detection limit for each facility.

Lines 300:

Grade 4 or higher.

The authors may wish to specify “Grade 4 hematological toxicities” rather than “Grade 4 or higher,” since Grade 5 corresponds to death.

<Response>

As you pointed out, Grade 5 corresponds to death. But we consider the death due to hematological toxicities induced by the study product should also be considered as DLT.

Lines 312-313:

If two or more patients have the DLT, then we will stop this study.

For improved readability, consider rewording as: “If two or more patients experience a DLT at the first dose cohort, the dose will not be escalated and the study will be stopped”

<Response>

I agree and replace the language as you suggested.

Line 361:

participants need to be hospitalized during the DLT period with intensive observation

For clarity and consistency, it may be helpful to use the terms “treatment period” and “observation period” as previously defined, without introducing a new “DLT period”. The line could be reworded as: “Participants need to be hospitalized during the treatment period for intensive observation and prompt identification of DLTs”

<Response>

I agree and replace the language as you suggested.
