# Supplementary material for: A phase I clinical trial to evaluate the tolerability and safety of an allogeneic iPSC-derived iNKT cell and α-GalCer-pulsed autologous DC combination therapy for patients with recurrent and advanced head and neck cancer: A study protocol
Source: PLoS One. 2026 Feb 26;21(2):e0342387. doi: 10.1371/journal.pone.0342387 (PMC12944769; doi:10.1371/journal.pone.0342387)
Supplement: Attachment — Submitted filename: Response to Reviewers_260118.docx [file pone.0342387.s010.docx]

Reviewer#3

Dear reviewer,

We sincerely appreciate your additional comment on our protocol paper. We’ve addressed your comments as below.

We would suggest three additional final edits to ensure that all sections are sufficiently clear and detailed for readers to appreciate the quality and impact of this important study.

1) Lines 79-81.

Please insert “that” after “responses”. The complete sentence will read as: "They can also promote broader antitumor immune responses that indirectly harm cancer cells by activating NK cells and cytotoxic T lymphocytes (CTLs) through release of interferon (IFN)-γ and other cytokines [12-14]"

<Response>

The clarity of this sentence has been enhanced by the contribution of your commentary. We revised it as your advice.

2) Tables 1-2.

We thank the authors for providing the rationale for the different methods and thresholds used to assess Mycoplasma and Endotoxin. This information may help readers appreciate the implications of generating and applying two distinct products produced under different conditions and in different facilities. We would suggest including this information in the manuscript as brief table footnotes. For example:

[Table 2 footnote]

"Mycoplasma testing methods are equivalent across products."

[Table 1 and Table 2 footnote]

"Endotoxin thresholds reflect the validated limit of detection at the manufacturing site."

<Response>

As you kindly advised, these footnotes would be helpful for readers. We revised it to add the footnotes as your advice.

3) Line 441-443.

Please complete the sentence with a main clause. One suitable option is provided below:

"Because the DC/Gal and iPSC-iNKT 442 cells are administered at one time, this trial will not assess the tolerability, safety, or efficacy of repeated dosing, restricting evaluation of long-term or repeated 443 combination therapy."

<Response>

The clarity of this sentence has been enhanced by the contribution of your commentary. We revised it as your advice.
